# Phenoxazines with a Phototransferable *N*‑Acetyl Group and Acrylate Linker: Assembly by C–H Activation, Photoconversion to Fluorescent Dyes, Biolabeling, and Super-Resolution Imaging

**DOI:** 10.1021/jacs.6c05772

**Published:** 2026-06-02

**Authors:** Elizaveta Savicheva, Jasmine Hubrich, Taukeer A. Khan, Mariano L. Bossi, Vladimir N. Belov, Lutz Ackermann, Stefan W. Hell

**Affiliations:** † Department of NanoBiophotonics, 28282 Max Planck Institute for Multidisciplinary Sciences (MPI NAT), Göttingen 37077, Germany; ‡ Department of Optical Nanoscopy, 28296 Max Planck Institute for Medical Research (MPI MR), Heidelberg 69120, Germany; § Institut für Organische und Biomolekulare Chemie (IOBC), 9375 Georg-August-Universität Göttingen, Göttingen 37077, Germany; ∥ German Center for Cardiovascular Research (DZHK), Berlin 10875, Germany

## Abstract

Photoactivatable (PA) fluorescent dyes with a reactive group are designated markers used in bioimaging techniques, tracking cellular processes, and observing the nanoscale organization of biological specimens with high spatiotemporal resolution. Conventional (non-PA) phenoxazine dyes are widely used in fluorescence microscopy owing to their high brightness, fluorescence emission in the far-red, and outstanding photostability, which allow their detection down to single molecules. So far, there has been no general synthetic route to PA oxazines with various emission colors and a reactive group. By applying metal-catalyzed C–H activation to symmetric and easily available N^10^-acetylphenoxazines, we demonstrated that this approach represents a powerful tool for the design of PA dyes. Versatile and relatively short syntheses directly involved 3,7-disubstituted-10-acetylphenoxazines or commercial Resazurin (7-hydroxy-10-oxyphenoxazin-3-one) as starting materials. These underwent site-selective Rh- or Ru-catalyzed C^1^–H activation followed by olefination with alkyl acrylates. The presence of the acrylate C=C bond attached to C^1^ in the N^10^-acetylphenoxazine scaffold results in a red shift (ca. 50 nm) in the absorption spectra, provides a site trapping the acetyl group cleaved off upon irradiation, and enables PA above 400 nm. Red-emitting PA oxazines having a CH=CHCONHR group were prepared and delivered to living and fixed cells. The PA probes incorporating HaloTag or BG-PEG amine (for labeling of Halo- or SNAP-Tags) were found to be cell-permeable and provided good images in (two-color) fluorescence microscopy and nanoscopy techniques, such as PALM (Photoactivation Localization Microscopy) and MINFLUX (MINimal FLUXes), based on the activation and detection of single molecules.

## Graphical abstract

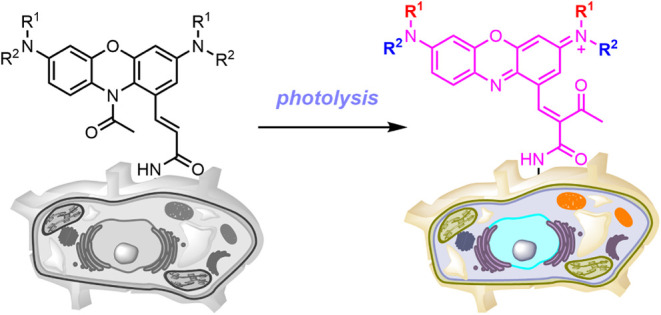

## Introduction

Photoactivatable fluorescent dyes (PA dyes) have emerged as powerful and robust tools for bioimaging techniques, the observation of cellular processes, and the assessment of the nanoscale organization of biological specimens with high spatiotemporal resolution.^1^ Recent developments in microscopy techniques, pushing the boundaries to the single-digit nanometer scale in spatial resolution and submillisecond range in temporal resolution,^2^ elicited new and more stringent demands for engineering new PA fluorophores, which should undergo controllable activation, have the required spectral properties, high photostability, reliable cellular delivery options, and specific targeting procedures. In particular, super-resolution methods put forward such features as sensitive detection (down to single molecules), high signal contrast between an initial dark (*off*) state and the fluorescent photoactivated product (*on* state), and tight control of the activation process to ensure that only a sparse subset of emitters is present in the *on* state at any given moment in time.^3^

The most common strategy to develop a PA dye relies on the incorporation (or attachment) of a photocleavable group into a known fluorescent core^4^ to break the extended conjugation, thus rendering the probe in a nonfluorescent form. Upon photolysis, this group is removed or modified to recover the fluorescent properties of the core. Recent advances in the field of PA dyes are based on the use of *o*-nitrobenzyl,^5^ α-diazoketone,^6^ azide,^7^ and *N*-nitroso^8^ groups as photocleavable residues. These groups have been successfully combined with rhodamine and silicon-rhodamine dyes, but rarely with oxazines, which are attractive candidates for caging due to their high brightness, photostability, and low phototoxicity in living specimens.

Importantly, oxazines possess a small and compact fluorophore with red to far-red emission (a rare feature), detectable in a spectral window where light penetration is relatively high, while autofluorescence and scattering are low. In particular, oxazine dyes Atto 655, Atto 680, and Atto 700 (AttoTec GmbH) have been successfully applied as markers in superresolution fluorescence microscopy.^9^ For non-PA fluorescent dyes, switching between *off* and *on* states is often induced by binding events (DNA-PAINT) or by the addition of complex blinking buffers to induce redox states, as opposed to photoactivation. The photoactivatable oxazine reported in the literature^7c^ contained an “azide and acyl” cage. Notably, PA oxazines possessing one photocleavable residue and a reactive group for binding with the target are missing in the literature. Unlike the rhodamine core, the “natural” oxazine structure lacks the carboxylic acid residue or another functional group suitable for conjugation. Therefore, the synthesis of “reactive” oxazines is often lengthy and tedious because the attachment of the reactive group often requires the reassembly of the fluorophore. Thus, reliable, versatile, and short syntheses leading to photoactivatable and functional oxazines are in high demand.

A promising approach to incorporate reactive groups required for bioconjugation (e.g., carboxylate) may be based on late-stage functionalization (LSF), a stepwise and atom-economical strategy.^10,11^ However, this concept has remained practically unexplored in the domain of fluorescent dyes, with only a few exceptions.^12^ In particular, despite many C–H activation reactions reported for arenes,^10^ the aromatic and heterocyclic systems of oxazines remained uninvolved. Led by the strategy of metal-catalyzed C–H activation, we embarked on the synthesis of PA oxazine dyes. By linking N^10^-acylphenoxazines with acrylates, we demonstrated that the basics of C–H activation can provide a direct entry point to novel PA dyes with asymmetric structures and the required properties. Thus, the synergistic effect and the power of C–H activation have been revealed in the domain of high-performing fluorescent dyes.

Our structures (Scheme 1) combine the N^10^-acetyl residue as a very compact caging group and the neighboring acrylate linker terminating with a functional group (used for further binding with the desired tag). The linker structure and its position were found to be crucial for accelerating the uncaging reaction. The acrylate linker provides auxochromic properties and a docking site to capture the acetyl radical.

**Scheme 1:**
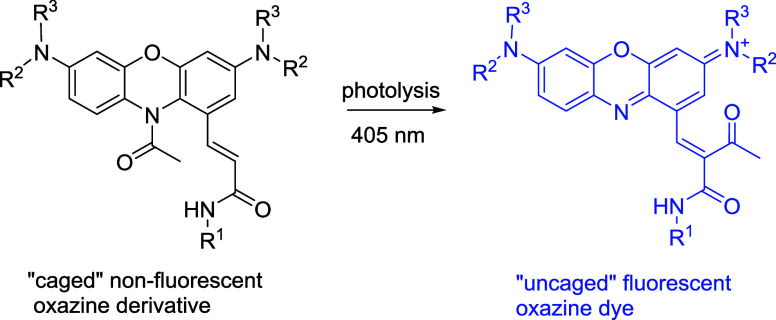
Irradiation with Light Transforms Phenoxazines Introduced in This Work into Fluorescent Products[Fn sch1-fn1]

i R^1^NH represents a tag or labeled biomolecule. Variable substituents R^2^ and R^3^ tune the emission color.

Apart from these features, we found that esters of acrylic acid readily underwent oxidative olefination of symmetric dye cores bearing *N* ^10^-acetyl residues (see the synthesis description in the main text). This methodology reduced the number of synthesis steps to 4 (**2**-H, Scheme 3), 6 (**10**-H-H, Scheme 5), or 5 (**10**-Me-H, Scheme 8) and gave cell-permeable probes capable of efficient intracellular staining. We synthesized various PA oxazines with emission maxima in the orange to far-red spectral region and applied them in confocal and super-resolution fluorescence microscopy based on the detection of single molecules.

## Results and Discussion

### Design of Photoactivatable Oxazines

Acyl groups (acetyl, benzoyl, as well as their brominated analogues) attached to the central (N^10^) nitrogen atom of the *N,N,N′,N′*-tetraethyl-3,7-diaminophenoxazine core (Scheme 2) have been reported to undergo photoinduced homolytic bond cleavage upon irradiation with UV light, followed by oxidation of intermediates to (highly colored and fluorescent) N^10^-deacetyl oxazine and other products.^13^ In organic solvents, the reaction was reported to proceed through the excited singlet state with the formation of oxazine and acetyl radicals and did not require the addition of proton sources (acids; buffer solutions).^13^ The product distribution and the quantum yield of the reaction were found to depend on the irradiation wavelength, the presence of oxygen, and the nature of the acyl group.^13^ In our hands, compound **1**-Me in Scheme 2 (R = Me), when irradiated in acetonitrile solution, showed full conversion to Oxazine 1. When irradiated in aqueous buffer at neutral pH, a ca. 2-fold decrease in the overall rate was observed, while Oxazine **1** and the product **A**-Me (formed upon N^10^-C^1^ acetyl migration in Scheme 2) were detected in a ratio of 3/2 (see also Figure S1; 10% v/v of acetonitrile was added to avoid precipitation). This kind of acyl transfer represents a photochemically induced [1,3] shift.^14^ The limiting step—a homolytic bond break—is so slow in aqueous solutions that this transformation becomes impractical, especially due to the very low absorption of the starting compound **1**-Me above 350 nm. We assumed that this drawback could be overcome by attaching an auxochromic group.

**Scheme 2:**
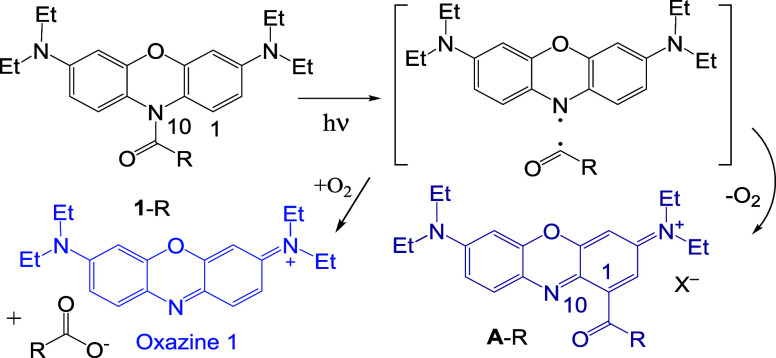
Photolysis of N^10^-Acyl Phenoxazines 1-R in Acetonitrile Solution; R = Me, CBr_3_, C_6_H_5_, 4-BrC_6_H_4_, 3,5-Br_2_C_6_H_3_ [Fn sch2-fn2]

a Reproduced from ref. 13 with permission (Elsevier license 6257160208006).

This group could also serve as a linker for attaching a reactive site that is able to bind with an external target. In contrast to current multistep syntheses of asymmetric oxazine dyes,^15^ the reported routes are shorter and benefit from the advantages of LSF based on C–H activation methods,^11^ with their versatile arsenal of synthesis tools. The control of selectivity is a major challenge in C–H transformations. A way to address this challenge is based on the use of directing groups (DG):^10,11^ innate, naturally occurring functionalities, or temporarily introduced DG. A variety of DGs containing heteroatoms has been developed and is actively in use. Importantly, many organic dyes naturally contain amino groups that can be transformed into DGs, like amides or carbamates.^16^ In particular, we expected that the weakly coordinating N-acetyl group in compound **1**-Me can be used for this purpose (Scheme 3).

**Scheme 3:**
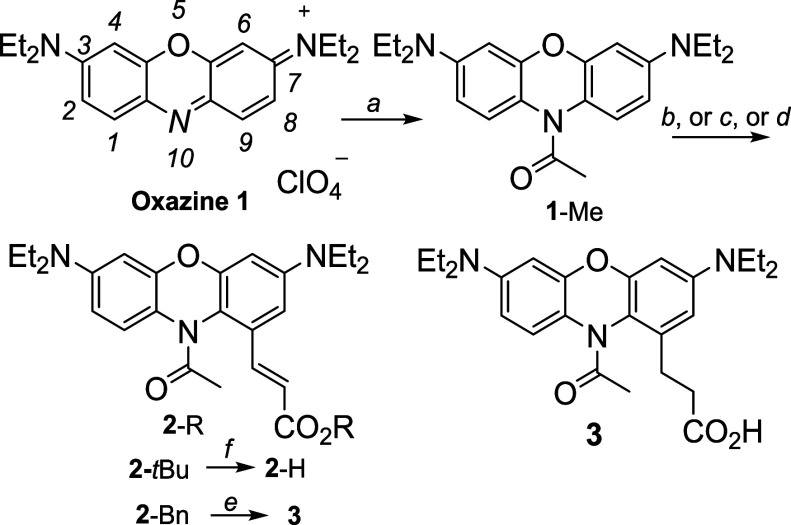
3,7-Bis­(*N,N*-diethylamino)­phenoxazines 2-R and 3 with an N^10^-Acetyl “Photocaging” Group and an Ethenyl or Alkyl Linker to COOH Reactive Site[Fn sch3-fn3]

a Reagents and conditions: a) H_2_, Pd/C, Ac_2_O, r.t., 20 h, 98% (**1**-Me); b) CH_2_=CHCO_2_ *t*Bu, [Ru­(cymene)­Cl_2_]_2_, AgSbF_6_, Cu­(OAc)_2_·H_2_O, acetone, 70 °C, 18 h, 13% (**2**-*t*Bu); c) CH_2_=CHCO_2_ *t*Bu, [RhCp*­(MeCN)_3_]­(SbF_6_)_2_, Cu­(OAc)_2_, acetone, 70 °C, 18 h, 46% (**2**-*t*Bu); d) CH_2_=CHCO_2_Bn, [Ru­(cymene)­Cl_2_]_2_, AgSbF_6_, Cu­(OAc)_2_·H_2_O, acetone, 70 °C, 18 h, 19% (**2**-Bn); e) H_2_, Pd/C, EtOAc, r.t., 20 h, 98% (**3**); f) TFA in DCM 20% v/v, 0 °C–r.t., 6 h, 70% (**2**-H).

To proceed with the synthesis based on the C–H activation strategy, we considered oxidative olefination of compound **1**-Me with acrylates, catalyzed by complexes of ruthenium,^17^ rhodium,^18^ and palladium.^19^ In contrast to esters of acrylic acid (Scheme 3), *N*-(2-hydroxyethyl)­acrylamide, monoethyl itaconate, and *tert*-butyl 3-butenoate were found to be unreactive toward compound **1**-Me under the conditions given in Scheme 3. The transformations shown in Scheme 3 are discussed in the synthesis section and Supporting Information. The direct connection of an acrylate (via terminal methylene (CH_2_−) residue) with the dye core expands the conjugated system, induces the desired bathochromic shift, and enhances the dye’s absorption at or close to the visible range. Acrylates provide a reactive carboxylate group required for further modifications described in the text. The CH=CH bond in compound **2**-R (Scheme 3) can be reduced (e.g., to compound **3**), making the linker more flexible. A comparison of the absorption of compounds **1**-Me, **2**-H, and **3** demonstrates that the presence of the double bond as an auxochrome is advantageous (if not mandatory) for the photolysis. Compounds **1**-Me and **3** have an absorption maximum at 270–280 nm, with a shoulder at 320 nm, without considerable absorption beyond 350–360 nm. In contrast, alkene **2**-H presents a second band at 370 nm, which extends the absorption into the visible range, well beyond 400 nm (Figure S2). In line with these observations, a much faster and more complete activation (by irradiation at 365 nm) was found for acrylate derivative **2**-H in aqueous buffer, in comparison with 3-arylpropionate **3** (Figure S3). Monitoring the photolysis of compound **2**-H shows a gradual bathochromic shift in the emission and markedly nonmonoexponential behavior of the absorption transient (Figure S3), suggesting consecutive reactions. The literature data^13^ and the results of LCMS analysis of the products formed in acetonitrile and aqueous buffer (Figure S4) are consistent with the mechanism shown in Scheme 4 for acrylic acids (e.g., **2**-H) and primary amides (Scheme 1). The acetyl radical is formed in the course of CH_3_CO-N^10^ bond cleavage.^13^ For both structures (**A** and **B** in Scheme 4), the acetyl residue can undergo a facile 1,5-shift and promptly add to the C=C bond in the acrylate chain. The following 1,5-hydrogen shift (from COOH) and electron transfer render intermediates. For the carboxylic acid, the intermediate undergoes decarboxylation followed by photooxidation of the saturated linker, which restores the C=C bond and accounts for the observed red-shifted emission of the final product (Scheme 4). For amides, decarboxylation is impossible, and photooxidation of the intermediate 3-oxoamide takes place. The preparative photolysis of compound **2**-H in acetonitrile (Figure S4) gave the α,β-unsaturated ketone shown in Scheme 4 which was isolated, and its structure was confirmed by NMR spectra. To further explore whether this caging concept is applicable to phenoxazines with emission expanded over a broader spectral window, we prepared compounds **8a** and **10**-H-H with primary and *N*-methyl amino groups, respectively. The synthesis routes are given in Scheme 5 and discussed in the synthesis section (at the end of the main text) and in Supporting Information. Compounds **8a** and **10**-H-H undergo smooth photolysis, with *N*-acetyl migration to the C=C bond (1,5-shift) and decarboxylation (Scheme 6). The same kind of reactivity pattern is observed for compound **2**-H (Scheme 4), with small differences in reaction rates (Figure S5). Traces of the nondecarboxylated product were detected for compound **10**-H-H. To further validate the role of the linker attached to C^1^, enabling facile 1,5-migration of the acetyl radical (eventually, inside the solvent cage), we prepared and photolyzed compounds **8b** and **8c** with the *tert*-butyl acrylate linker attached to C^2^ and C^4^, respectively (Scheme 5). In clear contrast to compound **8a,** positional isomers **8b** and **8c** failed to produce dye products upon photolysis, despite the presence of an auxochrome providing an absorption band around 350 nm (Figure S6). Thus, the presence of the acrylate linker attached to C^1^ (nearby N^10^-acetyl group) is crucial for PA. Beyond acting as an auxochrome (Figure S2), it facilitates the photocleavage reaction by capturing the acetyl radical, reducing the probability of N^10^-C^1^ acetyl migration (Scheme 2).

**Scheme 4:**
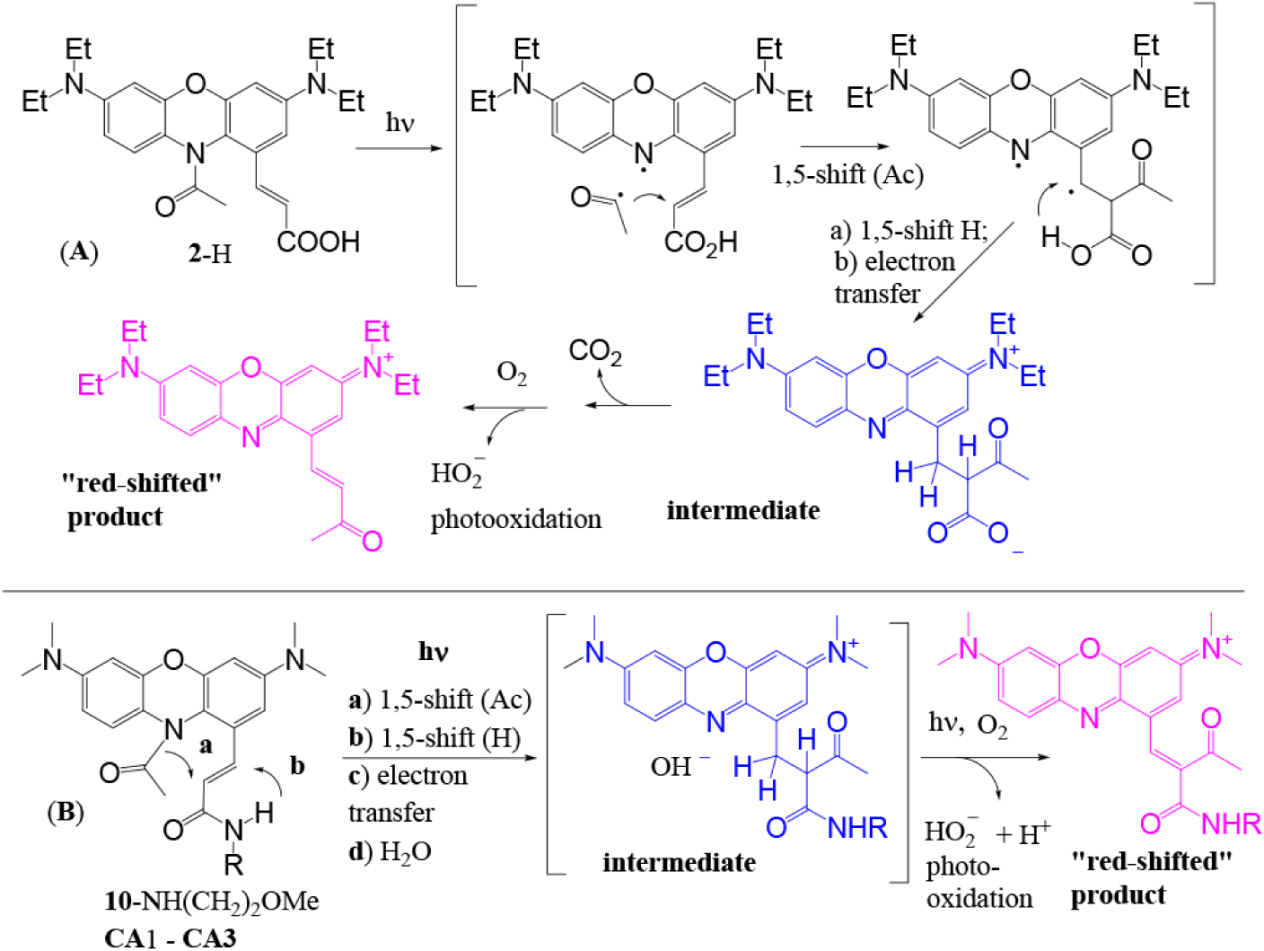
Proposed Mechanisms of Photolysis of Carboxylic Acids (A) and Primary Amides (B, Scheme 1)­[Fn sch4-fn4]

a For acrylic acids, decarboxylation of the intermediate followed by photooxidation to the “red-shifted” product was proposed by the reviewer.

**Scheme 5:**
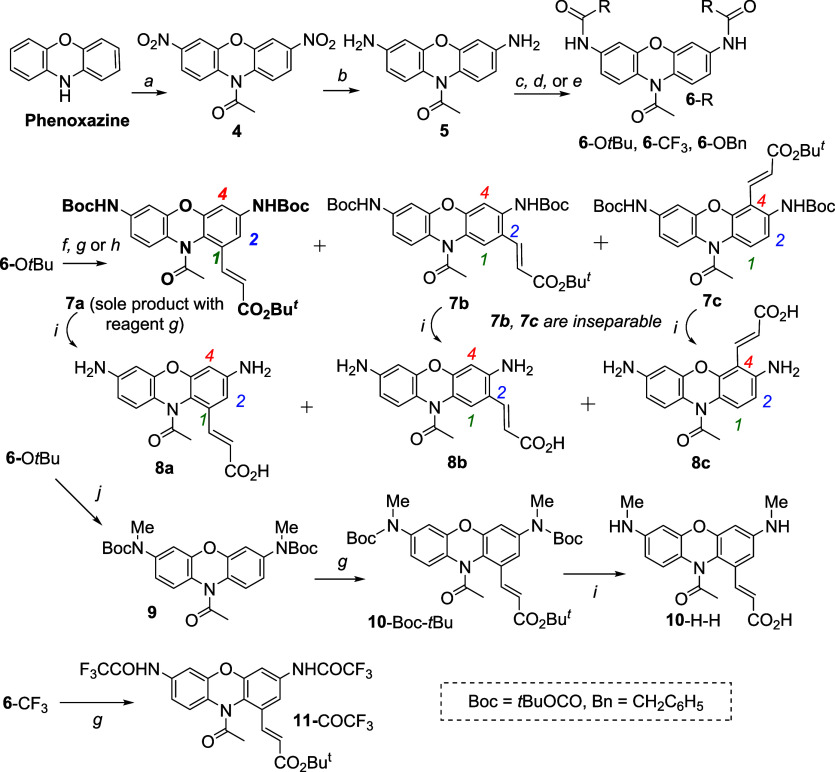
Synthetic Routes to Functionalized 3,7-Diamino Phenoxazines[Fn sch5-fn5]

a Reagents and conditions: a) NaNO_2_, AcOH, acetone, r.t., 72 h, then Ac_2_O, reflux, 3 h, 50% (**4**); b) H_2_, Pd/C, EtOAc, r.t., 20 h, 94% (**5**); c) Boc_2_O, EtOH, r.t., 18 h, 86% (**6**-O*t*Bu); d) TFAA, Et_3_N, DCM, 0 °C–r.t., 1 h, 70% (**6**-CF_3_); e) BnOCOCl, py, DCM, 0 °C–r.t., 4 h, 36% (**6**-OBn); f) CH_2_=CHCO_2_ *t*Bu, [Ru­(cymene)­Cl_2_]_2_, AgSbF_6_, Cu­(OAc)_2_·H_2_O, acetone, 70 °C, 18 h, 34% (**7a**), 20% (**7b**); g) CH_2_ = CHCO_2_ *t*Bu, [RhCp*­(MeCN)_3_]­(SbF_6_)_2_, Cu­(OAc)_2_, acetone, 70 °C, 18 h, 46% (**7a**), 47% (**10**-Boc-*t*Bu), 38% (**11**-COCF_3_); (h) CH_2_ = CHCO_2_ *t*Bu, [Cp*RhCl_2_]_2_, AgSbF_6_, Cu­(OAc)_2_·H_2_O, acetone, 70 °C, 18 h, 14% (**7a**), 49% (inseparable mixture of **7b** and **7c**); (i) TFA in DCM 20% v/v, 0 °C–r.t., 12 h, 61% (**8a**), 20% (**8b**), 18% (**8c**), 81% (**10**-H-H); j) NaH, MeI, THF, 0 °C–r.t., 2 h, 33% (**9**).

**Scheme 6:**
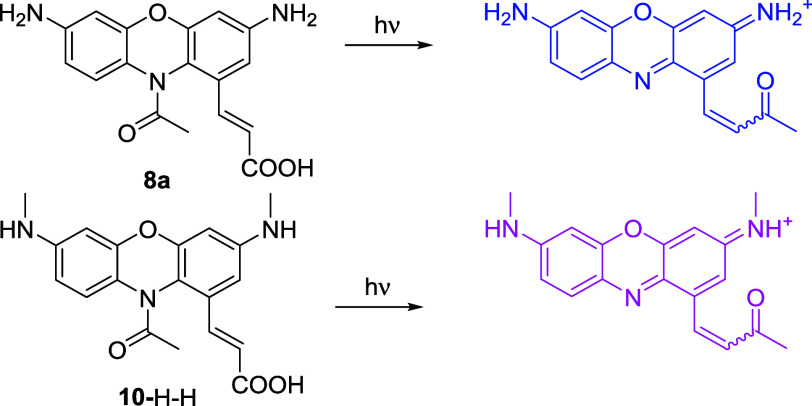
Photolysis of *N,N’*-Unsubstituted and *N,N’*-Bis­(methylamino) Derivatives 8a and 10-H-H Leads to Orange- and Red-Emitting Dyes[Fn sch6-fn6]

i See Figure S5 for details.

We assumed that converting the carboxylic acid residue into a poorer leaving group would prevent decarboxylation. Thus, we transformed carboxylic acid **10**-H-H into amide **10**-H-NH­(CH_2_)_2_OMe (Scheme 7) and studied its photolysis (Figure S7). The photolysis of acrylamide **10**-H-NH­(CH_2_)_2_OMe is approximately three times slower than the photolysis of acid **10**-H-H (compare Figures S5 and S7). As expected, the acetyl radical was trapped, and no decarboxylation products were observed. As a minor product (Figure S7), we detected a compound with a molecular mass corresponding to the intermediate with a saturated linker. This observation supports the mechanism proposed in Scheme 4 for amides (path **B**). Encouraged by these results, and using dye cores providing different emission colors upon photoactivation, we prepared amides **8a**-Halo, **10**-H-Halo, **2**-Halo, **10**-Me-Halo (**CA1-CA4**), and **10**-Me-NH-PEG-BG (see Scheme 7). Amides **CA1-CA4** contain an ω-chloroalkane residue as the ligand, enabling the selective, rapid, and irreversible (covalent) labeling of HaloTag fusion proteins, a well-established tool for targeting synthetic fluorophores to specific proteins in live cells.^20a^ Compound **10**-Me-NH-PEG-BG represents a PA probe for labeling SNAP-Tag fusion proteins.^20b^ *N,N,N’,N’*-Tetramethyl analog (**10**-Me-Halo) was intended to improve cell permeability and provide somewhat brighter emission upon photolysis compared with *N,N,N’,N’-*tetraethyl analog (**2**-Halo).

**Scheme 7:**
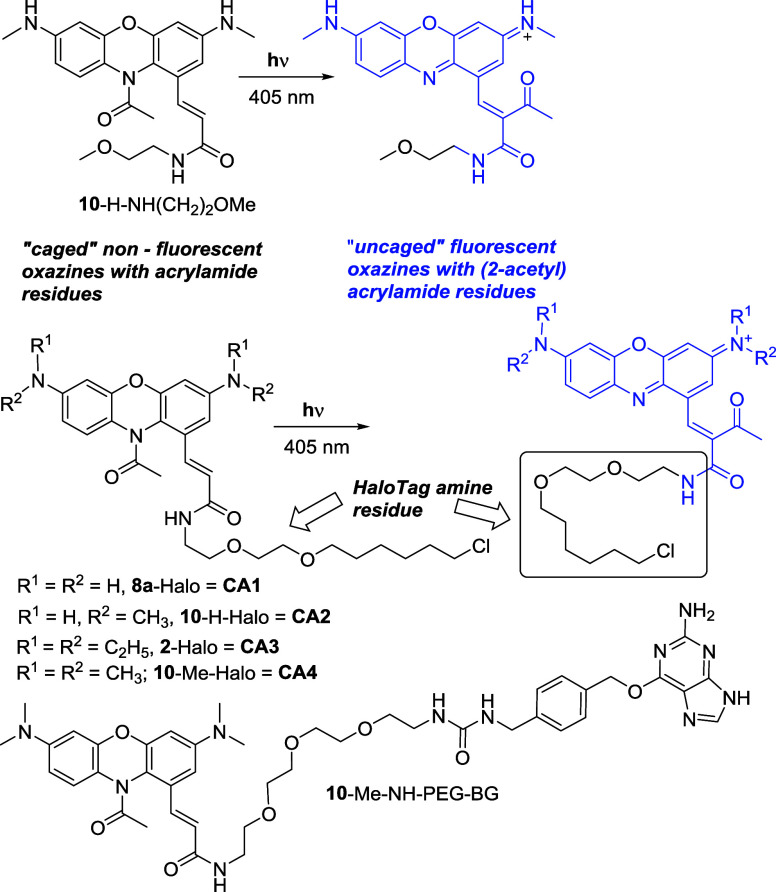
*N*-(ω-Chloroalkyl) Amides 8a-Halo, 10-H-Halo, 2-Halo, 10-Me-Halo (CA1-CA)— Ligands for HaloTag Protein Labeling—and Amide 10-Me-NH-PEG-BG for SNAP-Tag Labeling[Fn sch7-fn7]

a Probes **CA1–CA4** were prepared from Halo-Tag amine and carboxylic acids **8a**, **10**-H-H, **2**-H, and **10**-Me-H, respectively. For photolysis results, see Figures 1 (**CA4** = **10**-Me-Halo), Figure S8 (**CA1** = **8a**-Halo, **CA2** = **10**-H-Halo, **CA3** = **2**-Halo), and Supporting Information. For the synthesis of compound **10**-Me-H, see Scheme 8 and Supporting Information.

We first studied the photoactivation of these four ω-chloroalkane derivatives, **CA1-CA4,** in a free state and bound to HaloTag (HT7) protein (Figures 1 and S8). These experiments confirmed that their photochemical behavior is similar to that of the model compound **10**-NH­(CH_2_)_2_OMe. The amide bond cleavage (detachment of the dye) was not observed for amides **8a**-Halo, **10**-H-Halo, **2**-Halo, **10**-Me-Halo (**CA1-CA4** in Figures 1C and S8), and the protein conjugate retained the fluorescent label upon photolysis (Figure 1G). Fortunately, upon binding to the HT7 protein, we observed a considerable increase in photoactivation rates (Figure 1E) and longer fluorescence lifetimes of the photouncaged oxazines (Figure S9). The photophysical properties of compounds **8a**-Halo, **10**-H-Halo, **2**-Halo, **10**-Me-Halo are summarized in Table 1. The photoactivation quantum yields are relatively low. However, these values are in the same range as those reported for other photoactivatable fluorescent dyes (with various photocleavable residues).^5–8^

**Table 1:** Photophysical Properties of the Fluorescent Products Obtained Upon Photolysis of Amides CA1-CA4, Bound with HaloTag HT7 Protein and Measured in Aqueous Phosphate Buffer (100 mM) at pH = 7.0

**Compound**	***λ***_**abs**_ **max (nm)**	***λ***_**em**_ **max (nm)**	***ϕ***_**act**_ ^a^ **×10** ^**3**^	***Φ***_**fluo**_	***τ***_**fluo**_ **(ns)**
**CA1** (**8a-**Halo)	593	602	1.2	0.24	2.8
**CA2** (**10**-H-Halo)	614	635	0.8	0.31	2.7
**CA3** (**2**-Halo)	657	672	1.7	0.10	1.1
**CA4** (**10**-Me-Halo)	650	672	2.6	0.10	1.1

a Irradiation performed at 365nm.

**Figure 1:**
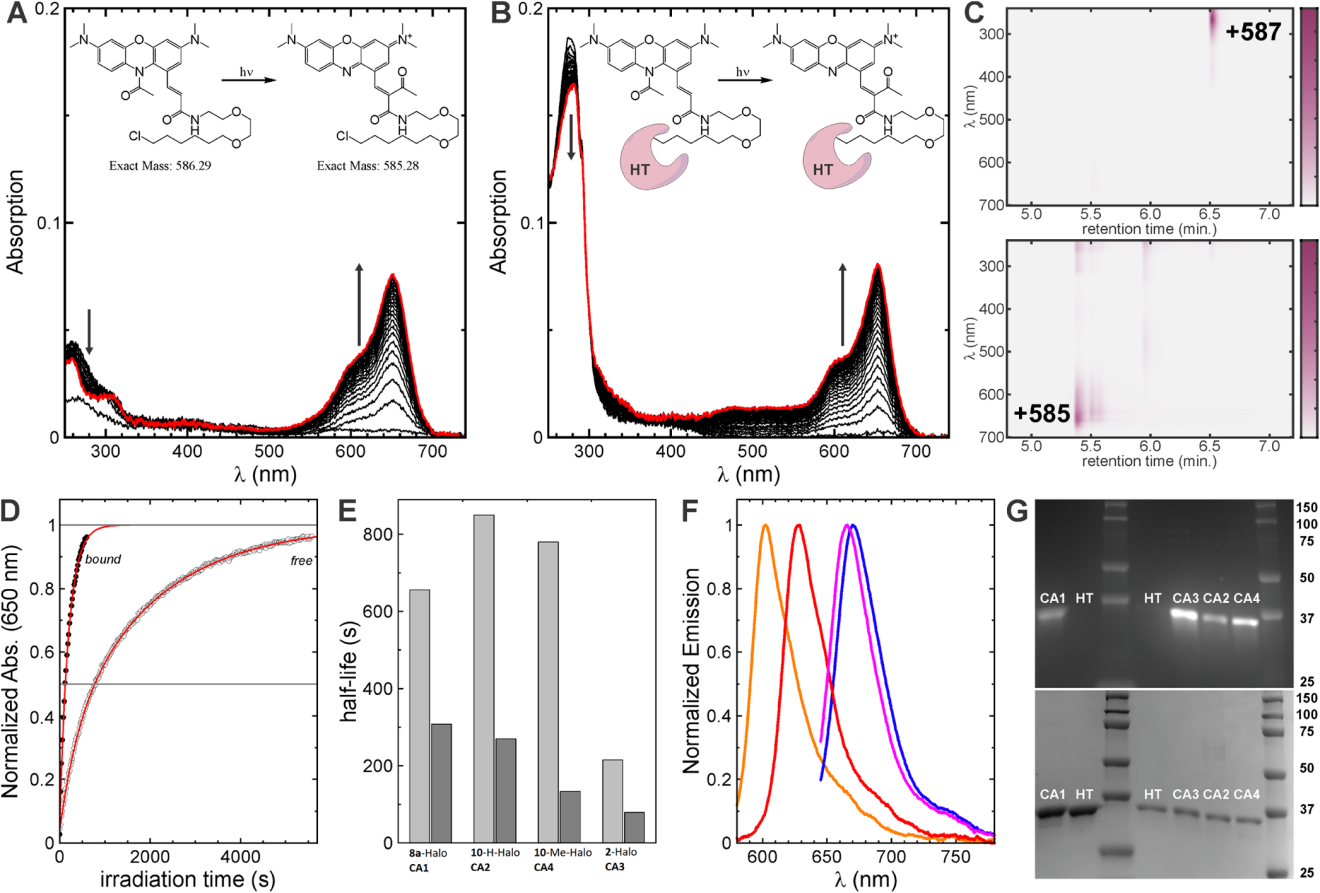
Absorption changes upon photoactivation of compound **10**-Me-Halo = **CA4** in buffered aqueous solutions (100 mM phosphate buffer, pH = 7) in a free state (A) and after binding with HaloTag HT7 protein (B). The LCMS plots of the solution before (top) and after (bottom) photoactivation of compound **10**-Me-Halo; the structure and the molecular masses (as M+H, or M+) of the starting compound **CA4** and the photolysis product are indicated in (A, C). (D) Transients obtained at 650 nm from photoactivation in cases (A) and (B). (E) Half-lives for the activation reactions of compounds **8a**-Halo, **10**-H-Halo, **2**-Halo, and **10**-Me-Halo (**CA1**, **CA2**, **CA3,** and **CA4**, respectively) in a free state (light gray bars) and bound to HT7 protein (dark gray bars), showing acceleration of the photolysis upon covalent binding with HT7 protein for all compounds. (F) Emission spectra of photoactivated oxazines bound to the protein (**8a**-Halo in orange, **10**-H-Halo in red, **2**-Halo in blue, and **10**-Me-Halo in magenta). (G) SDS-page gels of the solutions obtained after the photoactivation detected by fluorescence (top) and after Coomassie staining (bottom).

### Biolabeling and Imaging with Photocaged Phenoxazines

To evaluate the applicability of photoactivatable *N*-acetylphenoxazines for live-cell imaging, we specifically designed a plasmid (Figure S10) to express a HaloTag7 fused to Sec61β, one of the components of the translocation apparatus of the endoplasmic reticulum (ER) membrane. The U2OS cells transfected with the plasmid were incubated in the cell culture medium containing amides **CA1-CA4**, then washed and imaged using a confocal microscope (Figure 2).

**Figure 2:**
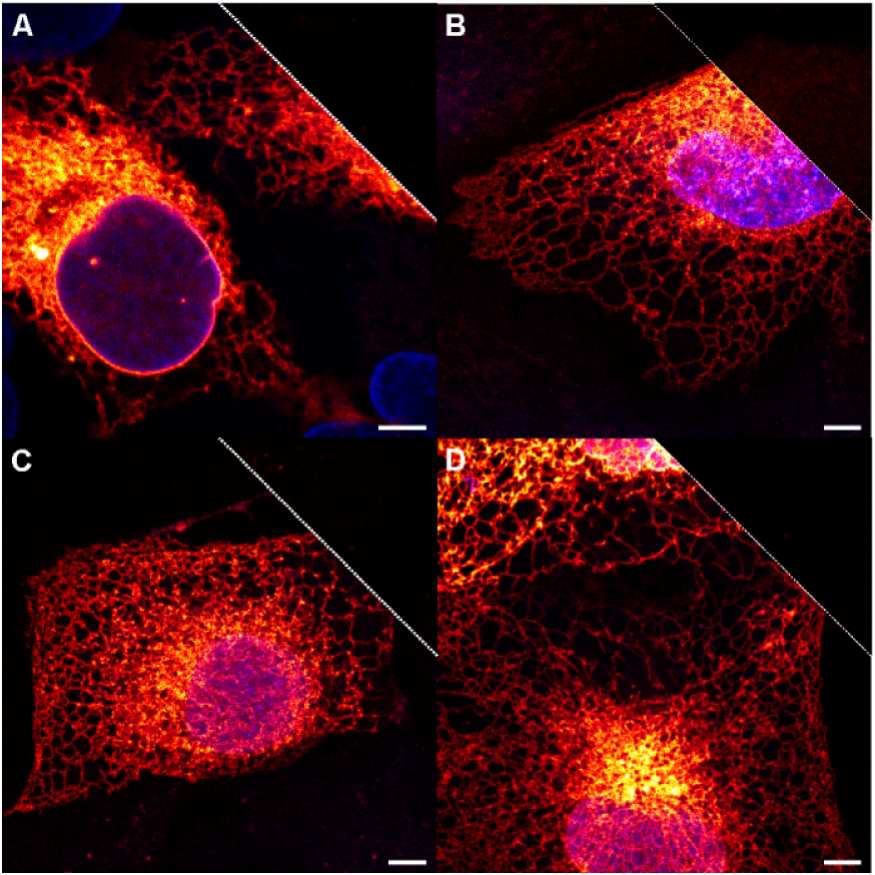
Confocal images of live U-2 OS cells expressing Halo7-Sec61β, labeled with compounds **2**-Halo (A), **10**-Me-Halo (B), **10**-H-Halo (C), and **8a**-Halo (D), and then irradiated with 405 nm light. Staining was performed with 1 μM solutions, except for **2**-Halo (250 nM). Cells were washed and imaged in supplemented FluoroBrite DMEM medium. Images before photoactivation are displayed in the top-right corners. Samples were additionally stained with a blue nuclear stain marker. Scale bars: 5 μm.

We observed high labeling specificity for all amides and a large contrast between the nonemissive “dark” *N*-acetyl-phenoxazines and the bright fluorescent products revealed upon photoactivation. PA was achieved by scanning with a 405 nm laser. Activation rates in the confocal microscope (Figure S11) were similar to those observed in cuvette experiments (Figure 1E). Similar results were obtained when other structures were labeled; for example, mitochondria (Tomm20), vimentin, tubulin (Cep41), and lamin (lamin A/C) (Figure S12). We found that amide **CA3** (**2**-Halo) has a slight tendency to (unspecifically) accumulate in mitochondria (Figure S13) when applied at a concentration of 1 μM. Thus, for live-cell imaging, the probe **CA3** was used at a lower concentration (250 nM).

To fully exploit the imaging performance of N-acetylphenoxazines, we applied them in super-resolution microscopy. Many organelles and subcellular structures are highly dynamic, and their movement and reorganization are faster than the image acquisition time of superresolution methods (being in general slower than conventional confocal microscopy). Therefore, we visualized the ER network by time-lapse confocal imaging on U2OS cells expressing Halo7-Sec61β protein (under conditions used in Figure 2), at a rate of 0.5 Hz. The pronounced changes in the ER structure (Figure S14) are clearly revealed, in line with other reports of active remodeling of the tubules at second and subsecond time scales.^21^ Therefore, super-resolution imaging was performed on fixed cells. Importantly, images were acquired in aqueous PBS as the mounting medium and without any special additives. We first acquired PALM images using a commercial microscope equipped with 560 nm (**8a**-Halo) or 640 nm (**10**-H-Halo, **2**-Halo, **10**-Me-Halo) excitation sources and 405 nm activation. All compounds showed balanced and controllable responses to PA, which were adjusted during the measurement to keep a sparse distribution of activated single emitters. Upon activation, the single emitters produced a large number of photons (≈800–1700 on average; see Figure S15), yielding sharp super-resolution images (Figure 3). Despite the fact that the emission maximum of **10**-H-Halo probe falls between two detection windows, the number of photons detected per single molecule and the calculated mean uncertainty of localization do not strongly differ from the values found for other compounds.

**Figure 3:**
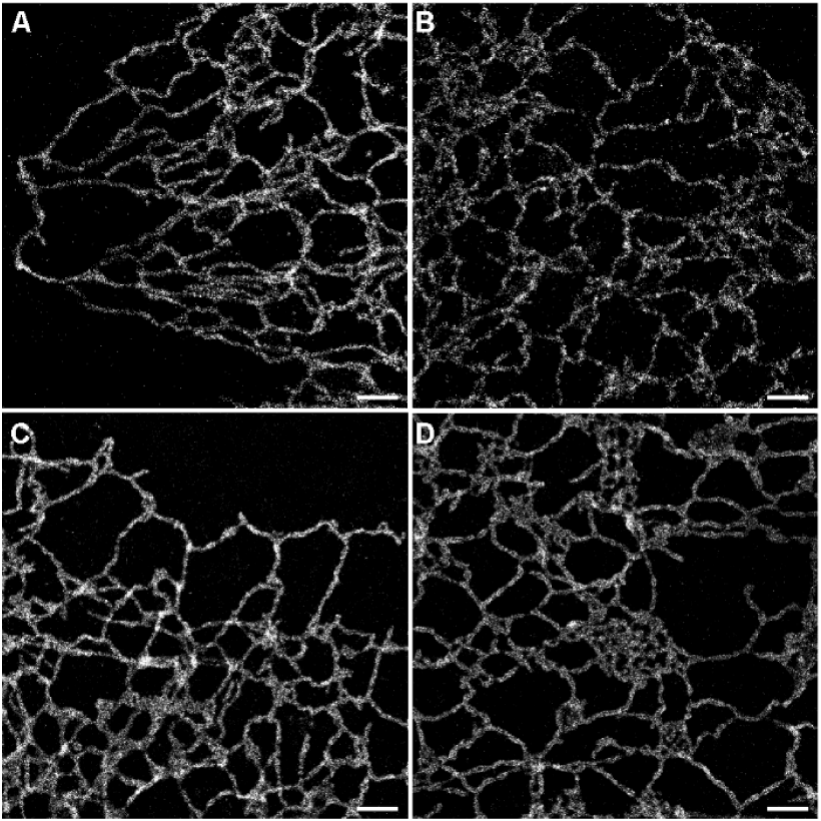
Super-resolution PALM images of fixed U-2 OS cells expressing Halo7-Sec61β, labeled with amides **8a**-Halo (A), **10**-H-Halo (B), **2**-Halo (C), and **10**-Me-Halo (D). Staining was performed on live cells with 1 μM solutions, except for **2**-Halo (250 nM). Imaging was performed on samples mounted in aqueous PBS buffer. Scale bars: 2 μm.

Thus, the utility of the **10**-H-Halo probe is not impaired. To benefit from the different emission properties of four dyes (Figure 1F and Table 1), we demonstrated the possibility to expand their application into two-color imaging. Based on their performance and spectral properties, we selected **8a**-Halo (Scheme 7) for the “orange channel” (560 nm excitation) and **10**-Me-Halo for the “red channel” (640 nm excitation). For the selective labeling of two separate structures, we used the **8a**-Halo probe and prepared the amino-reactive analog (**10**-Me-NHS, Scheme 5) of the probe **10**-Me-Halo (with the same dye core) and used it for antibody labeling. Staining with **8a-**Halo probe on live cells was followed by fixation and immunostaining with a secondary antibody labeled with compound **10**-Me-NHS. As model structures, we selected Tomm20—a protein of the translocase of the outer membrane complex located on the mitochondrial membrane—and a primary antibody against mitochondrial DNA. Confocal microscopy provided images with high contrast in both channels observed before and after photoactivation, as well as good color separation of the structures (Figure 4A–D). Importantly, the same pair of probes allowed for simultaneous two-channel imaging based on single-molecule localization (Figure 4E–G). A histogram of the evolution of the localizations over frames discriminated by channel (Figure S16) evidenced the different responses to the activation light (405 nm), in agreement with the activation quantum yields (Table 1) and the trends shown by these two compounds in other experiments (see Figures 1E and S11). This imaging approach can be readily extended to other structures (e.g., vimentin and NPC; see Figures S17 and S18). Moreover, we demonstrate the extension to two-color live-cell imaging by combining two orthogonal self-labeling enzymes, Halo- and SNAP-Tag.^22^ To this end, we synthesized **10**-Me-NH-PEG-BG (SNAP) (Scheme 7) and, in combination with **8a**-Halo (Halo), simultaneously labeled and imaged live cells stably expressing Mito-SNAP (mitochondrial OMP25 protein fused to SNAP-Tag) and ER-Halo (CalR and KDEL proteins fused to Halo-Tag) (Figure S19). Two-color images in Figures S17–S19 demonstrate specific labeling, clean signal separation between acquisition channels, and high contrast.

**Figure 4:**
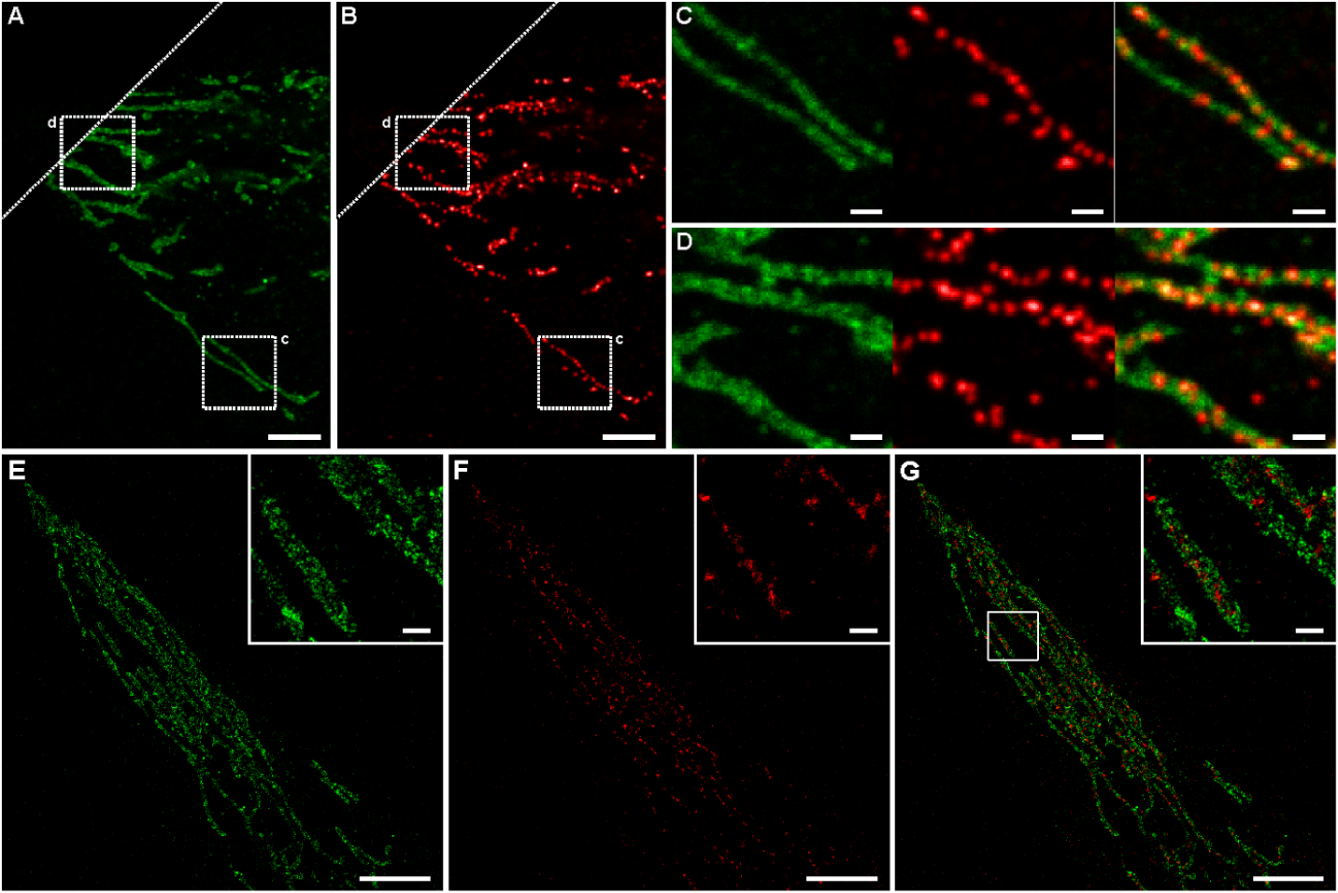
Two-color imaging on fixed cells. Tomm20-Halo cells costained with **8a**-Halo (live-cell labeling) and a combination of a primary antibody against dsDNA and a secondary antibody labeled with **10**-Me-NHS, and imaged in a confocal microscope (A-D) or a camera-based (PALM) superresolution microscope (E-G). (A) Confocal green channel (**8a**-Halo on Tom20), (B) Confocal red channel (**10**-Me-NHS on dsDNA). Upper left corners show the images before activation, and the ROIs indicated are enlarged in (C) and (D), respectively. PALM green channel (**8a**-Halo on Tom20), (F) PALM red channel (**10**-Me-NHS on dsDNA). The inset shows the enlarged ROI indicated in (G). Scale bars: 5 μm (A-B and E-G), 1 μm (C-D), 500 nm (insets in E-G).

We finally evaluated the possibility of using caged phenoxazines in MINFLUX nanoscopy, a technique with remarkably superior spatiotemporal precision compared to camera-based methods.^23^ Unlike the purely stochastic methods of super-resolution microscopy, MINFLUX uses an engineered excitation beam containing a nodal point (typically doughnut-shaped).^2^ MINFLUX combines a deterministic or targeted approach to localize fluorophores with a stochastic search and *on*-switching (photoactivation, in this case) of the probes. Samples prepared with the same protocol used for imaging by means of a camera-based method (Figure 3) were imaged in a commercial MINFLUX microscope equipped with 560 and 640 nm excitation beams.

We selected **CA1** (**8a**-Halo) and **CA4** (**10**-Me-Halo) probes based on their best performance and spectral match to the available channels. Indeed, we observed the distribution and clustering of the Sec61b protein in the ER membrane with unprecedented detail (Figure 5). With only 30–100 photons detected in the last MINFLUX iteration (Figure S20), a localization precision of 2.6 nm (standard deviation) was achieved for both compounds. Single molecules have been localized several times, yielding a total of over 1000 photons on average.

**Figure 5:**
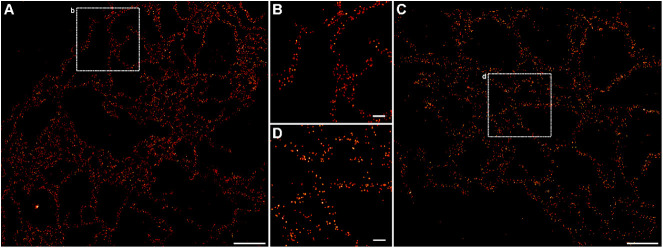
MINFLUX images in fixed U-2 OS cells expressing Halo7-Sec61β. (A-B) 560 nm MINFLUX of a sample labeled with **8a**-Halo; (C-D) 640 nm MINFLUX of a sample labeled with **10**-Me-Halo. Labeling was performed on live cells with 1 μM solutions, and imaging was performed after fixation in aqueous PBS buffer without additives. (B, D) Enlarged ROIs indicated in A and C, respectively. Scale bars: 1 μm (A, C) and 200 nm (B, D).

## Conclusion and Outlook

We prepared photoactivatable 3,7-diamino-N^10^-acetyl phenoxazine dyes as fluorescent markers for use in optical microscopy. The structures contain two NH_2_, NHR, or NR_2_ groups attached to C^3^ and C^7^, as well as the acrylate linker (CH=CH–COOR) attached to the C^1^ of the phenoxazine core. The molecular synthesis was based on metal-catalyzed C–H functionalization of phenoxazine scaffolds, promoted by the powerful *ortho*-directing effect of N^10^-acetyl group. Upon probing catalytic systems, we managed to identify effective conditions where the required asymmetric structures were formed with suitable selectivity, so that tedious HPLC *separation* of regioisomers was not required. This is true for the key compounds **7a** and **10**-Boc-*t*Bu formed from **6**-OtBu and **9** in Scheme 5, respectively, as well as **10**-Me-*t*Bu obtained either from compound **14** or **15** in Scheme 8. In some cases only, HPLC *isolation* of positional isomers was required (a drawback). Taking into account that the syntheses are short (see Schemes 3, 5, and 8), involve common precursors (**6**-R and **13**), and low (mg) amounts of the final products are sufficient for many imaging experiments, this limitation in scale can be tolerated. Before photoactivation, all compounds are essentially nonfluorescent. Irradiation with UV to violet light results in strong emission gain, which is due to cleavage of the acetyl group, capturing it by the neighboring C=C bond (via facile 1,5-shift), followed by oxidation to the conjugated oxazine chromophore. The photoactivation efficiency in aqueous media was demonstrated for the markers bound to a target protein in live and fixed cells. We obtained a set of probes undergoing excitation with commercially available lasers (i.e., 560 and 640 nm), emitting in the orange to far-red spectral region, and suitable for dual-color imaging. The versatility of the “caged” oxazine probes in labeling live cells (using the popular SNAP- and Halo-Tag techniques) or fixed cells (by a standard immunolabeling approach) was demonstrated. Photoactivation and fluorescence imaging were successfully performed in commercial light microscopes: confocal (with diffraction-limited resolution) and MINFLUX (with optical superresolution far beyond the diffraction barrier). As a perspective, the synthesis based on metal-catalyzed acrylate attachment to positions proximal to aromatic NHCOR groups may probably be extended to a variety of other symmetrically or asymmetrically structured dye derivatives with *N*-acyl or *N*-alkyloxycarbonyl groups, e.g., coumarins, rhodamines, and carbo-rhodamines. Importantly, when applied to symmetric dye cores, this approach provides functionalized structures with lower symmetry (higher solubility) and enhanced light absorption. Without using C–H activation and LSF methodologies, these syntheses would require tedious multistep procedures, reflecting the power of catalyzed C–H activation for resource- and step-economical molecular assembly.^15^

**Scheme 8:**
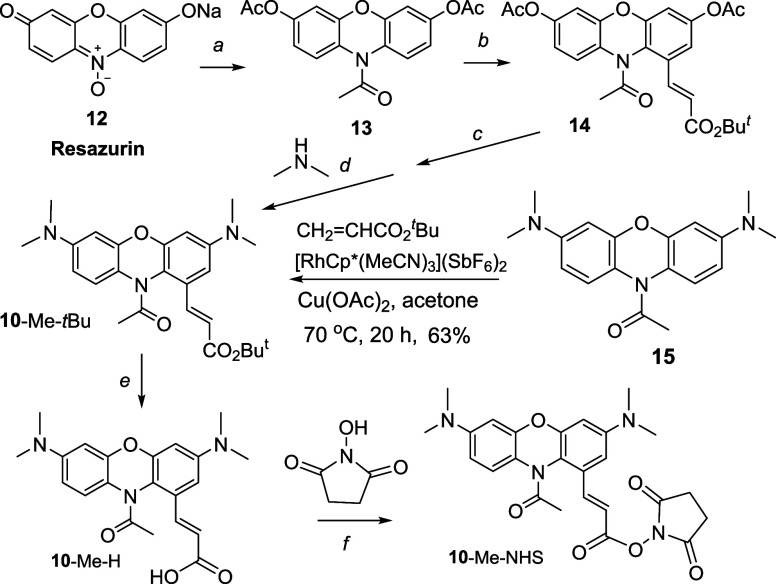
O-N Conversion: An Alternative Synthesis of Functionalized Phenoxazines[Fn sch8-fn8]

a Reagents and conditions: a) H_2_, Pd/C, Ac_2_O, r.t., 20 h, 93% (**13**); b) CH_2_=CHCO_*2*_ *t*Bu, [Ru­(cymene)­Cl_2_]_2_, AgSbF_6_, Cu­(OAc)_2_·H_2_O, acetone, 70 °C, 18 h, 56% (**14**); c) NaOH, EtOH, r.t., 1 h; then Tf_2_O, py, DCM, 0 °C – r.t., 1 h; d) Pd_2_dba_3_, XPhos, Cs_2_CO_3_, dioxane, reflux, 18 h, 59%; e) TFA in DCM, 20% v/v, 0 °C–r.t., 6 h, 70%; f) DCC, DCM, r.t. For experimental details, see Supporting Information.

## Methods

### Synthesis Based on C–H Activation

#### 3,7-Bis-[(*N*,*N*-diethylamino)]­phenoxazines (Scheme 3)

Commercially available dye Oxazine 1 may be used as a model compound for evaluation of late-stage functionalization perspectives. We reduced Oxazine 1 into its nonfluorescent and unstable *leuco* form and then *in situ* acetylated the central nitrogen (N^10^) in order to test whether the acetyl group at N^10^ acts as a directing group in C–H activation reactions. The initial reaction conditions were those optimized for C–H activation of *N*-acetyl rhodamines undergoing olefination with alkyl acrylates.^24^ [Ru­(cymene)­Cl_2_]_2_ was used as catalyst, AgSbF_6_ as the halogen scavenger, and copper (II) acetate as the oxidant, while *tert*-butyl and benzyl acrylates were applied as reagents for oxidative olefination. The reactions **b**, **c,** and **d** in Scheme 3 were carried out in boiling acetone. Initially, oxidative olefination of compound **1**-Me gave low yields (13%) of **2**-*t*Bu and (19%) of **2**-Bn, presumably due to the use of a silver salt as a halogen scavenger, which might oxidize the central nitrogen atom (N^10^) and, thus, the whole chromophore. The use of a halogen-free rhodium catalyst [RhCp*­(MeCN)_3_]­(SbF_6_)_2_ without a silver-containing halogen scavenger improved the yield to 46% for compound **2**-*t*Bu (**c**). Removal of the *tert*-butyl group (**f**) allowed the formation compound **2**-H having a terminal carboxylic acid function (in the acrylate residue connected via C^3^ with C^1^ of oxazine) and a photocleavable *N*-acetyl group. The use of benzyl acrylate as a coupling partner in C–H olefination of **1**-Me was motivated by the possibility to achieve simultaneous deprotection of the carboxyl group and reduction of the double bond in compound **2-**Bn. Thus, we prepared compound **3** having the saturated linker; for the results related to its photolysis, see the previous section and Figure S3.

#### NH-Phenoxazines (Scheme 5)

As the second set of compounds, we studied 3,7-bis-[*N,N’*-(*R*-oxycarbonyl)­amino]­phenoxazines and their oxidative olefination (Scheme 5). Carbamates having NHCO_2_R residues can be *N*-alkylated, allowing the addition of linkers or residues containing polar or functional groups that improve water solubility, cell permeability, or provide the reaction center(s). Importantly, the *N*-[(*tert*-butoxy)­carbonyl]­amino group (NHBoc) has been reported as an *ortho*-DG for oxidative olefination,^25^ which enables, for compound **6**-O*t*Bu, the consideration of the formation of regioisomers with an acrylate attached to C^1^ (acetyl direction), C^2^ (NHBoc direction), and/or C^4^ (NHBoc direction); for numeration of the atoms in the aromatic core, see Schemes 3 and 5. 10-Acetyl-3,7-diaminophenoxazine **5** is an attractive precursor because the amino groups can be transformed into amides or carbamates **6-**R. The synthesis of **5** from commercially available phenoxazine has been reported.^26^ For this, phenoxazine was first nitrated with sodium nitrite in acetone in the presence of acetic acid, yielding a mixture of mono-, di-(mostly), and trinitro phenoxazines. Acetylation of this mixture in boiling acetic anhydride, followed by flash chromatography, afforded dinitro compound **4**. It was then reduced to diamino compound **5,** which was converted to *N,Ń-*bis­(*tert*-butoxycarbonyl) (**6**-O*t*Bu), *N,Ń-*bis­(trifluoroacetyl) (**6**-CF_3_) and *N,Ń-*bis­(benzyloxycarbonyl) (**6-**OBn) derivatives.

We demonstrated that compound **6-**O*t*Bu (Scheme 5) undergoes oxidative olefination. However, with Ru-catalysts,^24^ the results were unsatisfactory. *tert*-Butyl acrylate was chosen as the alkene in order to combine the cleavage of the *tert*-butyl ester and the removal of the N-Boc protecting group in a single procedure, as both steps require acidic conditions.^27^ The use of [Ru­(cymene)­Cl_2_]_2_ (**f**, Scheme 5) as a catalyst led to the products of monosubstitution, **7a** and **7b**. Full conversion was not achieved, even when 3 equiv of acrylate were used. Surprisingly, the degree of polyolefination (the presence of several acrylate moieties in the molecule) and the yields of the desired compounds **7a** and **7b** were low. This may be due to the partial decomposition of the starting material (or products) due to the high temperature of the reaction and/or the use of AgSbF_6_, which can oxidize the chromophore. Carrying out the reaction at lower temperatures (50 °C and r.t.) resulted in very low conversion (<10%). The use of the halogen-free ruthenium catalyst [RuCp*­(MeCN)_3_]­PF_6_ (without silver salt) did not lead to the successful C–H activation of the substrate **6-**O*t*Bu.

It has been reported that, in the presence of [RhCp*­(MeCN)_3_]­(SbF_6_)_2_ as a catalyst and Cu­(II) salt as an oxidant, the NHBoc group directs the incoming acrylate to the *ortho* position.^25^ When we used this catalyst, we obtained only “the most useful” compound **7a** (**g**, Scheme 5) as one of three possible mono-olefinated products. In this case, the directing power of the acetyl, but not the N-Boc group, played the decisive role. The use of another catalyst, [Cp*RhCl_2_]_2_ (**h**, Scheme 5), allowed us to observe the formation of all three monosubstitution products; however, isomers **7b** and **7c** were inseparable. Simultaneous deprotection of *tert*-butyl ester and removal of *tert*-butoxycarbonyl group^27^ (**i**, Scheme 5) led to compounds **8a**–**c** having *N* ^10^-acetyl group and an acrylate linker attached to three different positions of the phenoxazine core. The structures were assigned on the basis of the *J-*values found in the ^1^H NMR spectra of compounds **7a**–**c** and **8a**–**c**. Two protons in the tetra-substituted aromatic rings (indicated as 2 and 4, 1 and 4, as well as 1 and 2 in Scheme 5 for compounds **7a**–**c** and **8a**–**c**) feature characteristic values of *J* ^2,4^ = 1.8–2.5 Hz (*meta*), *J* ^1,4^ ∼ 0–1 Hz (*para*), and *J* ^1,2^ *=* 8–9 Hz (*ortho*). Fortunately, the desired, readily photoactivatable compound **7a** (and the product of its deprotection, **8a**) has been prepared via route **g**, **i** in Scheme 5. Therefore, it was not a disappointment that compounds **8b** and **8c**, with an acrylate linker at the *ortho* position to the amino group, could be isolated only in low yields. For them, we observed the formation of δ-lactams (six-membered rings) resulting from cyclization involving the amino and carboxylic acid groups. For photolysis of compounds **8a**–**c**, see Figures S5 and S6.

Dyes with *N*-alkyl and *N,Ń*-dialkylamino substituents feature red shifts in the absorption and emission bands. The NHBoc groups in compound **6**-O*t*Bu can be deprotonated and *N*-alkylated by methyl iodide. Thus, the *N,N’*-bis-methylated compound **9** in Scheme 5 was obtained, subjected to Rh-catalyzed oxidative olefination with *tert*-butyl acrylate, and gave product **10-**Boc-*t*Bu. It was, in turn, converted to *the N,N’*-dimethyl oxazine derivative **10**-H-H having an acrylate linker and “photo-transferrable” acetyl group.

We also prepared compound **6-**CF_3_ (Scheme 5) in order to clarify whether the NHCOCF_3_ group can serve as a DG in oxidative olefination (no examples in the literature). This may be crucial for compounds having a trifluoroacetyl group as the only possible or preferred protection for the amino group. The [RhCp*­(MeCN)_3_]­(SbF_6_)_2_ complex provided oxidative olefination of **6**-CF_3_ with *tert*-butyl acrylate giving **11**-COCF_3_ as a sole product (Scheme 5). Here, again, the directing power of *N*-acetyl group played the decisive role, but, on the other hand, *N*-trifluoroacetyl group did not preclude olefination at C^1^ in compound **6**-CF_3_.

#### O,Ó,N-Tris-Acetyl-Protected Resorufins

*“O–N” conversion.* *N*-Acetyl-3,7-diaminophenoxazines decorated with one acrylic acid residue attached to the C^1^ of the heterocycle were prepared according to Schemes 3 and 5. Some synthesis steps pertinent to these schemes are low-yielding, lead to difficult separations of positional isomers, and are accompanied with decomposition of the dye core. Another limitation is that this approach leads to PA phenoxazines having primary or secondary amino groups in their structures. Thus, the synthesis of phenoxazines with tertiary amino groups remains challenging. These dyes are favored because the presence of *N,N*-dimethylamino groups induces red shifts in the absorption and emission spectra. To overcome these limitations in the synthesis and structure, we sought an alternative approach to prepare functionalized PA phenoxazines with 3,7-bis­(*N,N′*-dialkylamino) residues and, eventually, other substituents attached to nitrogen atoms.

To access such compounds, we used Resazurin as a commercially available and very affordable starting compound. The whole synthesis is simple and is given in Scheme 8. Reduction of Resazurin, accompanied by acetylation of the amino and hydroxy groups, yielded *O,Ó,N*-triacetylated derivative **13**. *O*-Acetate—a protecting group for the hydroxyl residues of Resorufin—was reported to act as an *ortho*-directing group in Ru-catalyzed oxidative olefination of arenes,^28^ and we expected to obtain positional isomers. Fortunately, under conditions tested before (**f**, Scheme 5), Ru-catalyzed oxidative olefination of triacetate **13** gave compound **14** with the position of an acrylate linker (attached to C^1^) optimal for facile photoactivation. The products of polyolefination and positional isomers were not detected. Thus, in this scaffold, the weakly coordinating directing ability of *N*-acetyl group was found to be superior to that of *O*-acetyl group. Thus, the addition of a C–H activation step into the synthesis route in Scheme 8 enabled the preparation of functionalized phenoxazines from commercially available Resazurin. For that, compound **14** was converted to triflate and subjected to a Pd-catalyzed C–N cross-coupling reaction with dimethylamine,^29^ affording 3,7-diaminophenoxazine **10**-Me-*t*Bu (Scheme 8). For compound **10**-Me-*t*Bu, no Michael addition occurred, though we might expect that in the course of Buchwald–Hartwig amination, methylamine can act as *aza*-Michael donor, and acrylate as a Michael acceptor. The structures of side products formed along with PA phenoxazines, as shown in the synthesis schemes, are given in Supporting Information. Acidic cleavage of *tert*-butyl ester **10**-Me-*t*Bu resulted in carboxylic acid **10**-Me-H suitable for further derivatization. Alternatively, ester **10**-Me-*t*Bu can be prepared from compound **15**, as shown in Scheme 8. However, the route involving diacetate **14** is more versatile because various primary or secondary amines may be used in Pd-catalyzed C–N cross-coupling reactions.

## Supplementary Material



## Notes

Details of photolysis experiments (photoactivation) in a cuvette and an optical microscope , as well as biolabeling and sample preparation procedures, the description of the synthesis with copies of LC-MS traces and NMR spectra of the new compounds (PDF)
