# Supplementary material for: Phenoxazines with a Phototransferable N‑Acetyl Group and Acrylate Linker: Assembly by C–H Activation, Photoconversion to Fluorescent Dyes, Biolabeling, and Super-Resolution Imaging
Source: J Am Chem Soc. 2026 Jun 2;148(23):24345–56. doi: 10.1021/jacs.6c05772 (PMC13281514; doi:10.1021/jacs.6c05772)
Supplement: Supplementary file 1 [file ja6c05772_si_001.pdf]

Supporting Information

Phenoxazines with Photo-transferrable *N*-Acetyl group and Acrylate Linker: Assembly by C-H Activation, Photoconversion to Fluorescent Dyes, Biolabeling and Superresolution Imaging

Elizaveta Savicheva,[a] Jasmine Hubrich,[b] Taukeer A. Khan,[a] Mariano L. Bossi,[b,*] Vladimir N. Belov,[a,*] Lutz Ackermann,[c,d*] and Stefan W. Hell[a,b,*]

[a]Department of Nanobiophotonics, Max Planck Institute for Multidisciplinary Sciences,

Am Fassberg 11, 37077 Göttingen Germany

[b]Department of Optical Nanoscopy, Max Planck Institute for Medical Research, Jahnstrasse 29, 69120 Heidelberg, Germany

[c]Institut für Organische und Biomolekulare Chemie, Georg-August-Universität Göttingen, Tammannstrasse 2, 37077 Göttingen, Germany

[d]German Center for Cardiovascular Research (DZHK), Potsdamer Straße 58, 10875 Berlin, Germany

*Corresponding authors: Mariano.Bossi@mr.mpg.de; vladmir.belov@mpinat.mpg.de; Lutz.Ackermann@chemie.uni-goettingen.de; Stefan.Hell@mpinat.mpg.de

Table of contests

Photolysis, imaging and biolabeling *S3*

Synthetic procedures *S21*

Copies of LC-MS traces and NMR spectra *S49*

Photolysis, imaging and biolabeling

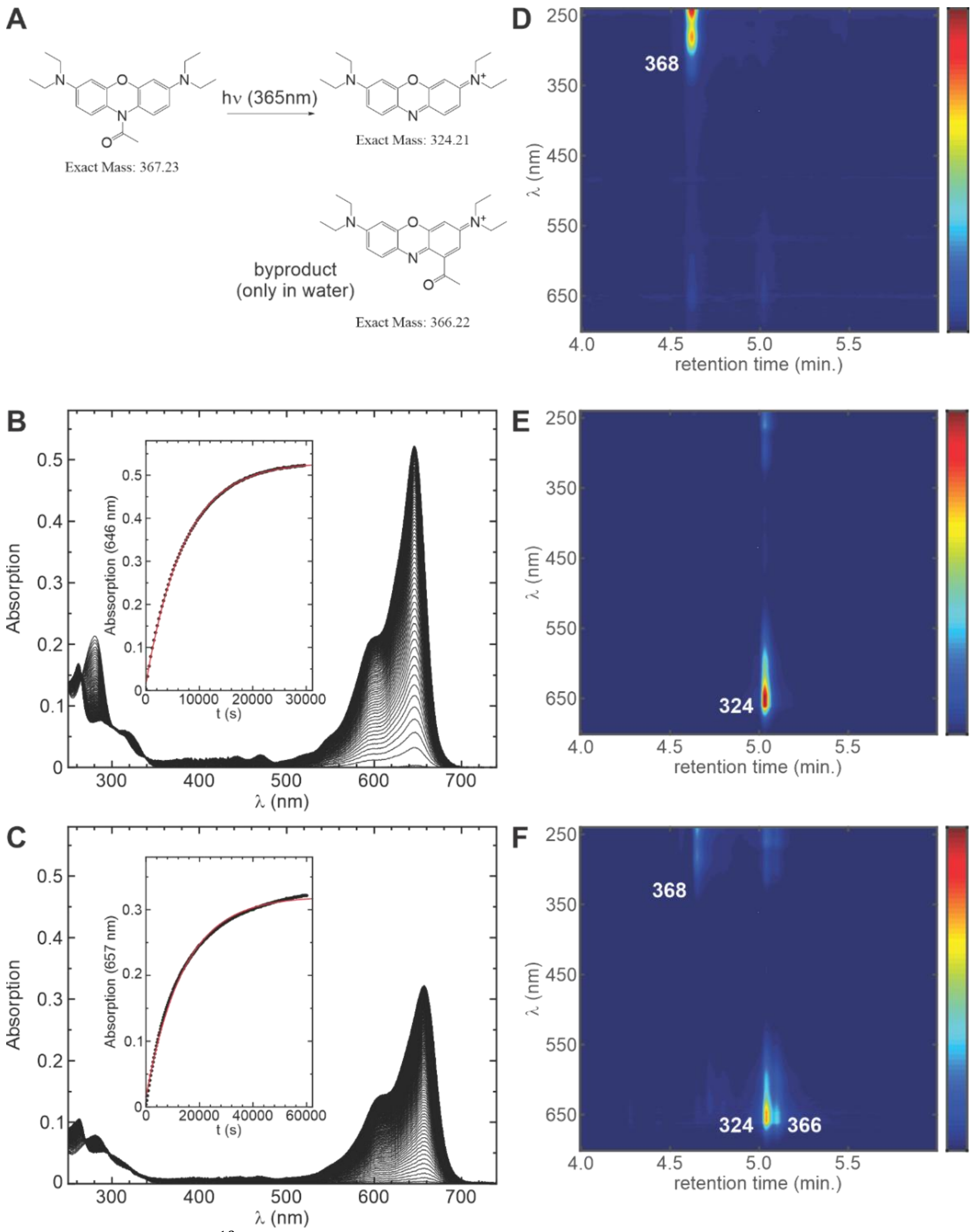

Figure S1. Irradiation of N^{10}-acetyl-*N,N,N',N'*-tetraethylphenoxazine (**1**-Me in Scheme 3 of the main text). Absorption changes during irradiation with 365 nm light in MeCN (B) and aqueous buffer solution containing 10% v/v MeCN (C). The insets show the transients at the absorption maxima of the products. (D-F) LC-MS 2D maps of the starting compound, and the irradiation in MeCN and aqueous solution, respectively. The *m/z* values of the main components (LC-MS peaks) are indicated. Due to the low absorption coefficient of the starting material at the irradiation wavelength (365 nm), the reaction quantum yield was not calculated.

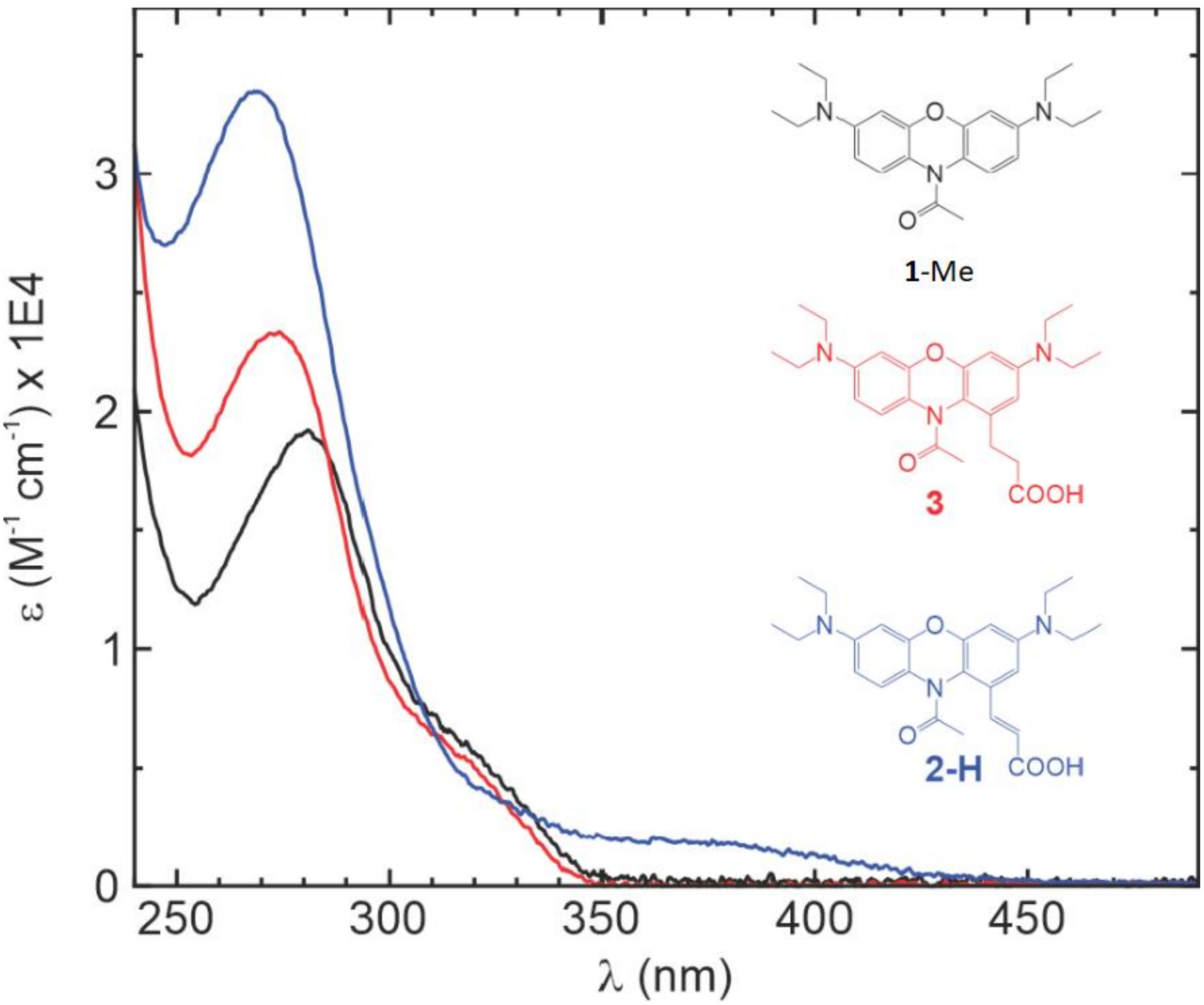

Figure S2. Absorption spectra of compounds **1**-Me, **2**-H and **3** (see Schemes 3 and 4 in the main text) dissolved in aqueous buffer (pH = 7).

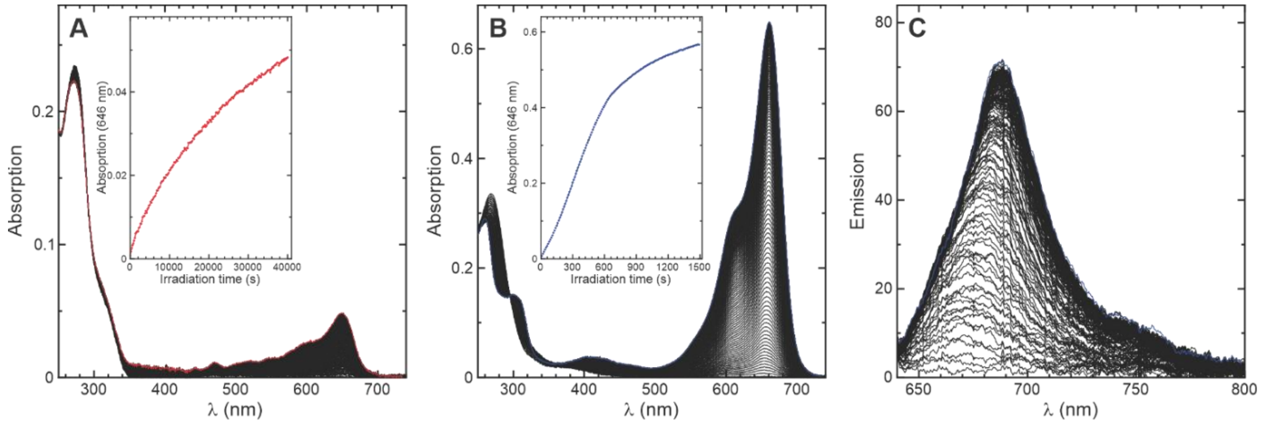

Figure S3. Photolysis of compounds **3** (A) and **2**-H (B-C) in aq. buffer (pH = 7) by irradiation with 365 nm light. The insets show the transients at the absorption maxima in the visible range. For structures, see Schemes 3 and 4 in the main text and Figure S2.

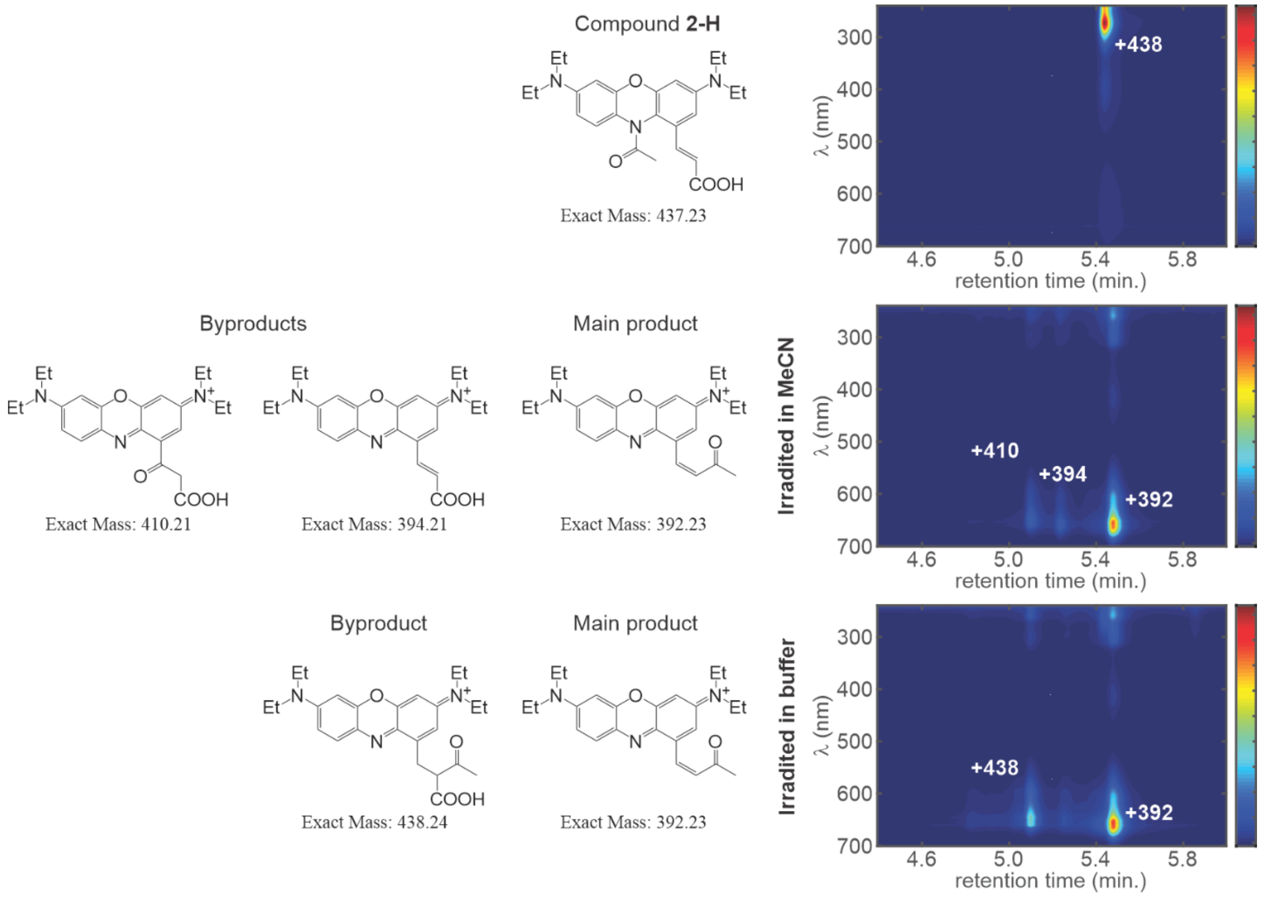

Figure S4. LC-MS analysis of the reaction mixtures obtained after photolysis of compound **2**-H in MeCN and aqueous buffer (pH = 7) with 365 nm light. The molecular masses of the main components are displayed and attributed to the corresponding peaks in 2D LC-MS plots (positive ionization mode).

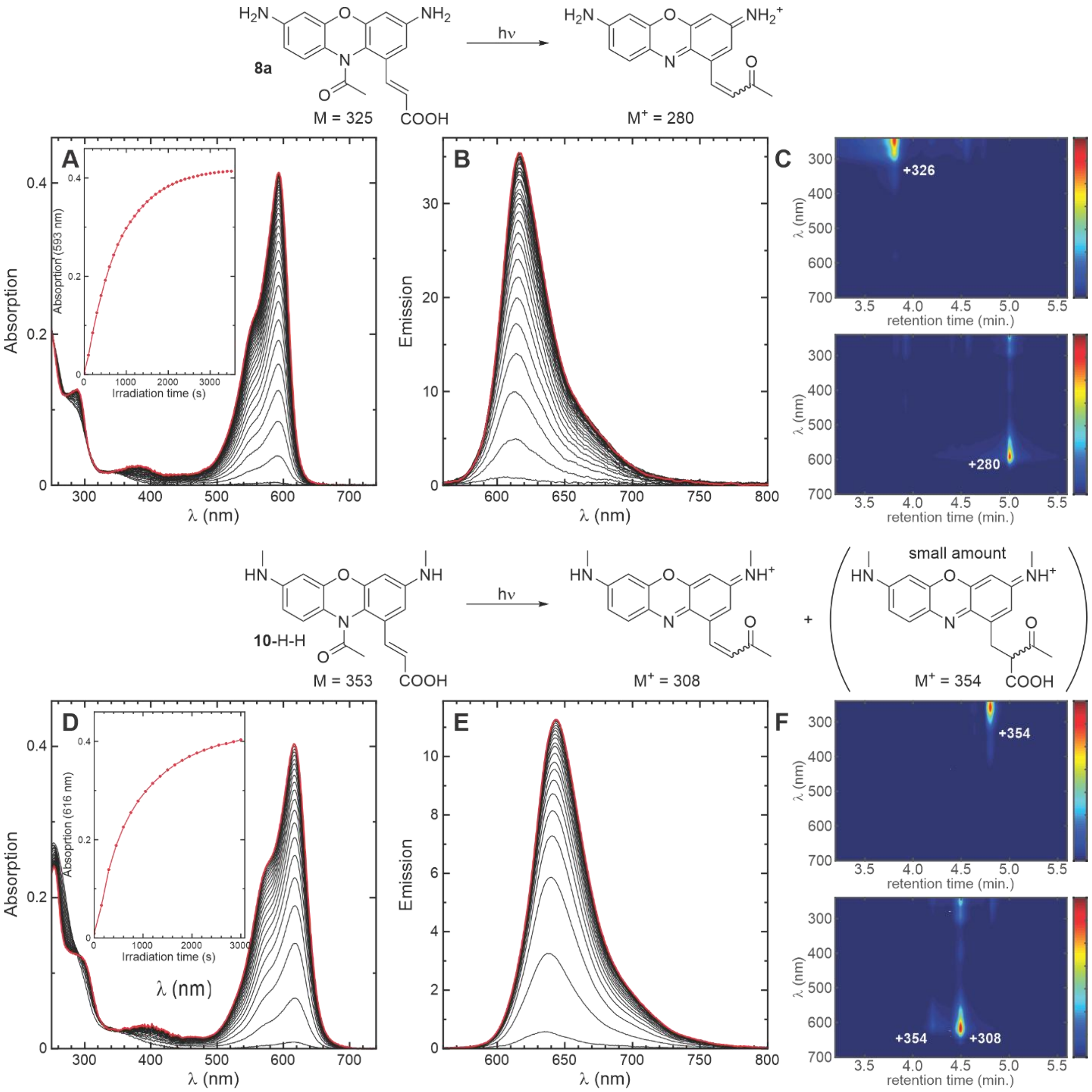

Figure S5. Photolysis of compounds **8a** (A-C) and **10**-H-H (D-F) (see Schemes 5 and 6 in the main text) in aqueous buffer (pH = 7). (A,D) Absorption changes, (B,E) emission changes and the LC-MS traces of the solutions (C, F) before (top) and after (bottom) the photolysis with 365 nm light. The insets in A and D show the transients at the absorption maxima in the visible range. The molecular masses of the main components are displayed and attributed to the corresponding peaks in 2D LC-MS plots (positive ionization mode).

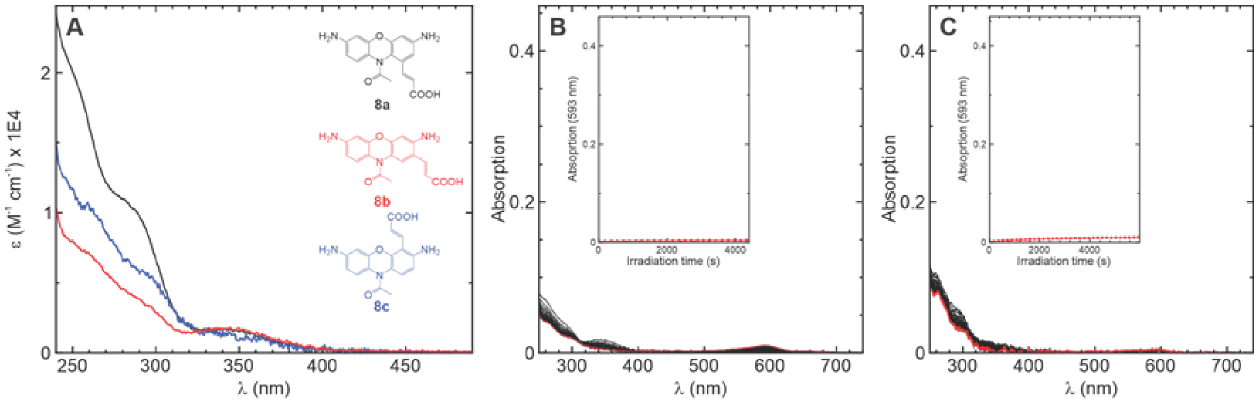

Figure S6. (A) Absorption spectra of compounds **8a-8c** (see Scheme 5 in the main text) in aqueous buffer solution (pH = 7). Photolysis of compounds **8b** (B) and **8c** (C) in aqueous buffer (pH = 7) with 365 nm light. The insets in B and C show the transients at the absorption maxima in the visible range. Compare with the photolysis results of compound **8a** shown in Figure S5.

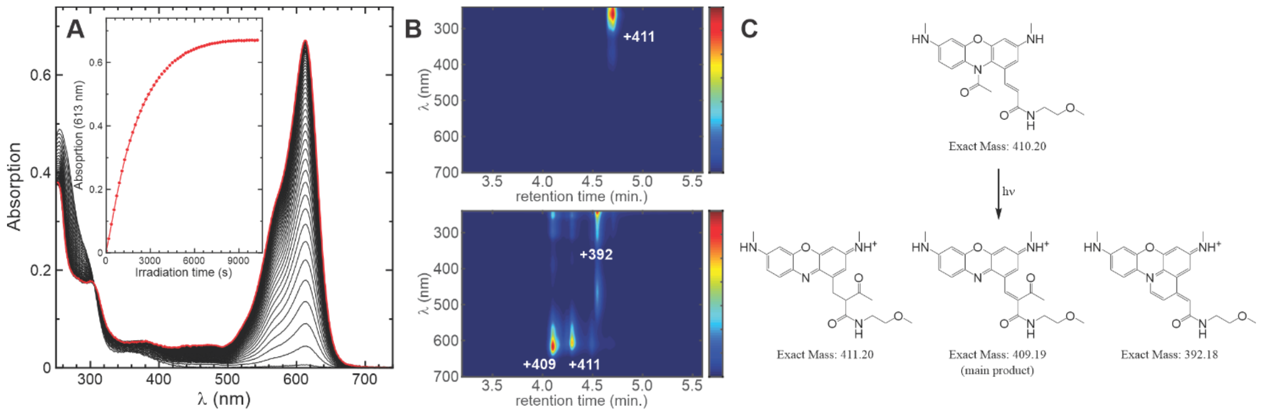

Figure S7. Photolysis of compound **10**-NH(CH_2)$_2$OMe (Scheme 7 in the main text) with 365 nm light. (A) Absorption changes; (B) LC-MS analysis of the solutions before (top) and after (bottom) the photolysis; (C) product distribution. The molecular masses of the main components are displayed and attributed to the corresponding peaks in 2D LC-MS plots (positive ionization mode).

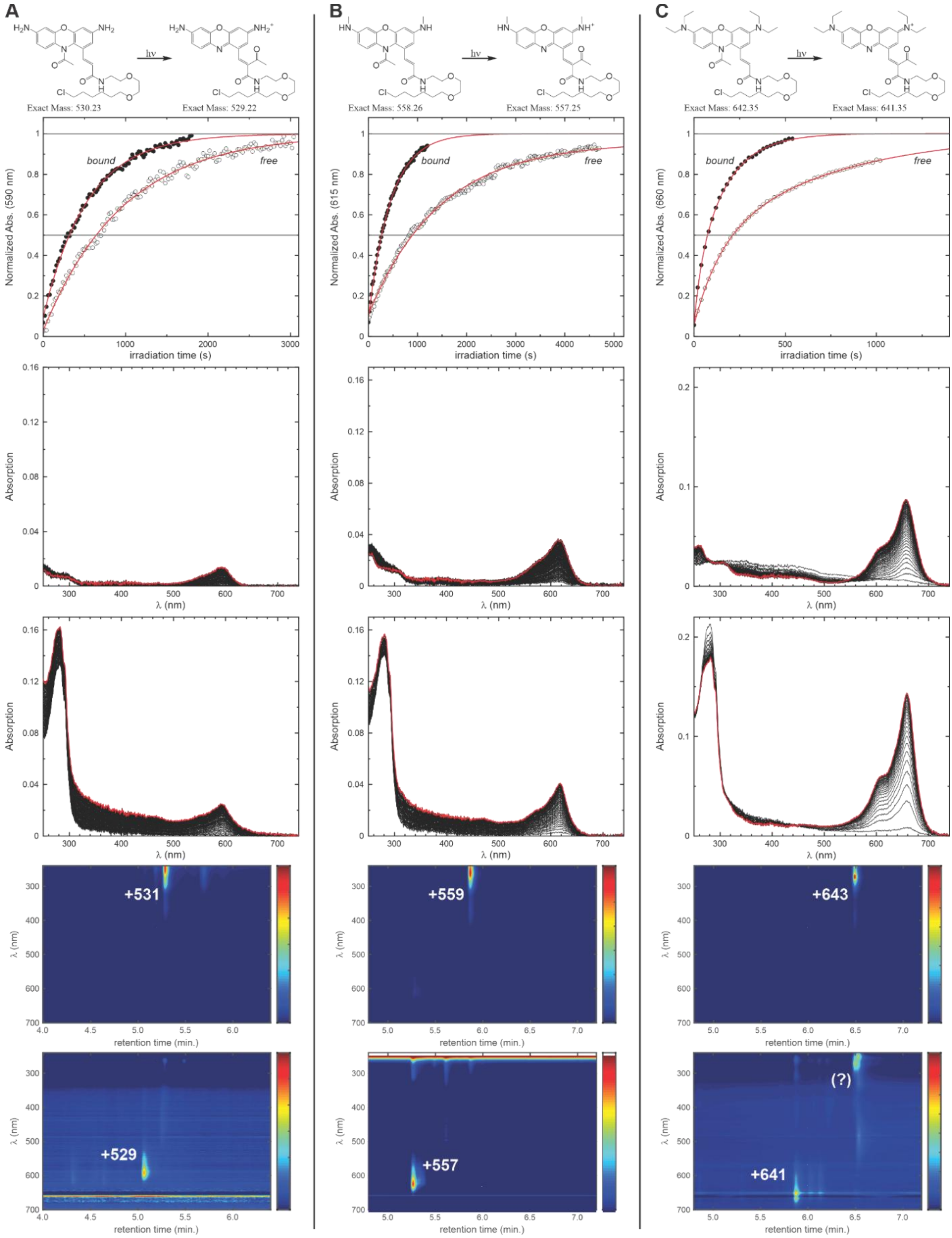

Figure S8. Photoactivation of HaloTag probes (A) **8**-H-Halo = **CA1**, (B) **10**-H-Halo = **CA2**, and (C) **2**-Halo = **CA3** (see Scheme 7 in the main text) in aqueous solutions in a free state and after binding to HaloTag protein. Absorption changes, transients in the visible range and 2D LC-MS plots before and after activation with 365 nm light. The molecular masses of the main components are displayed and attributed to the corresponding peaks in 2D LC-MS plots (positive ionization mode).

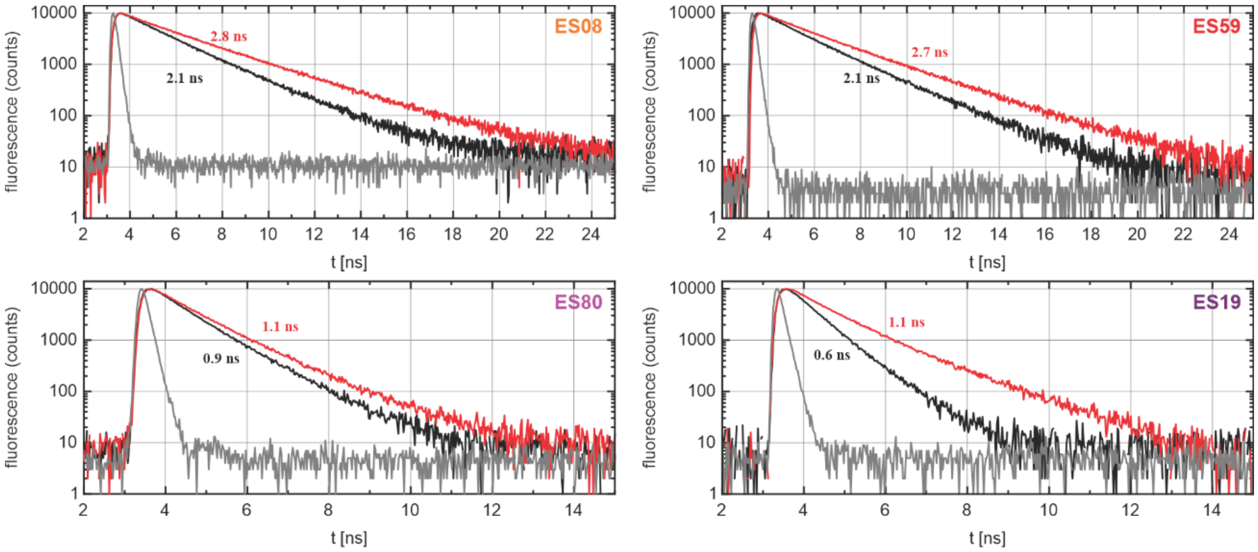

Figure S9. Fluorescence lifetimes of photoactivated oxazines **8a**-Halo (**CA1**), **10**-H-Halo **(CA2), 2**-Halo **(CA3)** and **10**-Me-Halo (**CA4**) in a free state (black) and bound to HT7 protein (red). For structures, see the main text (Scheme 7).

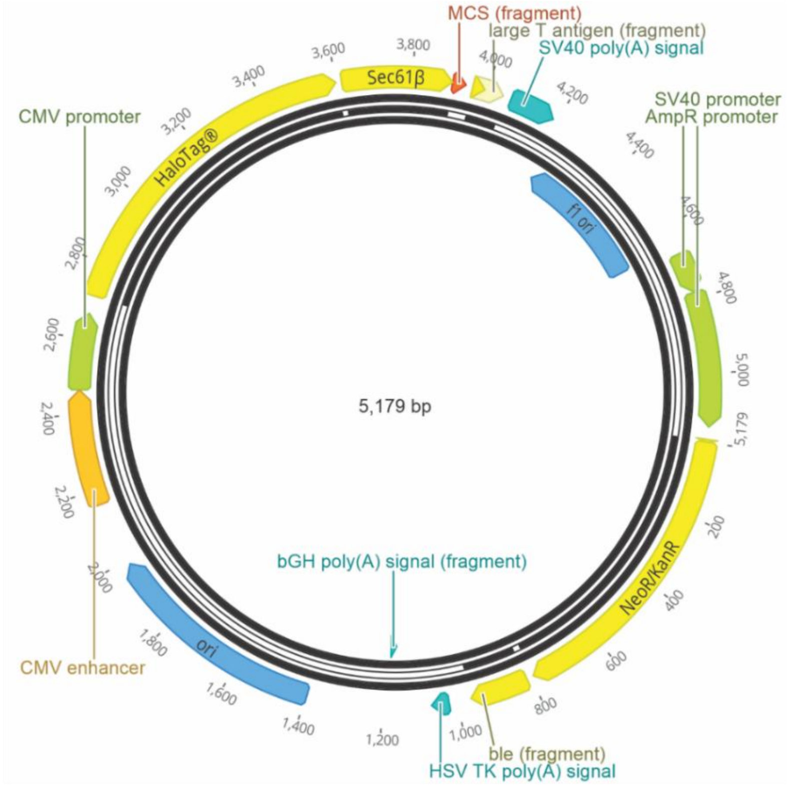

Figure S10. Annotated vector map of the engineered plasmid Halo7-Sec61ß used for expression in mammalian cells. The plasmid is based on pEGFP-C1 backbone and was engineered to express N-terminal Halo7 fused to the target protein Sec61ß (Sec61 translocon subunit beta). Expression is driven by a CMV promoter. This construct contains an origin of replication (ori) and provides resistance against Kanamycin antibiotic for bacterial selection. The map was generated by the Geneious Prime 2025.0.3 software.

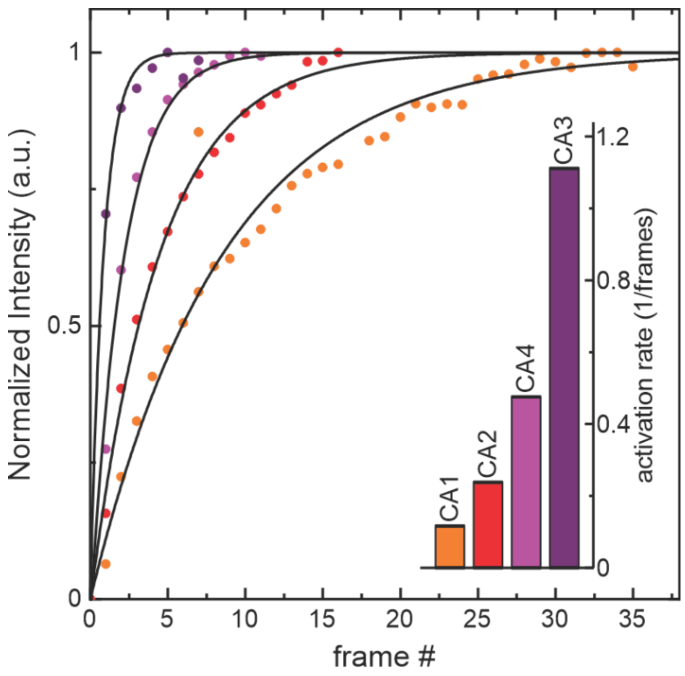

Figure S11. Photoactivation rates observed in a confocal microscope. Samples were labeled and imaged, as shown in Figure 2 (main text), upon activation by sequential irradiation with a 405 nm laser (15 µW, pixel size and dwell time 10 µs and 100 nm, respectively) and image acquisition. Fluorescence images were recordered with excitation wavelengths and detection windows adjusted for each compound (see table S1) to probe the intensity after each activation frame.

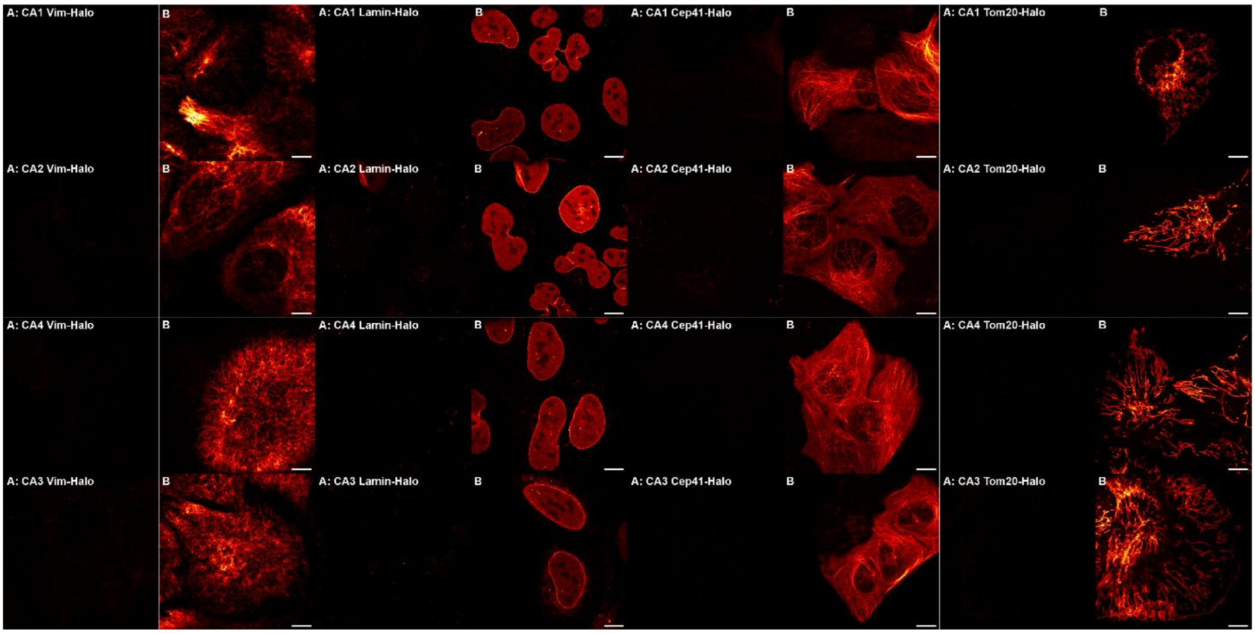

Figure S12. Confocal Images of live cells expressing various HaloTag fusion proteins: Vimentin, Lamin A/C, Cep41 (Tubulin) and Tomm20 (mitochondria) labeled with compounds **8a**-Halo = **CA1**, **10**-H-Halo = **CA2**, **2**-Halo = **CA3** and **10**-Me-Halo = **CA4**. Images shown before (A) and after (B) activation. Scale bars: 10 µm. For structures, see the main text (Scheme 7)**.**

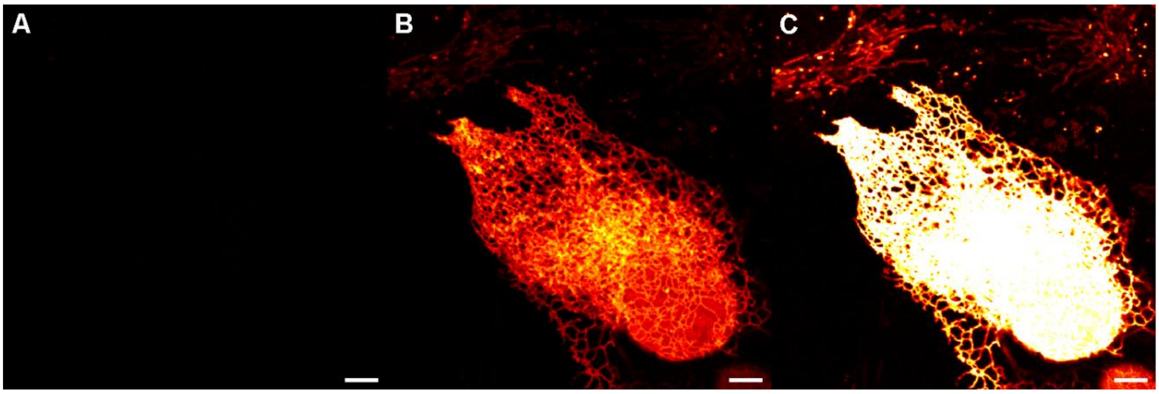

Figure S13. Confocal images of live U2OS cells expressing Halo7-Sec61β, labeled with compound **CA3** = **2**-Halo at a 1 µM concentration. Images were recorded before (A) and after (B-C) photoactivation. Images in A and B are displayed on the same brightness scale, and image C - with a "stretched" brightness scale, to enhanced dim structures (vesicles and mitochondria) in non-transfected cells. Scale bars: 5 µm. For the structure of **2**-Halo, see the main text (Scheme 7).

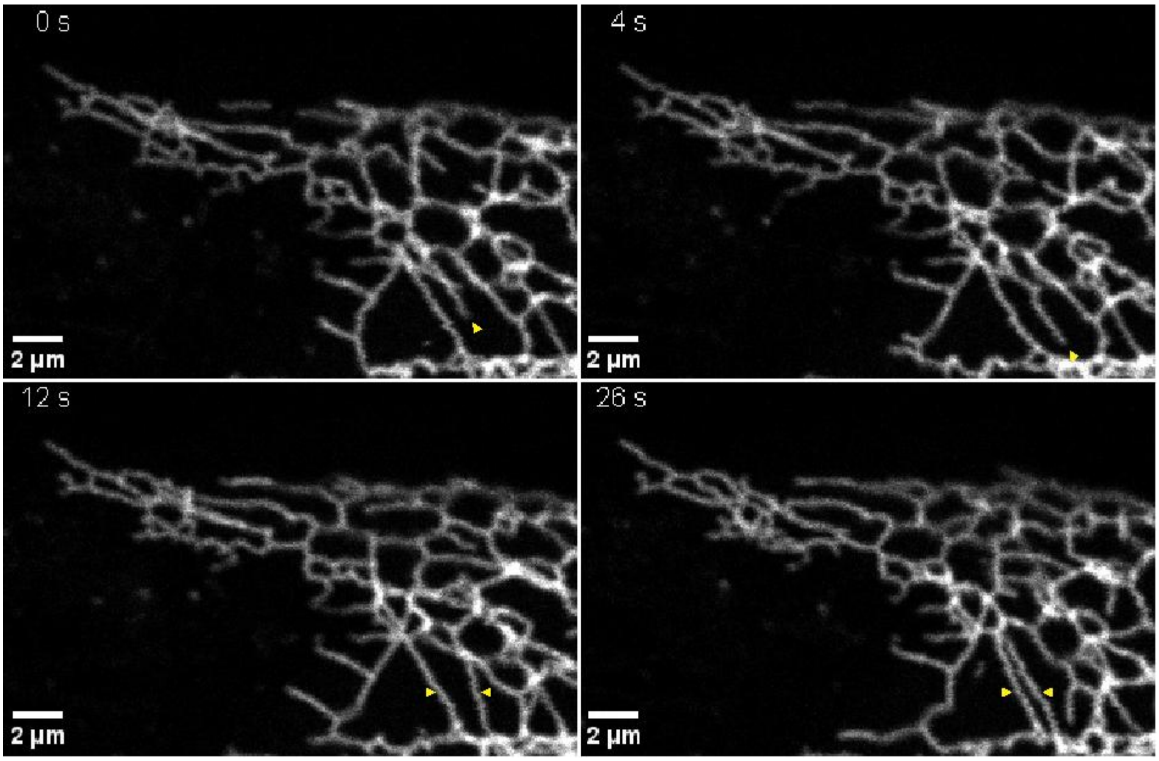

Figure S14. Time-lapse confocal images on live cells. U2OS cells expressing Halo-Sec61β were labeled with compound **10**-Me-Halo = **CA4**, washed and imaged in the supplemented FluoroBrite DMEM medium. Here, the frames at 0 s, 4 s, 12 s and 26 s are displayed, with yellow markings which indicate the growing tubular structure (images 1 and 2) and approaching of neighbouring tubes (images 3 and 4). Scale bar: 2 µm. For the structure of **10**-Me-Halo = **CA4**, see the main text (Scheme 7).

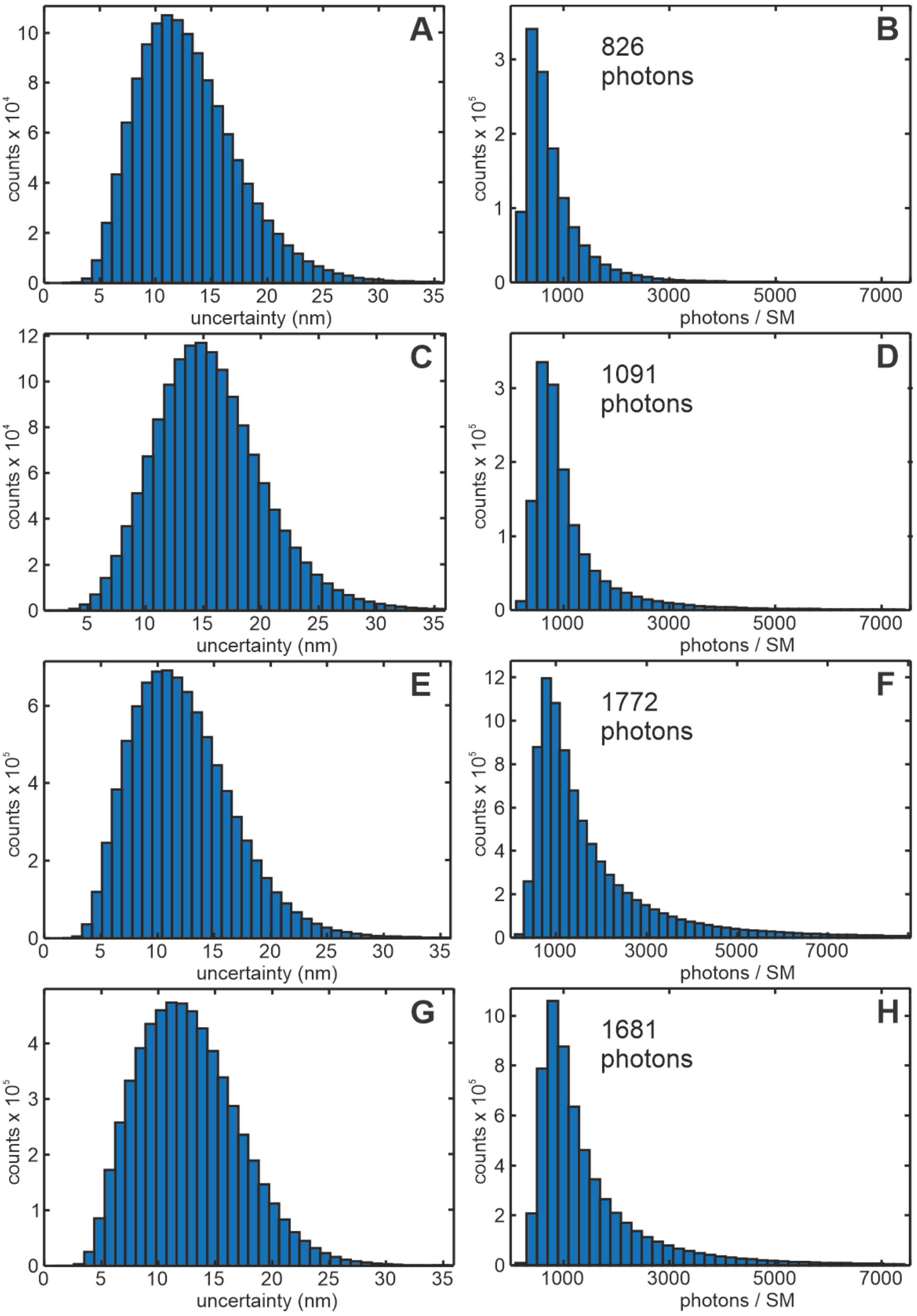

Figure S15. Histograms of the localization uncertainties (A, C, E, G) and the numbers of emitted photon per single molecules (B, D, F, H) obtained for Figure 3 in the main text. The mean numbers of photons per single molecule are given in (B, D, F, H). Images A, B correspond to compound **CA1 = 8a**-Halo, C, D – to compound **CA2 = 10**-H-Halo, E, F – to compound **CA3 = 2**-Halo, and G, H – to compound **CA4 = 10**-Me-Halo. For structures, see the main text (Scheme 7).

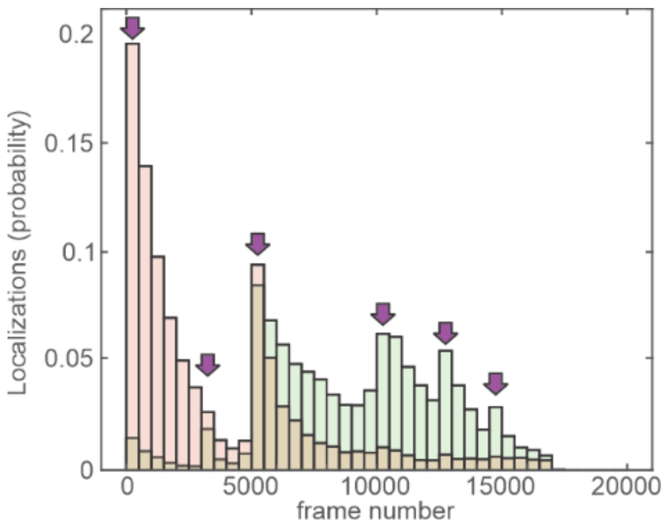

Figure S16. Histograms of localization probabilities *vs*. time (frame number), attributed to detection channel (green: 580 – 620 nm; red: 662 – 710 nm) and obtained during the recording of the images shown in Figures 4E-G (main text). The arrows show the approximate frames numbers, where the power of the activation laser (405 nm) was increased.

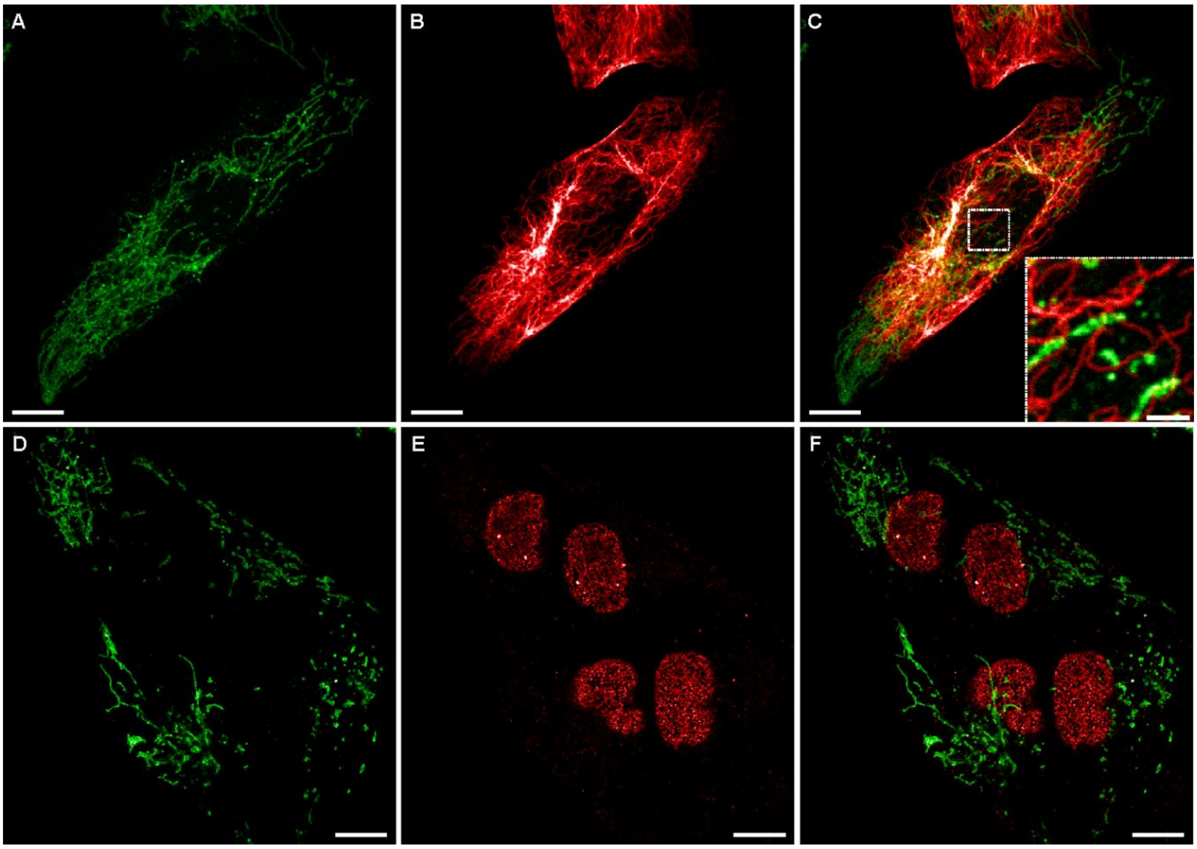

Figure S17. Two-color confocal imaging of fixed cells. Tomm20-Halo cells co-stained with **CA1** = **8a**-Halo (live-cell labeling) and a primary antibody against vimentin (A-C), or a primary antibody against ELYS (Nuclear pores, D-F), in combination with a secondary antibody labeled with **10**-Me-NHS. (A, D) Confocal green channel (**CA1** = **8a**-Halo on Tomm20), (B, E) Confocal red channel (**10**-Me-NHS on vimentin or NUP), and (C, F) composite two-color image. For structures of the probes, see the main text (Schemes 5 and 7). Scale bars: 10 µm (2 µm, inset in C).

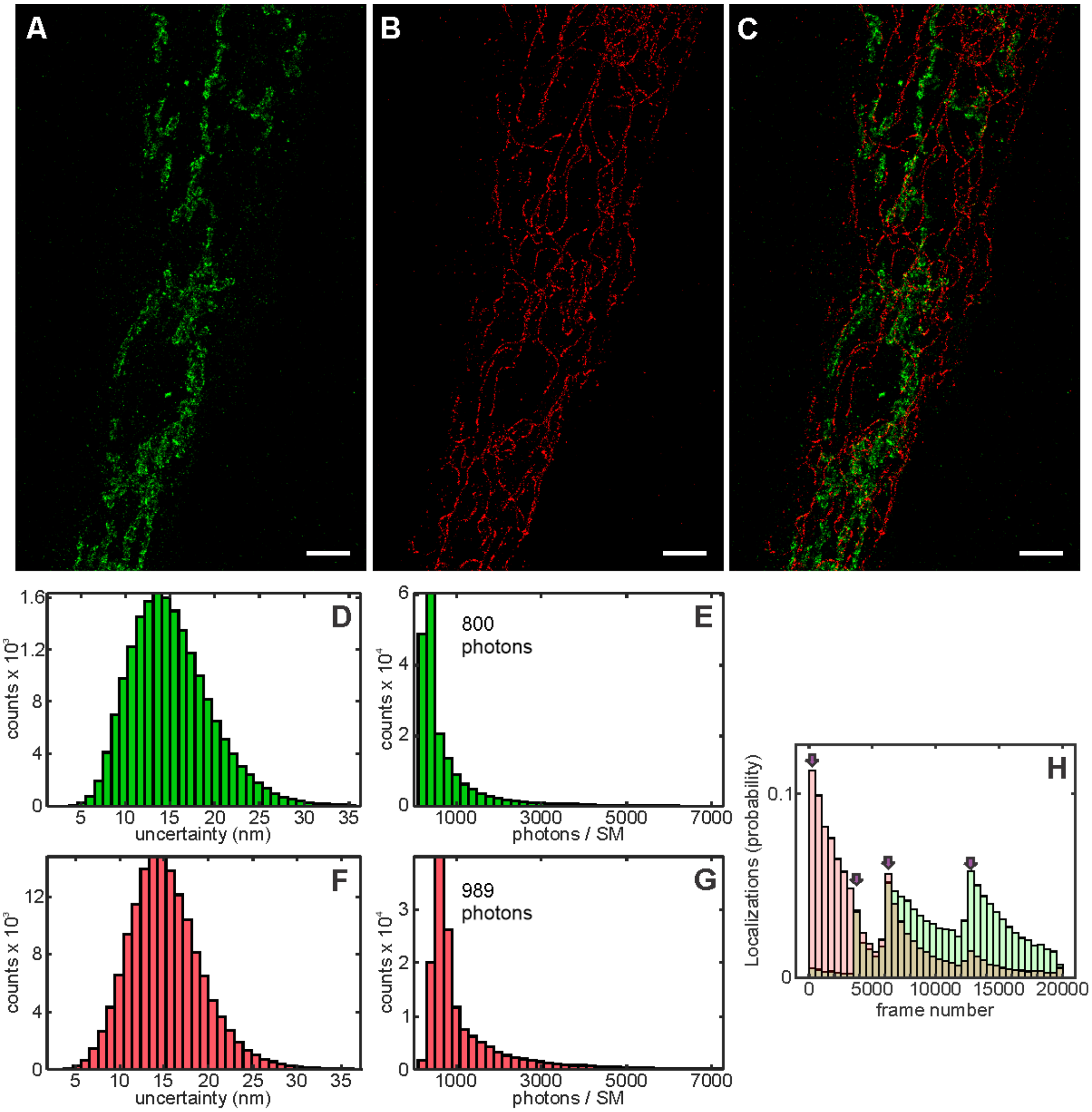

Figure S18. Two-color superresolution PALM imaging of fixed cells. Tomm20-Halo cells were co-stained with **CA1 = 8a**-Halo (live-cell labeling) and a pair of the primary antibody against vimentin and the secondary antibody labeled with **10**-Me-NHS. (A) Green channel (**CA1 = 8a**-Halo on Tom20), (B) red channel (**10**-Me-NHS on vimentin), and (C) composite two-color image. Histograms of localization uncertainties for the green (D) and red channels (F), and histograms of the numbers of emitted photon per single molecules of the green (E) and red channels (G), with the mean number of photons per single molecule indicated. (H) Histograms of the localization over time (frame number), attributed to the channels (green: 580 – 620 nm; red: 662 – 710 nm). The arrows show the approximate frames' positions, where the power of the activation laser (405 nm) was increased. Scale bars: 2 µm. For structures of the probes, see the main text (Schemes 5 and 7).

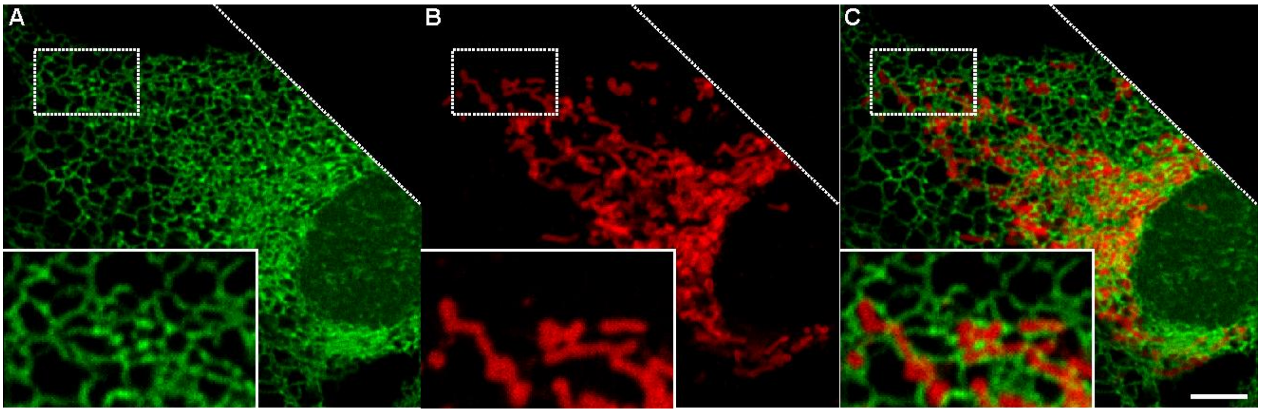

Figure S19. Confocal images of live U-2 OS cells stably expressing Mito-SNAP (OMP25 fused to SNAP-Tag) and ER-Halo (CalR and KDEL fused to Halo-Tag), simultaneously labeled with compounds **CA1** = **8a**-Halo and **10**-Me-NH-PEG-BG (SNAP). Staining was performed with 1 µM solutions, and cells were washed and imaged in the supplemented FluoroBrite DMEM medium. (A) Green channel (560 nm excitation); (B) Red channel (640 nm excitation); (C) Overlay. Images before photoactivation are displayed in the top-right corners, and a zoom of the indicated ROI is presented in the bottom-left corner. Scale bar: 5 µm. For structures of the probes, see the main text (Scheme 7).

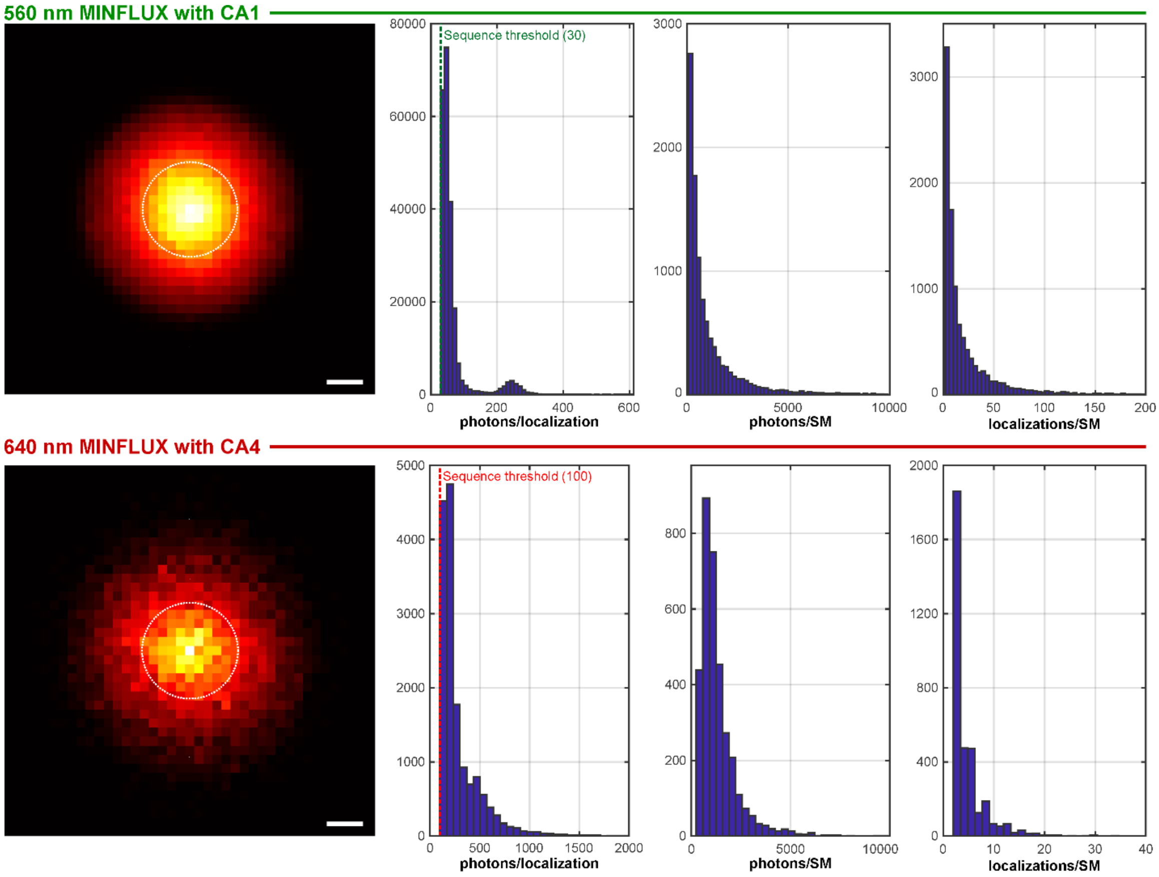

Figure S20. From left to right: localization spreads around the emitter centers (scale bar: 2 nm), histograms of the number of photons (N_{ph}) obtained per localization, N_{ph} obtained from a single molecule (SM), and the number of localizations obtained from a single molecule. The data corresponds to the MINFLUX images (560 nm and 640 nm) displayed in Figure 5 of the main text. The circles indicated in localization spreads correspond to the σ_{XY} value of a 2D-Gaussin fit of the data (2.6 nm for both images).

Photolysis experiments

Solutions of the compound in aqueous phosphate buffer (100 mM, pH = 7.0, 10 μM) were irradiated in the previously described home-built setup[i,ii] with a 365 nm (M365L2, Thorlabs Inc.) and 405 nm LED source (M405L3, Thorlabs Inc.). During the irradiation, samples were maintained at 20 °C and continuously stirred with a Peltier-based temperature-controlled cuvette holder (Luma 40, Quantum Northwest, Inc.). The absorption and emission of irradiated solutions were monitored at the desired irradiation intervals with a fiber-based spectrometer (Flame-S-UV-Vis-ES, Ocean Insight). For absorption measurements, the deuterium and tungsten halogen lamps was used as illumination sources (DH-2000-BAL, Ocean Insight), and fluorescence excitation was performed in a 90° configuration with an LED source (MINTL5 or M625L3, Thorlabs Inc.), in combination with an appropriate bandpass filter (FBH560-10 or FL632.8-1, Thorlabs Inc.). The data collection and analysis were performed with custom-made routines in MatLab. Samples for LC-MS or ESI-MS analyses were taken before and after the photolysis.

Antibody conjugation

Secondary antibodies were coupled with compound **10-**Me-NHS (for structure, see Scheme 5 in the main text) according to standard procedures. In brief, the pH of 420 μL of a 2.4 mg/ml antibody solution (Goat anti-mouse IgG, 115-005-003, or Goat anti-rabbit IgG, 111-005-003, Jackson Immunoresearch), containing ca. 1 mg of antibody, was set to pH 8 by adding 40 μL of 1.0 M aq. $NaHCO_3$; this solution was stirred and immediately mixed with 50 μg of the dye (16 equivalents) dissolved in 5 μl of DMSO. The mixture was stirred in the dark at room temperature for 1 h, and the protein was separated from the unreacted dye via size-exclusion chromatography with a Sephadex G-25 column (PD Minitrap, 28918007, Cytiva), using PBS (pH = 7.4) as the elution buffer (1 ml). The degrees of labeling (DOL) of obtained antibodies were estimated by UV-Vis absorption measurements of the conjugates in a small-volume spectrometer (DS-11+, DeNovix), assuming the absorption of the conjugated dye is identical to the one of the corresponding free carboxylate, and the absorption coefficient of the protein at 280 nm is 210 000 $M^{-1}cm^{-1}$ (for an IgG). The DOL's values were found to be 3.8 and 4.2 dye molecules/protein (anti-mouse and anti-rabbit, respectively).

Cell culture

U-2 OS, U-2 OS-Vimentin-HaloTag,[iii] U-2 OS-HaloTag-Lamin A/C[iv], U-2 OS Flp-In T-REx Tomm20-HaloTag,[v] and U2OS Flp-In T-REx Cep41-HaloTag[vi] cells were cultured for

12-72 h on glass coverslips in Dulbecco's modified Eagle's medium (Gibco, supplemented with 10% fetal bovine serum, 1% GlutaMAX and 1% penicillin/streptomycin). U-2 OS cells stably expressing Mito-SNAP and ER-Halo (Abberior GmbH, article # CELLS-0001-1VIAL) were cultured in McCoy's 5A Medium (supplemented with 10% fetal bovine serum, 1 mM Sodium Pyruvate, and 1% penicillin/streptomycin). Cells were maintained in a humidified incubator at 37 °C with 5% CO_2. They were split, when they reached 80-90% confluency and routinely tested for mycoplasma contamination. The cells were grown on glass coverslips for 48 h before labeling. Tomm20-Halo and Cep41-Halo cells were induced with doxycycline (100 ng/ml) 36–48 hours before labeling.

Plasmid design and preparation

The construct expressing Halo7-Sec61β (see Figure S10 for the plasmid map) was generated by replacing the mEmerald cassette of mEmerald-Sec61β (mEmerald-Sec61b-C1 was a gift from Jennifer Lippincott-Schwartz, Addgene plasmid # 90992 - https://www.addgene.org/90992/)[vii] with corresponding HaloTag7 (gBlocks Gene fragment, ordered from IDT) using flanking AgeI and Kpn2I restriction sites.

Plasmid Transfection

U2OS cells were transfected with 0.5 µg plasmid per well using 1 µL Lipofectamine 2000 (ThermoFisher, cat. # 11668027), according to the manufacturer's recommendations, and labeled with **CA1-4** 24 h after transfection.

Labeling and Sample Preparation

Staining of the HaloTag constructs were performed for 2 h at 1 µM or 250 nM concentration (probe **CA3**) in FluoroBrite DMEM cell culture media (Gibco, supplemented with 10% fetal bovine serum, 1% GlutaMAX and 1% penicillin/streptomycin). Then the cells were washed 2 times for 20-30 min in the same culture media (without the dye) and mounted in fresh medium for live imaging in a magnetic imaging chamber (CM-B18-1, Live Cell Instrument Co.).

For PALM and MINFLUX imaging, cells were rinsed with PBS (pH 7.4), fixed with 3% PFA + 0.1% glutaraldehyde in DPBS (Dulbecco's phosphate-buffered saline, Gibco) at RT/ 20 min, washed twice with freshly prepared 0.1% (wt/wt) $NaBH_4$ solution, and three times with DPBS (to preserve the ER structure), or with 4% formaldehyde solution in PBS at room temperature for 20 min, and washed 3 times (5 min each) with PBS (for other structures). For immunostaining (two-color imaging), samples were permeabilized with 0.1% Triton X-100 in

PBS (5 minutes), blocked with a 2% BSA solution (30 min) and overlaid with the primary antibody anti-ds DNA (Abcam, ab27156), anti-Vimentin (Abcam, ab92547), or anti-AHCTF1 (Sigma Aldrich, HPA031658) in blocking buffer (2% BSA in PBS), and incubated in a humid chamber for 1 h at room temperature. Then, the sample was washed with blocking buffer (3×5 min), and then overlaid with the secondary nanobody labeled with compound **10**-Me-NHS (see Scheme 5 in the main text) in a humid chamber for 1 h at room temperature. The samples were finally washed with PBS (3×5 min), and mounted in aqueous PBS buffer. For MINFLUX imaging, the samples were additionally incubated (5 min) with 150 nm gold beads (BBI Solutions, EM. GC150/7) and rinsed 3 times with PBS.

Optical Imaging. Confocal imaging was performed in an Abberior Expert Line setup (Abberior Instruments GmbH, Göttingen, Germany) built on a motorized inverted microscope IX83 (Olympus, Tokyo, Japan). The microscope was equipped with 405 nm, 561 nm, and 640 nm excitation lasers. Spectral detection was performed with avalanche photodiodes (APD) in spectral windows selected for each particular fluorophore. Images were acquired with a 100x/1.40 UPlanSApo Oil immersion objective lens (Olympus). Pixel size was 70 nm for all images. Laser powers and dwell times were optimized for each sample. Specific imaging parameters are given in Table S1. Image acquisition and image processing was performed with ImSpector software (v. 16.3.13367; Abberior Instruments GmbH, Göttingen, Germany), and all images are displayed as raw data.

Table S1. Confocal imaging parameters.

Figure	Comp.	Photoactivation	Excitation		Detection windows	Pixel size	Dwell time	Line acc.
			Laser	Power				
Fig. 2A[a], S11, S12	CA3, **2**-Halo	405 nm (15 µW)	640 nm	5 µW	650–763 nm	70 nm	20 µs	2
Fig. 2B[a], S11	CA4, **10**-Me-Halo	405 nm (15 µW)	640 nm	5 µW	650–763 nm	70 nm	20 µs	2
Fig. 2C[a], S11	CA2, **10**-H-Halo	405 nm (15 µW)	561 nm	12 µW	576–712 nm	70 nm	20 µs	2
Fig. 2D[a], S11	CA1, **8a**-Halo	405 nm (15 µW)	561 nm	24 µW	576–712 nm	70 nm	20 µs	2
Fig. 4A-D[b], S17	CA1, **8a**-Halo<br>**10**-Me-NHS	405 nm (15 µW)	561 nm<br>640 nm	36 µW<br>10 µW	576-630 nm<br>650-763 nm	70 nm	20 µs	3<br>2
Fig. S19	CA1, **8a**-Halo<br>**10**-Me-NH-PEG-BG	405 nm (15 µW)	561 nm<br>640 nm	36 µW<br>10 µW	576-630 nm<br>650-763 nm	70 nm	10 µs	2<br>2

[a] blue nuclear stain marker image was acquired after activation
[b] channels acquired by line steps

PALM imaging was performed in an ONI Nanoimager V3 (Oxford Nanoimaging, Oxford, UK), equipped with a 561 nm and 640 nm lasers for excitation, and a 405 nm activation laser, and a Hamamatsu Orca Flash4.0 v3 for detection, split in two detection channels with 580 – 620 nm and 662 – 710 nm ranges, and with a wide-field pixel size of 117 nm. Illumination was performed in HILO mode, the temperature in the imaging chamber was set at 20 °C, and the microscope focus was stabilized during measurements. Specific imaging parameters are given in Table S2. Data was analysed and processed using the ONI Nanoimager™ Software (Development build: Apr 9 2023 22:54:56 Version: 1.19.7.20230409223555 - 28f00b5), and post-processed and rendered using custom-build MatLab (version R2007a) routines.

Table S2. PALM imaging parameters.

Figure	Compound	Exposure Time	Excitation		Detection channel	Activation Laser[a]
			Laser	Power		
Fig. 3A	**CA1, 8a-Halo**	20 ms	561 nm	160 mW	580-620 nm	405 nm
Fig. 3B	**CA2, 10-H-Halo**[b]	20 ms	640 nm	270 mW	662-710 nm	405 nm
Fig. 3C	**CA3, 2-Halo**	20 ms	640 nm	270 mW	662-710 nm	405 nm
Fig. 3D	**CA4, 10-Me-Halo**	30 ms	640 nm	270 mW	662-710 nm	405 nm
Fig. 4E-G	**CA1**<br>**10**-Me-NHS	20 ms	561 nm<br>640 nm	160 mW<br>270 mW	580-620 nm<br>662-710 nm	405 nm
Fig. S16	**CA1**<br>**10**-Me-NHS	20 ms	561 nm<br>640 nm	160 mW<br>270 mW	580-620 nm<br>662-710 nm	405 nm

[a] Activation laser power was manually adjusted to keep a sparse distribution of single molecule events.

[b] when imaged, compound **CA2** presented larger signal in the red channel, than in the green one.

MINFLUX imaging was performed in an Abberior Instruments MINFLUX microscope, equipped with 561 and 640 nm excitation lines and a 405 nm activation line, filter-based detection channels with avalanche photo diodes in three different ranges: 580-630 nm, 650-685 nm, and 685-720 nm. A camera-based sample's stabilization system with a 975 nm laser was used, locked on gold beads (BBI Solutions, EM.GC150/7) incorporated into the sample. Imaging of **8a**-Halo, **CA1** probe was performed with the 561 nm MINFLUX line, 405 nm activation, and detection was performed with the signal combined from the 580-630 nm and 650-685 nm detection channels. Excitation power was set to 6% of the maximum power on the first iteration corresponding approximately to 80 µW. The power of the 405 nm activation line

was set to 0% gradually increased manually up to 100% (20 µW) to sustain the frequency of detected events. Imaging of **10**-Me-Halo, **CA4** probe was performed with the 640 nm MINFLUX line, 405 nm activation, while detection was performed with the signal combined from the 650-685 nm and 685-720 nm detection channels. Excitation power was set to 4% of the maximum power on the first iteration corresponding approximately to 120 µW. The power of the 405 nm activation line was set to 0% gradually increased manually up to 100% (20 µW) to sustain the frequency of detected events. Images were acquired using the default 2D imaging sequence, with an L in the last iteration step of 40 nm, and a photon threshold of 30 or 100 photons. A localization precision of 2.6 nm was obtained (see Figure S20). Images were post-processed with a custom-built MatLab routine with a density-based clustering algorithm dbscan (epsilon = 6 nm, minPts = 3) followed by filtering out localizations from the same molecule (TID) farther than 6 nm with respect to the mean position. The resulting image was rendered as normalized Gaussians with a fixed sigma of 3 nm and a pixel size of 1 nm, and with a nonlinear colour map in square roots for better visualization (gamma correction with A=1 and $\gamma = 0.5$).

Synthetic Procedures

Abbreviations

Ac_2O	acetic anhydride
Ar	argon
aq.	aqueous
ca.	approximately
Boc	*tert*-butoxycarbonyl
conc.	concentrated
CV	column volume
eq.	equivalent
em.	emission
d	doublet (NMR)
Da	Dalton (molecular mass unit)
dba	1,3-dibenzylideneacetone
DCM	dichloromethane
DIPEA	*N*,*N*-diisopropylethylamine
DMA	*N*,*N*-dimethylacetamide
DMF	*N*,*N*-dimethylformamide
EtOAc	ethyl acetate
ESI	electro spray ionization
h	hour
M	mol/L (concentration)
m	multiplet
NMR	nuclear magnetic resonance
NHS	*N*-hydroxysuccinimidyl (ester)
ppm	parts per million
prep.	preparative
q	quartet
r.t.	room temperature
R_f	retention factor
t_R	retention time
s	singlet

t	triplet
TFA	trifluoroacetic acid
TFAA	trifluoroacetic anhydride
THF	tetrahydrofuran
TLC	thin layer chromatography
TSTU	*N*,*N*,*N'*,*N'*-Tetramethyl-*O*-(*N*-succinimidyl)uronium tetrafluoroborate
v/v	volume/volume ratio

General experimental information

The reagents and starting materials were purchased from commercial suppliers – Sigma Aldrich (Merck), ABCR, Carbosynth, TCI, Alfa Aesar – and stored according recommendations of the producers and used without purification (unless stated otherwise). Solvents and Pd/C (oxidized form, 10% Pd, catalyst for hydrogenation) were obtained from Merck (Germany). Anhydrous solvents were stored over molecular sieves. Deuterated solvents for NMR spectroscopy (CD_3CN, CD_3OD, D_2O, $(CD_3)_2CO$, $CDCl_3$) were purchased from Deutero GmbH. The reactions were performed with magnetic stirring under argon. The temperature "0°C" corresponds to the cooling of the reaction mixture with an ice bath. Oil baths were used for heating the reaction mixtures, and the bath temperatures are given as reaction temperatures. Evaporations in vacuo were performed in a rotary evaporator with water-bath temperature not exceeding 45°C (unless stated overwise). Hydrogenation reactions were performed with pre-reduced Pd/C (10% Pd) in Schlenk flasks (stopped with septa) connected with a balloon filled with hydrogen and under vigorous stirring (500 – 1200 rpm). Brine is saturated aqueous solution of sodium chloride. Organic solutions of reactions products were dried over anhydrous Na_2SO_4 or $MgSO_4$ prior to evaporation in vacuo.

Preparative chromatography

Flash chromatography (normal phase; regular silica gel) was performed on an automated *Isolera™ One* system with commercially available cartridges (*Biotage GmbH*). For the isolation of photoactivatable compounds and dyes, we used a prep. column Interchim Uptisphere Strategy C18-HQ, 10 µm, 250×21.2 mm (Article No. US10C18HQ-250/212, Interchim), flow rate: 20 mL/min (if not stated otherwise).

Thin-layer chromatography (TLC)

Normal phase TLC was performed on regular *Silica gel 60 F_{254}* (*Merck Millipore*). Reversed-phase TLC was performed on *Silica gel RP-60 F_{254}* (*Merck Millipore*). The spots of compounds were detected by exposing TLC plates to UV-light (254 or 366 nm).

LC-MS analysis

LC-MS analyses were performed on Ultimate 3000 system (Thermo Fisher Scientific) with solvents: 0.1% v/v HCO_2H in MeCN and 0.1% v/v HCO_2H in water. The constant flow rate of 0.5 ml/min and the gradient of MeCN in H_2O (20/80 – 100/0) in 10 min (method A) or 7 min (method B) was applied with columns: *Phenomenex Kinetex C18* column (2.6 μm, 75×3 mm) (method A) or *Phenomenex Kinetex C18* column (1.7 μm, 50×2.1 mm) (method B). Detection: dioden-array detector UV-VIS (>225 nm) and Thermo Fisher Scientific ISQ EM mass-spectrometer.

Spectroscopy

Absorption spectra were recorded with a double-beam UV–vis spectrophotometer (*Varian 4000*) in quartz cuvettes with 1 cm path length. Emission spectra were recorded on a Cary Eclipse fluorescence spectrometer (*Varian*). Fluorescence quantum yields (absolute values) were measured on a *Quantaurus-QY Absolute PL* quantum yield spectrometer C11347 (*Quantaurus QY*). Excited states lifetimes were measured with *Quantaurus-Tau* device with TDC Unit M12977-01 (Hamamatsu).

Nuclear Magnetic Resonance (NMR) spectroscopy

NMR spectra were recorded at 25 °C on an Agilent 400-MR spectrometer at 400 MHz (^{1}H), 376.4 MHz (^{19}F) and 101 MHz (^{13}C). The chemical shifts values are reported in ppm. All ^{1}H spectra are referenced to tetramethylsilane (δ = 0 ppm) using the signals of the residual protons of HDO (4.79 ppm) in D_2O, CHD_2OD (3.31 ppm) in CD_3OD, CHD_2COCD_3 (2.05 ppm) in $(CD_3)_2CO$, CHD_2CN (1.94 ppm) in CD_3CN, DMSO-d_5 (2.50 ppm) in DMSO-d_6, $CHCl_3$ (7.26 ppm) in $CDCl_3$. Multiplicities of signals are described as follows: s = singlet, d = doublet, t = triplet, q = quartet, dd = double of doublets, m = multiplet or overlap of non-equivalent resonances, m_c – centrosymmetric multiplet, dm = clearly resolved doublet of multiplets, br. = broad. *J* values are given in Hz. ^{13}C spectra are referenced to tetramethylsilane (δ = 0 ppm) using the signals of the solvent: CD_3CN (118.7 ppm), CD_3OD (49.00 ppm), $(CD_3)_2CO$ (29.84 ppm), DMSO-d_6 (39.52 ppm), $CDCl_3$ (77.36 ppm).

Mass-Spectrometry (MS)

Low resolution mass spectra (*m/z* = 50 – 3500) with electro-spray ionization (ESI) were obtained on a *Varian 500-MS* spectrometer (Agilent). High resolution mass spectra (HRMS) were obtained on a *Bruker maXis* (ESI-QTOF-HRMS) or *Bruker Autoflex Speed* (MALDI-TOF HRMS) spectrometer (Institut für Organische und Biomolekulare Chemie, Georg-August-Universität Göttingen).

Experimental procedures

Synthesis of new phenoxazine derivatives

1-(3,7-*bis*(Diethylamino)-10*H*-phenoxazin-10-yl)ethan-1-on (1-Me)[viii]

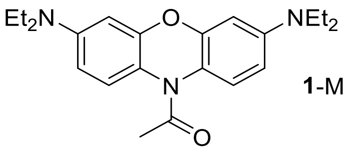

1-Me

$C_{22}H_{29}N_3O_2$
M = 367.23

An oven-dried Schlenk flask was charged with Pd/C (100 mg), Oxazine 1 perchlorate (212 mg, 0.5 mmol) and Ac_2O (15 ml) were added. The mixture was stirred under Ar for 10 min, purged with H_2 and vigorously stirred overnight under H_2 at r.t. The progress of the reaction was monitored by LC-MS (method A; starting material: t_R 4.5 min, λ_{abs} 650 nm, ESI-MS: 324 $[M]^+$; product **1**-Me: t_R 3.8 min, λ_{abs} 279 nm, ESI-MS: 368 $[M+H]^+$). The full conversion was achieved, and no side products detected. The reaction mixture was filtered through Celite, the filter-cake washed with EtOAc, and the solvent removed under reduced pressure to afford 180 mg (98%) of compound **1**-Me as bluish solid. ^{1}H NMR (400 MHz, acetonitrile-d_3) δ 7.24 (d, *J* = 8.9 Hz, 2H), 6.42 (dd, *J* = 8.9, 2.8 Hz, 2H), 6.38 (d, *J* = 2.9 Hz, 2H), 3.33 (q, *J* = 7.1 Hz, 8H), 2.17 (s, 3H), 1.11 (t, *J* = 7.0 Hz, 12H). This spectrum corresponds to ^{1}H-NMR spectrum (500 MHz, $CDCl_3$) of compound **1**-Me reported in the literature.[viii] Compound **1**-Me has been prepared from the dye Basic Blue 3 (the same structure as given in Scheme 3 for Oxazine 1 (see the main text), but Basic Blue contains chloride as an anion) by reduction with sodium dithionite followed by acetylation with acetyl chloride in toluene in the presence of triethylamine.[viii]

***tert*-Butyl (*E*)-3-(10-acetyl-3,7-bis(diethylamino)-10*H*-phenoxazin-1-yl) acrylate (2-*t*Bu)**

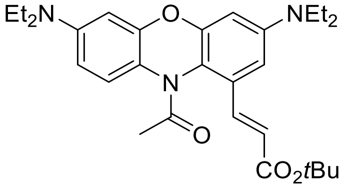

2-*t*Bu

$C_{29}H_{39}N_3O_4$
M = 493.29

A mixture of **1**-Me (92 mg, 0.25 mmol), [$RuCl_2$(*p*-cymene)]$_2$ (15 mg, 0.025 mmol) and $AgSbF_6$ (17 mg, 0.05 mmol) or [RhCp*(MeCN)$_3$](SbF_6)$_2$ (21 mg, 0.025 mmol), Cu(OAc)$_2$ (46 mg, 1 eq., 0.25 mmol), and *tert*-butyl acrylate (1.5 eq., 56 µl, 0.38 mmol) in acetone (2 ml) was stirred at 70 °C for 20 h. The progress of the reaction was monitored by LC-MS (method A; starting material **1**-Me: t_R 3.8 min, λ_{abs} 280 nm, ESI-MS: 368 $[M+H]^+$; product **2**-*t*Bu: t_R 9.5 min, λ_{abs} 271, 389 nm, ESI-MS: 494 $[M+H]^+$. After cooling down to r.t., the reaction mixture was diluted with H_2O (50 ml) and extracted with EtOAc (3×50 ml). The combined organic solutions were washed with brine, dried over anhydrous $MgSO_4$, evaporated under reduced pressure, and the residue submitted to flash chromatography (SNAP Ultra cartridge with 50 g SiO_2, gradient of 10-100% EtOAc in hexane over 6 CV). Compound **2**-*t*Bu was isolated as a greenish solid: 16 mg, 13% yield, with [$RuCl_2$(*p*-cymene)]$_2$ as catalyst; 57 mg, 46% yield, with [RhCp*(MeCN)$_3$](SbF_6)$_2$ as catalyst. ^{1}H NMR (400 MHz, acetonitrile-d_3) δ 7.58 (d, *J* = 16.1 Hz, 1H), 7.26 (d, *J* = 8.8 Hz, 1H), 6.69 (d, *J* = 2.7 Hz, 1H), 6.50 (d, *J* = 2.8 Hz, 1H), 6.46 (dd, *J* = 8.9, 2.8 Hz, 1H), 6.43 (d, *J* = 2.1 Hz, 1H), 6.40 (d, *J* = 11.2 Hz, 1H), 3.36 ("dq", *J* = 12.2, 7.0 Hz, 8H), 2.07 (s, 3H), 1.51 (s, 9H), 1.13 ("td", *J* = 7.0, 4.6 Hz, 12H). ^{13}C NMR (101 MHz, acetonitrile-d_3) δ 172.8, 167.5, 155.1, 154.3, 148.8, 148.3, 141.7, 132.4, 127.4, 121.3, 120.4, 119.6, 107.9, 104.8, 102.6, 100.5, 81.5, 45.59, 45.55, 28.8, 23.0, 13.16, 13.14. The yield was low, and in this experimental part there are 2 methods of synthesis of *N,N,N′,N′*-tetramethyl analog (compound **10**-Me-tBu in Scheme 8 of the main text) which was obtained in better yield.

Benzyl (*E*)-3-(10-acetyl-3,7-bis(diethylamino)-10*H*-phenoxazin-1-yl) acrylate (2-Bn)

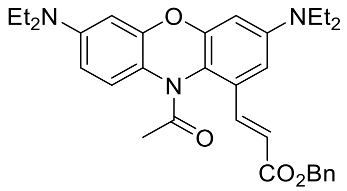

2-Bn

$C_{32}H_{37}N_3O_4$
M = 527.28

A mixture of compound **1**-Me (74 mg, 0.2 mmol), [$RuCl_2$(*p*-cymene)]$_2$ (12 mg, 0.02 mmol), $AgSbF_6$ (14 mg, 0.04 mmol), Cu(OAc)$_2$*H_2O (40 mg, 1 eq., 0.2 mmol), and *tert*-butyl acrylate (1.5 eq., 44 µl, 0.3 mmol) in acetone (2 mL) was stirred at 70 °C for 20 h. The progress of the reaction was monitored by LC-MS (method A, starting material **1**-Me: t_R 3.8 min, λ_{abs} 280 nm, ESI-MS: 368 $[M+H]^+$; product **2**-Bn: t_R 9.8 min, λ_{abs} 272, 398 nm, ESI-MS: 528 $[M+H]^+$. After cooling down to r.t., the reaction mixture was diluted with H_2O (50 ml) and extracted with EtOAc (3×50 ml). The combined organic solutions were washed with brine and dried over anhydrous $MgSO_4$. The solvents were evaporated under reduced pressure, and the

residue was subjected to flash chromatography (SNAP Ultra cartridge with 50 g SiO_2, gradient of 10-100% EtOAc in hexane over 6 CV) to provide ester **2**-Bn as a yellow solid (20 mg, 19% yield). ^1H NMR (400 MHz, Acetonitrile-*d3*) δ 7.71 (d, *J* = 16.1 Hz, 1H), 7.47 – 7.32 (m, 5H), 7.26 (d, *J* = 8.8 Hz, 1H), 6.72 (d, *J* = 2.8 Hz, 1H), 6.56 (d, *J* = 16.0 Hz, 1H), 6.51 (d, *J* = 2.8 Hz, 1H), 6.47 (dd, *J* = 8.8, 2.8 Hz, 1H), 6.42 (d, *J* = 2.8 Hz, 1H), 5.23 (s, 2H), 3.37 (“dq”, *J* = 9.8, 7.0 Hz, 8H), 2.07 (s, 3H), 1.13 (“td”, *J* = 7.0, 0.7 Hz, 12H). ESI-HRMS: found 528.2862 $[M+H]^+$, calculated 528.2857 for $C_{32}H_{37}N_3O_4$ $[M+H]^+$.

(*E*)-3-(10-Acetyl-3,7-bis(diethylamino)-10*H*-phenoxazin-1-yl) acrylic acid (2-H)

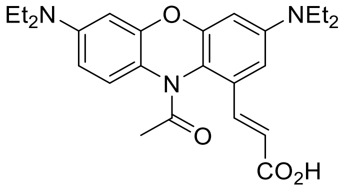

2-H

$C_{25}H_{31}N_3O_4$
M = 437.23

Ester **2**-*t*Bu (13 mg, 26 µmol) was dissolved in DCM (2 ml), the solution cooled to +5°C in an ice bath, and then TFA (200 µl) was added dropwise with stirring. The reaction mixture was allowed to warm-up to r.t. and stirred for 18 h. The progress of the reaction was monitored by LC-MS (Method A, starting compound **2**-*t*Bu, t_R 9.5 min, λ_{abs} 271, 389 nm, ESI-MS: 494 $[M+H]^+$; product **2**-H, t_R 5.1 min, λ_{abs} 272, 394 nm, ESI-MS: 438 $[M+H]^+$; side product **A**: t_R 4.5 min, λ_{abs} 660 nm, ESI-MS 394 $[M]^+$). The volatile materials were removed in vacuo, the residue was co-evaporated with DCM (3×) and kept in vacuo (0.1 mbar) for 2 h. Compound **2**-H was isolated by prep. HPLC on an Interchim puriFlash™ device with a 250 × 21.2 mm column (Knauer Eurosphere II 100-5 C18A, solvent A: H_2O + 0.1% v/v TFA; solvent B: MeCN + 0.1% v/v TFA. Gradient A/B: 80/20–0/100 in 25 min). Upon lyophilization, compound **2**-H (8 mg, 70% yield) was isolated as a bluish powder and the dye **A***2/3 Et_3N (2 mg, 15% yield) - as a dark blue powder. ^1H NMR (400 MHz, acetonitrile-*d3*) δ 7.64 (d, *J* = 16.1 Hz, 1H), 7.28 (d, *J* = 8.8 Hz, 1H), 6.72 (d, *J* = 2.8 Hz, 1H), 6.51 (d, *J* = 2.7 Hz, 1H), 6.49 (d, *J* = 16.1 Hz, 1H), 6.47 (dd, *J* = 8.7, 2.7 Hz, 1H), 6.43 (d, *J* = 2.7 Hz, 1H), 3.37 (dq, *J* = 12.6, 7.1 Hz, 8H), 3.04 (q, “3H”, CH_2N in Et_3N), 2.07 (s, 3H), 1.28 (t, “6H”, CH_3 in Et_3N), 1.13 (td, *J* = 7.0, 4.3 Hz, 12H). ^{13}C NMR (101 MHz, acetonitrile-*d3*) δ 172.9, 168.7, 155.1, 154.3, 148.3, 143.1, 132.3, 127.5, 119.6, 107.9, 105.0, 102.7, 100.5, 45.7 (CH_2N in Et_3N), 45.6, 30.8, 23.0, 13.1, 9.33 (CH_3 in Et_3N). ESI-HRMS: found 438.2388 $[M+H]^+$, calculated 438.2387 for $C_{25}H_{31}N_3O_4$ $[M+H]^+$.

(*E*)-*N*-(1-(2-Carboxyethenyl)-7-(diethylamino)-3*H*-phenoxazin-3-ylidene)-N-ethylethanaminium trifluoroacetate (A)

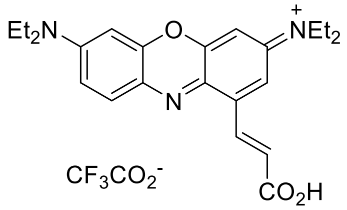

A

$C_{23}H_{28}N_3O_3^+$
$M^+ = 394.21$

^{1}H NMR (400 MHz, acetonitrile-*d*$_3$) δ 8.32 (d, *J* = 16.2 Hz, 1H), 7.79 (d, *J* = 9.7 Hz, 1H), 7.74 (d, *J* = 9.6 Hz, 1H), 7.46 (d, *J* = 2.6 Hz, 1H), 7.29 (ddd, *J* = 12.4, 9.7, 2.7 Hz, 1H), 7.01 (d, *J* = 16.2 Hz, 1H), 6.79 (d, *J* = 2.7 Hz, 2H), 3.72 (“dq”, *J* = 9.0, 6.6 Hz, 8H), 1.30 (m, 12H). ESI-HRMS: found 394.2139 [M]$^+$, calculated 394.2125 for $C_{23}H_{28}N_3O_3$, [M]$^+$. λ_{max} (absorption) 653 nm (ε = 85 000 $M^{-1}cm^{-1}$, EtOH), λ_{max} (emission) 667 nm (EtOH, excitation at 640 nm); fluorescence lifetime 1.4 ns (EtOH), fluorescence quantum yield 0.23 (absolute value in EtOH).

3-(10-Acetyl-3,7-bis(diethylamino)-10*H*-phenoxazin-1-yl)-propanoic acid (3)

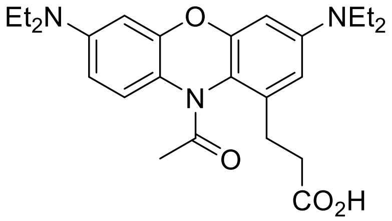

3

$C_{25}H_{33}N_3O_4$
M = 439.25

An oven-dried Schlenk flask was charged with Pd/C (10 mg) in EtOAc (2 ml), and the mixture was stirred under Ar for 10 min, then flushed with hydrogen. A solution of **2**-Bn (20 mg, 38 µmol) in 2 ml of EtOAc was added via syringe, and the reaction mixture vigorously stirred under hydrogen at r.t. overnight. The reaction was complete (method A, monitored by LCMS; starting compound **2**-Bn: t_R 9.8 min, λ_{abs} 272, 398 nm, ESI-MS 528 [M+H]$^+$; product **3**: t_R 3.1 min, λ_{abs} 275 nm, ESI-MS 440 [M+H]$^+$). The reaction mixture was filtered through Celite, and conc. in vacuo to afford 12 mg of compound **3** as a greenish powder (72% yield). ^{1}H NMR (400 MHz, acetonitrile-*d*$_3$) δ 7.23 (d, *J* = 8.8 Hz, 1H), 6.49(dd, *J* = 8.8, 2.8 Hz, 1H), 6.45 (d, *J* = 2.8 Hz, 1H), 6.37 (d, *J* = 2.8 Hz, 1H), 6.33 (d, *J* = 2.8 Hz, 1H), 3.37 (p, *J* = 7.1 Hz, 8H), 2.96 (“dt”, *J* = 14.4, 7.8 Hz, 1H), 2.81 – 2.70 (m, 1H), 2.64 – 2.53 (m, 2H), 2.10 (s, 3H), 1.15 (“td”, *J* = 7.0, 4.6 Hz, 12H).

ESI-HRMS: found 438.2388 [M+H]$^+$, calculated 438.2387 for $C_{25}H_{31}N_3O_4$ [M+H]$^+$.

3,7-Dinitro-(10-acetyl-10*H)*-phenoxazine (4)

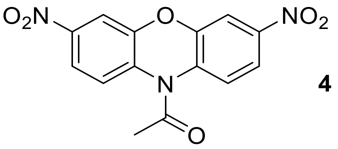

4

$C_{14}H_9N_3O_6$
M = 315.05

1. *Nitration.* To a solution of phenoxazine (1.0 g, 5.5 mmol) and sodium nitrite (760 mg, 11 mmol) in acetone (100 ml), glacial acetic acid (2 ml, 35 mmol) was added. The mixture was stirred at r.t. for 3 days. The reaction progress was monitored by LCMS (method A); starting material: t_R 4.7 min, λ_{abs} 238 nm, ESI-MS 182, $[M-H]^-$; product (dinitro compound): t_R 6.8 min, λ_{abs} 475 nm, ESI-MS 272, $[M-H]^-$; mononitro compound t_R 6.7 min, λ_{abs} 457 nm, ESI-MS 227, $[M-H]^-$; trinitro compound t_R 7.1 min, λ_{abs} 468 nm, ESI-MS 317 $[M-H]^-$. The reaction mixture was poured into 80 m1 of aq. NaOH (0.1 M). The precipitate was filtered off with suction, washed with water and dried in vacuo.

2. *Acetylation.* The residue (1.3 g) was suspended in acetic anhydride (35 ml) and heated under reflux for 3 h. The reaction progress was monitored by LCMS (method A); starting material: t_R 6.8 min, λ_{abs} 475 nm, ESI-MS 272 $[M-H]^-$; product **4**: t_R 6.5 min, λ_{abs} 337 nm, ESI-MS 272 $[M-Ac]^-$. The reaction mixture was conc. in vacuo, and the residue was submitted to flash chromatography on Isolera™ One system (SNAP Ultra cartridge, 50 g SiO_2, hexane/EtOAc with 20-100% EtOAc gradient over 7 CV) to provide compound **4** as a beige solid (875 mg, 50% yield). ^{1}H NMR (400 MHz, acetone-*d*6) δ 8.14 (dd, *J* = 8.9, 2.5 Hz, 2H), 8.03 (d, *J* = 2.6 Hz, 2H), 7.95 (d, *J* = 8.9 Hz, 2H), 2.47 (s, 3H). ^{13}C NMR (101 MHz, acetone-*d*6) δ 169.5, 151.0, 147.1, 135.8, 126.8, 120.5, 113.1, 23.5. ESI-HRMS: found 316.0641 $[M+H]^+$, calculated 316.0642 for $C_{14}H_9N_3O_6$, $[M+H]^+$.

3,7-Diamino-(10-acetyl-10*H*)-phenoxazine (5)

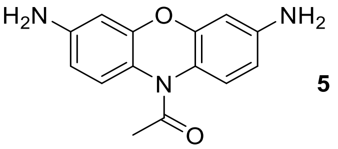

5

$C_{14}H_{13}N_3O_2$
M = 255.10

An oven-dried Schlenk flask was charged with Pd/C (100 mg) in EtOAc (5 ml), and the mixture was stirred under hydrogen for 10 min. Then a solution of **4** (170 mg, 0.54 mmol) in 5 ml of EtOAc was added via syringe, and the reaction mixture vigorously stirred overnight under hydrogen at r.t. The progress of the reaction was monitored by LCMS (column A); gradient MeCN / H_2O +0.1% HCO_2H: 5/95-50/50 in 7 min; starting material **4**: t_R 8.2 min, λ_{abs} 337 nm, ESI-MS 272 $[M-Ac]^-$; product **5**: t_R 2.9 min, λ_{abs} 225, 260 nm, ESI-MS 256, $[M+H]^+$; the full conversion was detected, and no side products found. The reaction mixture was filtered through Celite, and the solvent was removed under reduced pressure to afford 130 mg of bluish solid

(94% yield of compound **5**). ^{1}H NMR (400 MHz, acetonitrile-*d*$_3$) δ 7.16 (d, *J* = 9.2 Hz, 2H), 6.38 (dd, *J* = 6.6, 2.3 Hz, 2H), 6.36 (d, *J* = 2.3 Hz, 1H), 2.15 (s, 3H). ^{13}C NMR (101 MHz, acetonitrile-*d*$_3$) δ 170.8, 153.1, 148.4, 127.0, 121.3, 110.3, 102.8, 23.4. ESI-HRMS: found 256.1080 [M+H]$^+$, calculated 256.1081 for $C_{14}H_{13}N_3O_2$, [M+H$^+$].

Di-*tert*-butyl (10-acetyl-10*H*-phenoxazine-3,7-diyl)dicarbamate (6-O*t*Bu**)**

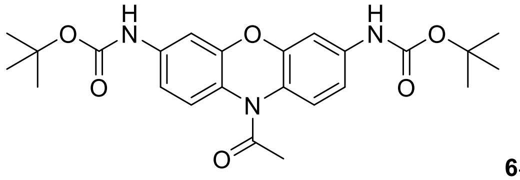

$C_{24}H_{29}N_3O_6$
M = 455.21

6-O*t*Bu

To a solution of compound **5** (130 mg, 0.51 mmol) in ethanol (4 ml) was added Boc_2O (2.3 eq., 1.2 mmol, 272 mg), and the reaction mixture was stirred at r.t. overnight. The formation of compound **6-**O*t*Bu was observed by TLC (hexane/EtOAc, 1:1, R_f = 0.3); starting compound **5**, R$_f$ = 0; and confirmed by LCMS (method A); starting material **5**: t_R 1.1 min, λ_{abs} 225, 260 nm, ESI-MS 256, [M+H]$^+$; **6-**O*t*Bu: t_R 7.8 min, λ_{abs} 256, 286 nm, ESI-MS 456, [M+H]$^+$. The reaction mixture was conc. in vacuo, and the residue submitted to flash chromatography (SNAP Ultra cartridge with 25 g SiO_2, hexane/EtOAc with 10-100% EtOAc gradient over 5 CV) to provide compound **6-**OBu (200 mg, 86 % yield) as a yellowish powder. ^{1}H NMR (400 MHz, Acetone-*d*$_6$) δ 8.58 (s, 2H), 7.54 (d, *J* = 2.4 Hz, 2H), 7.48 (d, *J* = 8.8 Hz, 2H), 7.26 (dd, *J* = 8.8, 2.4 Hz, 2H), 2.27 (s, 3H), 1.49 (s, 18H). ^{13}C NMR (101 MHz, Acetone-*d*$_6$) δ 169.6, 153.7, 151.9, 139.5, 126.2, 125.0, 113.7, 107.1, 80.5, 30.6, 28.6, 23.0. ESI-HRMS: found 456.2121 [M+H]$^+$, calculated 456.2129 for $C_{24}H_{29}N_3O_6$, [M+H$^+$].

***N,N'*-(10-Acetyl-10*H*-phenoxazine-3,7-diyl)bis(2,2,2-trifluoroacetamide) (6-**CF_3**)**

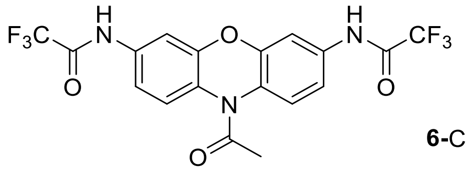

6-CF_3

$C_{18}H_{11}F_6N_3O_4$
M = 447.07

To a solution of **5** (80 mg, 0.31 mmol) in DCM (5 ml) were added at 0 ºC TFAA (3 eq., 1 mmol, 160 µl) and Et_3N (2 mmol, 140 µl), and the reaction mixture was stirred for 1 h. The formation of compound **6**-CF_3 was observed by LCMS (method B); starting material **5**: t_R 1.1 min, λ_{abs} 225, 260 nm, ESI-MS 256, [M+H]$^+$; product **6**-CF_3: t_R 3.1 min, λ_{abs} 246, 293 nm, ESI-MS 448, [M+H]$^+$. The reaction mixture was conc. in vacuo, and the crude residue submitted to flash chromatography (SNAP Ultra cartridge with 25 g SiO_2, hexane/EtOAc with 10-100% EtOAc gradient over 5 CV) to provide compound **6-**CF_3 (97 mg, 70 % yield) as a colorless

powder. ^{1}H NMR (400 MHz, acetone-d_6) δ 10.5 (br. s, 2H, NH), 7.69 (d, J = 2.4 Hz, 2H), 7.63 (d, J = 8.7 Hz, 2H), 7.49 (dd, J = 8.8, 2.4 Hz, 2H), 2.32 (s, 3H).

Dibenzyl (10-acetyl-10*H*-phenoxazine-3,7-diyl)dicarbamate (6-OBn**)**

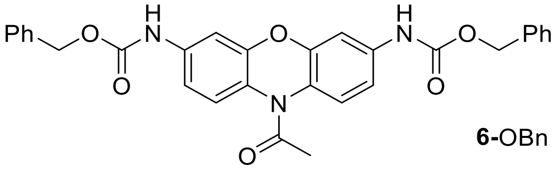

6-OBn

$C_{30}H_{25}N_3O_6$
M = 523.17

To a solution of compound **5** (200 mg, 0.80 mmol) in DCM (4.0 ml) were added pyridine (2 mmol, 161 µl) and $C_6H_5CH_2OCOCl$ (2.0 mmol, 341 mg) at 0 ºC, and the reaction mixture was stirred for 6 h at r. t. The formation of compound **6-**OBn was monitored by TLC (hexane/EtOAc, 1:1), R_f = 0.3; starting compound **5**, R_f = 0. LC-MS (method A), product **6-**OBn: t_R 8.0 min, λ_{abs} 269 nm, ESI-MS 524 [M+H]$^+$. The reaction mixture was conc. in vacuo, and the crude residue submitted to flash chromatography (SNAP Ultra cartridge with 25 g SiO_2, 10-100% gradient of EtOAc in hexane over 5 CV) to provide compound **6-**OBn (150 mg, 36% yield) as a yellowish powder. ^{1}H NMR (400 MHz, acetone-d_6) δ 8.96 (br. s, 2H, NH), 7.56 (d, J = 2.4 Hz, 2H), 7.51 (d, J = 8.7 Hz, 2H), 7.48 – 7.32 (m, 10H), 7.28 (dd, J = 8.7, 2.4 Hz, 2H), 5.19 (s, 4H), 2.28 (s, 3H). ESI-HRMS: found 546.1636 [M+Na]$^+$, calculated 546.1640 for $C_{30}H_{25}N_3O_6$, [M+Na$^+$].

***tert*-Butyl (*E*)-3-(10-acetyl-3,7-bis((tert-butoxycarbonyl)amino)-10*H*-phenoxazin-1-yl) acrylate (7a)**

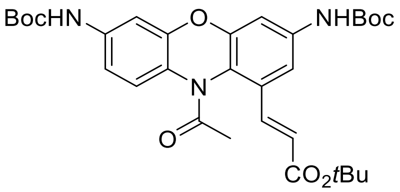

7a

$C_{31}H_{39}N_3O_8$
M = 581.27

Compound **6-**O*t*Bu (340 mg, 0.75 mmol), *tert*-butyl acrylate (2 eq, 220 µl, 1.5 mmol), $[RuCl_2(p\text{-cymene})]_2$ (46 mg, 75 µmol), $AgSbF_6$ (52 mg, 0.15 mmol) and $Cu(OAc)_2*H_2O$ (150 mg, 0.75 mmol) were suspended in 10 ml acetone in a sealed tube. The reaction mixture was stirred at 70°C under Ar overnight. The progress of the reaction was monitored by TLC (hexane-EtOAc 1:1, product R_f 0.5; starting material R_f 0.3) and LCMS (method A); educt **6-**O*t*Bu t_R 7.8 min, λ_{abs} 256, 286 nm, ESI-MS 456 [M+H]$^+$; product **7a**, t_R 9.5 min, λ_{abs} 284, 340 nm, ESI-MS 582 [M+H]$^+$; product **7b** t_R 9.2 min, λ_{abs} 261, 331 nm, ESI-MS 582 [M+H]$^+$. The mixture was diluted with water (100 ml) and extracted with EtOAc (3×100 ml). The combined organic solutions were washed with brine and dried over anhydrous $MgSO_4$. After filtration and

evaporation of the solvents under reduced pressure, the product was isolated by column chromatography (SNAP Ultra cartridge with 50 g SiO_2, elution with 10-100% EtOAc gradient in hexane over 5 CV). Isomers **7a** and **7b** were separated by prep. HPLC on Interchim puriFlash™ with a 250 × 21.2 mm column (Knauer Eurosphere II 100-5 C18A, solvent A: H_2O + 0.1% v/v TFA; solvent B: MeCN + 0.1% v/v TFA. Gradient A/B: 80/20–0/100 in 25 min). Compound **7a**, 148 mg, 34% yield; Compound **7b**, 87 mg, 20% yield. ^{1}H NMR (400 MHz, acetone-d_6) δ 8.66 (s, 1H, NH), 8.61 (s, 1H, NH), 7.77 (d, *J* = 2.3 Hz, 1H), 7.64 (d, *J* = 16.0 Hz, 1H), δ 7.62 (d, *J* = 2.2 Hz, 1H), 7.58 (d, *J* = 2.3 Hz, 1H), 7.57 (d, *J* = 8.7 Hz, 1H), 7.34 (dd, *J* = 8.7, 2.4 Hz, 1H), 6.38 (d, *J* = 16.0 Hz, 1H), 2.17 (s, 3H), 1.53 (s, 9H), 1.51 (s, 9H), 1.48 (s, 9H). ESI-HRMS: found 582.2798 $[M+H]^+$, calculated 582.2810 for $C_{31}H_{39}N_3O_8$, $[M+H^+]$.

***tert*-Butyl *(E)*-3-(10-acetyl-3,7-bis((*tert*-butoxycarbonyl)amino)-10*H*-phenoxazin-2-yl) acrylate (7b)**

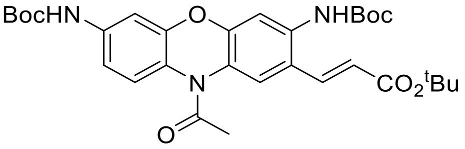

7b

$C_{31}H_{39}N_3O_8$
M = 581.27

^{1}H NMR (400 MHz, acetone-d_6) δ 8.59 (s, 1H, NH), 8.41 (s, 1H, NH), 7.92 (s, 1H), 7.75 (d, *J* = 15.7, 1H), 7.54 – 7.48 (m, 1H), 7.46 (d, *J* = 8.8 Hz, 1H), 7.27 (dd, *J* = 8.8, 2.4 Hz, 1H), 6.32 (d, *J* = 15.7 Hz, 1H), 2.30 (s, 3H), 1.48 (s, 9H), 1.46 (s, 9H), 1.44 (s, 9H). ESI-HRMS: found 582.2803 $[M+H]^+$, calculated 582.2810 for $C_{31}H_{40}N_3O_8$, $[M+H^+]$.

(*E*)-3-(10-Acetyl-3,7-diamino-10*H*-phenoxazin-1-yl) acrylic acid (8a)

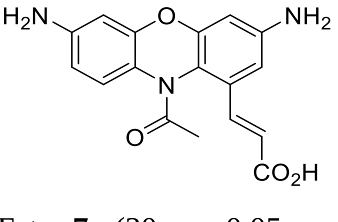

8a

$C_{17}H_{15}N_3O_4$
M = 325.11

Ester **7a** (30 mg, 0.05 mmol) was placed into an amber-colored glass flask, 2.5 ml of DCM added under Ar, and the solution cooled to +5°C in an ice bath. TFA (500 µl) was added dropwise with stirring. The reaction mixture was allowed to warm-up to r.t. and stirred for 18 h. The reaction progress was monitored by LCMS (method A); with starting compound **7a** t_R 9.5 min, λ_{abs} 284, 340 nm, ESI-MS 582 $[M+H]^+$; product **8a** t_R 3.1 min, λ_{abs} 284, 348 nm, ESI-MS 326 $[M+H]^+$; side product **S1** t_R 4.9 min, λ_{abs} 592 nm, ESI-MS 282 $[M+H]^+$. The volatile materials were removed in vacuo, the residue co-evaporated with DCM (3×) and kept in vacuo (0.1 mbar) for 2 h. The title compound was isolated by prep. HPLC on Interchim puriFlash™

device with a 250 × 21.2 mm column (Knauer Eurosphere II 100-5 C18A, solvent A: H_2O + 0.1% v/v TFA; solvent B: MeCN + 0.1% v/v TFA. Gradient A/B: 95/5–0/100 in 25 min. Compound **8a** (10 mg, 61% yield) was isolated as a violet powder; dye **S1** was separated and isolated (5 mg, 35% yield). ^{1}H NMR (400 MHz, Acetonitrile-d_3) δ 7.58 (d, J = 16.1 Hz, 1H), 7.20 (d, J = 8.8 Hz, 1H), 6.79 (d, J = 2.5 Hz, 1H), 6.50 (d, J = 2.5 Hz, 1H), 6.44 (dd, J = 8.7 and 2.5 Hz, 1H), 6.43 (s, 1H), 6.35 (d, J = 16.1 Hz, 1H), 2.05 (s, 3H). ESI-HRMS: found 326.1143 $[M+H]^+$, calculated 326.1135 for $C_{17}H_{15}N_3O_4$, $[M+H^+]$.

(*E*)-7-Amino-1-(2-carboxyvinyl)-3*H*-phenoxazin-3-iminium trifluoroacetate (S1)

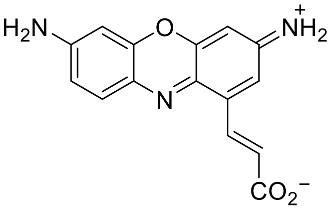

S1

$C_{15}H_{11}N_3O_3$
M 281.08

^{1}H NMR (400 MHz, Methanol-d_4) δ 8.21 (d, J = 16.2 Hz, 1H), 7.84 (d, J = 9.3 Hz, 1H), 7.36 (d, J = 2.2 Hz, 1H), 7.14 (dd, J = 9.3, 2.2 Hz, 1H), 6.82 (d, J = 16.3 Hz, 1H), 6.74 (d, J = 2.3 Hz, 1H), 6.73 (d, J = 2.3 Hz, 1H). ESI-HRMS: found 282.0877 $[M+H]^+$, calculated 282.0873 for $C_{15}H_{12}N_3O_3$, $[M+H]^+$. λ_{max} (absorption) 597 nm (ε = 105 000 $M^{-1}cm^{-1}$, EtOH), λ_{max} (em.) 607 nm (EtOH, excit. at 590 nm); fluorescence lifetime 3.4 ns (EtOH), fluorescence quantum yield 0.46 (absolute value in EtOH).

***(E)*-3-(10-Acetyl-3,7-diamino-10*H*-phenoxazin-2-yl) acrylic acid (8b)**

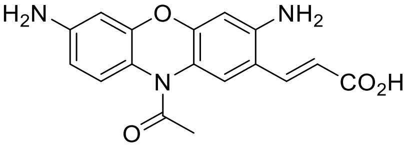

$C_{17}H_{15}N_3O_4$
M = 325.11

8b

Ester **7b** (30 mg, 0.05 mmol) was dissolved in DCM (2.5 ml), the solution cooled to +5°C in an ice bath, and then TFA (500 µl) was added dropwise with stirring. The reaction mixture was allowed to warm-up to r.t. and stirred for 18 h. The progress of the reaction was monitored by LC-MS (method A); educt **7b**, t_R 9.2 min, λ_{abs} 261, 331 nm, ESI-MS 582 $[M+H]^+$; product **8b**, t_R 5.2 min, λ_{abs} 260, 366 nm, ESI-MS 326 $[M+H]^+$; side product **S2**, t_R 4.7 min, λ_{abs} 597 nm, ESI-MS 282 $[M+H]^+$; side product **S3**, t_R 5.1 min, λ_{abs} 336 nm, ESI-MS 308 $[M+H]^+$. The volatile materials were removed in vacuo, the residue was co-evaporated with DCM (3 ×) and kept in vacuo (0.1 mbar) for 2 h. The title compound was isolated by prep. HPLC on Interchim puriFlash™ device with a 250 × 21.2 mm column (Knauer Eurosphere II 100-5 C18A, solvent A: H_2O + 0.1% v/v TFA; solvent B: MeCN + 0.1% v/v TFA). Gradient A/B: 90/10–0/100 in 25 min. Compound **8b** (3.0 mg, 18% yield) was isolated as a violet powder; dye

S2 was separated and isolated (2.0 mg, 14% yield), as well as lactam **S3** (6.5 mg, 42% yield). ^{1}H NMR (400 MHz, acetonitrile-d_3) δ 7.59 (d, *J* = 15.7 Hz, 1H), 7.47 (s, 1H), 7.18 (d, *J* = 8.2 Hz, 1H), 6.43 (s, 1H), 6.42 – 6.36 (m, 2H), 6.27 (d, *J* = 15.7 Hz, 1H), 2.18 (s, 3H). ESI-HRMS: found 326.1132 [M+H]$^+$, calculated 326.1135 for $C_{17}H_{15}N_3O_4$, [M+H$^+$].

(E)-7-Amino-2-(2-carboxyvinyl)-3*H*-phenoxazin-3-iminium trifluoroacetate (S2)

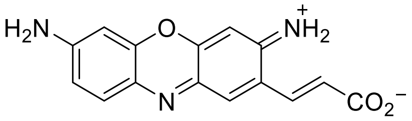

S2

$C_{15}H_{11}N_3O_3$
M = 281.08

^{1}H NMR (600 MHz, methanol-d_4) δ 7.93 (s, 1H), 7.78 (d, *J* = 9.2 Hz, 1H), 7.44 (d, *J* = 15.5 Hz, 1H), 7.16 (dd, *J* = 9.2, 2.2 Hz, 1H), 6.82 (s, 1H), 6.77 (d, *J* = 2.3 Hz, 1H), 6.57 (d, *J* = 15.6 Hz, 1H). ESI-HRMS: found 282.0874 [M+H]$^+$, calculated 282.0873 for $C_{15}H_{12}N_3O_3$, [M+H]$^+$. λ_{max} (absorption) 604 nm (ε = 100 000 M^{-1}cm^{-1}, EtOH), λ_{max} (em.) 630 nm (EtOH, excit. at 590 nm); fluorescence lifetime 2.4 ns (EtOH), fluorescence quantum yield 0.41 (absolute value in EtOH).

6-Acetyl-9-amino-1,6-dihydro-2*H*-pyrido[3,2-b]phenoxazin-2-one (S3)

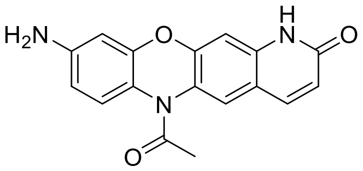

$C_{17}H_{13}N_3O_3$
M = 307.10

S3

^{1}H NMR (400 MHz, DMSO-d_6) δ 7.88 (d, *J* = 9.6 Hz, 1H), 7.86 (s, 1H), 7.21 (d, *J* = 8.5 Hz, 2H), 6.99 (s, 1H), 6.45 – 6.38 (m, 3H), 6.35 (dd, *J* = 12.6 and 2.6 Hz, 1H), 2.22 (s, 3H). ESI-HRMS: found 308.1039 [M+H]$^+$, calculated 308.1035 for $C_{17}H_{14}N_3O_3$, [M+H]$^+$.

(*E*)-3-(10-Acetyl-3,7-diamino-10*H*-phenoxazin-4-yl) acrylic acid (8c)

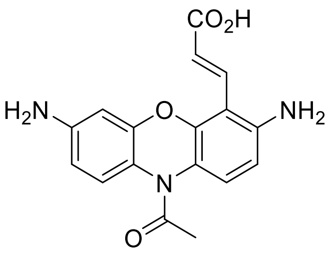

$C_{17}H_{15}N_3O_4$
M = 325.11

8c

The mixture containing esters **7b** and **7c** ~1:1 (58 mg, 0.1 mmol) was dissolved in DCM (5 ml), the solution cooled to +5°C in an ice bath, and then TFA (1 ml) was added dropwise with stirring. The reaction mixture was allowed to warm-up to r.t. and stirred for 18 h. The progress of the reaction was monitored by LC-MS (method A); educt **7c**, t_R 9.1 min, λ_{abs} 248,

288, 358 nm, ESI M+ 582, $[M+H]^+$; product **8c** t_R 5.3 min, λ_{abs} 292, 365 nm, ESI M+ 326 $[M+H]^+$). The volatile materials were removed in vacuo, the residue was co-evaporated with DCM (3×) and kept in vacuo (0.1 mbar) for 2 h. The title compound was isolated by flash chromatography on Interchim puriFlash™ with a 250 × 21.2 mm column (Knauer Eurosphere II 100-5 C18A, solvent A: H_2O + 0.1% v/v TFA; solvent B: MeCN + 0.1% v/v TFA. Gradient A/B: 95/5–0/100 in 25 min). Compound **8c** (3 mg, 18% yield) was isolated as a violet powder. The dye (compound **8c** without acetyl group) and lactam (structural analog of compound **S3**) were observed, but not isolated.

^{1}H NMR (400 MHz, acetonitrile-d_3) δ 7.79 (d, *J* = 16.3 Hz, 1H), 7.22 (d, *J* = 8.8 Hz, 1H), 7.17 (d, *J* = 8.6 Hz, 1H), 6.66 (d, *J* = 16.3 Hz, 1H), 6.51 (d, *J* = 8.8 Hz, 1H), 6.47 (d, *J* = 2.5 Hz, 1H), 6.42 (dd, *J* = 8.5, 2.5 Hz, 1H), 2.16 (s, 3H). ESI-HRMS: found 326.1133 $[M+H]^+$, calculated 326.1135 for $C_{17}H_{15}N_3O_4$, $[M+H^+]$.

Di-*tert*-butyl (10-acetyl-10*H*-phenoxazine-3,7-diyl)bis(methylcarbamate) (9)

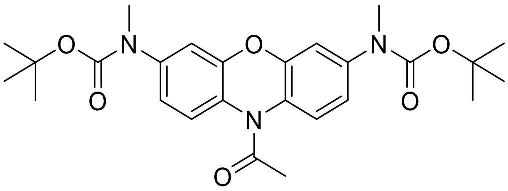

9

$C_{26}H_{33}N_3O_6$
M = 483.24

To a solution of **6**-O*t*Bu (225 mg, 0.5 mmol) in dry THF (8 ml) were added NaH (60% dispersion in mineral oil, 2.2 eq., 1.1 mmol, 45 mg) and MeI (10 eq, 620 µl) at 0 ºC, and the reaction mixture was stirred for 2 h The course of the reaction was monitored by LC-MS (method B); starting material **6**-O*t*Bu, t_R 3.7 min, λ_{abs} 286 nm, ESI MS: 456 $[M+H]^+$; product **9**: t_R 3.9 min, λ_{abs} 283 nm, ESI-MS 484 $[M+H]^+$. The reaction mixture was conc. in vacuo, and submitted to flash chromatography (SNAP Ultra cartridge with 25 g SiO_2, gradient of 10-100% EtOAc in hexane over 5 CV) to provide compound **9** (80 mg, 33% yield) as a yellowish powder. ^{1}H NMR (400 MHz, Acetone-d_6) δ 7.56 (d, *J* = 8.7 Hz, 2H), 7.16 (d, *J* = 2.3 Hz, 2H), 7.13 (dd, *J* = 8.6, 2.4 Hz, 2H), 3.26 (s, 6H), 2.31 (s, 3H), 1.45 (s, 18H). ESI-HRMS: found 506.2276 $[M+Na]^+$, calculated 506.2262 for $C_{26}H_{33}N_3O_6$, $[M+Na^+]$.

***tert*-Butyl (*E*)-3-(10-acetyl-3,7-bis[(*tert*-butoxycarbonyl)(methyl)amino]-10*H*-phenoxazin-1-yl) acrylate (10-Boc-*t*Bu)**

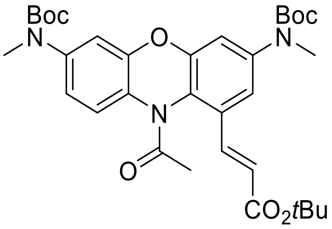

10-Boc-*t*Bu

$C_{33}H_{43}N_3O_8$
M = 609.31

Compound **9** (80 mg, 0.17 mmol), *tert*-butyl acrylate (1.5 eq, 35 µl, 0.24 mmol), $[RhCp^*(MeCN)_3](SbF_6)_2$ (14 mg, 17 µmol), $Cu(OAc)_2$ (30 mg, 0.17 mmol) were suspended in 3 ml acetone and placed into a sealed tube. The reaction mixture was stirred at 70°C for 18 h. The reaction progress was monitored by LC-MS (method B); starting compound **9**, t_R 3.9 min, λ_{abs} 283 nm, ESI-MS 484, $[M+H]^+$; product **10**-Boc-*t*Bu, t_R 4.6 min, λ_{abs} 246, 282 nm, ESI-MS 610 $[M+H]^+$. The mixture was diluted with water (50 ml) and extracted with EtOAc (3×50 ml). The combined organic solutions were washed with brine and dried over anhydrous $MgSO_4$. After filtration and evaporation of the solvents under reduced pressure, the product was isolated by column chromatography from the residue (SNAP Ultra cartridge with 50 g SiO_2, gradient of 10-100% EtOAc in hexane over 5 CV). Yield 49 mg (47%) of compound **10**-Boc-O*t*Bu as a yellow powder. ^1H NMR (400 MHz, acetone-d_6) δ 7.68 (d, J = 8.5 Hz, 1H), 7.64 (d, J = 16.0 Hz, 1H), 7.61 (d, J = 2.3 Hz, 1H), 7.29 (d, J = 2.3 Hz, 1H), 7.25 (d, J = 2.3 Hz, 1H), 7.22 (dd, J = 8.5, 2.4 Hz, 1H), 6.52 (d, J = 16.0 Hz, 1H), 3.32 (s, 3H), 3.28 (s, 3H), 2.20 (s, 3H), 1.52 (s, 9H), 1.46 (s, 9H), 1.45 (s, 9H). ^{13}C NMR (101 MHz, Acetone-d_6) δ 169.9, 165.4, 153.6, 152.1, 143.3, 143.0, 138.7, 131.1, 127.2, 125.8, 125.5, 121.1, 120.4, 117.6, 114.9, 113.5, 81.0, 80.0, 79.8, 36.4, 36.3, 27.4, 27.3, 21.4. ESI-HRMS: found 632.2973 $[M+Na]^+$, calculated 632.2942 for $C_{33}H_{43}N_3O_8$ $[M+Na^+]$.

(*E*)-3-(10-acetyl-3,7-bis(methylamino)-10*H*-phenoxazin-1-yl) acrylic acid (10-H-H)

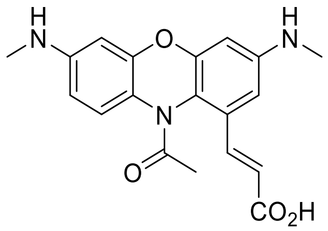

10-H-H

$C_{19}H_{19}N_3O_4$
M = 353.14

Ester **10**-Boc-O*t*Bu (49 mg, 0.08 mmol) was dissolved in 1,2-dichloroethane (5 ml), the solution cooled to +5°C in an ice bath, and then TFA (1 ml) was added dropwise with stirring. The reaction mixture was allowed to warm-up to r.t. and stirred for 18 h. The course of the reaction was monitored by LCMS (method B), starting compound **10**-Boc-O*t*Bu t_R 4.6 min, λ_{abs} 246, 282 nm, ESI M^+ 610, $[M+H]^+$; product **10**-H-H t_R 3.7 min, λ_{abs} 260, 370 nm, ESI M^+ 354 $[M+H]^+$; dye product (**S4**) t_R 1.9 min, λ_{abs} 616 nm, ESI M^+ 310 $[M]^+$). The volatile materials were removed in vacuo, the residue co-evaporated with DCM (3×) and kept in vacuo (0.1 mbar) for 2 h. The title compound was isolated by prep. HPLC on Interchim puriFlash™ with a 250 × 21.2 mm column (Knauer Eurosphere II 100-5 C18A, solvent A: H_2O + 0.1% v/v TFA; solvent B: MeCN + 0.1% v/v TFA. Gradient A/B: 90/10–0/100 in 25 min). Compound **10**-H-

H (23 mg, 81% yield) was isolated as a violet powder; and dye product (**S4**) was separated (3 mg, 12% yield). ^{1}H NMR (400 MHz, acetonitrile-d_3) δ 7.62 (d, *J* = 16.1 Hz, 1H), 7.34 (d, *J* = 8.6 Hz, 1H), 6.80 (d, *J* = 2.5 Hz, 1H), 6.59 (dd, *J* = 8.6, 2.5 Hz, 1H), 6.56 (d, *J* = 2.5 Hz, 1H), 6.54 (d, *J* = 2.5 Hz, 1H), 6.45 (d, *J* = 16.0 Hz, 1H), 2.81 (s, 3H), 2.81 (s, 3H), 2.07 (s, 3H). ESI-HRMS: found 354.1445 $[M+H]^+$, calculated 354.1148 for $C_{19}H_{19}N_3O_4$ $[M+H^+]$.

_N_-((_Z_)-1-((_E_)-2-carboxyvinyl)-7-(methylamino)-3_H_-phenoxazin-3-ylidene) methanaminium trifluoroacetate (S4)

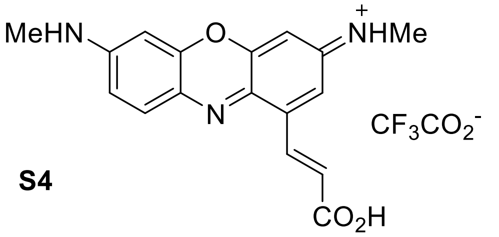

S4

$C_{17}H_{16}N_3O_3^+$
M = 310.12

This structure was assigned tentatively (based on the similarity of chemical transformations proved for the analogs listed above). ESI-HRMS: found 310.1195 $[M^+]$, calculated 310.1192 for $C_{17}H_{16}N_3O_3$, $[M^+]$. λ_{max} (abs.) 616 nm (ε = 90 000 $M^{-1}cm^{-1}$, EtOH), λ_{max} (em.) 627 nm (EtOH, excitation at 610 nm); fluorescence lifetime 1.8 ns (EtOH), fluorescence quantum yield 0.24 (absolute value in EtOH).

Benzyl (_E_)-3-(10-acetyl-3,7-bis(((benzyloxy)carbonyl)amino)-10_H_-phenoxazin-1-yl) acrylate (11-CO_2Bn**)**

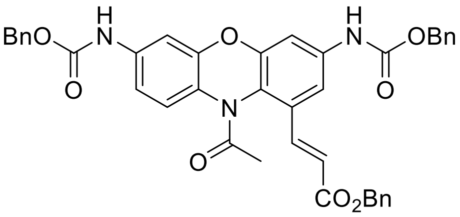

Chemical Formula: $C_{40}H_{33}N_3O_8$
Exact Mass: 683.23

11-OBn

Compound **6-**CO_2Bn (150 mg, 0.3 mmol), benzyl acrylate (1.5 eq, 70 µl, 0.45 mmol), $[RuCl_2(p\text{-cymene})]_2$ (19 mg, 0.03 mol), $AgSbF_6$ (21 mg, 0.06 mmol) and $Cu(OAc)_2{\cdot}H_2O$ (60 mg, 0.3 mmol) were suspended in 5 ml of acetone and placed in a sealed tube. The reaction mixture was stirred at 70 °C overnight. The reaction completion was monitored by LCMS (method A); starting compound **6-**OBn t_R 8.0 min, λ_{abs} 269 nm, ESI M^+ 524, $[M+H]^+$; product **11-**OBn t_R 9.4 min, λ_{abs} 286, 340 nm, ESI M^+ 684 $[M+H]^+$). The mixture was diluted with water (50 ml) and extracted with EtOAc (3x50 ml). The combined organic extract was washed with brine and dried over anhydrous $MgSO_4$. After filtration and evaporation of the solvents under reduced pressure, the crude product was purified by column chromatography (SNAP Ultra

cartridge with 50 g SiO_2, hexane/EtOAc with 10-100% EtOAc gradient over 5 CV). Yield 18 mg of yellow powder (9%).

^{1}H NMR (400 MHz, acetone-d_6) δ 9.01 (s, 1H, NH), 8.97 (s, 1H, NH), 7.77 (d, *J* = 11.4 Hz, 1H), 7.76 (d, *J* = 2.4 Hz, 1H), 7.63 (d, *J* = 2.0 Hz, 2H), 7.61 (d, *J* = 8.7 Hz, 1H), 7.48 – 7.33 (m, 16H), 6.51 (d, *J* = 16.0 Hz, 1H), 5.26 (d, *J* = 2.1 Hz, 2H), 5.20 (d, *J* = 1.6 Hz, 4H), 2.18 (s, 3H).

^{13}C NMR (101 MHz, acetone-d_6) δ 171.3, 166.9, 154.4, 153.8, 152.9, 141.6, 137.6, 132.9, 129.43, 129.41, 129.40, 129.14, 129.11, 129.06, 128.96, 127.1, 126.1, 119.8, 114.4, 111.6, 109.0, 107.4, 67.4, 67.3, 66.8, 22.5. ESI-HRMS: found 684.2330 $[M]^+$, calculated 684.2340 for $C_{40}H_{33}N_3O_8$, $[M+H]^+$.

***tert*-Butyl (*E*)-3-(10-acetyl-3,7-bis(2,2,2-trifluoroacetamido)-10*H*-phenoxazin-1-yl) acrylate (11-**CF_3**)**

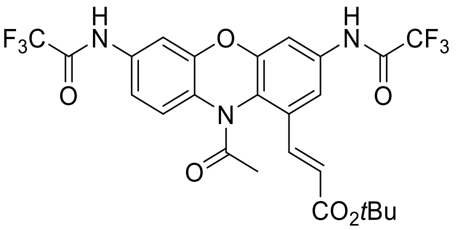

11-CF_3

$C_{25}H_{21}F_6N_3O_6$
M = 573.13

Compound **6**-CF_3 (90 mg, 0.2 mmol), *tert*-butyl acrylate (1.5 eq, 45 µl, 0.3 mmol), $[RhCp^*(MeCN)_3](SbF_6)_2$ (10 mg, 20 µmol), $Cu(OAc)_2$ (37 mg, 0.2 mmol) were suspended in 4 ml acetone and placed into a sealed tube under Ar. The reaction mixture was stirred at 70°C overnight. The completion of the reaction was detected by LCMS (method A; starting compound **6**-CF_3 t_R 3.0 min, λ_{abs} 238, 293 nm, ESI-MS 448 $[M+H]^+$; product **11**-CF_3 t_R 3.7 min, λ_{abs} 250 nm, ESI-MS 574 $[M+H]^+$). The reaction mixture was diluted with water (50 ml) and extracted with EtOAc (3×50 ml). The combined organic solutions were washed with brine and dried over anhydrous $MgSO_4$. The product was isolated by column chromatography (SNAP Ultra cartridge with 50 g SiO_2, gradient of 20-100% EtOAc in hexane over 5 CV). Yield - 40 mg of yellow powder (38%). ^{1}H NMR (400 MHz, acetone-d_6) δ 10.46 (s, 1H, NH), 10.38 (s, 1H, NH), 7.93 (d, *J* = 2.3 Hz, 1H), 7.79 – 7.76 (m, 3H), 7.62 (d, *J* = 16.0 Hz, 1H), 7.59 (dd, *J* = 8.1, 2.2 Hz, 1H), 6.41 (d, *J* = 16.0 Hz, 1H), 2.24 (s, 3H), 1.52 (s, 9H). ESI-HRMS: found 596.1256 $[M+Na]^+$, calculated 596.1227 for $C_{25}H_{21}N_3O_6F_6$ $[M+Na]^+$.

10-Acetyl-*10H*-phenoxazine-3,7-diyl diacetate (13)

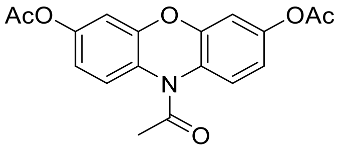

13

$C_{18}H_{15}NO_6$
M = 341.09

An oven-dried Schlenk flask was charged with Pd/C (100 mg), resazurin sodium salt **12** (300 mg, 1.2 mmol) was added followed by Ac_2O (10 ml), and the reaction mixture was stirred under Ar for 10 min. Then the reaction mixture was flushed with hydrogen and vigorously stirred overnight under hydrogen at r.t. The progress of the reaction was monitored by TLC (hexane/EtOAc 1:1, R_f 0.3 of the product; the spot of the starting material remain on the start) and LCMS (method A, product **13**: t_R 5.6 min, λ_{abs} 281 nm, ESI M^+ 342 $[M+H]^+$). A full conversion was detected, and no side products observed. The reaction mixture was diluted with water (50 ml) and extracted with EtOAc (3×150 ml). The combined organic solutions were washed with brine, dried over anhydrous $MgSO_4$, and evaporated to afford a brown residue. It was subjected to flash chromatography (SNAP Ultra cartridge with 50 g SiO_2, elution with 20-100% gradient of EtOAc in hexane over 5 CV) to provide compound **13** as a yellow solid (380 mg, yield 93%). ^1H NMR (400 MHz, acetone-d_6) δ 7.63 (dd, J = 8.7, 0.4 Hz, 2H), 7.00 (d, J = 2.3 Hz, 2H), 6.97 (dd, J = 8.7, 2.6 Hz, 2H), 2.32 (s, 3H), 2.27 (s, 6H). ESI-HRMS: found 342.0968 $[M+H]^+$, calculated 342.0972 for $C_{18}H_{15}NO_6$ $[M+H]^+$.

(*E*)-10-Acetyl-1-(3-(*tert*-butoxy)-3-oxoprop-1-en-1-yl)-10*H*-phenoxazine-3,7-diyl diacetate (14)

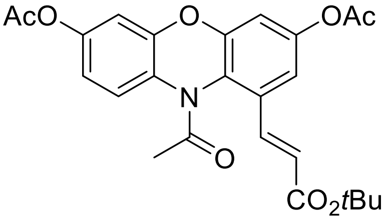

14

$C_{25}H_{25}NO_8$
M = 467.16

Triacetate **13** (340 mg, 1.00 mmol), *tert*-butyl acrylate (220 µl, 196 mg, 1.5 mmol), $[RuCl_2(p\text{-cymene})]_2$ (60 mg, 10 mol %), $AgSbF_6$ (70 mg, 20 mol %) and $Cu(OAc)_2{\times}H_2O$ (200 mg, 1 mmol) were suspended in 20 ml acetone, purged with Ar and stirred at 70°C overnight in a sealed tube. The progress of the reaction was monitored by TLC (hexane-EtOAc 1:1, product R_f 0.5, starting material R_f 0.3) and LCMS (method A): starting compound **13** t_R 5.6 min, λ_{abs} 281 nm, ESI-MS 342 $[M+H]^+$; product **14** t_R 7.5 min, λ_{abs} 280 nm, ESI-MS 468 $[M+H]^+$. The mixture was diluted with water (50 ml) and extracted with EtOAc (3×50 ml). The combined organic solutions were washed with brine and dried over anhydrous $MgSO_4$. The crude product was purified by flash chromatography (SNAP Ultra cartridge with 50 g SiO_2, gradient of 20-100% EtOAc in hexane over 5 CV). Yield 56% (263 mg of ester **14** as a yellow

solid). ^{1}H NMR (400 MHz, acetone-d_6) δ 7.74 (dd, J = 8.5, 0.4 Hz, 1H), 7.62 (d, J = 16.0 Hz, 1H), 7.45 (dd, J = 2.4, 0.4 Hz, 1H), 7.08 (d, J = 2.4 Hz, 1H), 7.04 (dd, J = 8.6 and 2.5 Hz, 1H), 6.51 (d, J = 16.0 Hz, 1H), 2.28/2.29 (two s, 6H), 2.20 (s, 3H), 1.51 (s, 9H). ^{13}C NMR (101 MHz, acetone-d_6) δ 170.9, 169.5, 166.2, 153.6, 152.7, 150.8, 150.6, 139.1, 133.1, 128.9, 127.5, 122.8, 118.4, 115.9, 112.9, 111.8, 81.0, 28.4, 22.4, 21.0. ESI-HRMS: found 490.1473 [M+Na]$^+$, calculated 490.1472 for $C_{25}H_{25}NO_8Na^+$ [M+Na$^+$].

***tert*-Butyl (*E*)-3-(10-acetyl-3,7-bis(((trifluoromethyl)sulfonyl)oxy)-10*H*-phenoxazin-1-yl) acrylate (S6)**

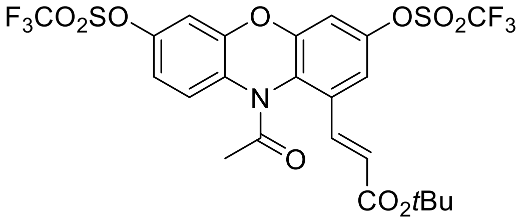

$C_{23}H_{19}F_6NO_{10}S_2$
M = 647.04

S6

1. *Saponification of diacetate **14**.* To a solution of diacetate **14** (100 mg, 0.21 mmol) in EtOH (6 ml), 5 M aq. NaOH was added dropwise (200 µl, 1.0 mmol, 5 eq). The color immediately turned to fuchsia. The reaction was stirred at r.t. for 1 h. The progress of the reaction was monitored by TLC (EtOAc, product R_f 0.5, starting material R_f 0.75) and LCMS (method B; starting compound **14** t_R 7.5 min, λ_{abs} 280 nm, ESI-MS 468 [M+H]$^+$; product t_R 5.6 min, λ_{abs} 284, 336 nm, ESI-MS 328 [M-*t*Bu+2H]$^+$). The red solution was acidified with 1 M aq. HCl (5 ml); the colour turned to yellow. The reaction mixture was diluted with water and extracted with EtOAc (2×50 ml). The combined organic solutions were washed with brine and dried over anhydrous $MgSO_4$. The solvents were evaporated under reduced pressure to provide a reddish solid (90 mg of a crude product) which was used in the next step without further purification

2. *Preparation of bis-triflate.* The crude material from the previous step was suspended in DCM (5 mL), and the solution cooled to 0°C. Pyridine (134 µl, 1.7 mmol, 8 eq) and trifluoromethanesulfonic anhydride (141 µl, 0.84 mmol, 4 eq) were added, and the ice bath removed. The reaction mixture was stirred at r.t. for 1 h. The progress of the reaction was monitored by TLC (hexane/EtOAc 4:1, product R_f 0.5; starting compound R_f 0) and LCMS (starting compound t_R 5.6 min, λ_{abs} 284 nm, ESI-MS 328 [M-*t*Bu+2H]$^+$; product **S6** t_R 10.1 min, λ_{abs} 281 nm, ESI-MS 592 [M-*t*Bu+2H]$^+$). The reaction mixture was diluted with water and extracted with DCM (2×50 ml). The combined organic solutions were dried over anhydrous $MgSO_4$, filtered, and evaporated. The crude product was purified by flash chromatography (Isolera SNAP Ultra cartridge with 50 g SiO_2, elution with 2-50-100% v/v EtOAc in hexane

over 5 CV). Yield 62% of **S6** (85 mg of a yellowish solid). ^{1}H NMR (400 MHz, chloroform-*d*) δ 7.56 (d, *J* = 8.3 Hz, 1H), 7.54 (d, *J* = 16.0 Hz, 1H), 7.35 (d, *J* = 2.7 Hz, 1H), 7.21 – 7.13 (m, 2H), 6.45 (d, *J* = 16.0 Hz, 1H), 2.15 (s, 3H), 1.52 (s, 9H). ^{19}F NMR (376 MHz, chloroform-*d*) δ -72.57, -72.59. ESI-HRMS: found 670.0250 $[M+Na]^+$, calculated 670.0247 for $C_{23}H_{19}NO_{10}F_6S_2$, $[M+Na]^+$.

***tert*-Butyl (*E*)-3-(10-acetyl-3,7-bis(dimethylamino)-10*H*-phenoxazin-1-yl)acrylate (10-Me-*t*Bu)**

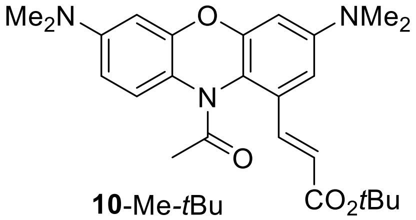

$C_{25}H_{31}N_3O_4$
M = 437.23

10-Me-*t*Bu

A Schlenk tube was charged with ditriflate **S6** (65 mg, 0.1 mmol), $Pd_2(dba)_3$ (9 mg, 0.01 mmol), XPhos (14 mg, 0.03 mmol and Cs_2CO_3 (98 mg, 0.30 mmol). The vial was sealed and evacuated/backfilled with Ar (3×). Dioxane (2 ml) was added followed by 2 M solution of Me_2NH in THF (0.4 ml), and the reaction mixture was stirred at 105°C for 18 h. The progress of the reaction was monitored by LCMS (method A); starting compound **S6** t_R 4.5 min, λ_{abs} 281 nm, ESI M^+ 592 $[M-^tBu+2H]^+$; product **10**-Me-*t*Bu t_R 4.9 min, λ_{abs} 267, 385 nm, ESI M^+ 438 $[M+H]^+$; side product **S7** (of C-H acetyl addition to the acrylate C=C bond; see structure below) t_R 4.2 min, λ_{abs} 238, 289 nm, ESI-MS 438 $[M+H]^+$. The reaction mixture was diluted with water and extracted with EtOAc (3×50 ml). The combined organic solutions were washed with brine and evaporated in vacuo. The product was isolated by flash chromatography (Isolera SNAP Ultra cartridge with 50 g SiO_2, with 10-100% v/v gradient of EtOAc in hexane). Yield of compound **16 -** 59% (27 mg of a white-rosy solid); side product **S7** – 9 mg (20%). ^{1}H NMR (400 MHz, Acetonitrile-d_3) δ 7.58 (d, *J* = 16.0 Hz, 1H), 7.49 (d, *J* = 9.1 Hz, 1H), 7.03 (d, *J* = 2.7 Hz, 1H), 6.89 (dd, *J* = 9 and 2.7 Hz, 1H), 6.88 (s, 1H), 6.80 (d, *J* = 2.7 Hz, 1H), 6.49 (d, *J* = 16.0 Hz, 1H), 3.03 (s, 6H), 3.02 (s, 6H), 2.09 (s, 3H), 1.51 (s, 9H). ^{13}C NMR (126 MHz, Acetonitrile-d_3) δ 172.8, 167.4, 154.7, 153.9, 150.5, 150.8, 141.2, 132.4, 127.4, 122.2, 121.7, 120.8, 109.3, 106.1, 103.7, 102.1, 81.6, 41.8, 41.6, 28.8, 23.0.

ESI-HRMS: found 438.2384 $[M+H]^+$, calculated 438.2387 for $C_{25}H_{31}N_3O_4$, $[M+H]^+$.

tert-Butyl 2-(5,9-bis(dimethylamino)-1-oxo-2,3-dihydro-1*H*-pyrido[3,2,1-kl] phenoxazin-3-yl) acetate (S7)

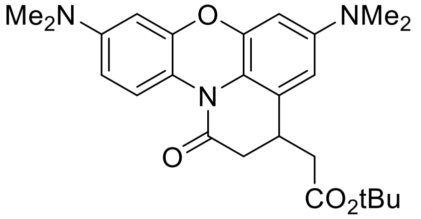

S7

$C_{25}H_{31}N_3O_4$
M = 437.23

^{1}H NMR (400 MHz, acetonitrile-d_3) δ 8.14 (d, *J* = 9.2 Hz, 1H), 6.45 (d, *J* = 9.0 Hz, 1H), 6.36 (d, *J* = 2.9 Hz, 1H), 6.32 (d, *J* = 6.7 Hz, 1H), 6.26 (d, *J* = 2.7 Hz, 1H), 3.33 (tt, *J* = 7.6, 5.5 Hz, 1H), 2.91 (s, 6H), 2.90 (s, 6H), 2.76 (dd, *J* = 16.2, 5.3 Hz, 1H), 2.55 (dd, *J* = 16.1, 5.6 Hz, 1H), 2.52–2.43 (m, 2H), 1.42 (s, 9H). ESI-HRMS: found 438.2389 $[M+H]^+$, calculated 438.2387 for $C_{25}H_{31}N_3O_4$ $[M+H]^+$.

Alternative synthesis of *tert*-butyl ester 10-Me-*t*Bu

***(E)*-3-(10-Acetyl-3,7-bis(dimethylamino)-10*H*-phenoxazin-1-yl) acrylic acid (10-Me-H)**

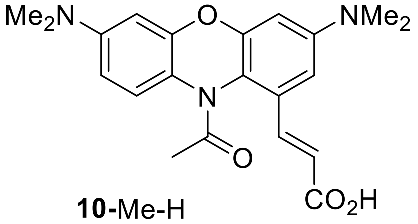

$C_{21}H_{23}N_3O_4$
M = 381.17

10-Me-H

Ester **10-**Me-*t*Bu (24 mg, 0.05 mmol) was dissolved in DCM (2 ml), the solution cooled to +5°C in an ice bath, and then TFA (400 µl) was added dropwise with stirring. The reaction mixture was allowed to warm-up to r.t. and stirred for 18 h. The course of the reaction was monitored by LCMS (method B, starting compound **10**-Me-*t*Bu: t_R 4.9 min, λ_{abs} 267, 385 nm, ESI-MS 438 $[M+H]^+$; product **10**-Me-H: t_R 2.8 min, λ_{abs} 268, 387 nm, ESI-MS 382 $[M+H]^+$; side product **S9**: t_R 1.6 min, λ_{abs} 646 nm, ESI-MS 338 $[M+H]^+$). The volatile materials were removed in vacuo, the residue was co-evaporated with DCM (3×) and kept in vacuo (0.1 mbar) for 2 h. The title compound was isolated by prep. HPLC on Interchim puriFlash™ device with a 250 × 21.2 mm column (Knauer Eurosphere II 100-5 C18A, solvent A: H_2O + 0.1% v/v TFA; solvent B: MeCN + 0.1% v/v TFA. Gradient A/B: 80/20–0/100 in 25 min). Compound **10**-Me-H (15 mg, 70% yield) was isolated as a bluish powder; along with compound **S9** (3 mg, 16% yield). ^{1}H NMR (400 MHz, acetonitrile-d_3) δ 7.94 (d, *J* = 16.1 Hz, 1H), 7.32 (d, *J* = 8.8 Hz, 1H), 6.88 (d, *J* = 2.8 Hz, 1H), 6.78 (d, *J* = 16.0 Hz, 1H), 6.64 (d, *J* = 2.7 Hz, 1H), 6.54 (dd, *J* = 8.8, 2.8 Hz, 1H), 6.50 (d, *J* = 2.8 Hz, 1H), 2.99 (s, 6H), 2.94 (s, 6H), 2.09 (s, 3H). ^{13}C NMR (126 MHz, acetonitrile-d_3) δ 172.9, 168.5, 154.7, 153.9, 151.2, 143.0, 132.1, 127.3, 121.1,

120.4, 119.5, 108.5, 105.7, 103.4, 101.2, 41.2, 23.0. HRMS (ESI) calcd for $C_{21}H_{23}N_3O_4$ [M-H]$^+$ 380.1616, found 380.1613.

In another run, TFA (0.3 mL) was added to the solution of ester **10**-Me-*t*Bu (5 mg, 11 μmol) in DCM (1.5 mL), and the reaction mixture was stirred for 5 h at r.t. The reaction course was monitored by TLC. Upon completion of the reaction, the solution was evaporated under reduced pressure, and the residue co-evaporated with acetonitrile. The product was isolated on SiO_2 (10 g cartridge) on a Biotage Isolera device using 0 – 10% MeOH in DCM to get compound **10**-Me-H (3.5 mg, 83%) as light green solid.

(*E*)-*N*-(1-(2-Carboxyvinyl)-7-(dimethylamino)-3*H*-phenoxazin-3-ylidene)-*N*-methylmethanaminium trifluoroacetate (S9)

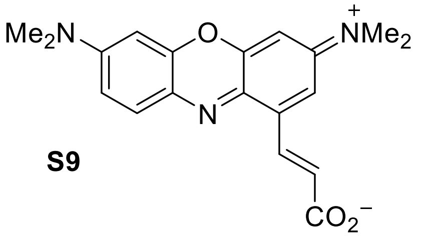

S9

$C_{19}H_{19}N_3O_3$
M = 337.15

^{1}H NMR (400 MHz, Methanol-d_4) δ 8.43 (d, J = 16.2 Hz, 1H), 7.89 (d, J = 9.6 Hz, 1H), 7.63 (d, J = 2.7 Hz, 1H), 7.45 (dd, J = 9.6, 2.7 Hz, 1H), 7.01 – 6.94 (m, J = 2.7 Hz, 3H), 3.45 (s, 12H). ESI-HRMS: found 338.1503 [M+H]$^+$, calculated 338.1499 for $C_{19}H_{20}N_3O_3$, [M+H]$^+$.

Alternative synthesis of ester 10-Me-*t*Bu

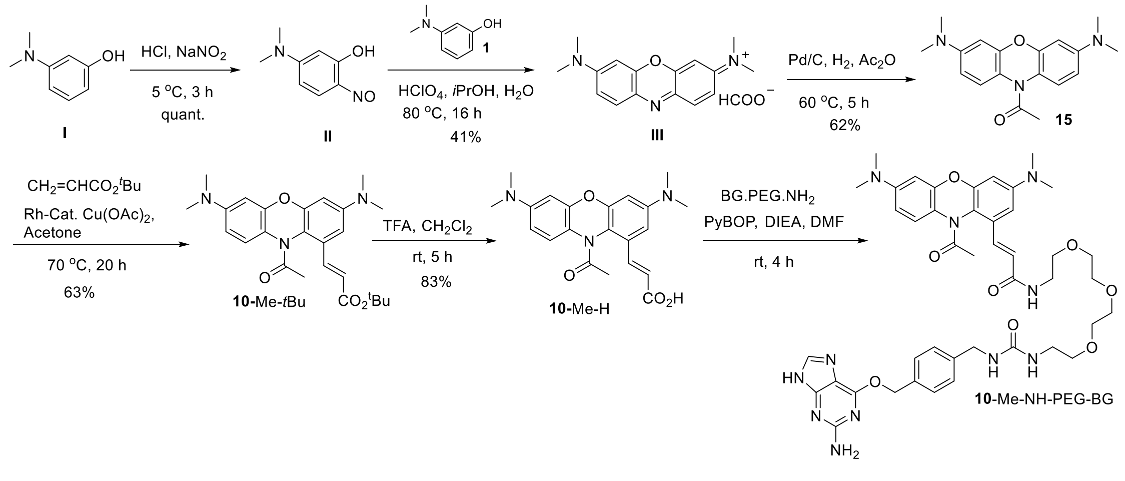

4-(Dimethylamino)-2-nitrosophenol (B): 3-Dimethylaminophenol (**I**) (2.00 g, 14.6 mmol) was dissolved in ice-cold 6 M aq. HCl (10 mL), then $NaNO_2$ (1.03 g, 14.9 mmol) was added in small portions over 1 h while maintaining the temperature of ca. +5 °C by cooling with ice water. The reaction mixture was stirred in an ice bath for 2 h. The formed precipitate was filtered off and washed with small portions of ice cold 2 M aq. HCl solution. The product was

dried in air and then under vacuum to get compound **II** (2.0 g) as light brown solid. The crude material was used in the next step without further purification.

Oxazine dye III: 3-Dimethylaminophenol (**I**) (500 mg, 3.64 mmol) was dissolved in aq. isopropanol *i*PrOH/H_2O = 9/1 v/v (10 mL), and then a suspension of 4-(dimethylamino)-2-nitrosophenol (**II**) (640 mg, 3.85 mmol) and $HClO_4$ (70%, 350 µL) in 90% v/v aq. *i*PrOH (20 mL, *i*PrOH/H_2O, 18/2) was added in 4 portions over 1 h. The reaction mixture was heated at 80 ^oC for 16 h. The dark blue solution was evaporated under reduced pressure, and the residue was purified on a Biotage Isolera device using a 25 g SiO_2 cartridge (with spheric SiO_2) and eluting with 0–15% MeOH in DCM (+0.25% v/v HCO_2H in both components). The fractions with product were pooled and evaporated to obtain dye **III** (450 mg, 41%) as dark blue solid. ^1H NMR (400 MHz, Acetonitrile-d_3) δ 7.79 (d, J = 9.6 Hz, 2H), 7.32 (dd, J = 9.6, 2.7 Hz, 2H), 6.83 (d, J = 2.7 Hz, 2H), 3.37 (s, 12H). ^{13}C NMR (126 MHz, acetonitrile-d_3) δ 158.8, 150.1, 135.3, 134.9, 97.2, 42.1. HRMS (ESI) calcd for $C_{16}H_{18}N_3O^+$ 268.1444, found 268.1443.

1-(3,7-*bis*(Dimethylamino)-10*H*-phenoxazin-10-yl)ethan-1-on (15): A mixture of dye **III** (98 mg, 0.31 mmol), Pd/C (50 mg) and Ac_2O (7 ml) in a 25 mL flask was heated at 60 ^oC for 5 h, monitored by TLC. The reaction mixture was filtered through Celite, washed with ethyl acetate and the filtrate was evaporated. The crude product was purified on a Biotage Isolera flash chromatography device using a 10 g SiO_2 cartridge and eluting with a gradient of 0 – 10% MeOH in DCM to get compound **15** (60 mg, 62%) as a pale green solid. ^1H NMR (400 MHz, acetonitrile-d_3) δ 7.31 (d, J = 8.8 Hz, 2H), 6.51 (dd, J = 8.8, 2.8 Hz, 2H), 6.47 (d, J = 2.8 Hz, 2H), 2.94 (s, 12H), 2.20 (s, 3H). ^{13}C NMR (126 MHz, Acetonitrile-d_3) δ 170.3, 152.6, 150.7, 126.3, 120.1, 107.8, 100.8, 40.8, 23.0. HRMS (ESI) calcd for $C_{18}H_{21}N_3O_2$ $[M+Na]^+$ 334.1526, found 334.1523.

***tert*-Butyl (*E*)-3-(10-Acetyl-3,7-bis(dimethylamino)-10*H*-phenoxazin-1-yl)acrylate (10-Me-*t*Bu):** A mixture of compound **15** (22 mg, 0.07 mmol), $[RhCp^*(MeCN)_3](SbF_6)_2$ (6 mg, 0.007 mmol), $Cu(OAc)_2$ (13 mg, 1 eq., 0.07 mmol), and *tert*-butyl acrylate (1.2 eq., 16 µl, 0.08 mmol) were combined in acetone (1.5 ml) in a 10 mL microwave vial and stirred at 70 ºC for 20 h. After cooling to r.t., the reaction mixture was evaporated under reduced pressure, the residue was taken up in H_2O (25 ml) and extracted with EtOAc (3×). The combined organic solutions were washed with brine and dried over anhydrous Na_2SO_4. After filtration and evaporation of the solvents under reduced pressure, the residue was subjected to flash

chromatography (cartridge with 10 g SiO_2). Elution with hexane/EtOAc mixture with 5-100% EtOAc gradient over 10 CV) gave compound **10**-Me-*t*Bu as greenish solid (22 mg, 63%). The substance contains bis-acrylated product as impurity. The product was re-purified by prep. HPLC on reversed phase to get pure compound **10**-Me-*t*Bu (12 mg) as a pale green solid. The NMR spectra are given above. HRMS (ESI) calcd for $C_{25}H_{31}N_3O_4$ $[M+Na]^+$ 460.2207, found 460.2206.

10-Me-NH-PEG-BG (see Scheme 7 in the main text)**:** Compound **10**-Me-H (1.56 mg, 4 µmol, 1 eq), BG-PEG-NH_2 (2 mg, 6 µmol, 1.5 eq) and DIPEA (5 µL, 40 µmol, 10 eq) were taken in dry DMF (1 mL) and then PyBOP (3.21 mg, 6 µmol, 1.5 eq) was added at rt. The reaction mixture was stirred at rt for 4 h. The completion of the reaction was confirmed by LCMS. The reaction mixture was conc. under vacuum, and the residue purified by prep. HPLC (Interchim, 16 × 250 mm column C18, A/B 20/80 → 0/100 in 25 min, A – acetonitrile, B – water + 0.1% TFA in both components). Yield: 1 mg (64%) of light green solid. HRMS (ESI) calcd for $C_{43}H_{53}N_{11}O_8$ $[M+Na]^+$ 874.3971, found 874.3961.

Preparative photolysis of compound 2-H

(see Scheme 4 in the main text and Figure S4)

(*E*)-*N*-(7-(Diethylamino)-1-(3-oxobut-1-en-1-yl)-3*H*-phenoxazin-3-ylidene)-*N*-ethylethanaminium trifluoroacetate (ES-1006; main product)

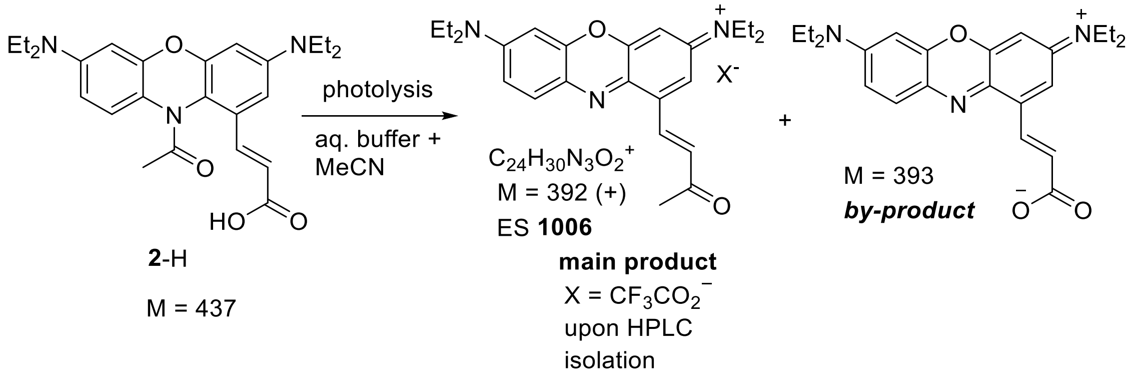

Compound **2**-H (7 mg, 0.016 mmol) was dissolved in 5.0 ml MeCN, and the stirred solution irradiated with commercially available UV Mounted LED 365 nm (Thor labs M365L3, Output Power 880 mW / 1290 mW, Band width 9 nm). The course of photolysis was controlled by LC-MS (method B); starting compound **2**-H: t_R 2.6 min, λ_{abs} 273, 392 nm, ESI-MS 438 $[M+H]^+$; main product: t_R 2.5 min, λ_{abs} 659 nm, ESI-MS 392 $[M]^+$; by-product: t_R 2.3 min, λ_{abs} 660 nm, ESI-MS: 394 $[M+H]^+$). The full conversion was achieved in 7 h. The solvent was removed in vacuo, and the residue subjected to prep. HPLC on an Interchim puriFlash™ device equipped with a 250 × 21.2 mm column (Knauer Eurosphere II 100-5 C18A, H_2O (0.1% v/v

TFA) - MeCN (0.1% v/v TFA), gradient of MeCN 10 – 100% in 25 min). The title compound **ES-1006** (4 mg of blue powder) was isolated as main product in 64% yield. The by-product was detected by means of LC-MS (see Figure S4). **ES-1006**, ^{1}H NMR (400 MHz, acetonitrile-d_3) δ 8.27 (d, J = 16.5 Hz, 1H), 7.82 (d, J = 9.7 Hz, 1H), 7.48 (d, J = 2.7 Hz, 1H), 7.32 (dd, J = 9.6, 2.7 Hz, 1H), 7.20 (d, J = 16.5 Hz, 1H), 6.82 (dd, J = 5.6, 2.7 Hz, 2H), 3.73 (m, 8H), 2.43 (s, 3H), 1.31 (t, J = 7.2 Hz, 12H). ^{13}C NMR (126 MHz, acetonitrile-d_3) δ 199.4, 158.0, 156.5, 150.92, 150.88, 139.3, 136.3, 135.9, 135.4, 133.8, 133.6, 119.4, 115.4, 98.4, 97.4, 48.1, 47.8, 28.8, 13.5. ESI-HRMS: found 392.2336 $[M]^+$, calculated 392.2333 for $C_{24}H_{30}N_3O_2$, $[M]^+$. λ_{max} (abs.) 659 nm (ε = 79 000 $M^{-1}cm^{-1}$, EtOH), λ_{max} (em.) 676 nm (EtOH, excitation at 640 nm), fluorescence lifetime 1.5 ns (EtOH), fluorescence quantum yield 0.11 (absolute value in EtOH).

Synthesis of amides, including HaloTag ligands

(*E*)-3-(10-Acetyl-3,7-bis(methylamino)-10*H*-phenoxazin-1-yl)-*N*-(2-methoxyethyl) acrylamide (Scheme 7, main text)

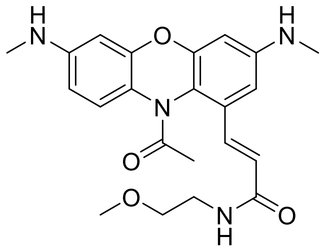

$C_{22}H_{26}N_4O_4$
M = 410.20

ES 1081

Compound **10**-H-H (Scheme 5 in the main text; 17 mg, 50 µmol) was dissolved in dry DMF (1.5 ml). TSTU (25 mg, 80 µmol, 1.6 eq) and DIPEA (35 µL, 0.2 mol, 4 eq) were added at r.t., and the reaction mixture was stirred for 10 min. 2-Metoxyethylamine (5.3 µl, 60 µmol, 1.2 eq) was added, and the reaction mixture was stirred for 2 h. The course of the reaction was monitored by LCMS (Method B); starting compound **10**-H-H t_R 1.4 min, λ_{abs} 260 nm, ESI MS 354 $[M+H]^+$; product **ES1081** t_R 1.6 min, λ_{abs} 260, 365 nm, ESI-MS 411 $[M+H]^+$. The reaction mixture was conc. in vacuum, and the residue subjected to prep. HPLC on an Interchim puriFlash™ device with a 250 × 21.2 mm column (Knauer Eurosphere II 100-5 C18A, H_2O (0.1% v/v TFA) - MeCN (0.1% v/v TFA), gradient of MeCN 10 – 100% in 25 min). The product (4 mg, 19%) was isolated as bluish powder.^{1}H NMR (400 MHz, Acetonitrile-d_3) δ 7.45 (d, J = 15.7 Hz, 1H), 7.28 (d, J = 8.5 Hz, 1H), 6.71 (br. s, J = 2.5 Hz, 1H, NHCO), 6.67 (d, J = 2.5 Hz, 1H), 6.53 (d, J = 15.8 Hz, 1H), 6.45 (d, J = 8.7, 2.5 Hz, 1H), 6.44 (d, J = 2.5 Hz, 1H), 3.44 (m, 4H), 3.32 (s, 3H), 2.79 (s, 3H), 2.77 (s, 3H), 2.04 (s, 3H). ESI-HRMS: found 411.2035 $[M]^+$, calculated 411.2027 for $C_{22}H_{26}N_4O_4$, $[M+H]^+$.

(*E*)-3-(10-Acetyl-3,7-bis(diethylamino)-10*H*-phenoxazin-1-yl)-*N*-(2-(2-((6-chlorohexyl)oxy)ethyloxy)ethyl)acrylamide (2-Halo, CA3)

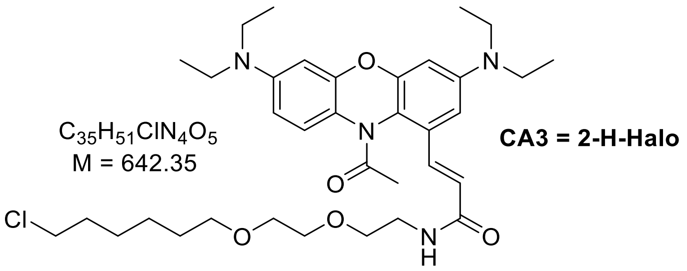

Compound **2**-H (62 mg, 0.14 mmol) was dissolved in dry DMF (2 ml). TSTU (68 mg, 0.22 mmol, 1.6 eq) and *N,N*-diisopropylethylamine (250 µl, 1.4 mmol, 10 eq) were added at r.t., and the reaction mixture was stirred for 10 min. $Cl(CH_2)_6O(CH_2)_2O(CH_2)_2NH_2$*HCl (47 mg, 0.18 mmol, 1.25 eq) was added and the reaction mixture was stirred for 2 h; then, additional amount (10 mg) of $Cl(CH_2)_6O(CH_2)_2O(CH_2)_2NH_2$*HCl was added. The reaction course was monitored by LCMS (method B): starting compound **2**-H t_R 2.6 min, λ_{abs} 273, 392 nm, ESI-MS 438 $[M+H]^+$; the product **CA3** t_R 3.6 min, λ_{abs} 272, 385 nm, ESI-MS 643 $[M+H]^+$. The reaction mixture was conc. in vacuum, and the residue separated on an Interchim puriFlash™ device with a 250 × 21.2 mm column (Knauer Eurosphere II 100-5 C18A, H_2O (0.1% v/v TFA) - MeCN (0.1% v/v TFA), gradient of MeCN 10 – 100% in 25 min). 28 mg of bluish powder of compound **2**-Halo = **CA3** was isolated (31% yield).

^{1}H NMR (400 MHz, Acetonitrile-d_3) δ 7.48 (d, *J* = 15.7 Hz, 1H), 7.33 (d, *J* = 8.6 Hz, 1H), 6.74 (d, *J* = 2.8 Hz, 1H), 6.58 (d, *J* = 15.8 Hz), 6.56 – 6.49 (m, 3H), 3.61 – 3.49 (m, 8H), 3.46 – 3.33 (m, 12H), 2.05 (s, 3H), 1.68 (dq, *J* = 14.1, 6.8 Hz, 2H), 1.51 (p, *J* = 6.8 Hz, 2H), 1.40 – 1.25 (m, 4H), 1.13 ("dt", *J* = 10.3, 7.0 Hz, 12H). ESI-HRMS: found 643.3620 $[M+H]^+$, calculated 643.3621 for $C_{35}H_{51}N_4O_5Cl$, $[M+H]^+$.

(*E*)-3-(10-Acetyl-3,7-diamino-10*H*-phenoxazin-1-yl)-*N*-(2-(2-((6-chlorohexyl)oxy)ethoxy)ethyl) acrylamide (8a-Halo = CA1)

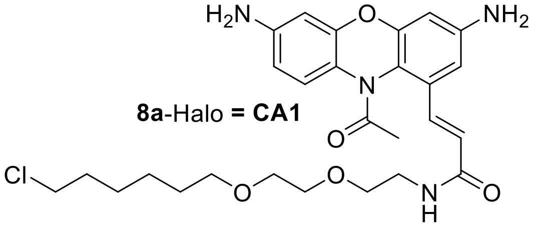

C27H35ClN4O5
M = 530.23

Compound **8a** (32 mg, 0.1 mmol) was dissolved in dry DMF (2 ml). TSTU (48 mg, 0.16 mmol, 1.6 eq) and DIPEA (176 µl, 1.4 mmol, 10 eq) were added at r.t., and the reaction mixture was stirred for 10 min. $Cl(CH_2)_6O(CH_2)_2O(CH_2)_2NH_2$*HCl (32 mg, 0.12 mmol, 1.25 eq) was

added and the reaction mixture stirred for 2 h. The reaction course was monitored by LCMS (method B), gradient 5/95-50/50, starting compound **8a** t_R 2.2 min, λ_{abs} 282, 350 nm, ESI-MS 326 $[M+H]^+$; the product **8a**-Halo = **CA1** t_R 4.3 min, λ_{abs} 287, 351 nm, ESI-MS 531 $[M+H]^+$). The reaction mixture was conc. in vacuum, and the residue separated on an Interchim puriFlash™ device with a 250 × 21.2 mm column (Knauer Eurosphere II 100-5 C18A, H_2O (0.1% v/v TFA) - MeCN (0.1% v/v TFA), gradient of MeCN 5 – 50% in 20 min). The amount of 5.4 mg of compound **8**-H-Halo = **CA1** was isolated as dark powder (10% yield). ^{1}H NMR (400 MHz, Acetonitrile-d_3) δ 7.42 (d, J = 15.7 Hz, 1H), 7.20 (d, J = 8.8 Hz, 1H), 6.70 (s, NHCO, 1H), 6.70 (d, J = 2.4 Hz, 1H), 6.46 (d, J = 16 Hz, 1H), 6.45 (d, J = 2.4 Hz, 1H), 6.43 (dd, J = 8 and 2.4 Hz, 1H), 6.42 (s, 1H), 3.59 – 3.50 (m, 8H), 3.46 – 3.39 (m, 4H), 2.03 (s, 3H), 1.80 – 1.66 (m, 2H), 1.58 – 1.48 (m, 2H), 1.47 – 1.21 (m, 4H). ESI-HRMS: found 531.2376 $[M+H]^+$, calculated 531.2369 for $C_{27}H_{35}N_4O_5Cl$, $[M+H]^+$.

(*E*)-3-(10-Acetyl-3,7-bis(methylamino)-10*H*-phenoxazin-1-yl)-*N*-(2-(2-((6-chlorohexyl)oxy)ethoxy)ethyl) acrylamide (10-H-Halo, CA2)

10-H-Halo = **CA2**

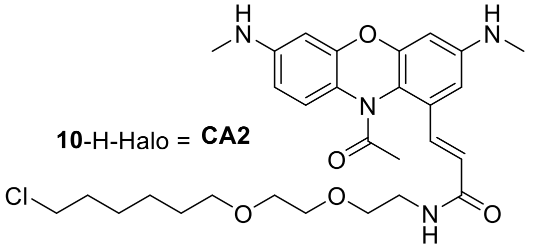

$C_{29}H_{39}ClN_4O_5$
M = 558.26

Compound **10-**H-H (23 mg, 0.07 mmol) was dissolved in dry DMA (2 ml). TSTU (33 mg, 0.11 mmol, 1.6 eq) and DIPEA (125 µl, 0.7 mmol, 10 eq) were added at r. t., and the reaction mixture was stirred for 10 min. $Cl(CH_2)_6O(CH_2)_2O(CH_2)_2NH_2$*HCl (28 mg, 0.1 mmol, 1.5 eq) was added, and the reaction mixture was stirred for 16 h. The reaction completion was monitored by LCMS (method B); starting compound **10**-H-H t_R 1.8 min, λ_{abs} 262, 372 nm, ESI-MS 354 $[M+H]^+$; product **10**-H-Halo = **CA2** t_R 3.0 min, λ_{abs} 260, 371 nm, ESI-MS 559 $[M+H]^+$. The reaction mixture was conc. in vacuum, and the residue separated on an Interchim puriFlash™ device with a 250 × 21.2 mm column (Knauer Eurosphere II 100-5 C18A, H_2O (0.1% v/v TFA) - MeCN (0.1% v/v TFA), gradient of MeCN 5 – 50% in 20 min). Compound **10**-H-Halo = **CA2** (8 mg) was isolated as greenish powder in 20% yield. ^{1}H NMR (400 MHz, acetonitrile-d_3) δ 7.45 (d, J = 15.7 Hz, 1H), 7.29 (d, J = 8.5 Hz, 1H), 6.74 (br. t. 1H, NH), 6.68 (d, J = 2.5 Hz, 1H), 6.53 (d, J = 15.7 Hz, 1H), 6.48 – 6.44 (m, 2H), 6.43 (d, J = 2.5 Hz, 1H), 3.60 – 3.49 (m, 8H), 3.46 – 3.38 (m, 4H), 2.79 (s, 3H), 2.77 (s, 3H), 2.04 (s, 3H), 1.70 (dq, J =

7.8 and 6.7 Hz, 2H), 1.52 (dq, J = 7.8 and 6.7 Hz, 2H), 1.42–1.28 (m, 4H). ESI-HRMS: found 559.2689 $[M]^+$, calculated 559.2682 for $C_{29}H_{39}N_4O_5Cl$, $[M+H]^+$.

(*E*)-3-(10-Acetyl-3,7-bis(dimethylamino)-10*H*-phenoxazin-1-yl)-*N*-(2-(2-((6-chlorohexyl)oxy)ethoxy)ethyl) acrylamide (10-Me-Halo = CA4)

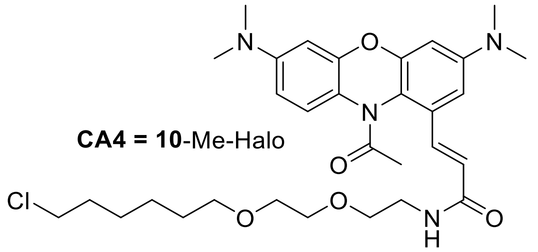

$C_{31}H_{43}ClN_4O_5$
M = 586.29

Compound **10**-Me-H (40 mg, 0.1 mmol) was dissolved in dry DMF (2 ml). TSTU (48 mg, 0.16 mmol, 1.6 eq) and DIPEA (350 µl, 2 mmol, 20 eq) were added at r. t., and the reaction mixture stirred for 2 min. $Cl(CH_2)_6O(CH_2)_2O(CH_2)_2NH_2$*HCl (33 mg, 0.12 mmol, 1. 5 eq) was added, and the reaction mixture stirred for 2 h. Then, additional amount (10 mg) of $Cl(CH_2)_6O(CH_2)_2O(CH_2)_2NH_2$*HCl was added. The reaction course was monitored by LCMS (method B); starting compound **10**-Me-H t_R 2.8 min, λ_{abs} 278, 384 nm, ESI-MS 438 $[M+H]^+$; product **10**-Me-Halo = **CA4** t_R 3.7 min, λ_{abs} 279, 374 nm, ESI-MS 587 $[M+H]^+$. The reaction mixture was conc. in vacuum, and the residue separated on an Interchim puriFlash™ device equipped with a 250 × 21.2 mm column (Knauer Eurosphere II 100-5 C18A, H_2O (0.1% v/v TFA) - MeCN (0.1% v/v TFA), gradient of MeCN 5 – 50% in 20 min). Compound **CA4** (10 mg) was isolated as greenish powder in 17% yield. ^1H NMR (400 MHz, acetonitrile-d_3) δ 7.57 (d, J = 9.5 Hz, 1H), 7.50 (d, J = 15.8 Hz, 1H), 7.11 (d, J = 2.7 Hz, 1H), 7.04 (dd, J = 6.1, 2.8 Hz, 2H), 6.98 (t, J = 2.7 Hz, NH, 1H), 6.87 (d, J = 2.6 Hz, 1H), 6.66 (d, J = 15.7 Hz, 1H), 3.62 – 3.49 (m, 8H), 3.54 (m, 2H), 3.42 (t, J = 7.0, 6.6 Hz, 2H), 3.08 (s, 6H), 3.05 (s, 6H), 2.07 (s, 3H), 1.69 (dq, J = 8.1, 6.7 Hz, 2H), 1.52 (dq, J = 7.8, 6.7 Hz, 2H), 1.44 – 1.28 (m, 4H). ESI-HRMS: found 587.2992 $[M+H]^+$, calculated 587.2995 for $C_{31}H_{43}N_4O_5Cl$, $[M+H]^+$.

Copies of LC-MS traces and NMR spectra

Compound 1-Me: LC-MS trace, ^{1}H-NMR spectrum (CD_3CN).

Probe : es986

Lösungsmittel : MeCN/H2O **Aufgabemenge:** 2.0 µl
Säule: Phenomenex Kinetex C18 1.7 µm **Länge:** 50 mm **iO :** 2.1 mm
Fluß (ml / Min) : 0.5 **Temperatur :** 25.0
Detektor: DAD-3000 **Pumpe:** HPG-3200SD **Sampler:** WPS-3000
Laufmittel: **A = Acetonitril 0.1% FA** **B = Wasser 0.1% FA**
Gradient: A 20.0 % B 80.0 % ----> A 100.0 % B 0.0 % T = 4 Min.

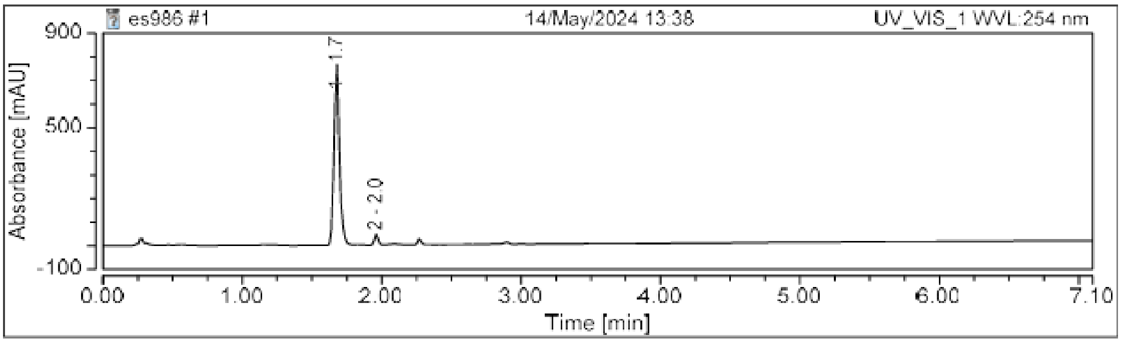

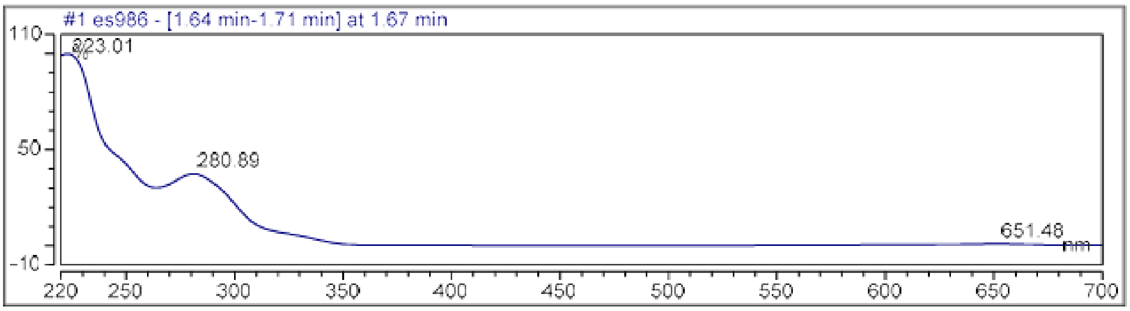

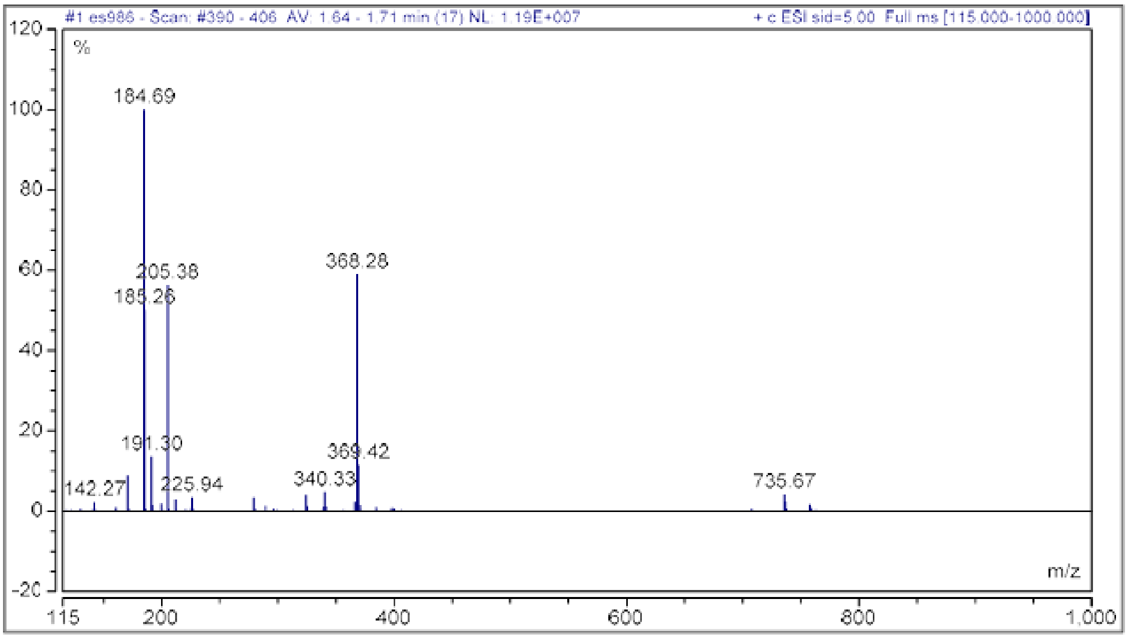

^{1}H NMR (400 MHz, Acetonitrile-d_3) δ 7.24 (d, J = 8.9 Hz, 2H), 6.41 (dd, J = 8.9, 2.9 Hz, 2H), 6.37 (d, J = 2.8 Hz, 2H), 3.33 (q, J = 7.0 Hz, 8H), 2.17 (s, 3H), 1.11 (t, J = 7.0 Hz, 12H).

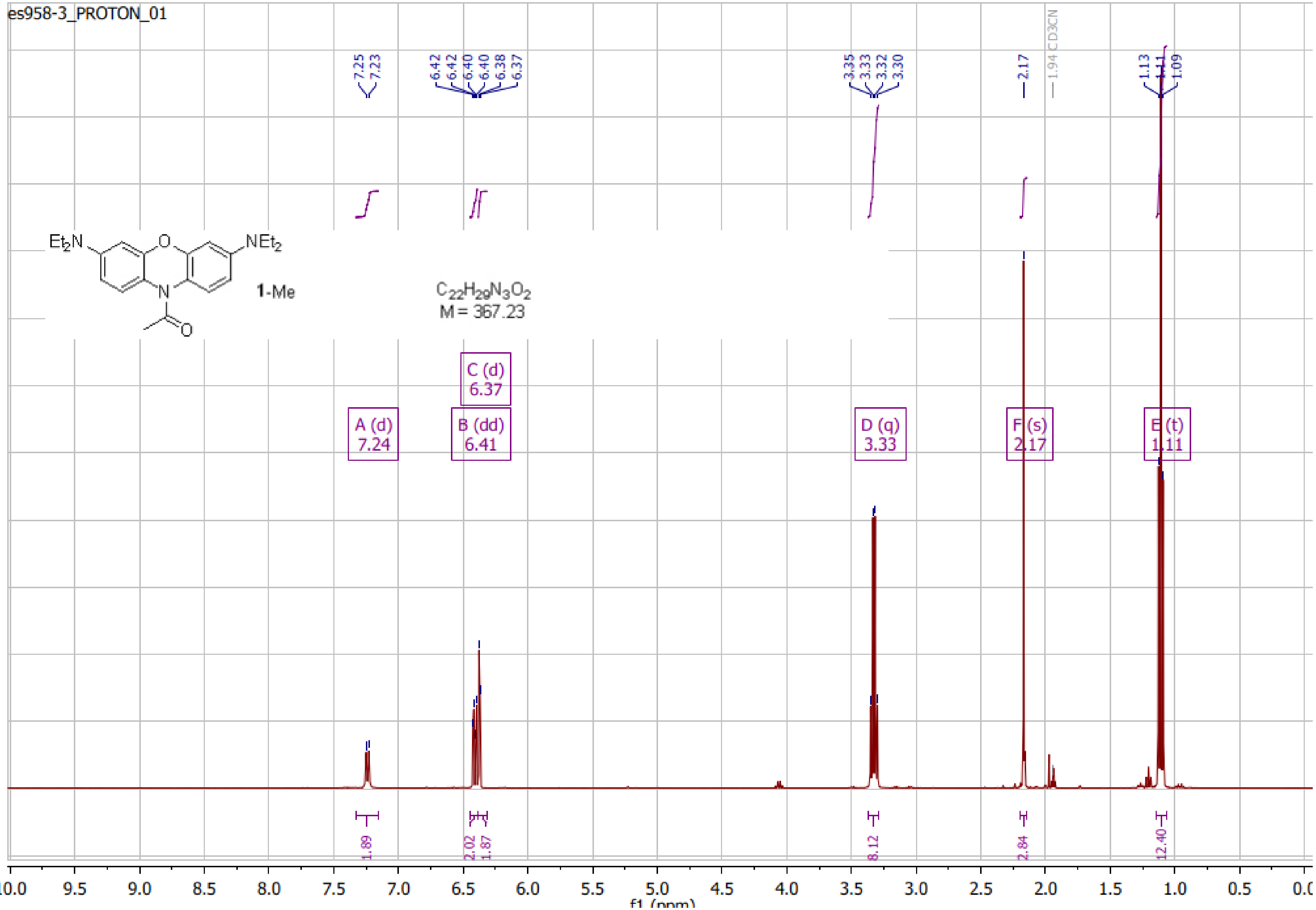

Compound **2**-*t*Bu. ^{1}H NMR (400 MHz, Acetonitrile-d_3) δ 7.58 (d, J = 16.0 Hz, 1H), 7.26 (d, J = 8.8 Hz, 1H), 6.69 (d, J = 2.8 Hz, 1H), 6.50 (d, J = 2.8 Hz, 1H), 6.47 – 6.36 (m, 3H), 3.36 (dq, J = 12.2, 7.0 Hz, 9H), 2.07 (s, 3H), 1.51 (s, 9H), 1.13 (td, J = 7.0, 4.3 Hz, 14H).

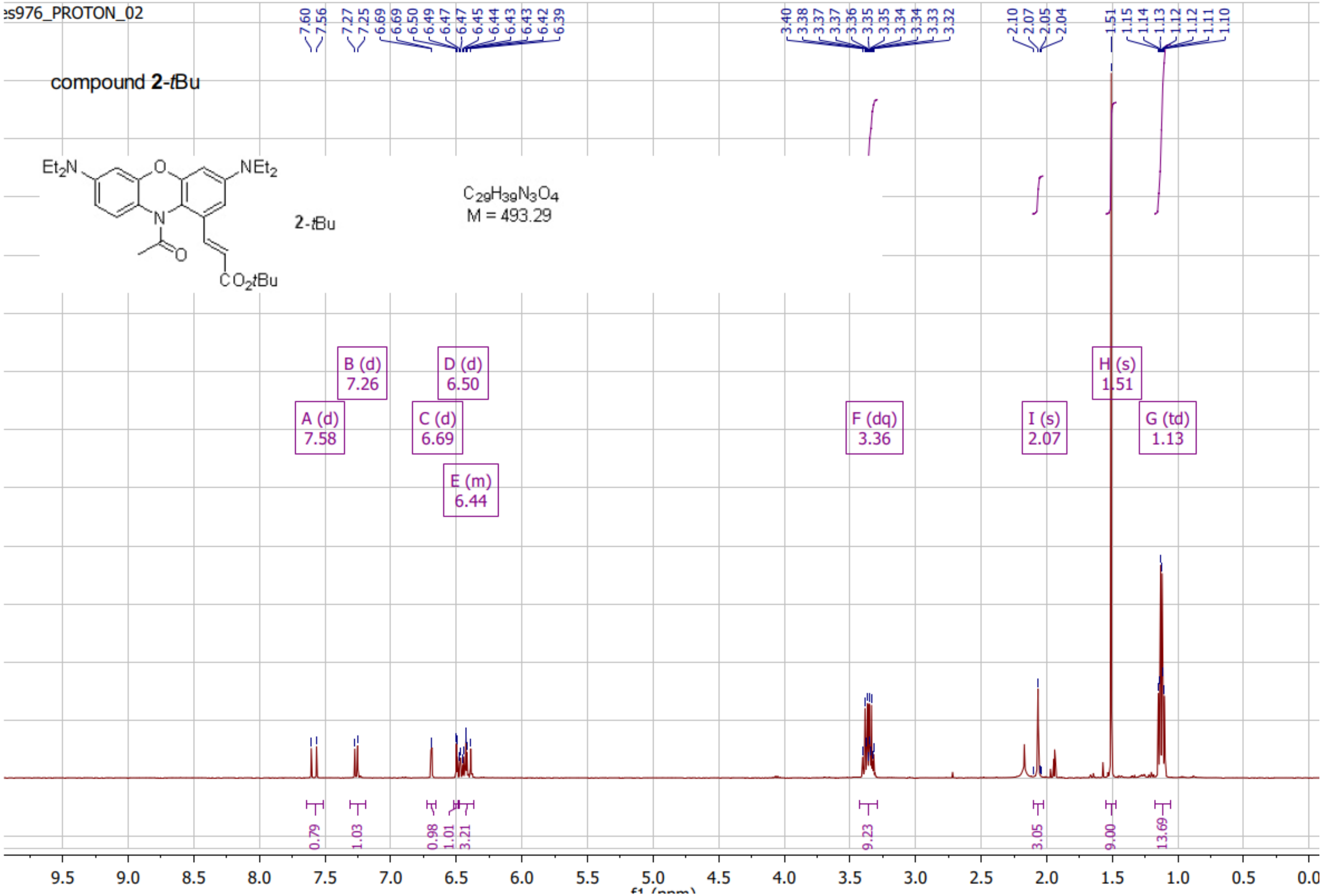

Compound 2-*t*Bu: ^{1}H-and ^{13}C-NMR spectra (CD_3CN)

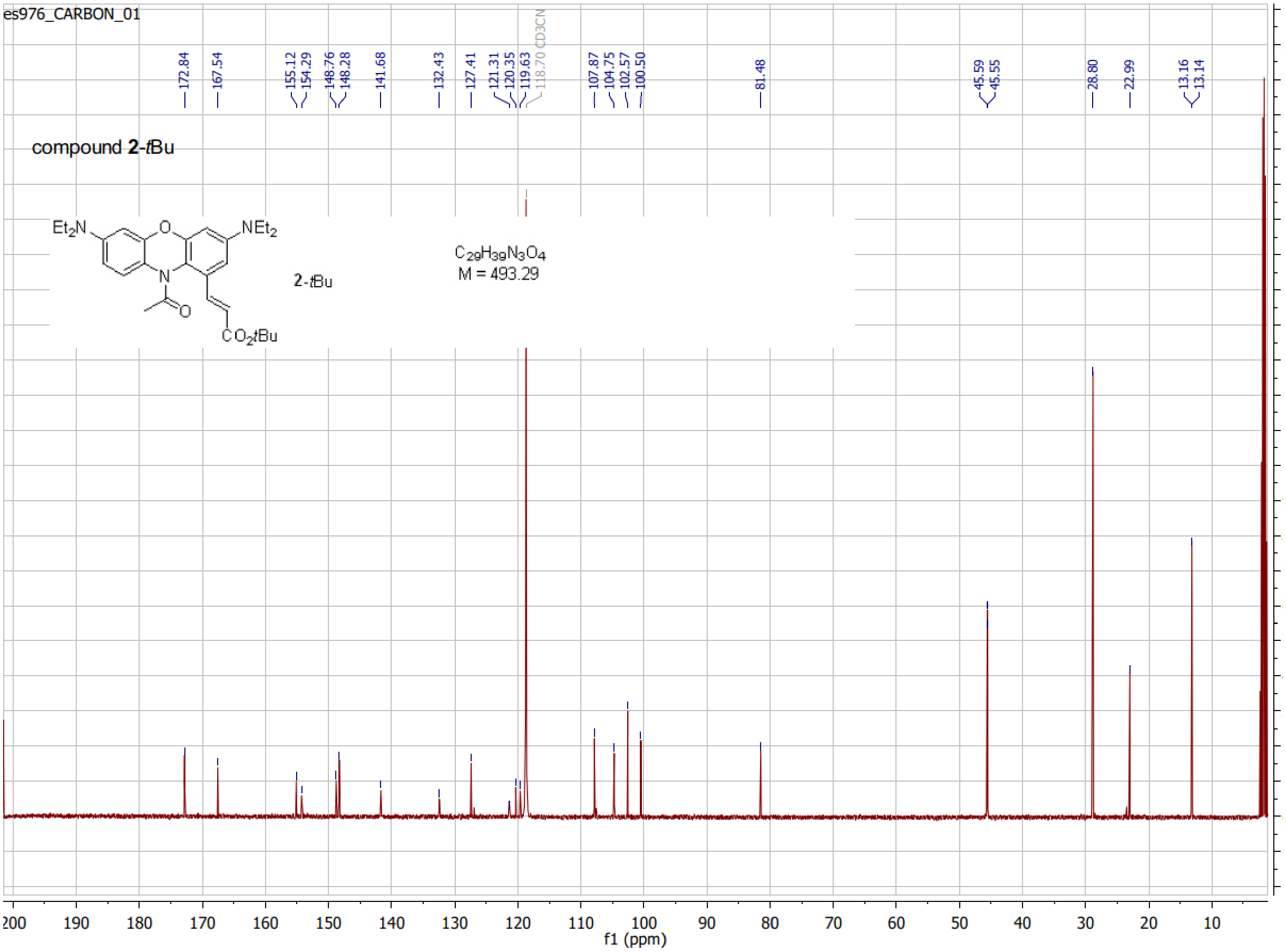

es976_CARBON_01
172.84
167.54
155.12
154.29
148.76
148.28
141.68
132.43
127.41
121.31
120.35
119.63
118.70 CD3CN
107.87
104.75
102.57
100.50
81.48
45.59
45.55
28.80
22.99
13.16
13.14
compound 2-tBu
2-tBu
$C_{29}H_{39}N_3O_4$
M = 493.29
f1 (ppm)

Compound 2-Bn: ^{1}H-NMR spectrum (CD_3CN).

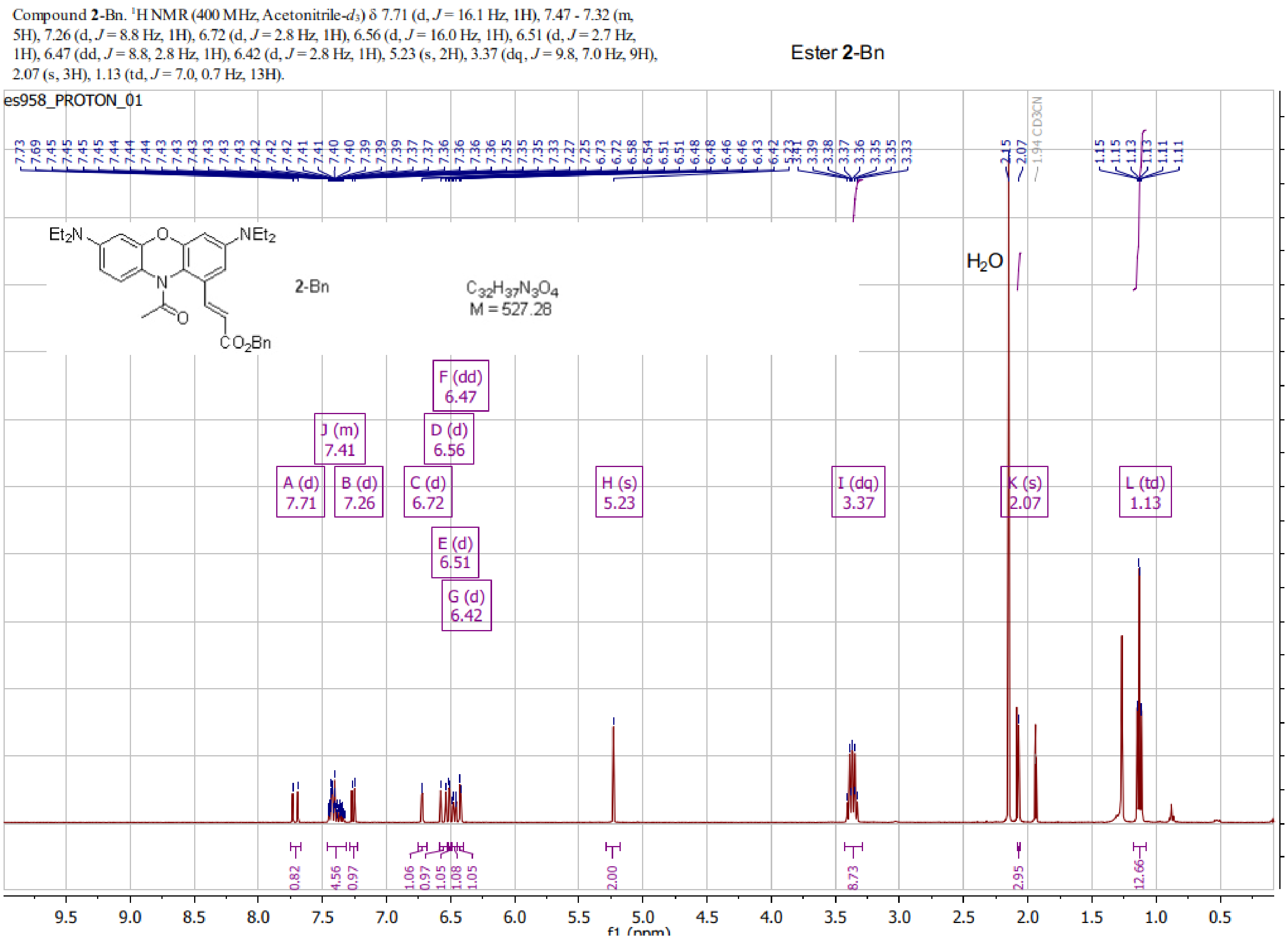

Compound **2**-H: LC-MS trace, ^{1}H- and ^{13}C-NMR spectra (CD_3CN):

Probe : es979

**Lösungsmittel :**	MeCN/H2O	**Aufgabemenge:**	3.0 µl		
**Säule:**	Phenomenex Kinetex C18 2.6 µm	**Länge:**	75 mm	**iO :**	3.0 mm
**Fluß (ml / Min) :**	0.5	**Temperatur :**	25.0		
**Detektor:**	DAD-3000	**Pumpe:**	HPG-3200SD	**Sampler:**	WPS-3000
**Laufmittel:**	**A = Acetonitril 0.1% FA**			**B = Wasser 0.1% FA**	
**Gradient:**	A 20.0 % B 80.0 % ---->	A 100.0 % B 0.0 %	T = 10 Min.		

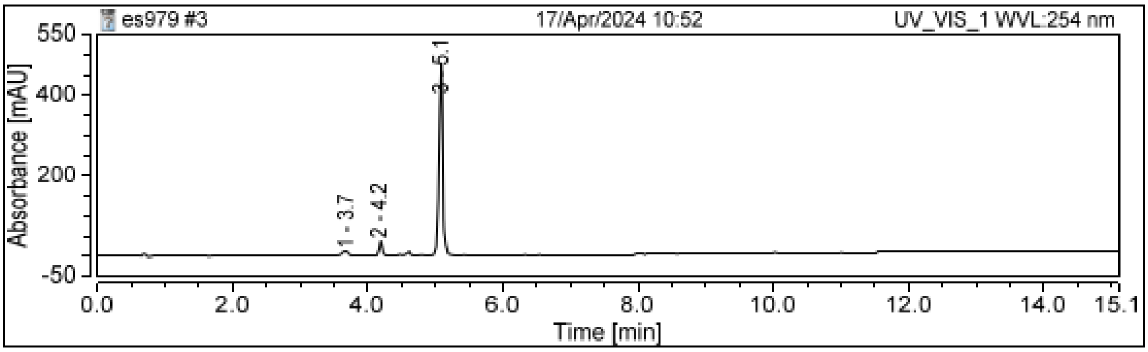

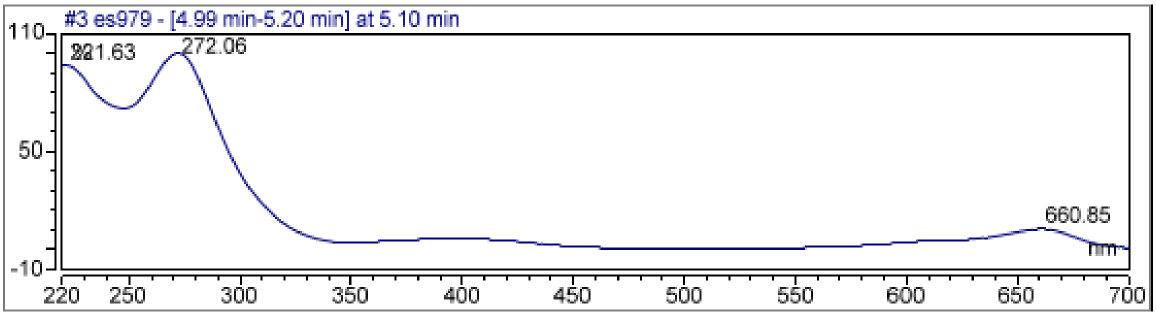

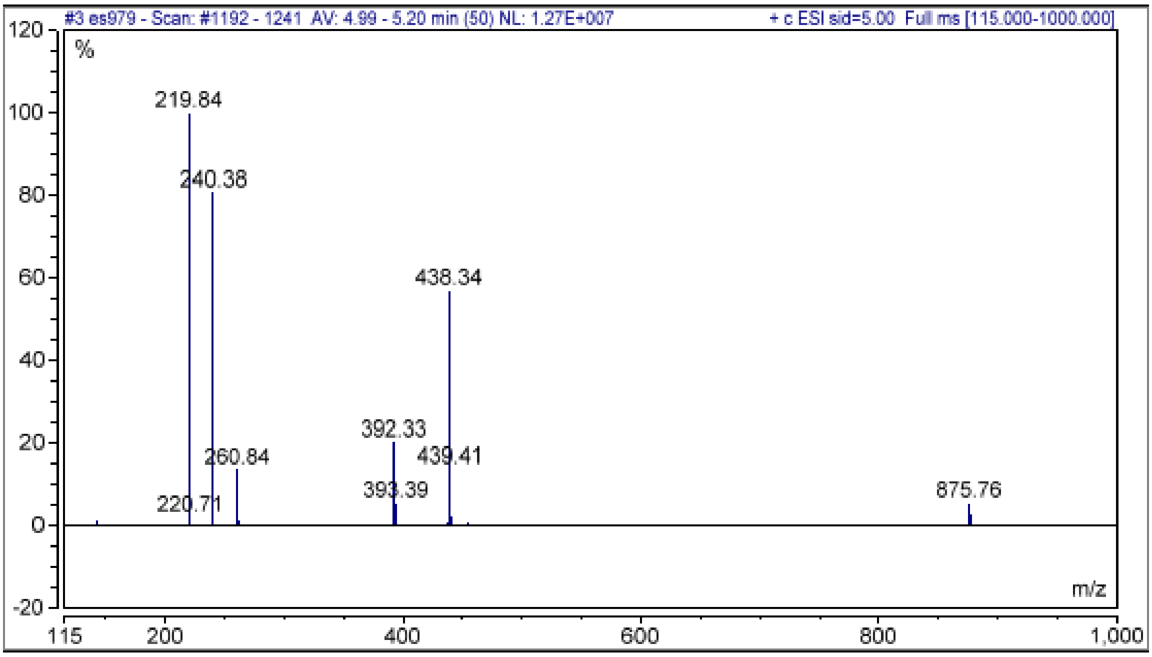

Compound **2**-H * **$(C_2H_5)_3N$**: ^{1}H NMR (400 MHz, Acetonitrile-d_3) δ 7.64 (d, J = 16.1 Hz, 1H), 7.28 (d, J = 8.8 Hz, 1H), 6.72 (d, J = 2.8 Hz, 1H), 6.54 – 6.49 (m, 2H), 6.49 – 6.39 (m, 2H), 3.37 (dq, J = 12.6, 7.1 Hz, 10H), 3.04 (q, J = 7.3 Hz, 3H, CH_2N in Et_3N), 2.07 (s, 4H), 1.28 (t, J = 7.3 Hz, 6H, CH_3 in Et_3N), 1.13 (td, J = 7.0, 4.3 Hz, 14H).

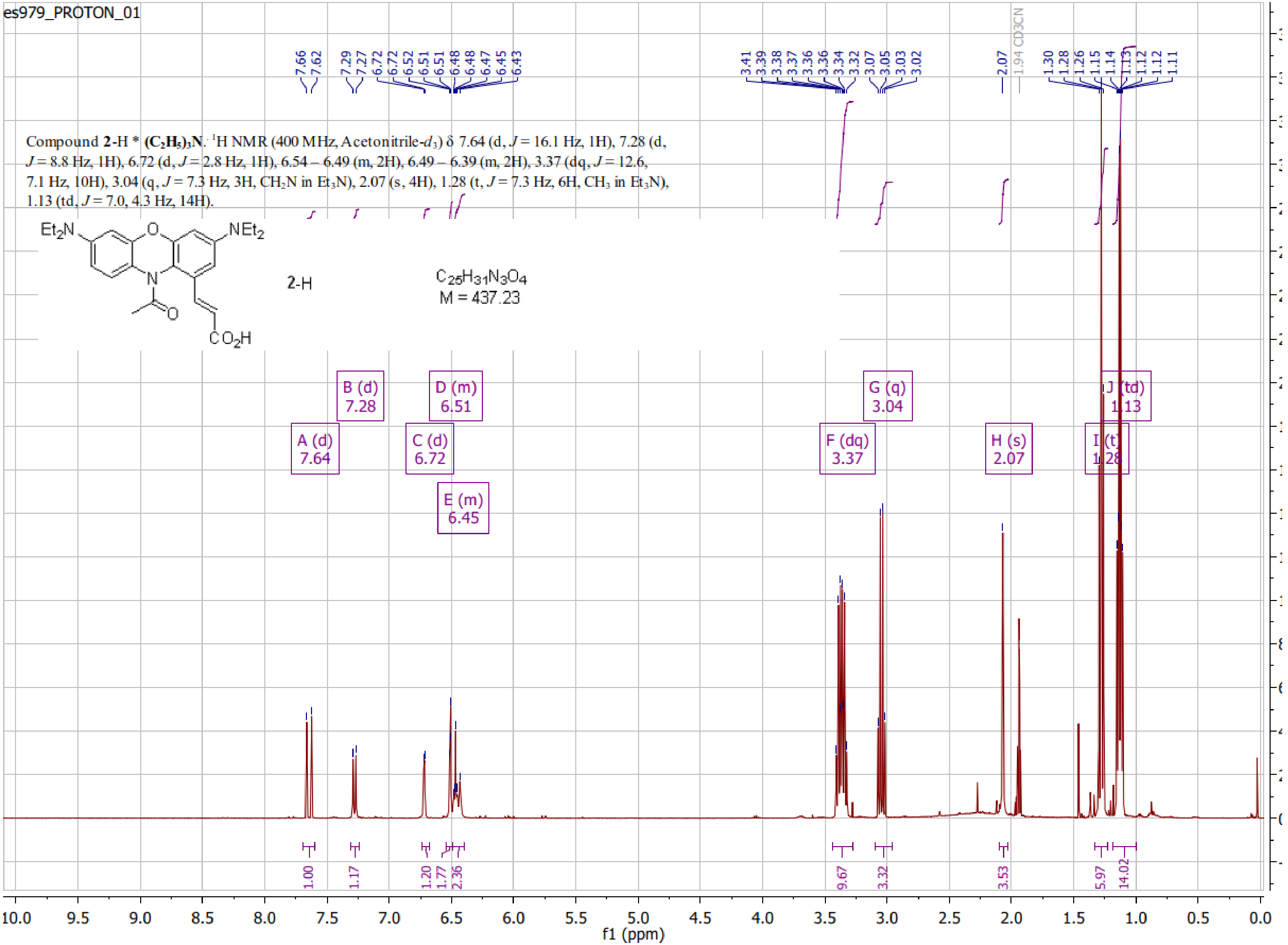

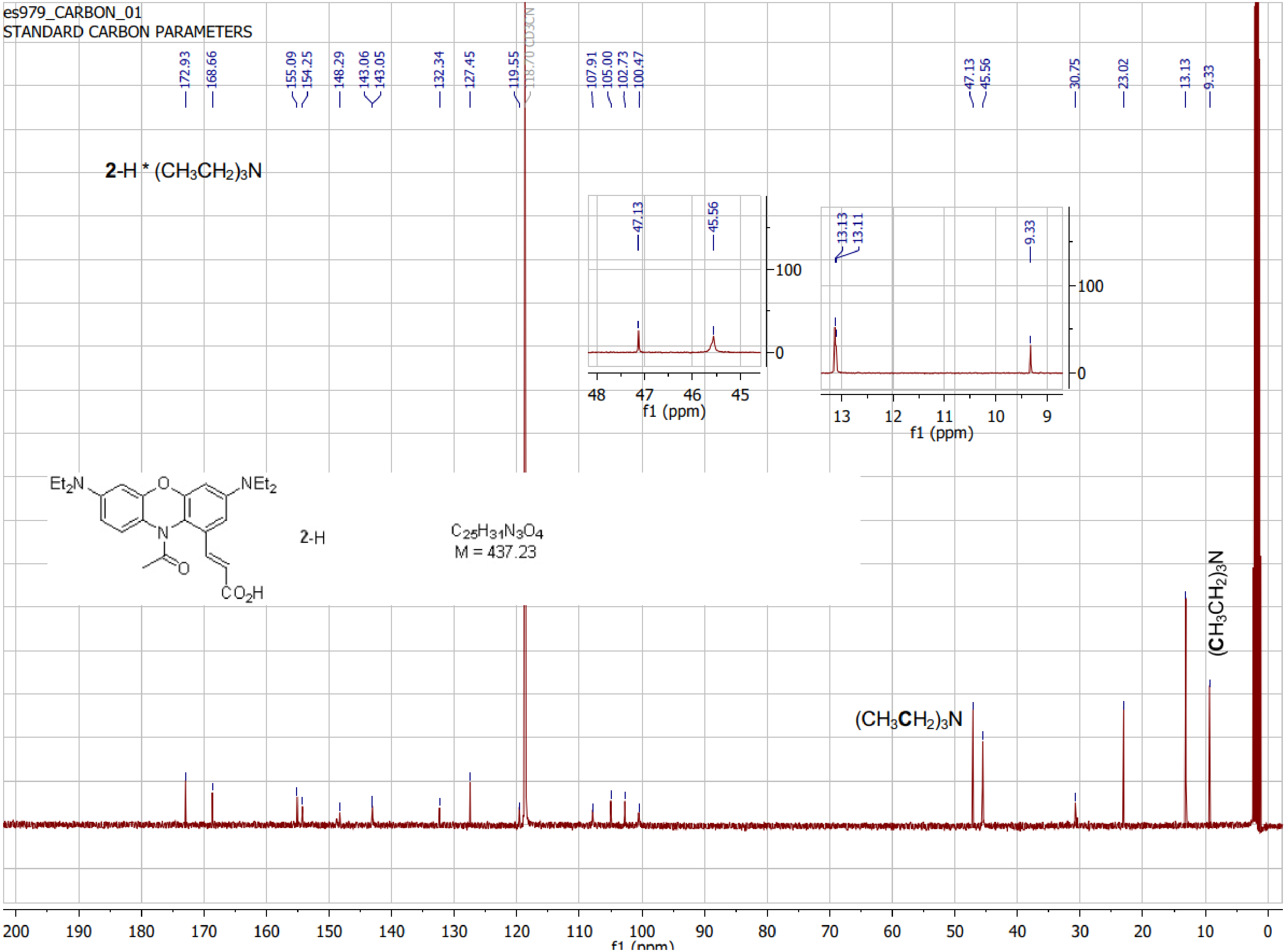

es979_CARBON_01
STANDARD CARBON PARAMETERS
172.93
168.66
155.09
154.25
148.29
143.06
143.05
132.34
127.45
119.55
118.70 CD3CN
107.91
105.00
102.73
100.47
47.13
45.56
30.75
23.02
13.13
9.33
2-H * (CH3CH2)3N
2-H
C25H31N3O4
M = 437.23
13.13
13.11
(CH3CH2)3N
(CH3CH2)3N
f1 (ppm)

Dye **A**, LC-MS trace and ^{1}H-NMR spectrum (CD_3CN):

Probe : es995-12 rein

**Lösungsmittel :**	MeCN/H2O	**Aufgabemenge:**	1.0 µl		
**Säule:**	Phenomenex Kinetex C18 1.7 µm	**Länge:**	50 mm	**iO :**	2.1 mm
**Fluß (ml / Min) :**	0.5	**Temperatur :**	25.0		
**Detektor:**	DAD-3000	**Pumpe:**	HPG-3200SD	**Sampler:**	WPS-3000
**Laufmittel:**	**A = Acetonitril 0.1% FA**			**B = Wasser 0.1% FA**	
**Gradient:**	A 20.0 % B 80.0 % ---->	A 100.0 % B 0.0 %	T = 4 Min.		

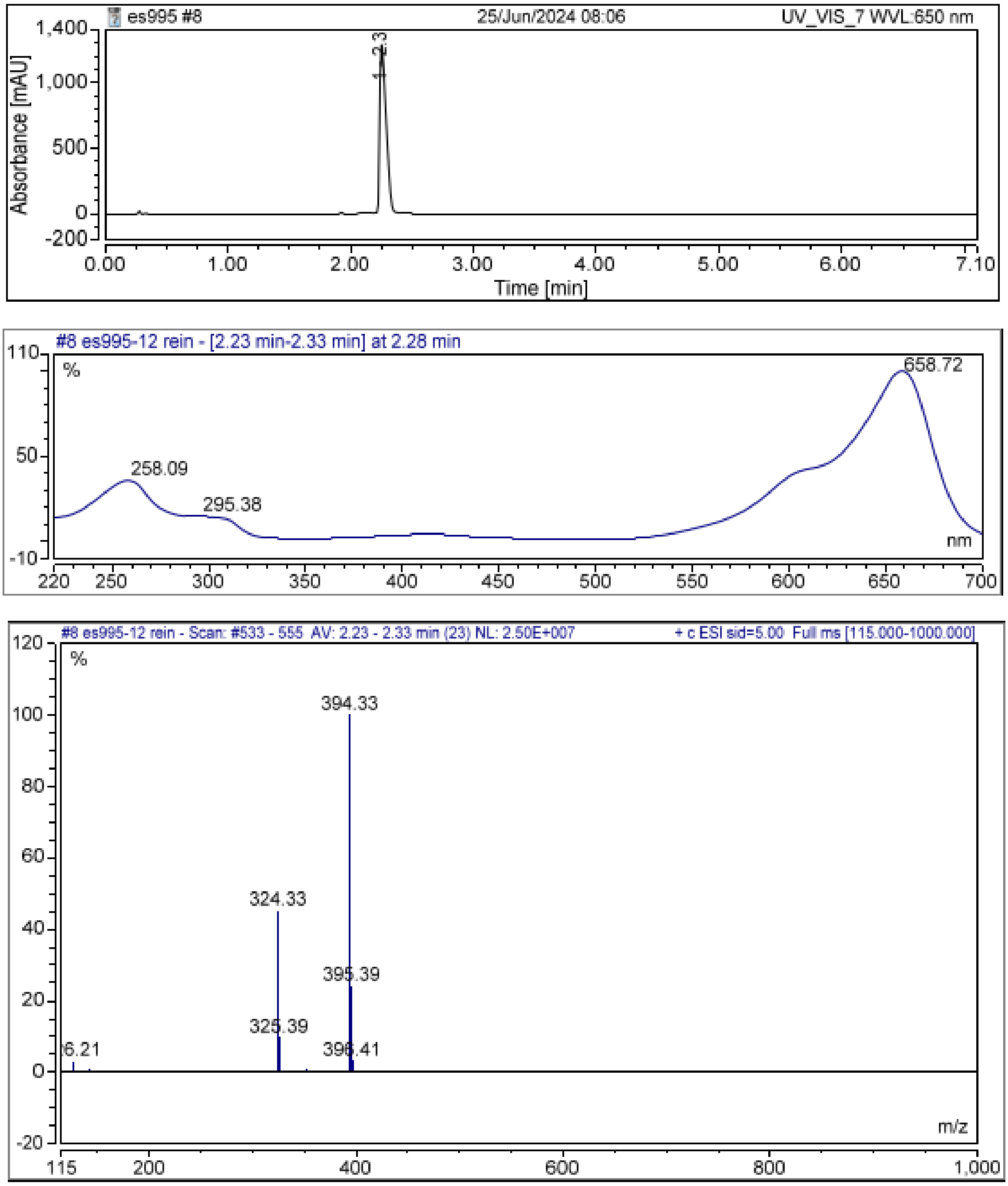

Dye **A**. ^{1}H NMR (400 MHz, Acetonitrile-d_3) δ 8.32 (dd, J = 16.2, 0.5 Hz, 1H), 7.79 (d, J = 9.7 Hz, 1H), 7.74 (d, J = 9.6 Hz, 0H), 7.46 (dd, J = 2.7, 0.6 Hz, 1H), 7.29 (ddd, J = 12.4, 9.7, 2.7 Hz, 1H), 7.01 (d, J = 16.2 Hz, 1H), 6.79 (d, J = 2.7 Hz, 2H), 3.72 (dt, J = 9.0, 6.6 Hz, 9H), 1.35 – 1.27 (m, 13H).

Dye **A**

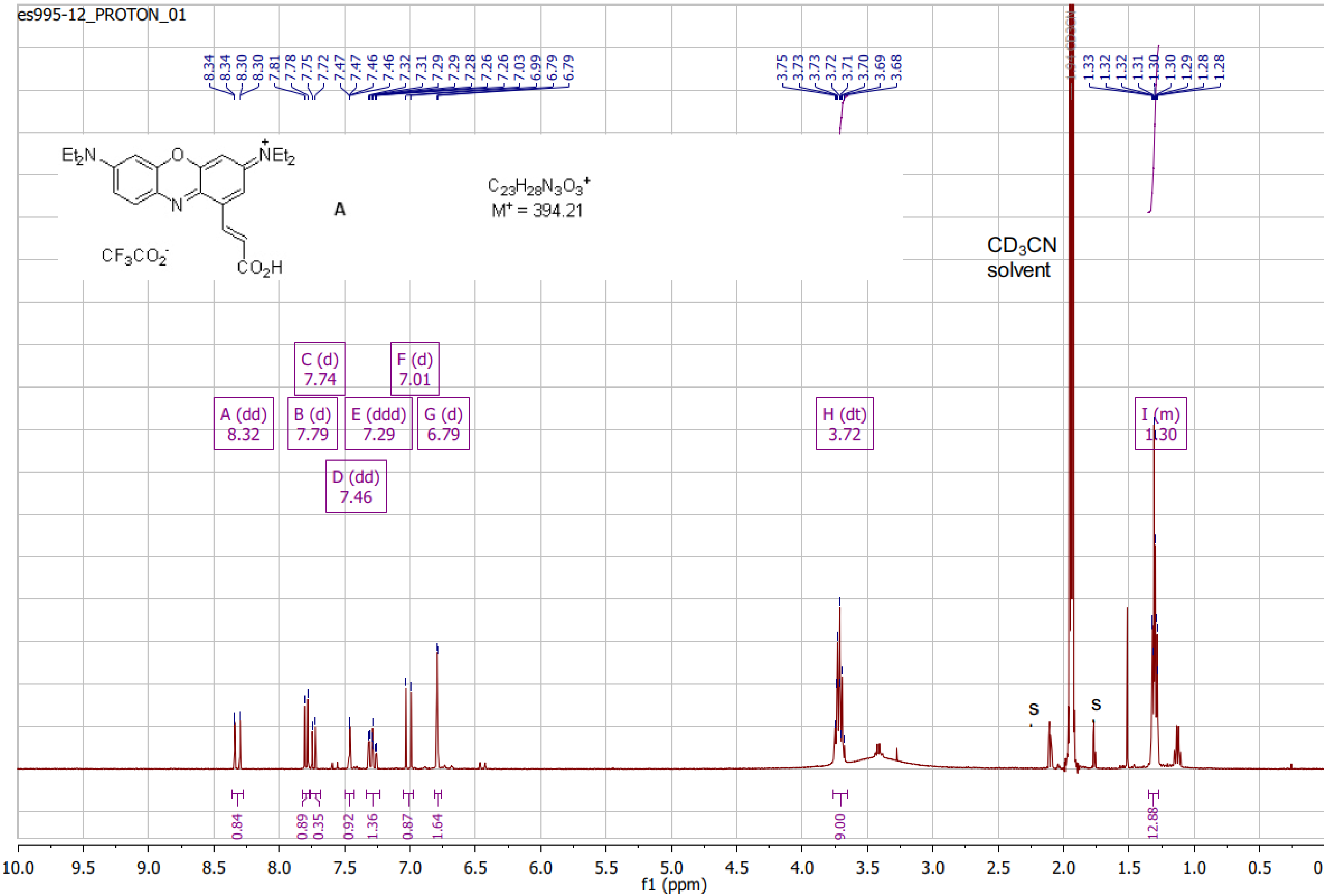

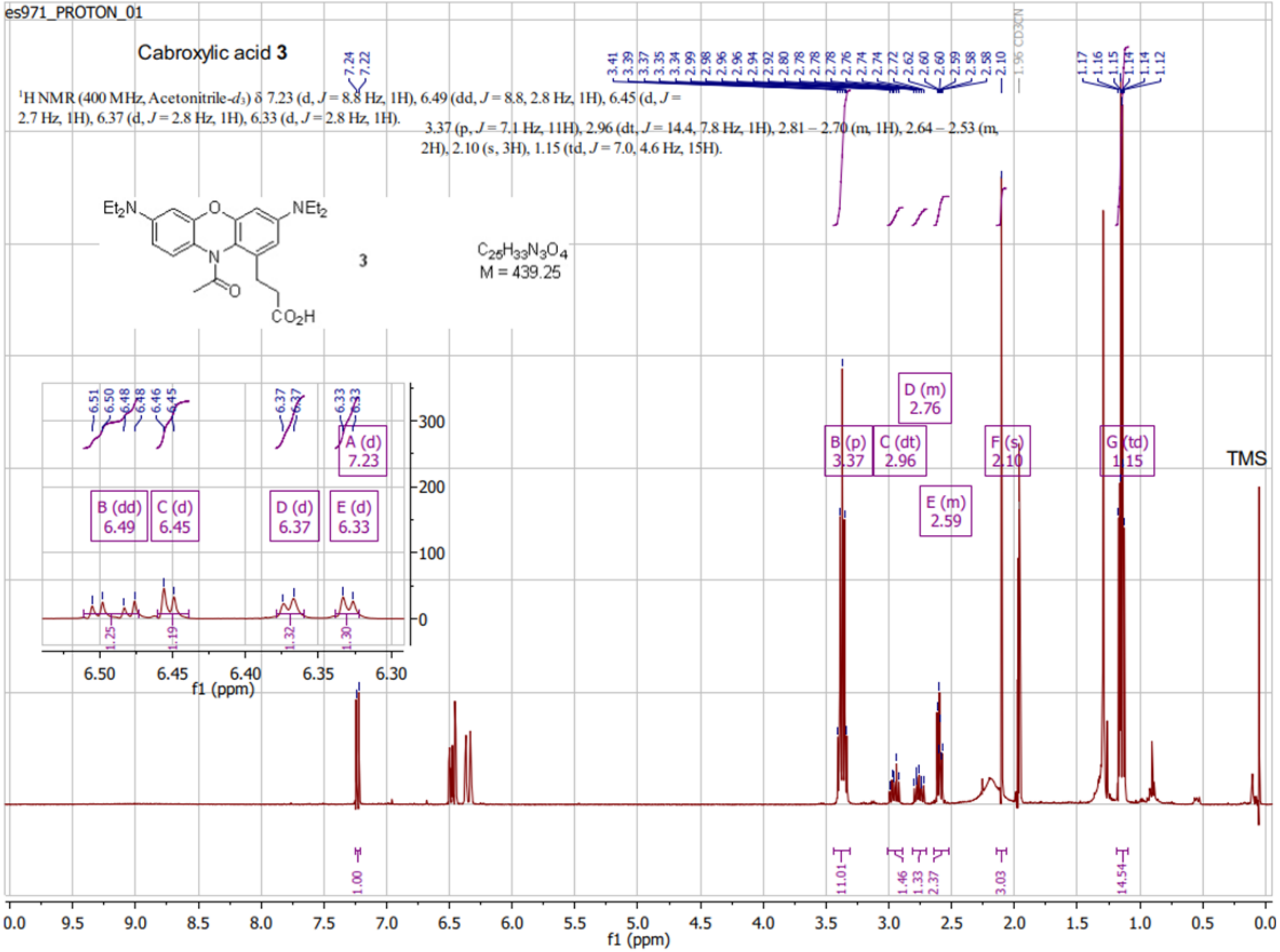

Carboxylic acid **3**: ^{1}H-NMR spectrum (CD_3CN) and LC-MS trace:

Probe : es971

Lösungsmittel : MeCN/H2O
Aufgabemenge: 3.0 µl
Säule: Phenomenex Kinetex C18 2.6 µm
Länge: 75 mm
iO : 3.0 mm
Fluß (ml / Min) : 0.5
Temperatur : 25.0
Detektor: DAD-3000
Pumpe: HPG-3200SD
Sampler: WPS-3000
Laufmittel: A = Acetonitril 0.1% FA B = Wasser 0.1% FA
Gradient: A 20.0 % B 80.0 % ----> A 100.0 % B 0.0 % T = 10 Min.

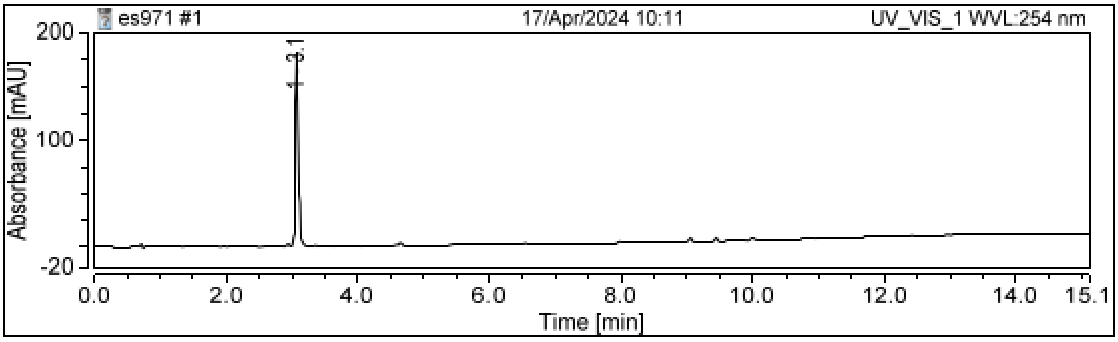

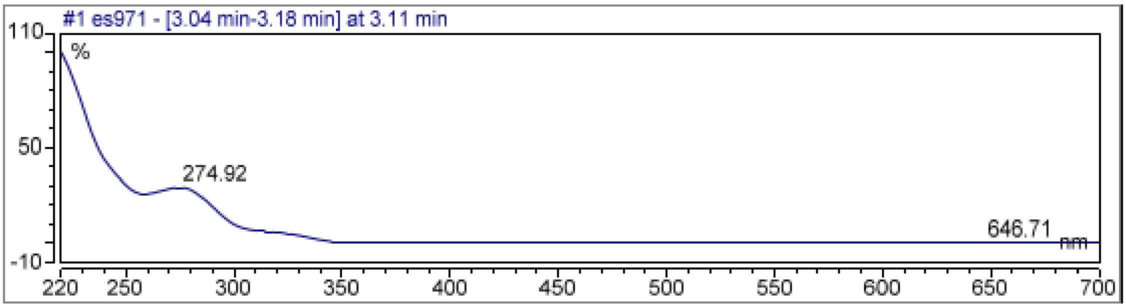

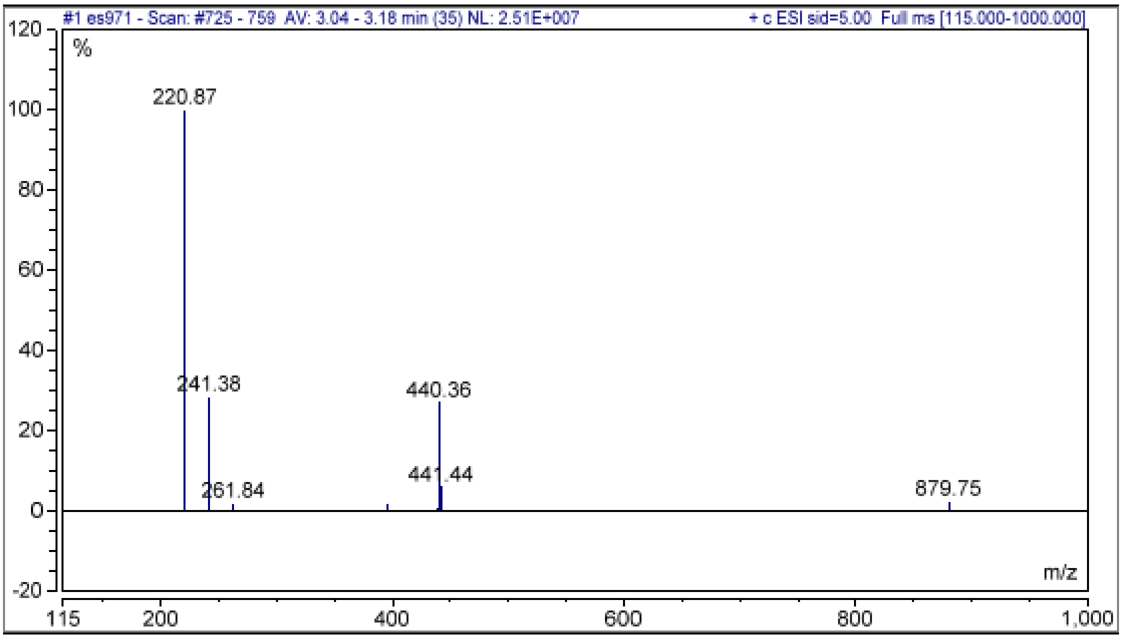

Compound **4**, ^{1}H- and ^{13}C-NMR spectra (acetone-d_6):

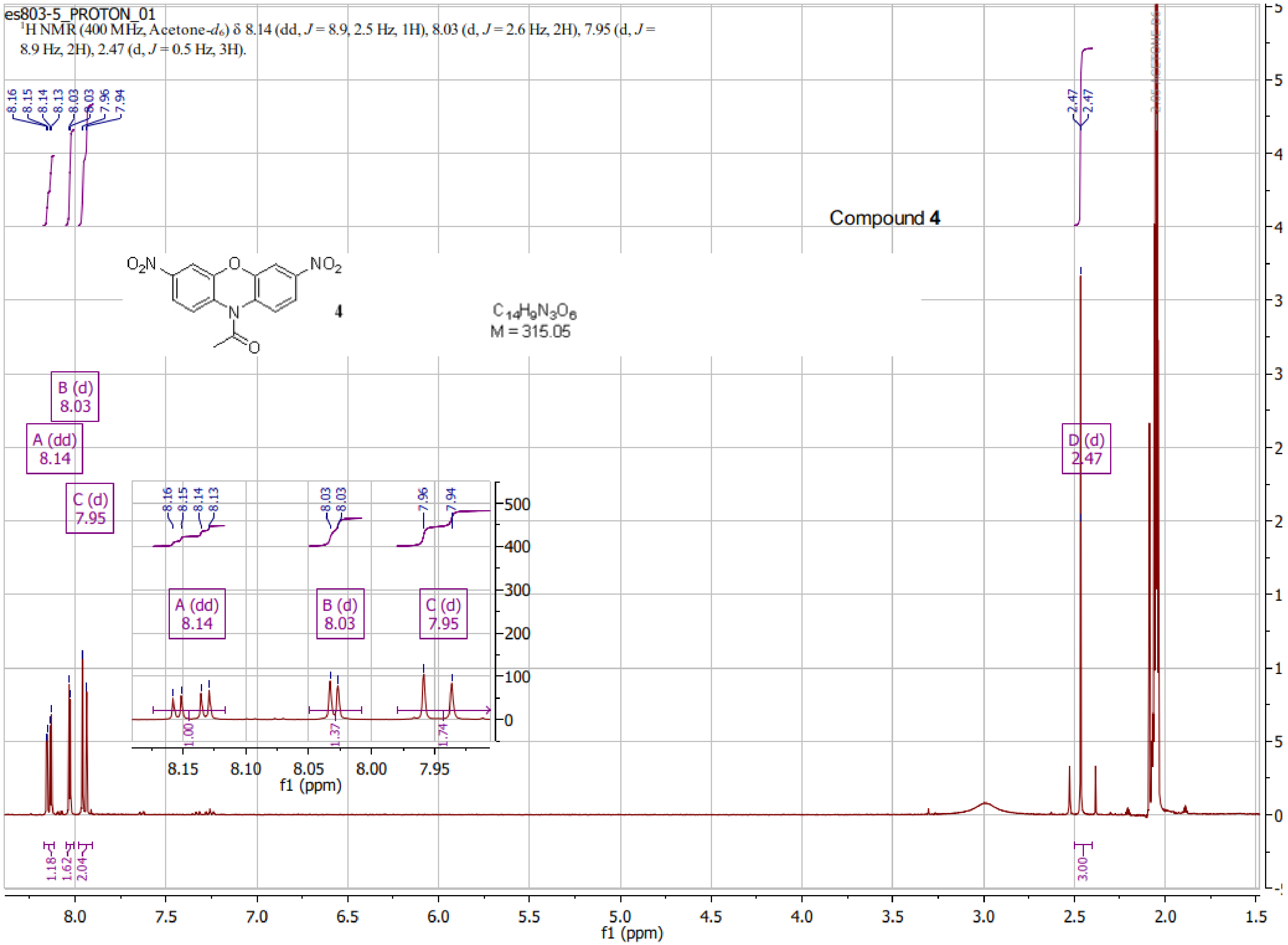

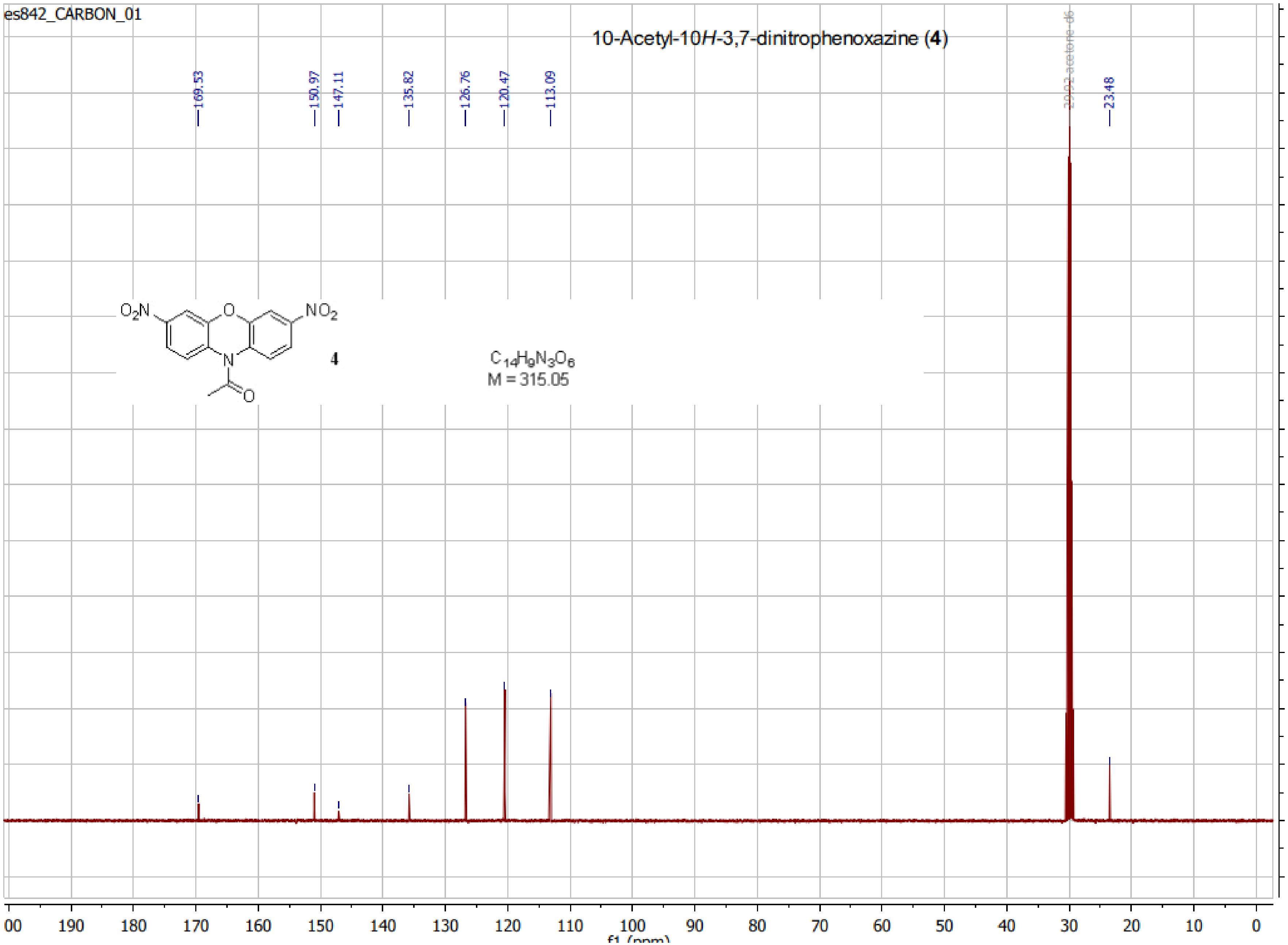

es842_CARBON_01
10-Acetyl-10H-3,7-dinitrophenoxazine (4)
169.53
150.97
147.11
135.82
126.76
120.47
113.09
29.92 acetone-d6
23.48
O2N
NO2
O
N
O
4
C14H9N3O6
M = 315.05
00
190
180
170
160
150
140
130
120
110
100
90
80
70
60
50
40
30
20
10
0
f1 (ppm)

Compound **5**, ^{1}H- and ^{13}C-NMR spectra (CD_3CN):

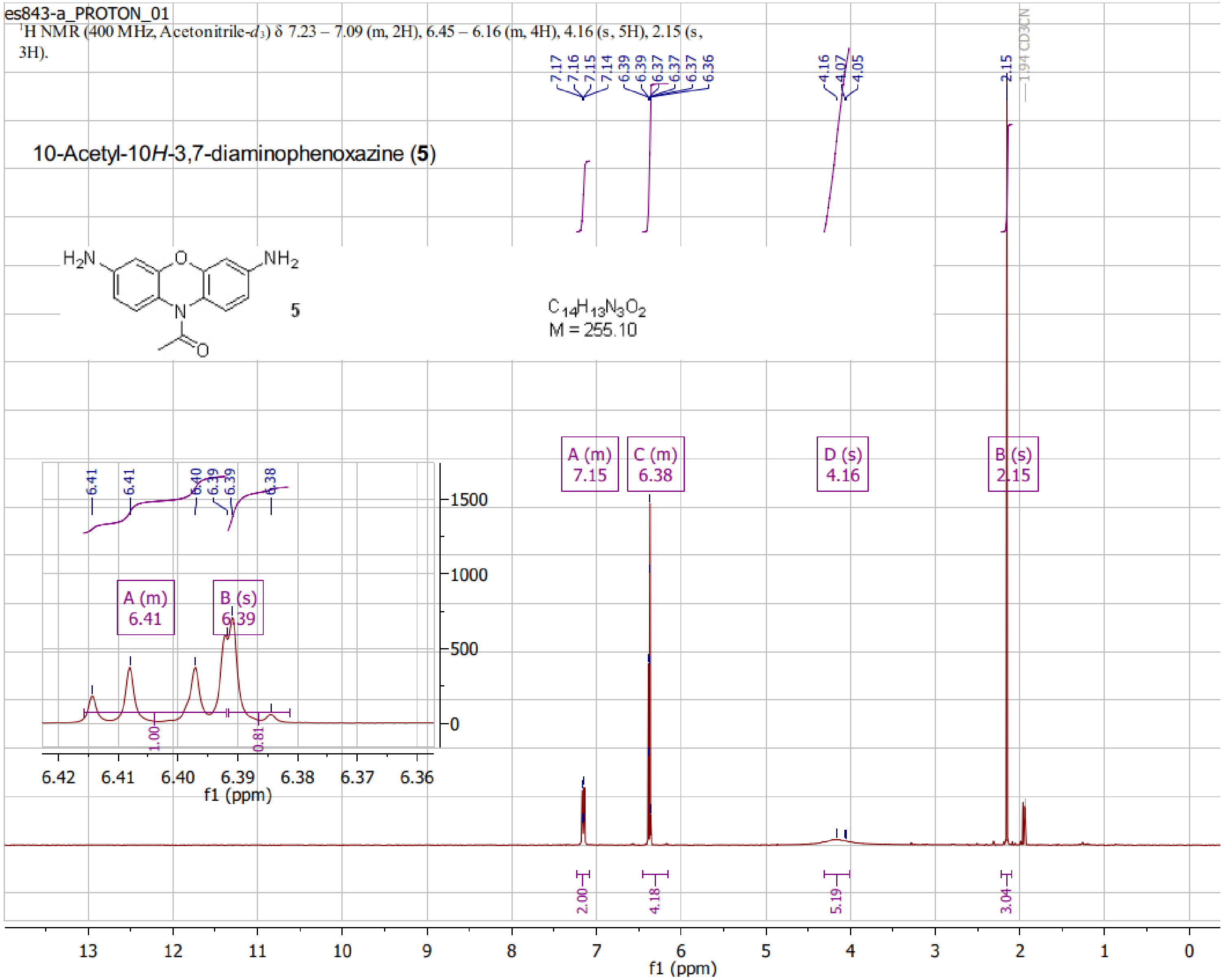

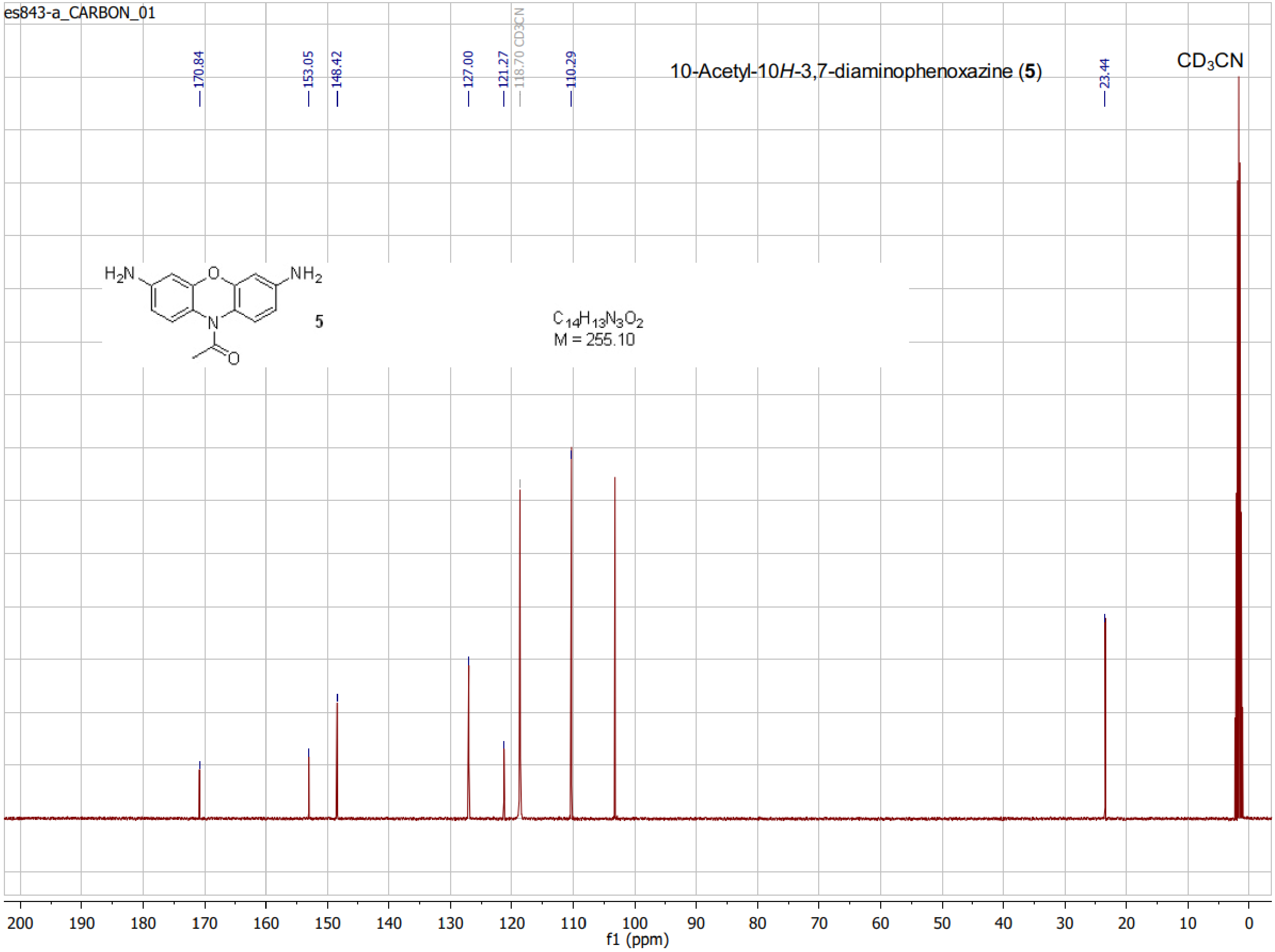

es843-a_CARBON_01
170.84
153.05
148.42
127.00
121.27
118.70 CD3CN
110.29
23.44
10-Acetyl-10H-3,7-diaminophenoxazine (5)
CD3CN
H2N
O
NH2
N
O
5
C14H13N3O2
M = 255.10
200 190 180 170 160 150 140 130 120 110 100 90 80 70 60 50 40 30 20 10 0
f1 (ppm)

Compound **6**-OBu^t. LC-MS trace, ^{1}H-, ^{13}C-NMR spectra (CD_3CN):

Probe : es961-2

Lösungsmittel : MeCN/H2O
Aufgabemenge: 3.0 µl
Säule: Phenomenex Kinetex C18 2.6 µm
Länge: 75 mm
iO : 3.0 mm
Fluß (ml / Min) : 0.5
Temperatur : 25.0
Detektor: DAD-3000
Pumpe: HPG-3200SD
Sampler: WPS-3000
Laufmittel: A = Acetonitril 0.1% FA B = Wasser 0.1% FA
Gradient: A 20.0 % B 80.0 % ---> A 100.0 % B 0.0 % T = 10 Min.

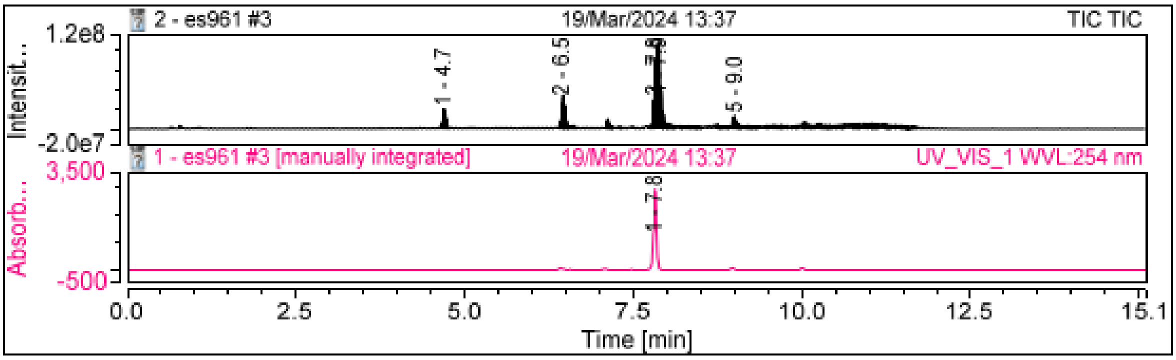

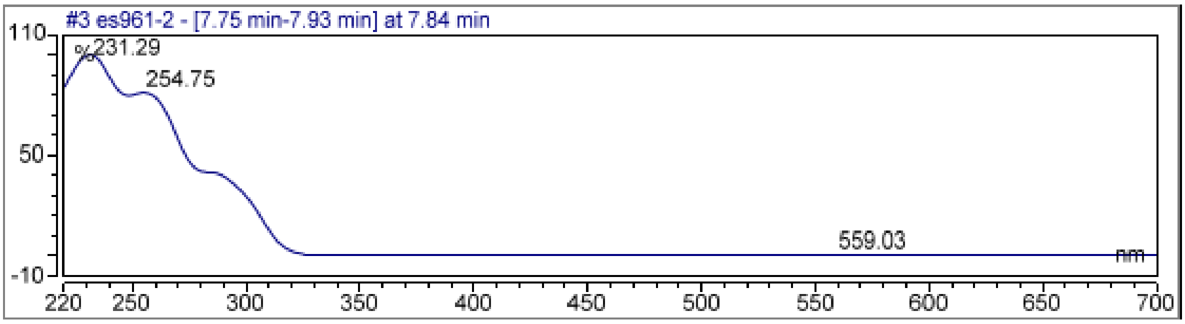

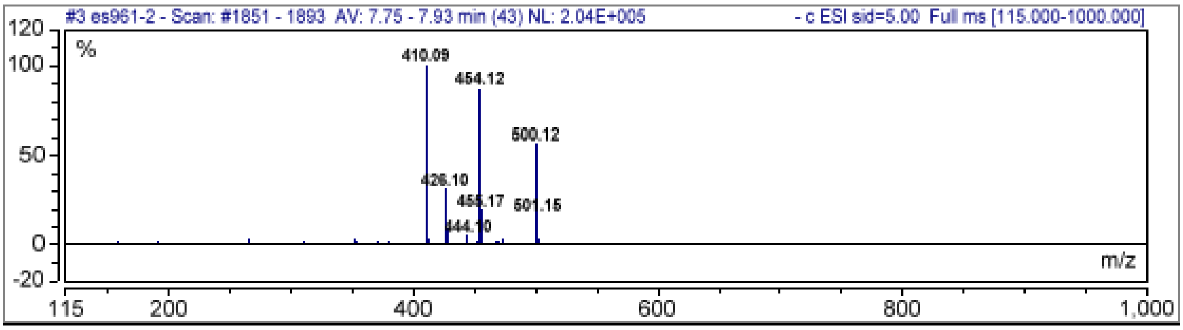

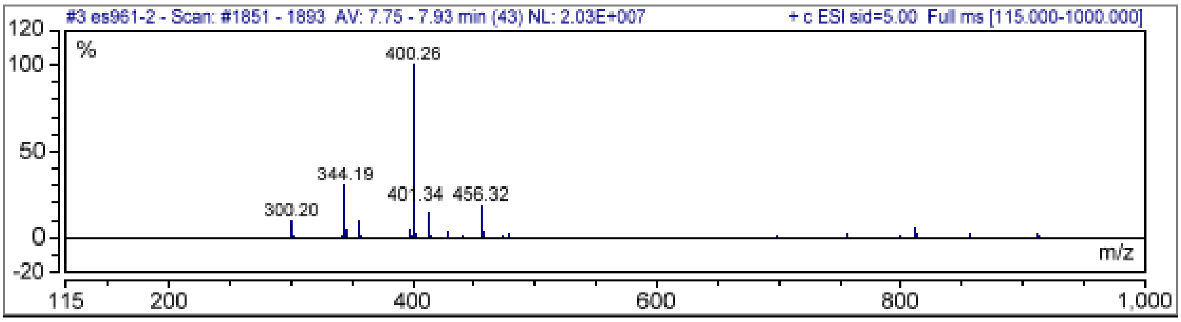

Di-*tert*-butyl 10-acetyl-10*H*-phenoxazine-3,7-diyl)dicarbamate (**6**-O*t*Bu)

^{1}H NMR (400 MHz, Acetone-d_6) δ 8.59 (s, 2H), 7.55 (d, J = 2.4 Hz, 2H), 7.48 (d, J = 8.8 Hz, 2H), 7.27 (dd, J = 8.8, 2.4 Hz, 2H), 2.28 (s, 3H), 1.50 (s, 18H).

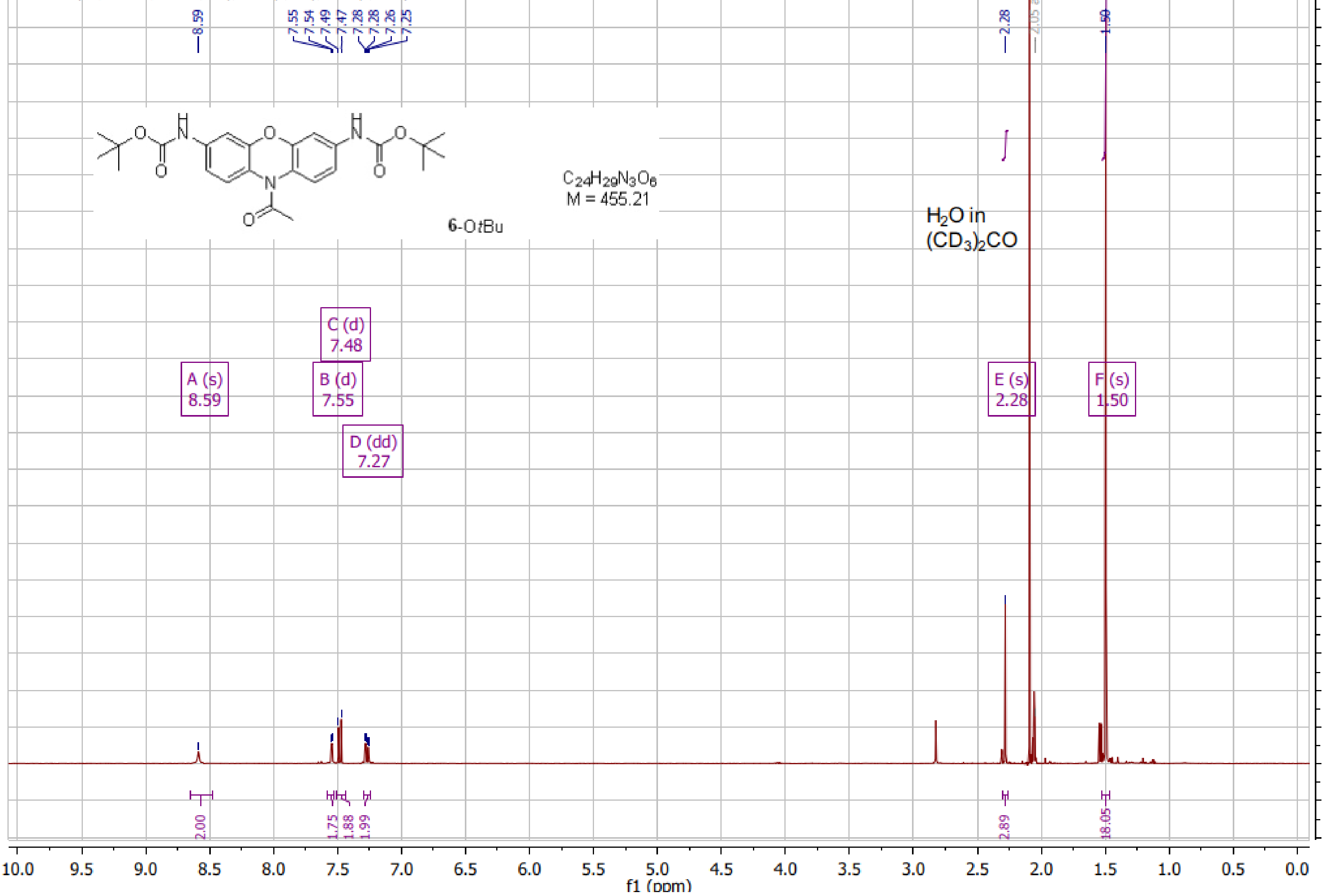

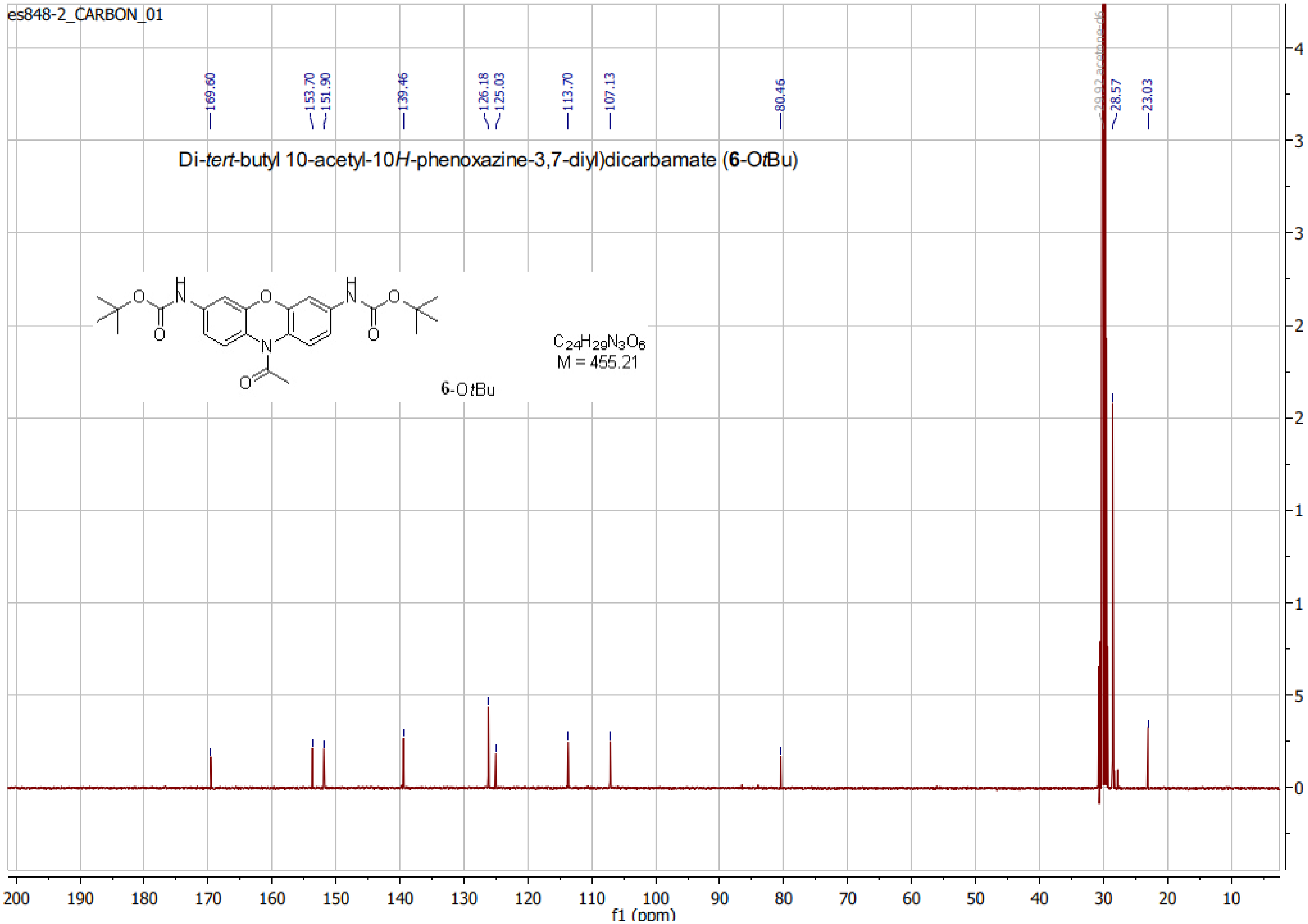

es848-2_CARBON_01
169.60
153.70
151.90
139.46
126.18
125.03
113.70
107.13
80.46
29.92 acetone-d6
28.57
23.03
Di-tert-butyl 10-acetyl-10H-phenoxazine-3,7-diyl)dicarbamate (6-OtBu)
C24H29N3O6
M = 455.21
6-OtBu
f1 (ppm)

Compound **6**-CF_3. LC-MS trace and ^{1}H-NMR spectrum (acetone-d_6):

Probe : es1014

Lösungsmittel : MeCN/H2O **Aufgabemenge:** 2.0 µl
Säule: Phenomenex Kinetex C18 1.7 µm **Länge:** 50 mm **iO :** 2.1 mm
Fluß (ml / Min) : 0.5 **Temperatur :** 25.0
Detektor: DAD-3000 **Pumpe:** HPG-3200SD **Sampler:** WPS-3000
Laufmittel: **A = Acetonitril 0.1% FA** **B = Wasser 0.1% FA**
Gradient: A 20.0 % B 80.0 % ----> A 100.0 % B 0.0 % T = 4 Min.

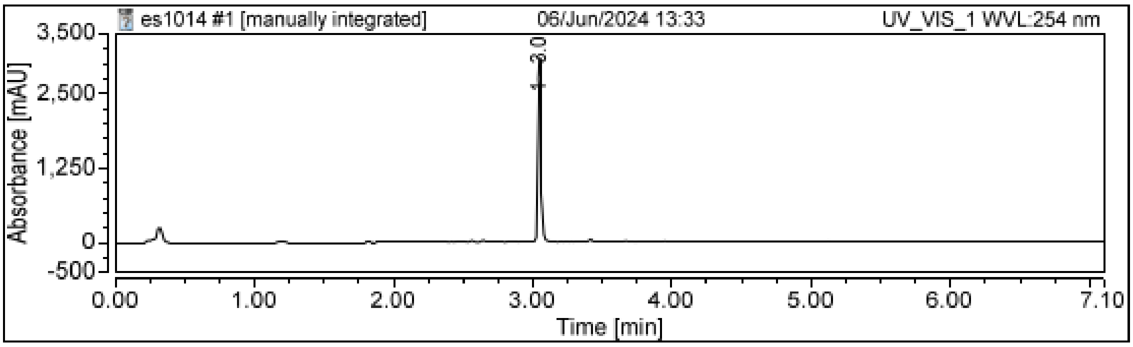

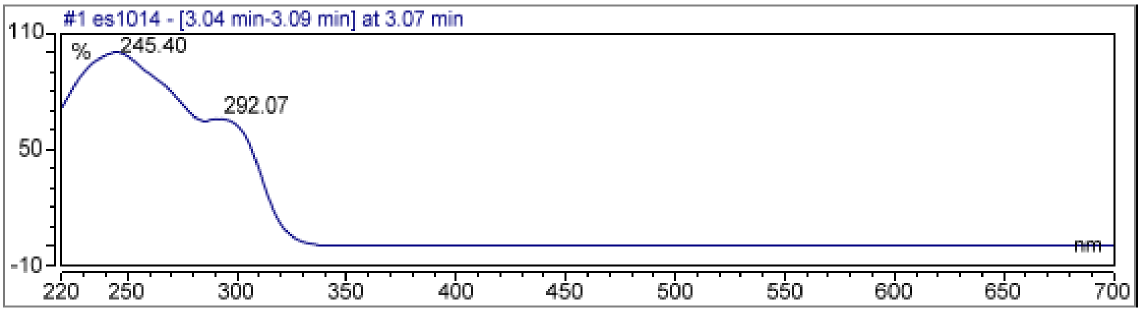

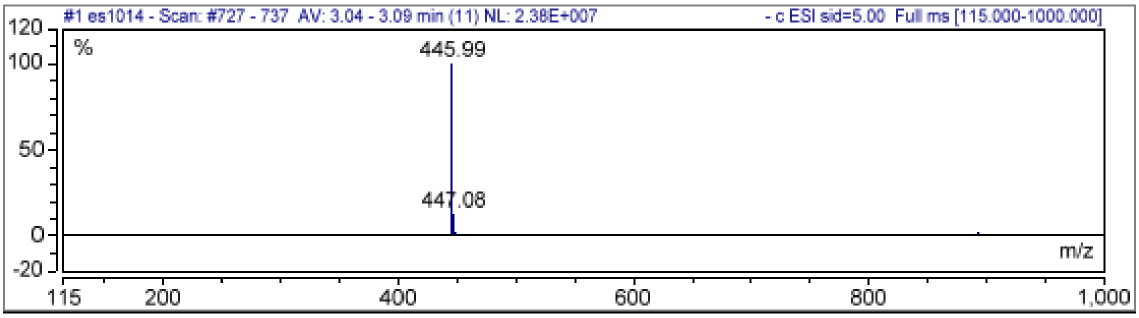

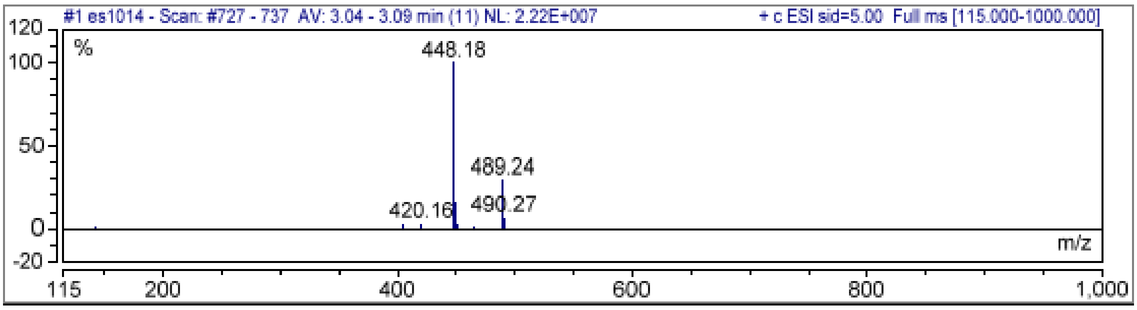

^{1}H NMR (400 MHz, Acetone-d_6) δ 10.50 (s, 2H), 7.69 (d, J = 2.3 Hz, 2H), 7.63 (d, J = 8.7 Hz, 2H), 7.50 (dd, J = 8.8, 2.4 Hz, 2H), 2.33 (s, 3H).

Compound **6**-CF_3

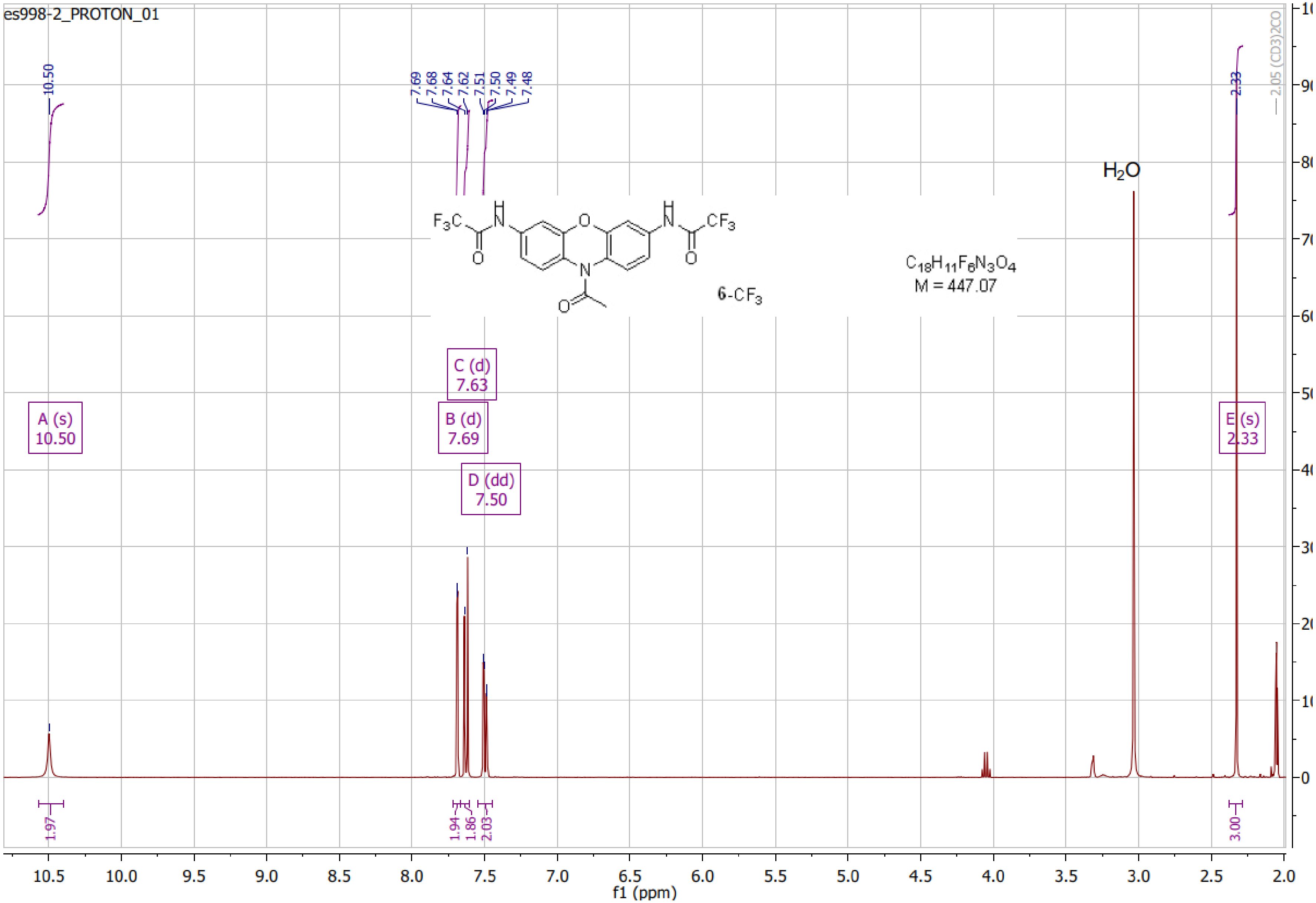

Compound **6**-OBn. ^{1}H-NMR spectrum (acetone-d6) and LC-MS trace:

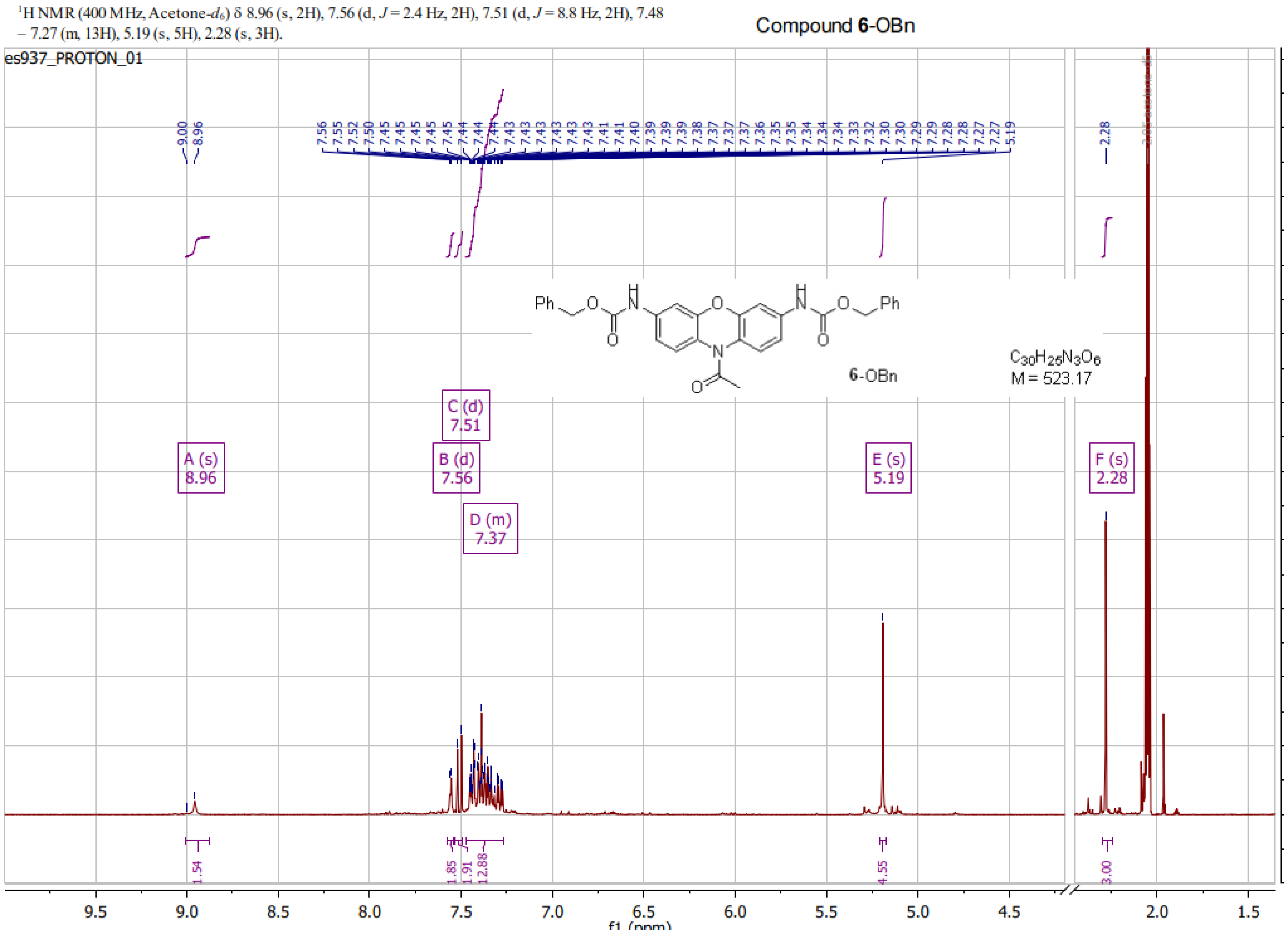

Probe : es950

Lösungsmittel : MeCN/H2O | Aufgabemenge: 3.0 µl
Säule: Phenomenex Kinetex C18 2.6 µm | Länge: 75 mm | iO : 3.0 mm
Fluß (ml / Min) : 0.5 | Temperatur : 25.0
Detektor: DAD-3000 | Pumpe: HPG-3200SD | Sampler: WPS-3000
Laufmittel: A = Acetonitril 0.1% FA | B = Wasser 0.1% FA
Gradient: A 20.0 % B 80.0 % ----> A 100.0 % B 0.0 % T = 10 Min.

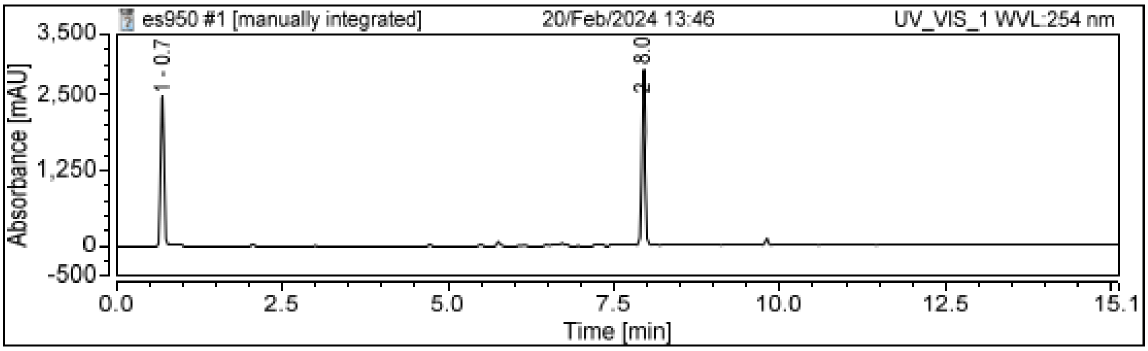

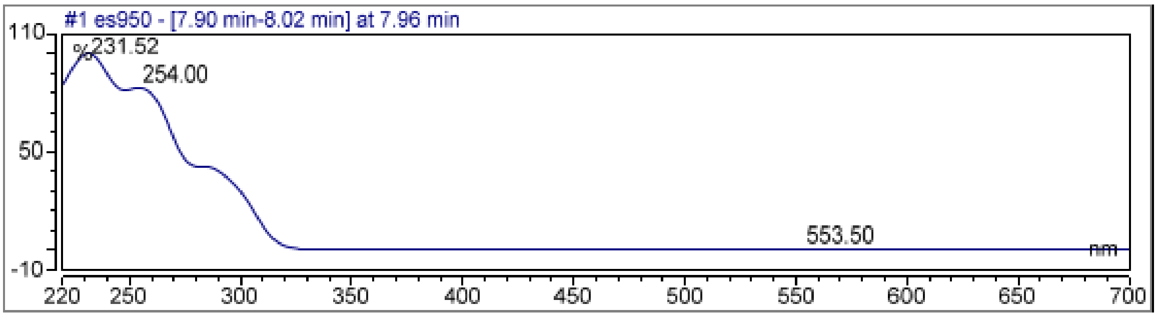

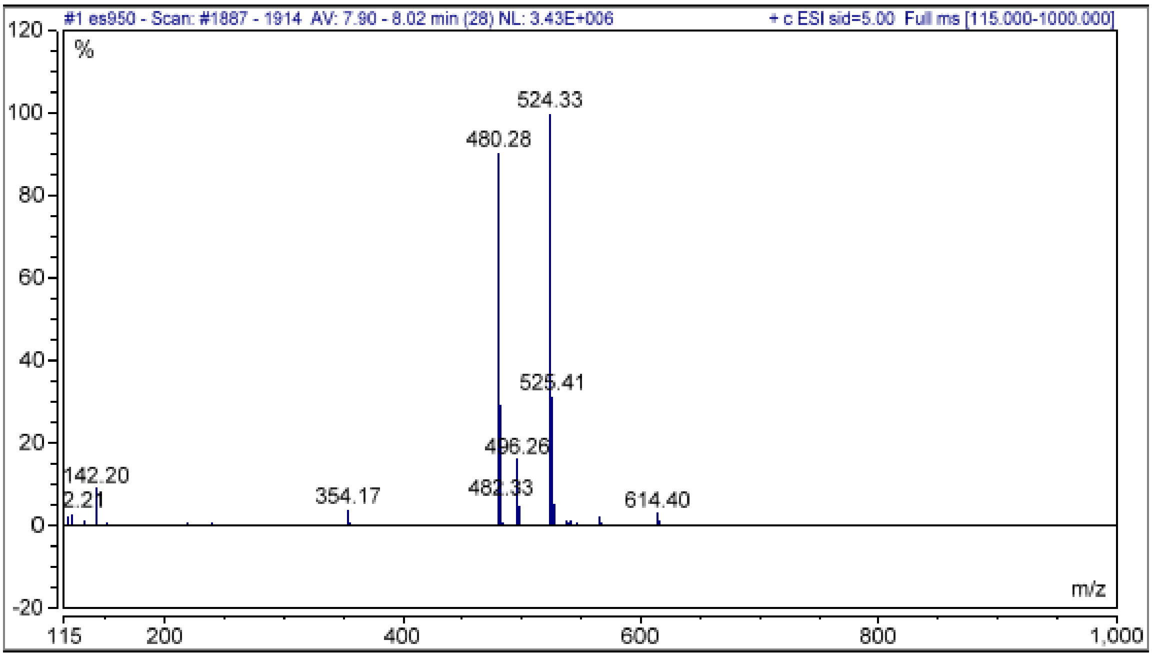

Compound **7a**. LC-MS trace and ^{1}H-NMR spectrum:

Probe : es870 prep HPLC Peak 2 RT=11,7

**Lösungsmittel :**	MeCN/H2O	**Aufgabemenge:**	10.0 µl		
**Säule:**	Phenomenex 2.6 µm	**Länge:**	75 mm	**iO :**	3.0 mm
**Fluß (ml / Min) :**	0.5	**Temperatur :**	25.0		
**Detektor:**	DAD-3000	**Pumpe:**	HPG-3200SD	**Sampler:**	WPS-3000
**Laufmittel:**	A = Acetonitril 0.1% FA			B = Wasser 0.1% FA	
**Gradient:**	A 20.0 % B 80.0 % ---->	A 100.0 % B 0.0 %	T = 10 Min.		

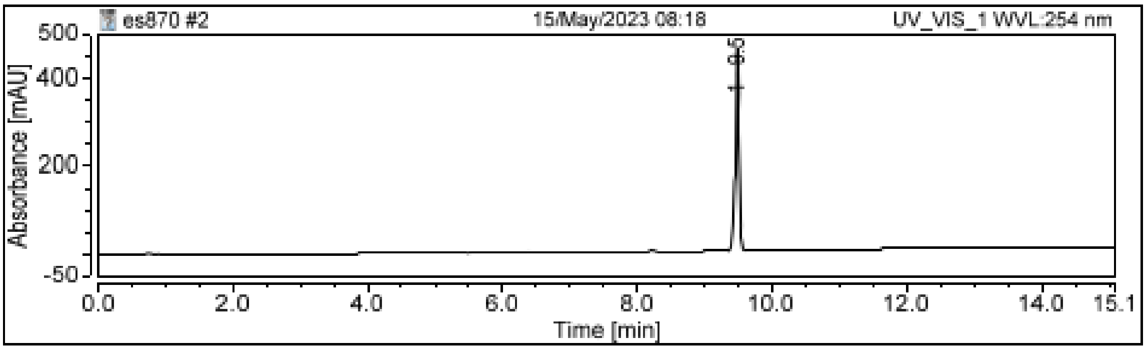

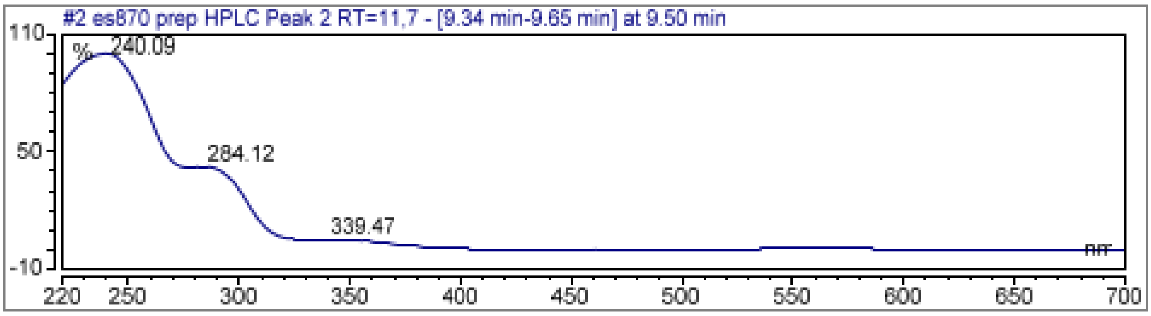

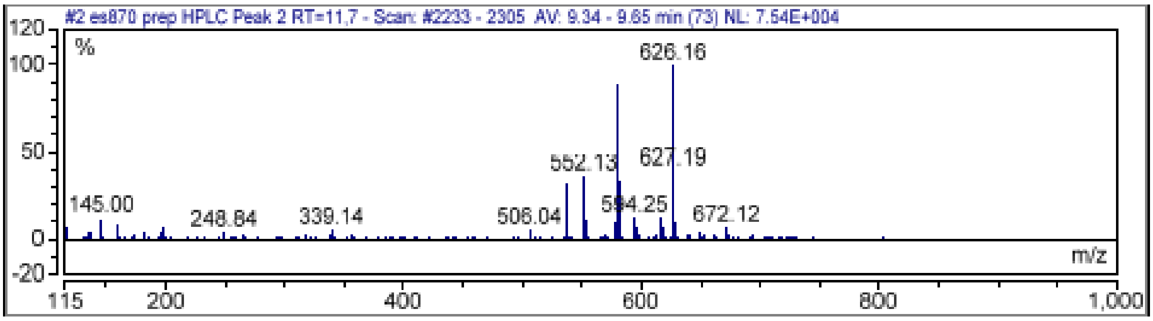

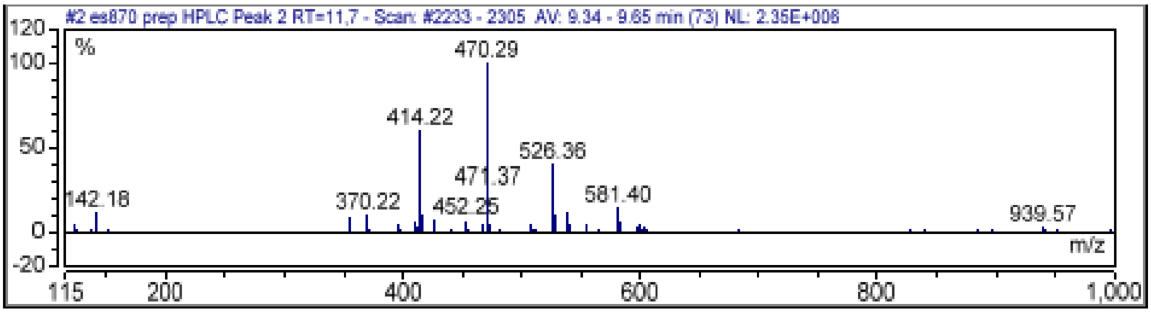

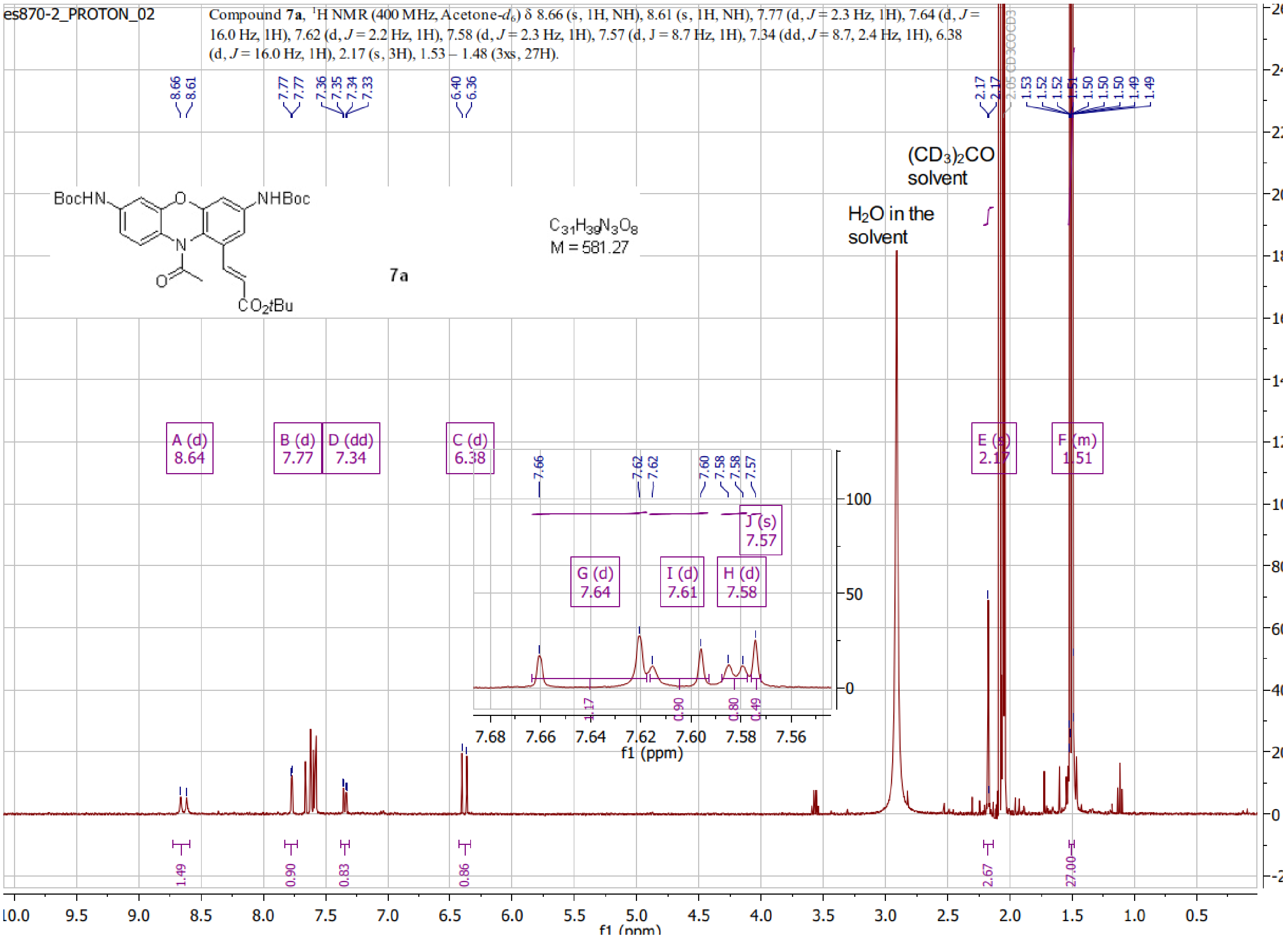
es870-2_PROTON_02
Compound 7a, ¹H NMR (400 MHz, Acetone-d6) δ 8.66 (s, 1H, NH), 8.61 (s, 1H, NH), 7.77 (d, J = 2.3 Hz, 1H), 7.64 (d, J = 16.0 Hz, 1H), 7.62 (d, J = 2.2 Hz, 1H), 7.58 (d, J = 2.3 Hz, 1H), 7.57 (d, J = 8.7 Hz, 1H), 7.34 (dd, J = 8.7, 2.4 Hz, 1H), 6.38 (d, J = 16.0 Hz, 1H), 2.17 (s, 3H), 1.53 – 1.48 (3xs, 27H).
BocHN
NHBoc
CO2tBu
7a
C31H39N3O8
M = 581.27
(CD3)2CO solvent
H2O in the solvent
A (d) 8.64
B (d) 7.77
D (dd) 7.34
C (d) 6.38
E (s) 2.17
F (m) 1.51
G (d) 7.64
I (d) 7.61
H (d) 7.58
J (s) 7.57
f1 (ppm)

^{1}H NMR (400 MHz, Acetone-d_6) δ 8.59 (s, 1H), 8.41 (s, 1H), 7.92 (s, 1H), 7.75 (dd, J = 15.7, 0.5 Hz, 1H), 7.54 – 7.48 (m, 1H), 7.46 (d, J = 8.8 Hz, 1H), 7.27 (dd, J = 8.8, 2.4 Hz, 1H), 6.32 (d, J = 15.7 Hz, 1H), 2.30 (s, 3H), 1.48 – 1.44 (m, 27H).

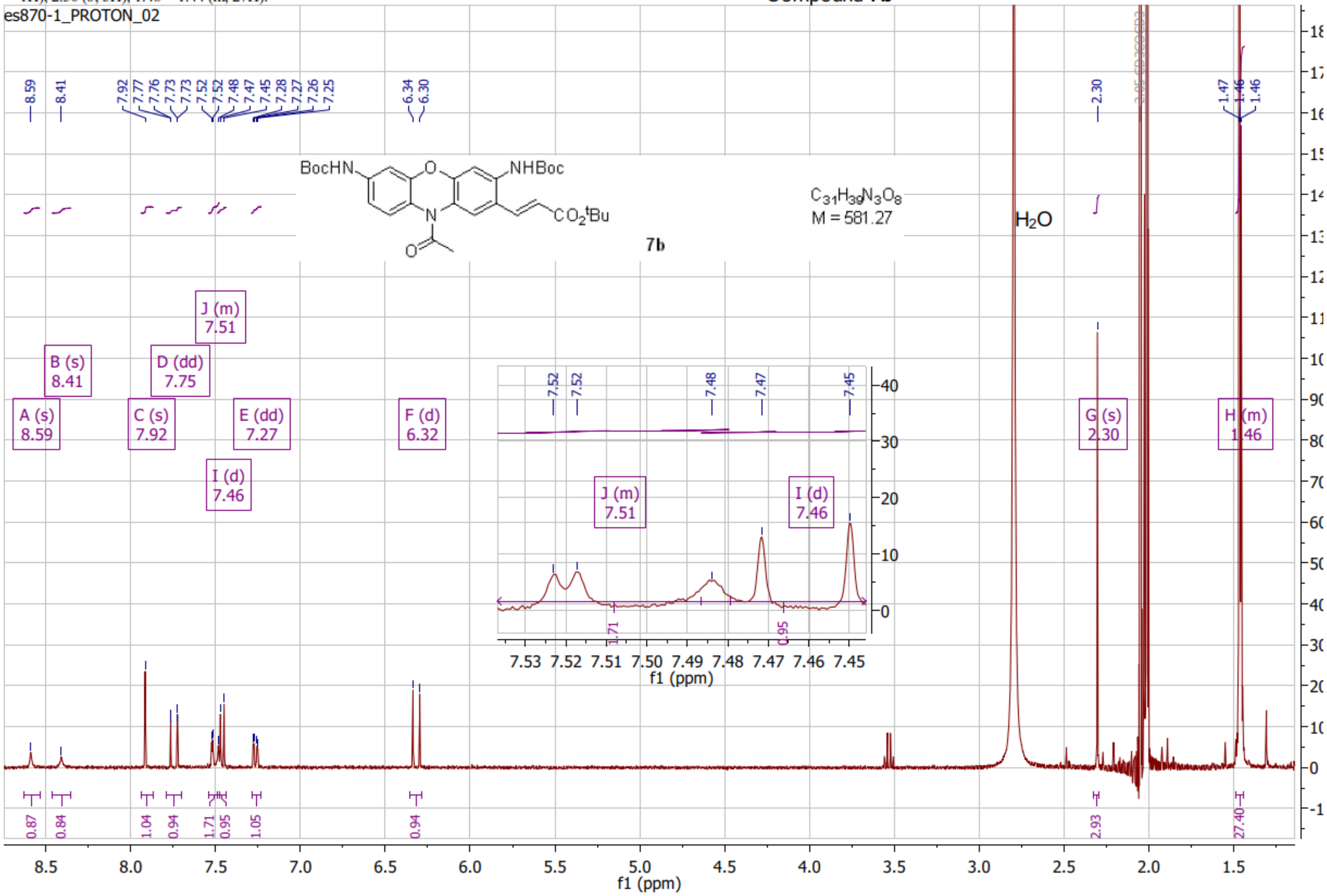

Compound **7b**. ^{1}H-NMR spectrum (acetone-*d*6), LC-MS trace:

Probe : es870 prep HPLC Peak 1 RT=10,4

Lösungsmittel : MeCN/H2O **Aufgabemenge:** 10.0 µl
Säule: Phenomenex 2.6 µm **Länge:** 75 mm **iO :** 3.0 mm
Fluß (ml / Min) : 0.5 **Temperatur :** 25.0
Detektor: DAD-3000 **Pumpe:** HPG-3200SD **Sampler:** WPS-3000
Laufmittel: **A = Acetonitril 0.1% FA** **B = Wasser 0.1% FA**
Gradient: A 20.0 % B 80.0 % ----> A 100.0 % B 0.0 % T = 10 Min.

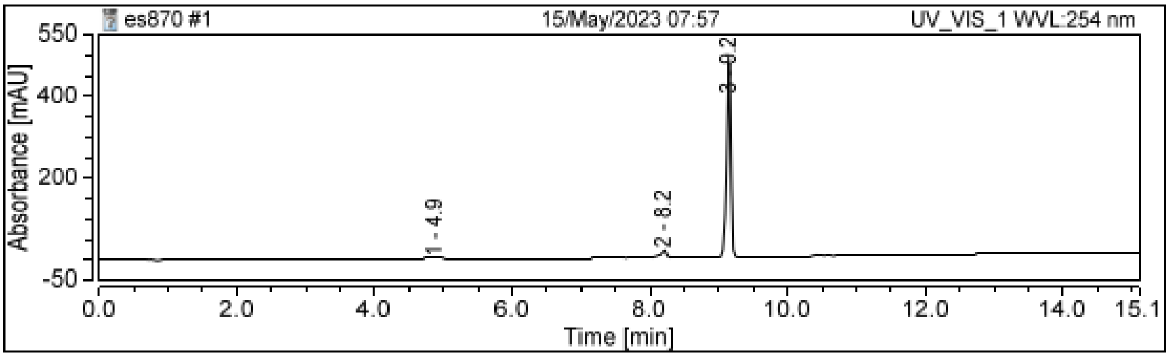

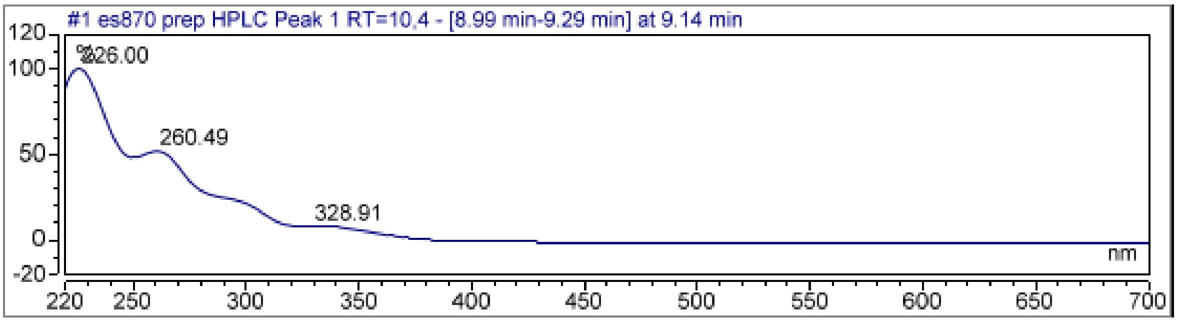

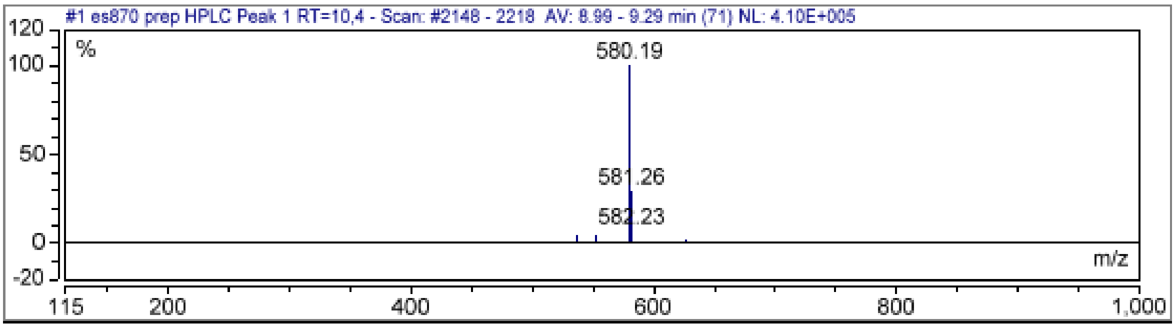

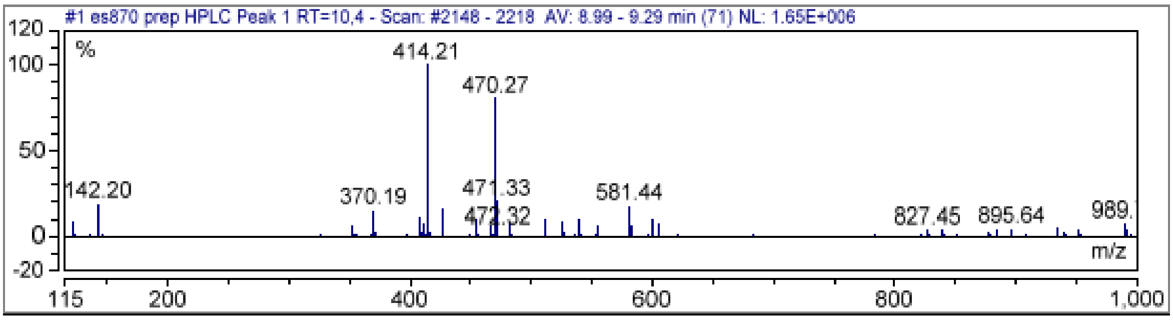

Carboxylic acid **8a**. LC-MS trace and ^{1}H-NMR spectrum (CD_3CN):

Probe : es883 prep peak 1

Lösungsmittel : MeCN/H2O **Aufgabemenge:** 15.0 µl
Säule: Phenomenex Kinetex C18 2.6 µm **Länge:** 75 mm **iO :** 3.0 mm
Fluß (ml / Min) : 0.5 **Temperatur :** 25.0
Detektor: DAD-3000 **Pumpe:** HPG-3200SD **Sampler:** WPS-3000
Laufmittel: **A = Acetonitril 0.1% FA** **B = Wasser 0.1% FA**
Gradient: A 5.0 % B 95.0 % ----> A 50.0 % B 50.0 % T = 10 Min.

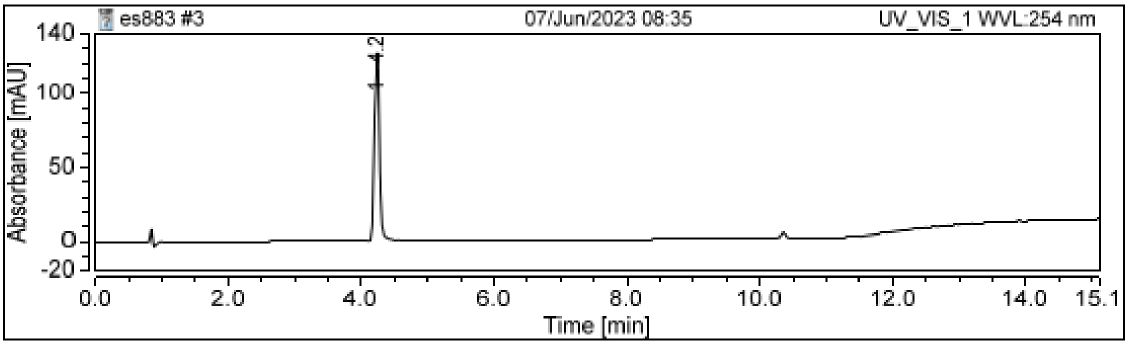

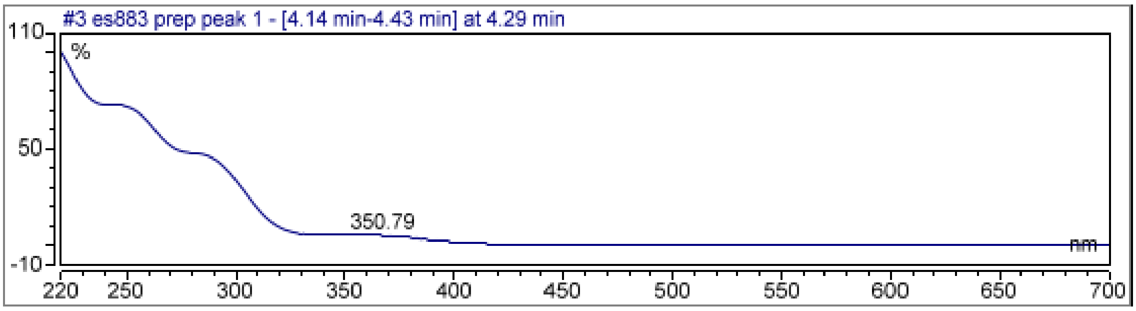

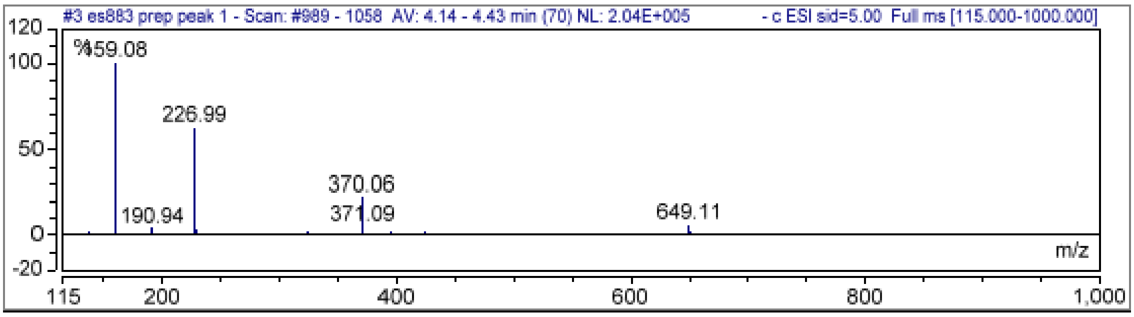

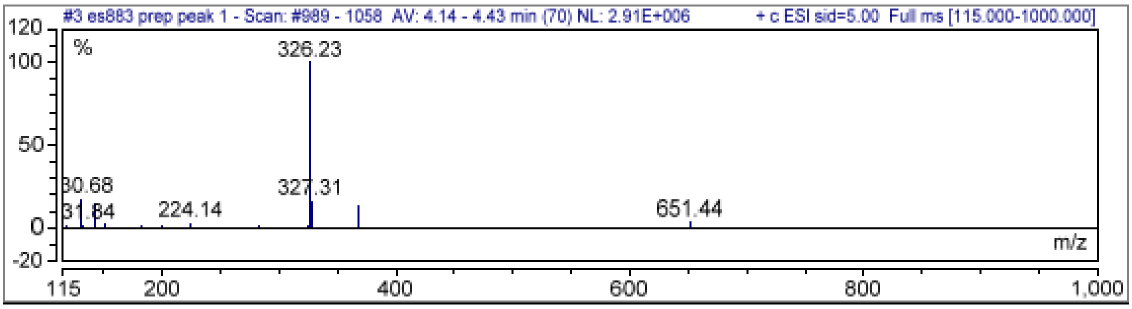

Compound **8a**, ^{1}H NMR (400 MHz, Acetonitrile-d_3) δ 7.58 (d, J = 16.1 Hz, 1H), 7.20 (d, J = 8.8 Hz, 1H), 6.79 (d, J = 2.5 Hz, 1H), 6.50 (d, J = 2.4 Hz, 1H), 6.44 (dd, J = 8.7 and 2.5 Hz, 1H), 6.43 (s, 1H), 6.35 (d, J = 16.1 Hz, 1H), 2.05 (s, 3H)

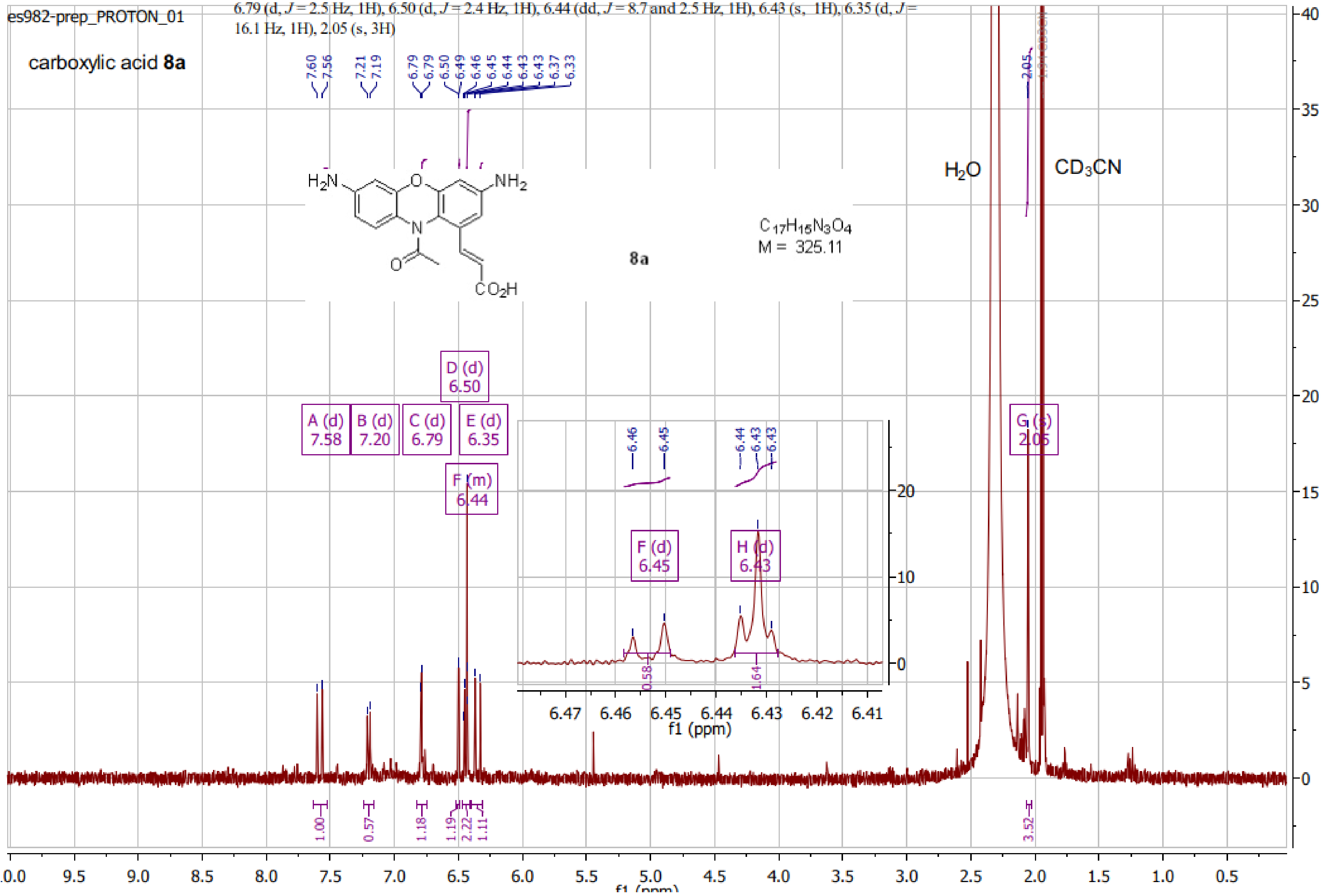

Dye **S1**. ^{1}H-NMR Spectrum (MeOH-d_4):

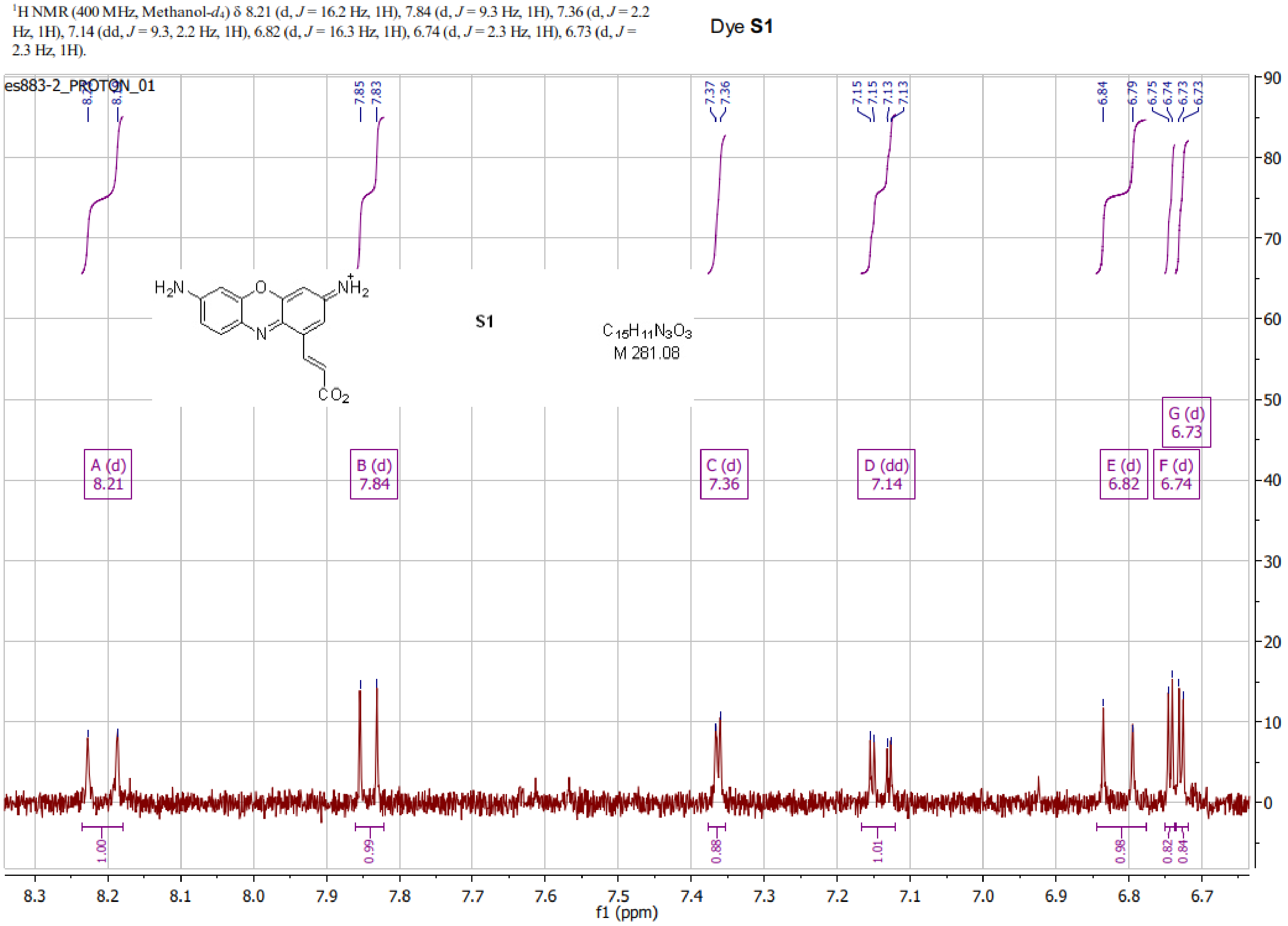

Compound **8b**. LC-MS trace and ^{1}H-NMR spectrum (CD_3CN):

Probe : es982 prep rein

**Lösungsmittel :**	MeCN/H2O		**Aufgabemenge:**	5.0 µl	
**Säule:**	Phenomenex Kinetex C18	2.6 µm	**Länge:** 75 mm	**iO :**	3.0 mm
**Fluß (ml / Min) :**	0.5	**Temperatur :**	25.0		
**Detektor:**	DAD-3000	**Pumpe:**	HPG-3200SD	**Sampler:**	WPS-3000
**Laufmittel:**	**A = Acetonitril 0.1% FA**			**B = Wasser 0.1% FA**	
**Gradient:**	A 5.0 % B 95.0 %	---->	A 50.0 % B 50.0 %	T = 10 Min.	

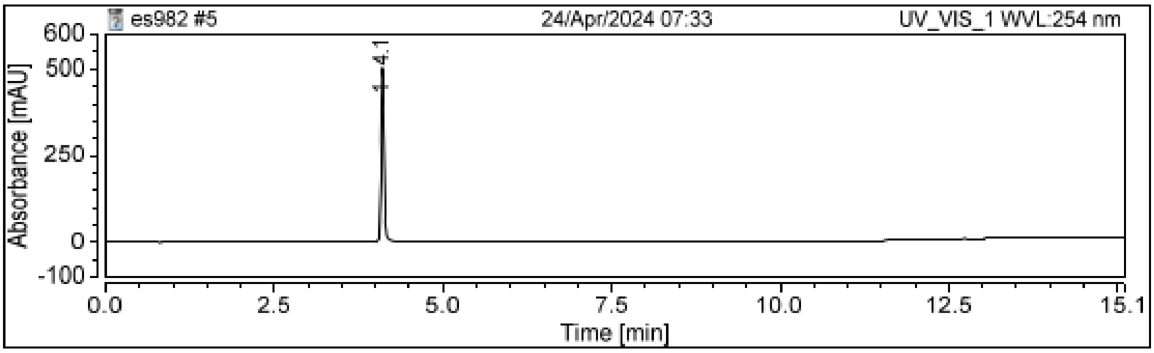

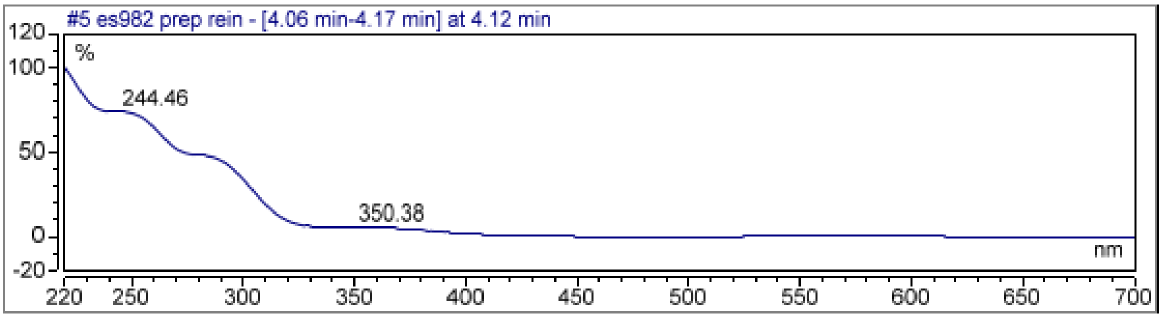

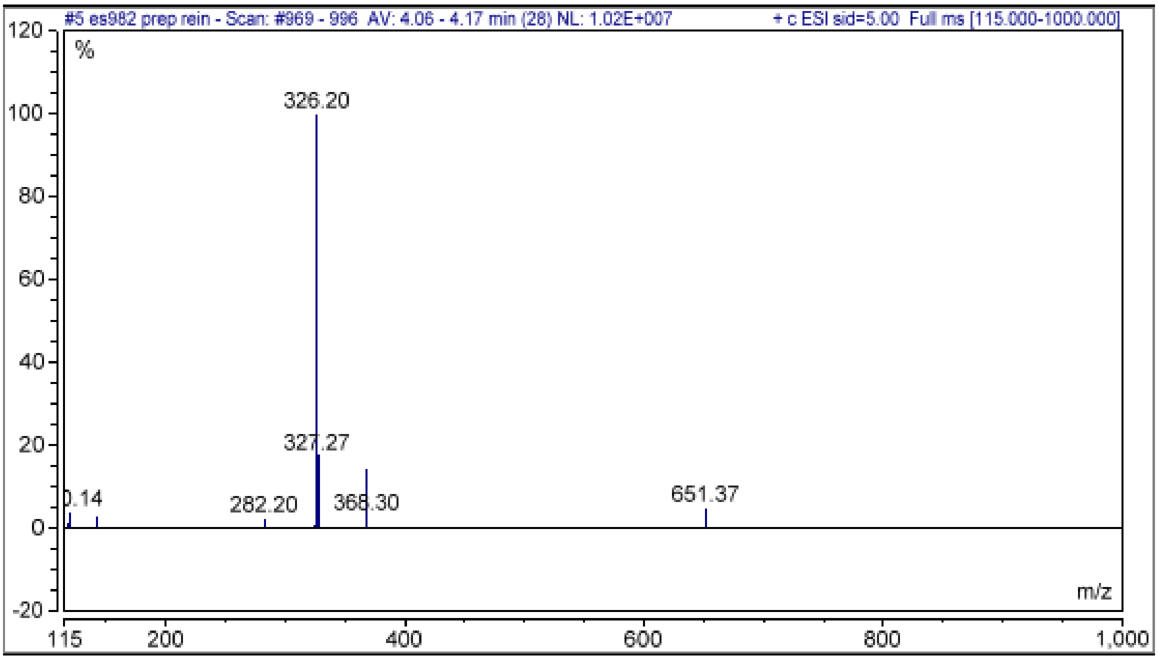

^{1}H NMR (400 MHz, Acetonitrile-d_3) δ 7.59 (dd, J = 15.6, 0.5 Hz, 1H), 7.47 (s, 1H), 7.18 (d, J = 8.2 Hz, 1H), 6.43 (s, 1H), 6.42 – 6.36 (m, 2H), 6.27 (d, J = 15.7 Hz, 1H), 2.18 (s, 3H).

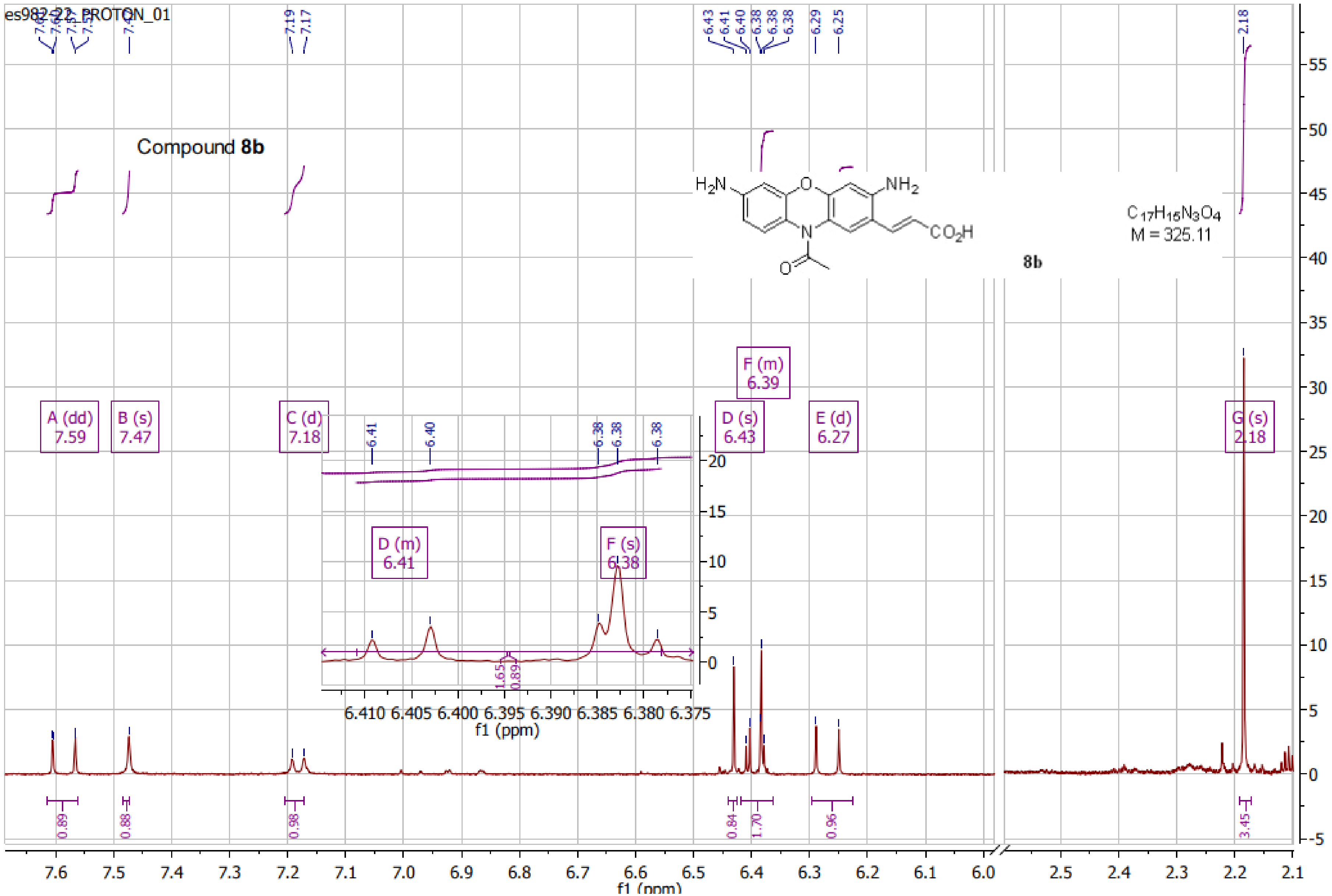

Dye **S2**. ^{1}H-NMR spectrum (600 MHz, MeOH-d_4) and LC-MS trace:

^{1}H NMR (600 MHz, Methanol-d_4) δ 7.92 (d, J = 0.7 Hz, 1H), 7.77 (d, J = 9.2 Hz, 1H), 7.43 (d, J = 15.5 Hz, 1H), 7.15 (dd, J = 9.2, 2.3 Hz, 1H), 6.81 (s, 1H), 6.76 (d, J = 2.3 Hz, 1H), 6.56 (d, J = 15.6 Hz, 1H), 2.15 (s, 4H).

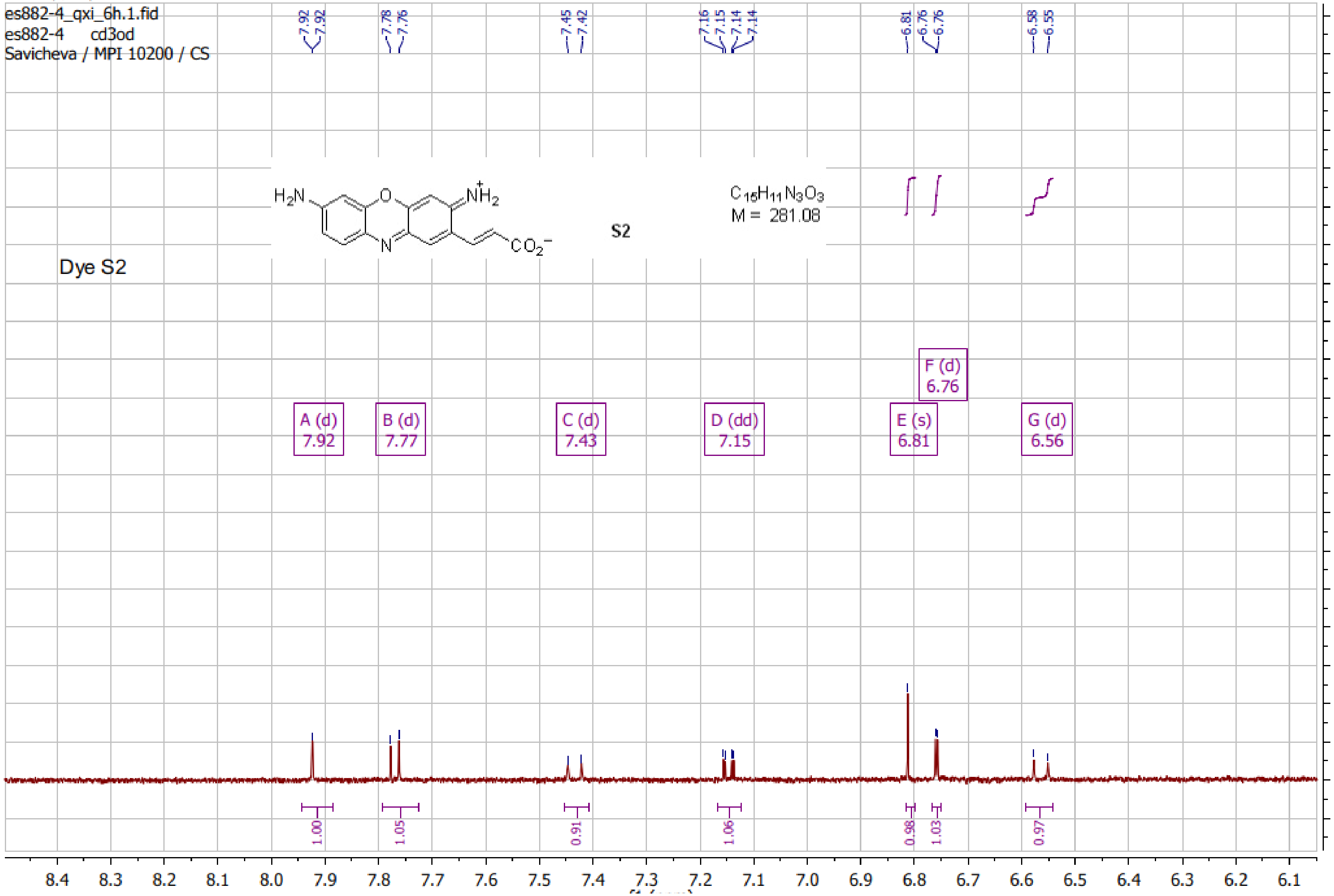

Probe : es882 prep peak4 rt=9,3

Lösungsmittel : MeCN/H2O **Aufgabemenge:** 15.0 µl
Säule: Phenomenex Kinetex C18 2.6 µm **Länge:** 75 mm **iO :** 3.0 mm
Fluß (ml / Min) : 0.5 **Temperatur :** 25.0
Detektor: DAD-3000 **Pumpe:** HPG-3200SD **Sampler:** WPS-3000
Laufmittel: **A = Acetonitril 0.1% FA** **B = Wasser 0.1% FA**
Gradient: A 5.0 % B 95.0 % ----> A 50.0 % B 50.0 % T = 10 Min.

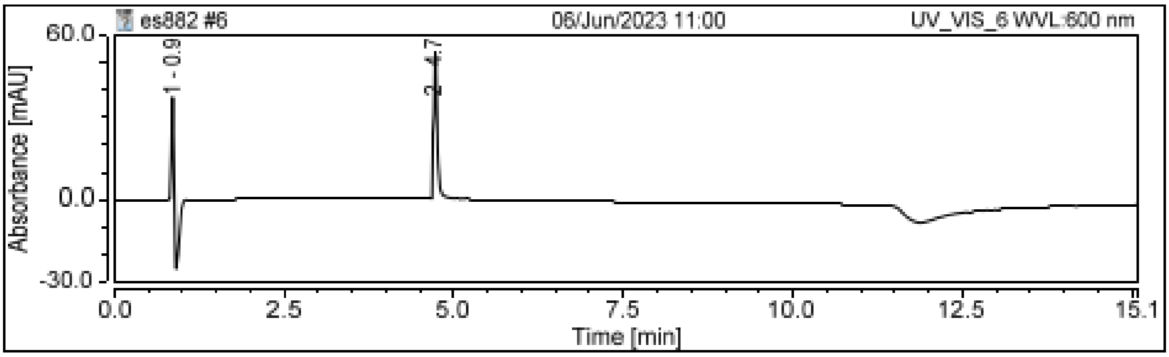

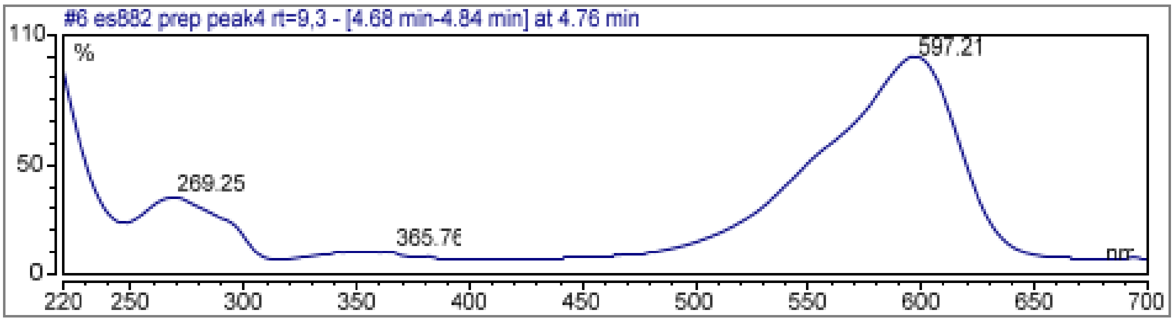

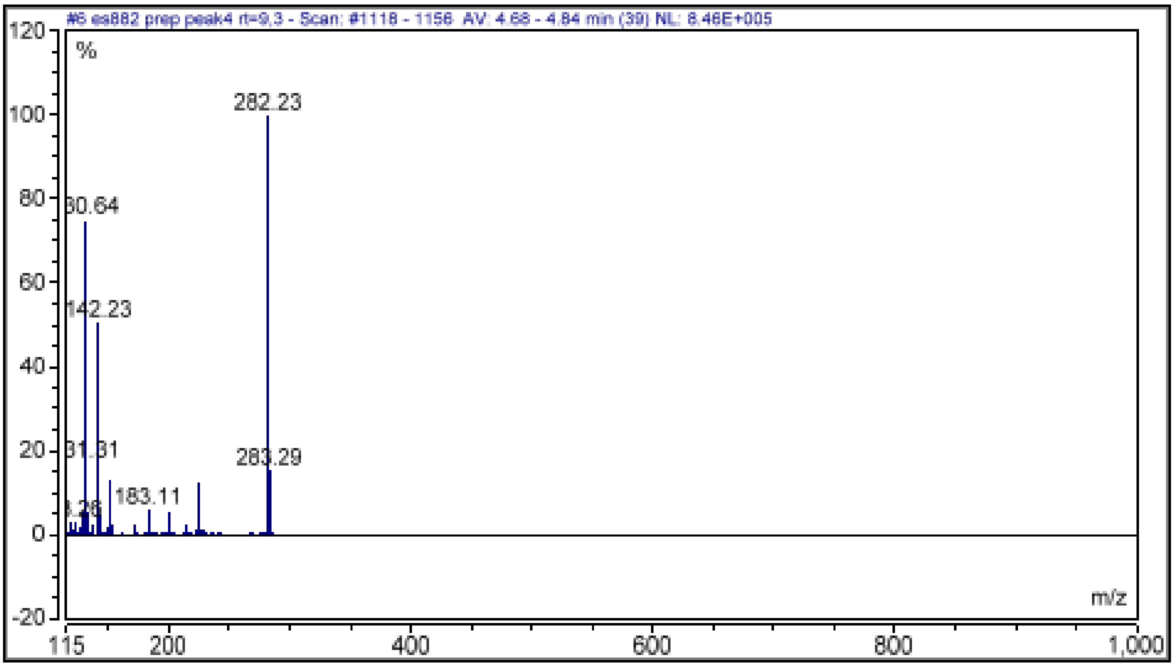

Lactam **S3**. LC-MS trace and ^{1}H-NMR spectrum (DMSO-d_6):

Probe : es882 prep peak2 rt=8,2

Lösungsmittel : MeCN/H2O **Aufgabemenge:** 15.0 µl
Säule: Phenomenex Kinetex C18 2.6 µm **Länge:** 75 mm **iO :** 3.0 mm
Fluß (ml / Min) : 0.5 **Temperatur :** 25.0
Detektor: DAD-3000 **Pumpe:** HPG-3200SD **Sampler:** WPS-3000
Laufmittel: **A = Acetonitril 0.1% FA** **B = Wasser 0.1% FA**
Gradient: A 5.0 % B 95.0 % ---> A 50.0 % B 50.0 % T = 10 Min.

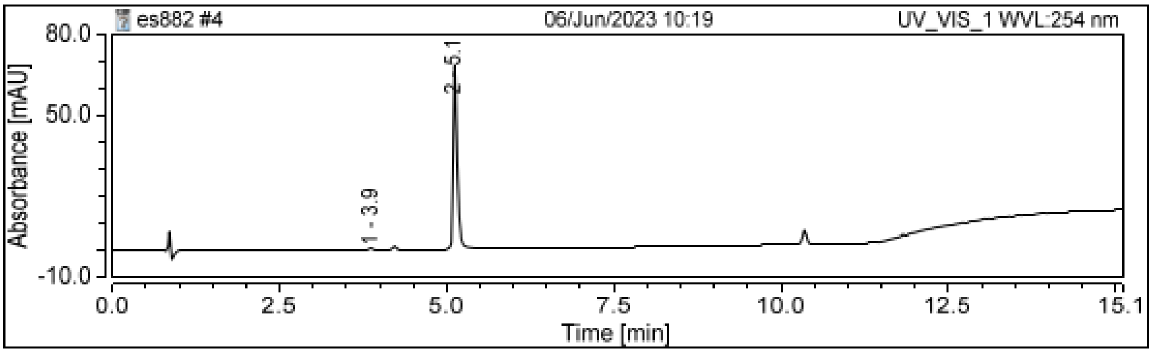

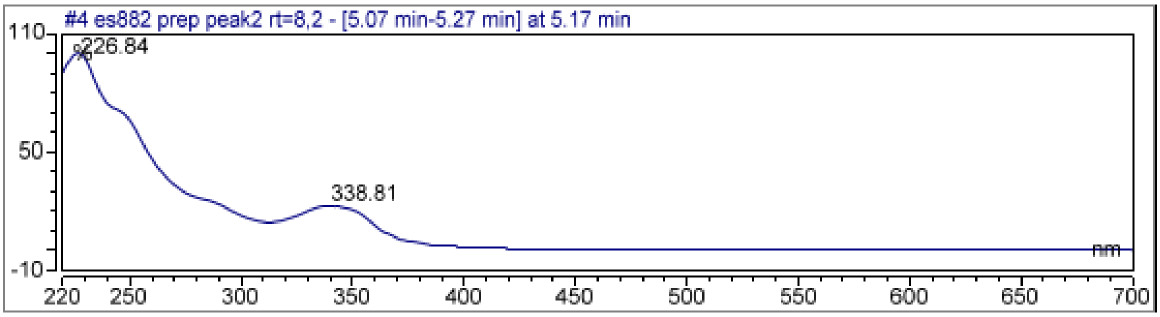

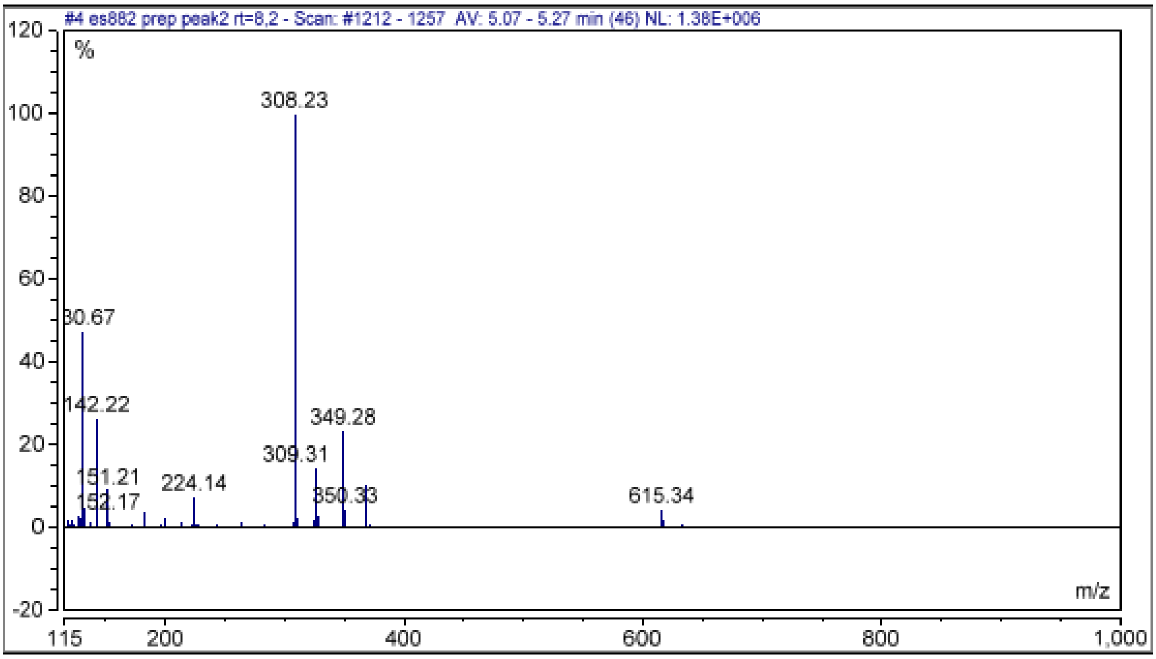

^{1}H NMR (400 MHz, DMSO-d_6) δ 7.89 (d, J = 9.5 Hz, 1H), 7.86 (s, 1H), 7.21 (d, J = 8.5 Hz, 1H), 7.00 (s, 1H), 6.45 – 6.38 (m, 3H), 6.35 (dd, J = 12.6, 2.6 Hz, 1H), 2.22 (s, 3H).

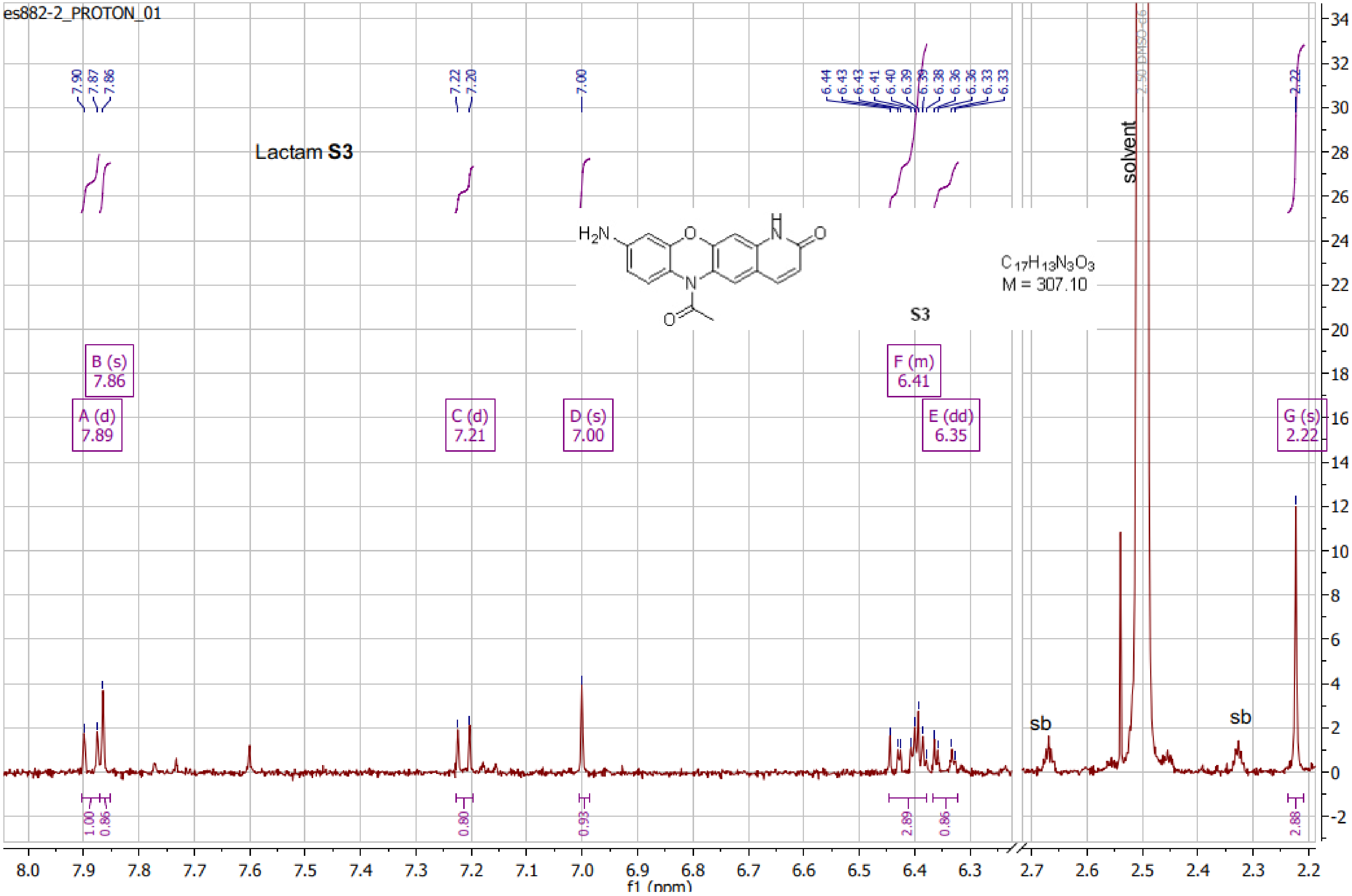

Compound **8c**. ^{1}H-NMR spectrum and LC-MS trace:

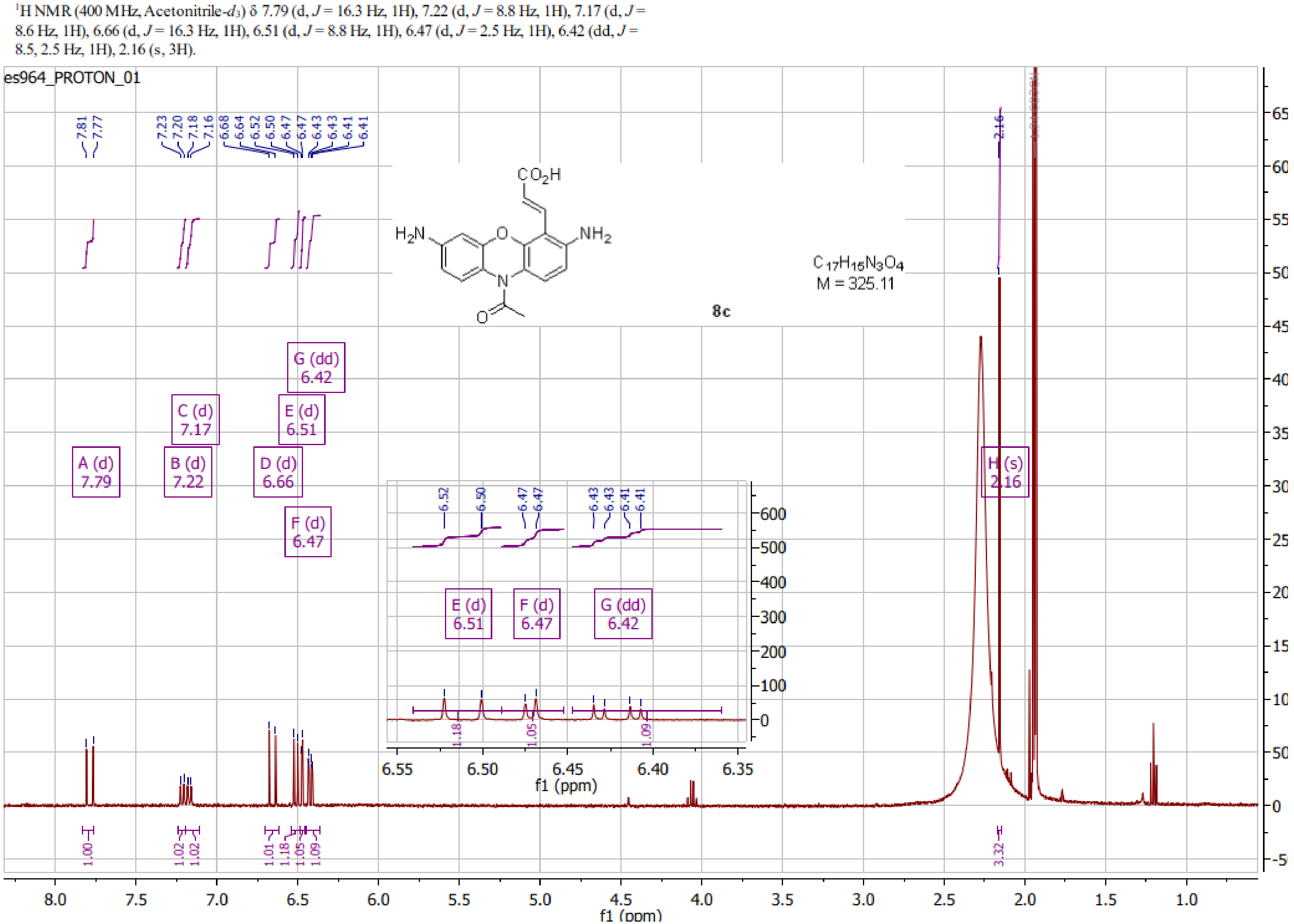

Probe : es964 prep rein

Lösungsmittel : MeCN/H2O **Aufgabemenge:** 10.0 µl
Säule: Phenomenex Kinetex C18 2.6 µm **Länge:** 75 mm **iO :** 3.0 mm
Fluß (ml / Min) : 0.5 **Temperatur :** 25.0
Detektor: DAD-3000 **Pumpe:** HPG-3200SD **Sampler:** WPS-3000
Laufmittel: **A = Acetonitril 0.1% FA** **B = Wasser 0.1% FA**
Gradient: A 5.0 % B 95.0 % ----> A 50.0 % B 50.0 % T = 10 Min.

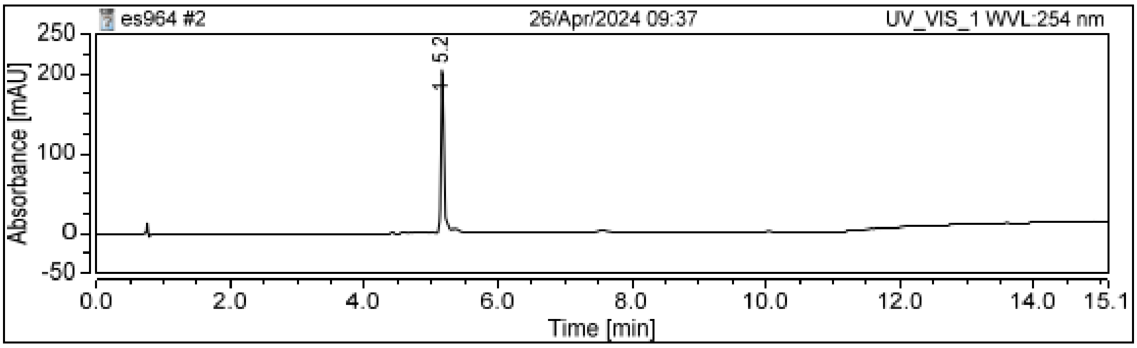

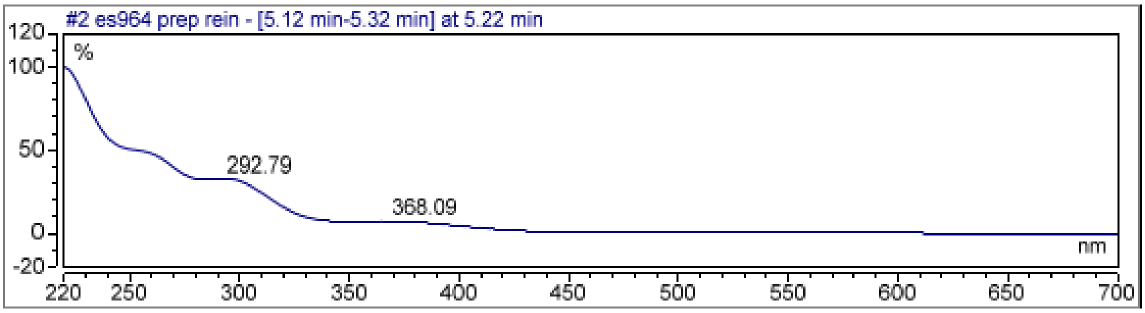

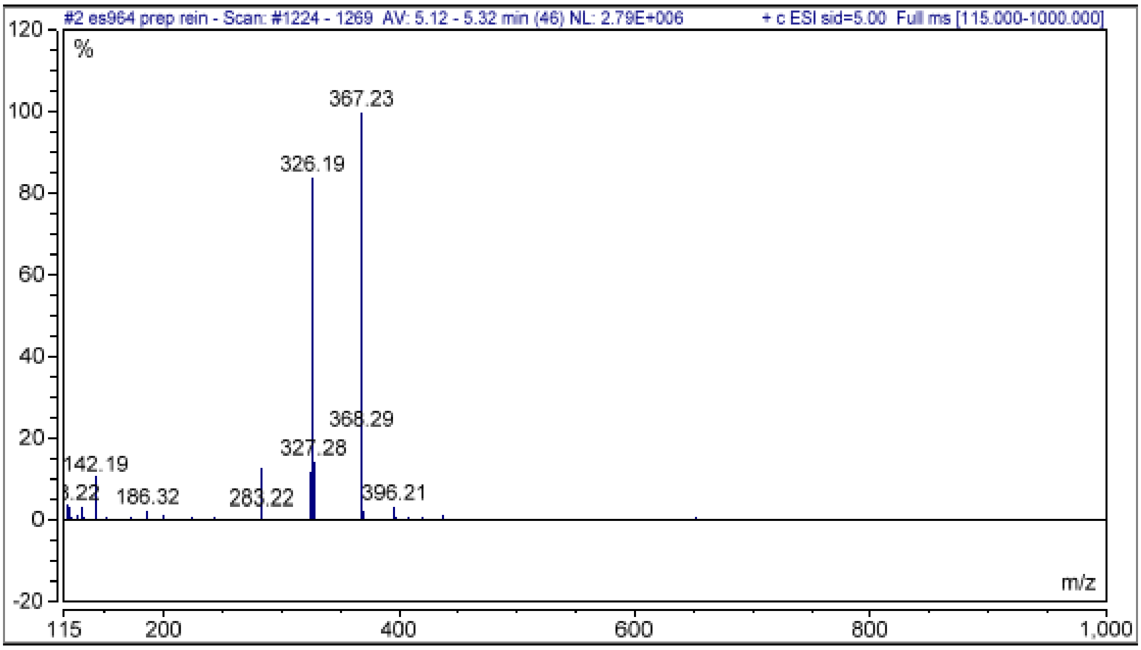

Compound **9**. ^{1}H-NMR spectrum (acetone-d_6):

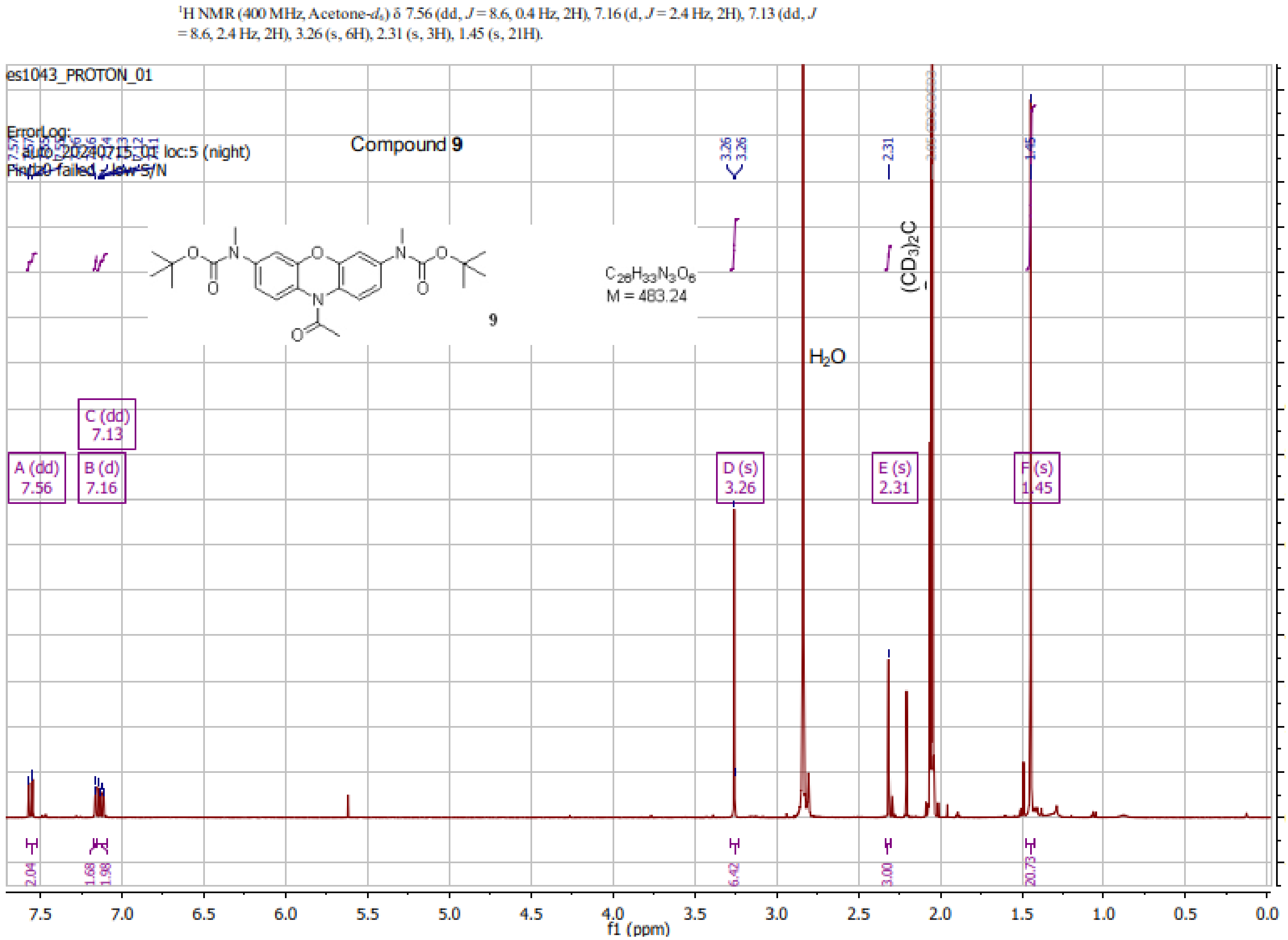

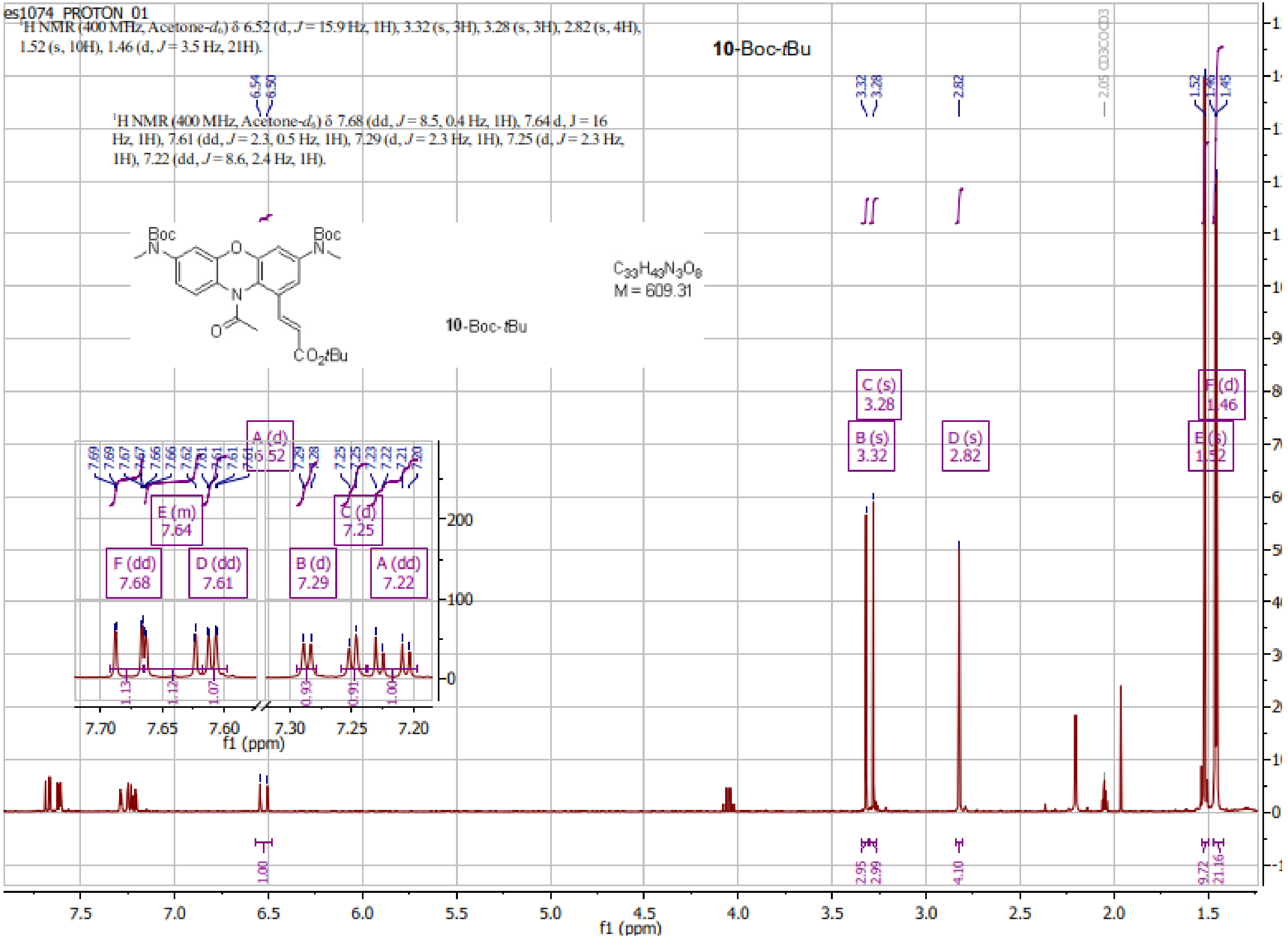

Compound **10**-Boc-*t*Bu. ^{1}H-NMR and ^{13}C-NMR spectra (acetone-d_6):

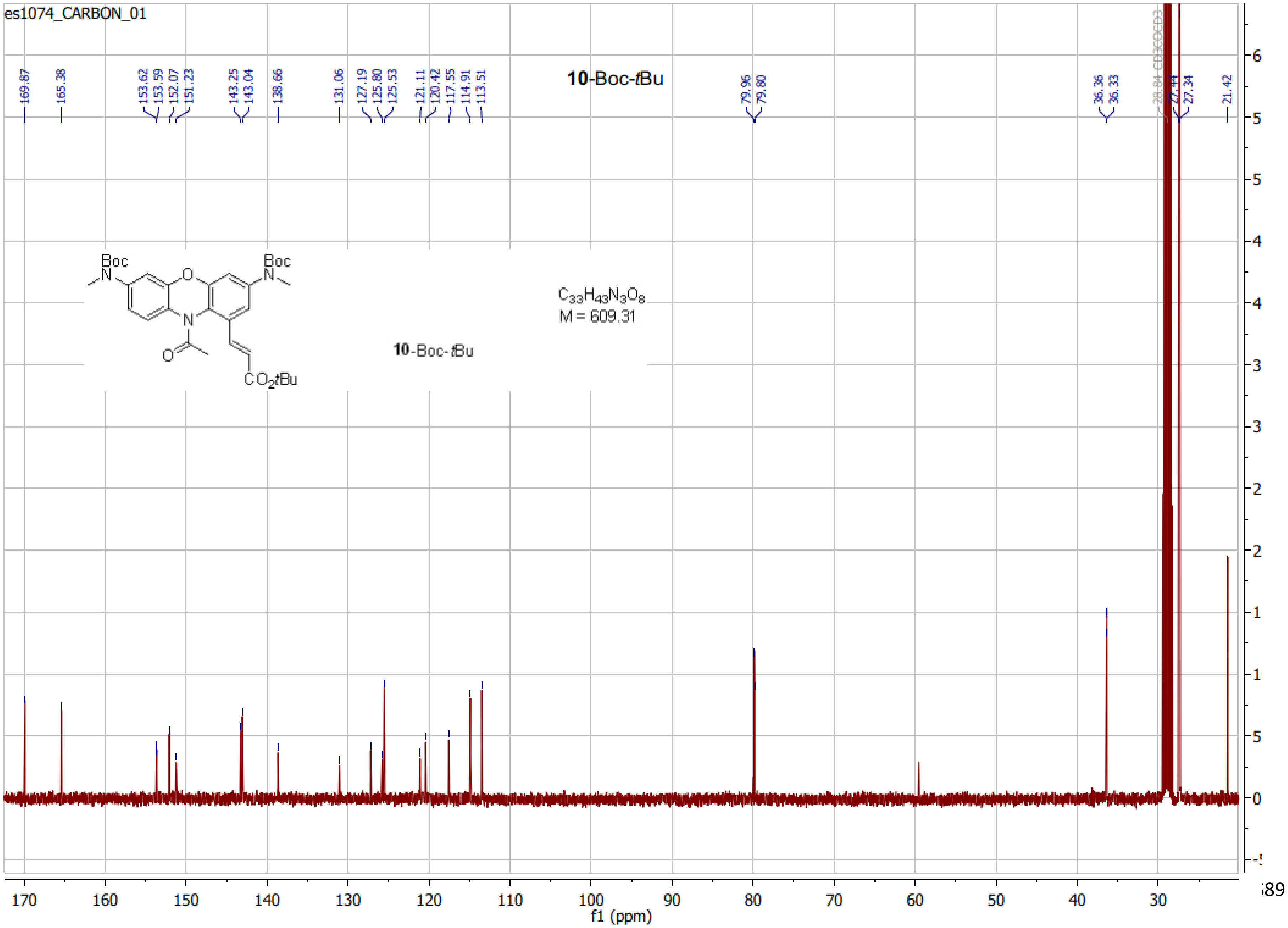
es1074_CARBON_01
10-Boc-tBu
169.87
165.38
153.62
153.59
152.07
151.23
143.25
143.04
138.66
131.06
127.19
125.80
125.53
121.11
120.42
117.55
114.91
113.51
79.96
79.80
36.36
36.33
27.34
21.42
Boc
Boc
N
O
N
N
O
CO2tBu
10-Boc-tBu
C33H43N3O8
M = 609.31
170
160
150
140
130
120
110
100
90
80
70
60
50
40
30
f1 (ppm)

Compound **10**-H-H. LC-MS trace and ^{1}H-NMR-spectrum (CD_3CN):

Probe : es1077 13

**Lösungsmittel :**	**MeCN/H2O**		**Aufgabemenge:**	**0.5 µl**	
**Säule:**	Phenomenex Kinetex C18	1.7 µm	**Länge:** 50 mm	**iO :**	2.1 mm
**Fluß (ml / Min) :**	**0.5**	**Temperatur :**	**25.0**		
**Detektor:**	DAD-3000	**Pumpe:**	HPG-3200SD	**Sampler:**	WPS-3000
**Laufmittel:**	**A = Acetonitril 0.1% FA**			**B = Wasser 0.1% FA**	
**Gradient:**	A 20.0 % B 80.0 %	---->	A 100.0 % B 0.0 %	T = 4 Min.	

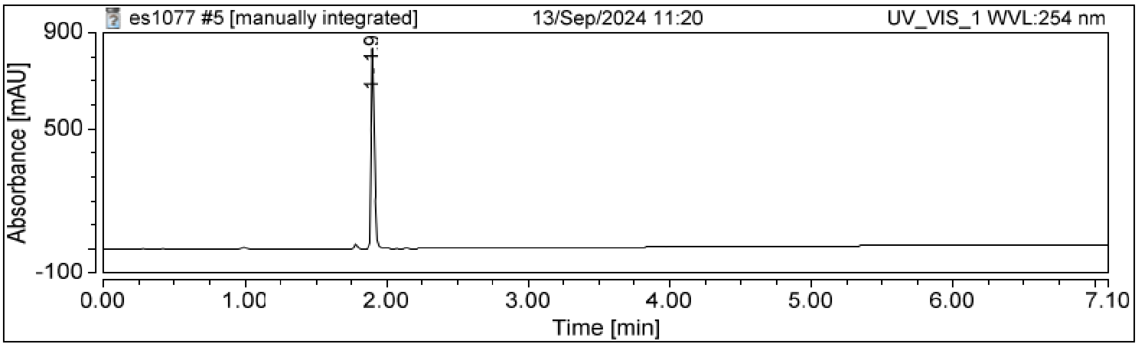

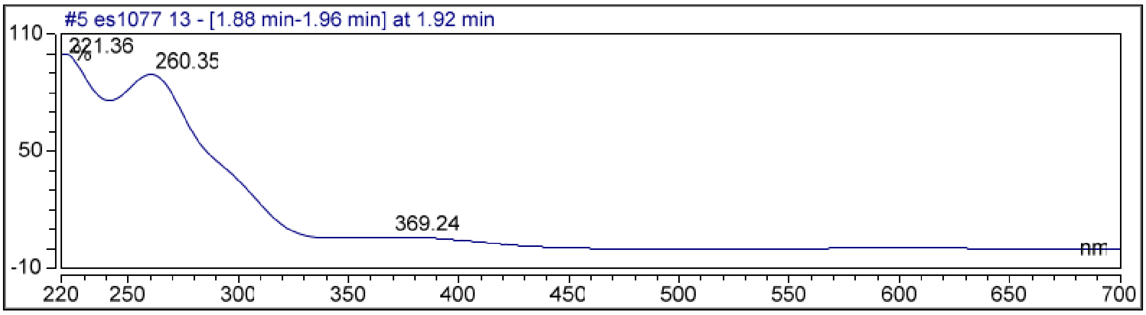

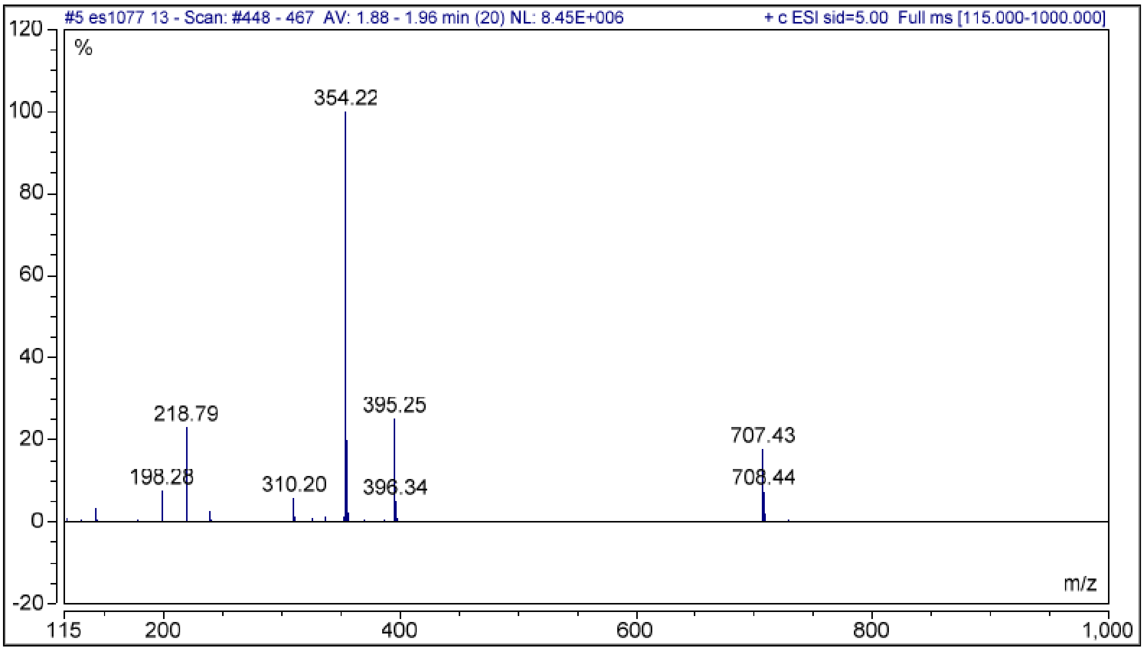

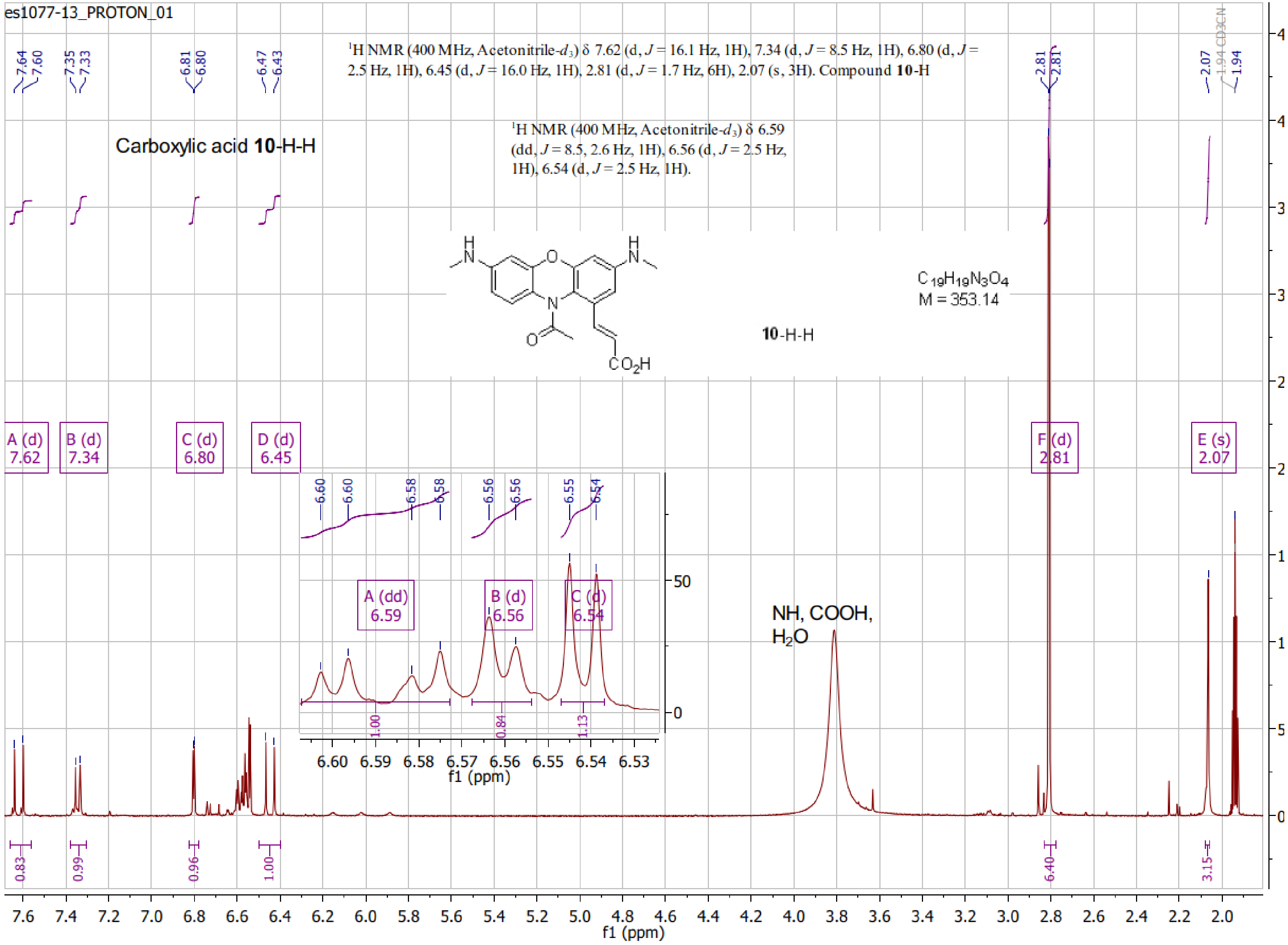

es1077-13_PROTON_01
¹H NMR (400 MHz, Acetonitrile-d_3) δ 7.62 (d, J = 16.1 Hz, 1H), 7.34 (d, J = 8.5 Hz, 1H), 6.80 (d, J = 2.5 Hz, 1H), 6.45 (d, J = 16.0 Hz, 1H), 2.81 (d, J = 1.7 Hz, 6H), 2.07 (s, 3H). Compound **10**-H
¹H NMR (400 MHz, Acetonitrile-d_3) δ 6.59 (dd, J = 8.5, 2.6 Hz, 1H), 6.56 (d, J = 2.5 Hz, 1H), 6.54 (d, J = 2.5 Hz, 1H).
Carboxylic acid **10**-H-H
10-H-H
$C_{19}H_{19}N_3O_4$
M = 353.14
A (d) 7.62
B (d) 7.34
C (d) 6.80
D (d) 6.45
F (d) 2.81
E (s) 2.07
A (dd) 6.59
B (d) 6.56
C (d) 6.54
NH, COOH, H_2O
1.94 CD3CN
f1 (ppm)

Compound **11-**OBn. ^{1}H-NMR, LC-MS trace and ^{13}C-NMR spectrum:

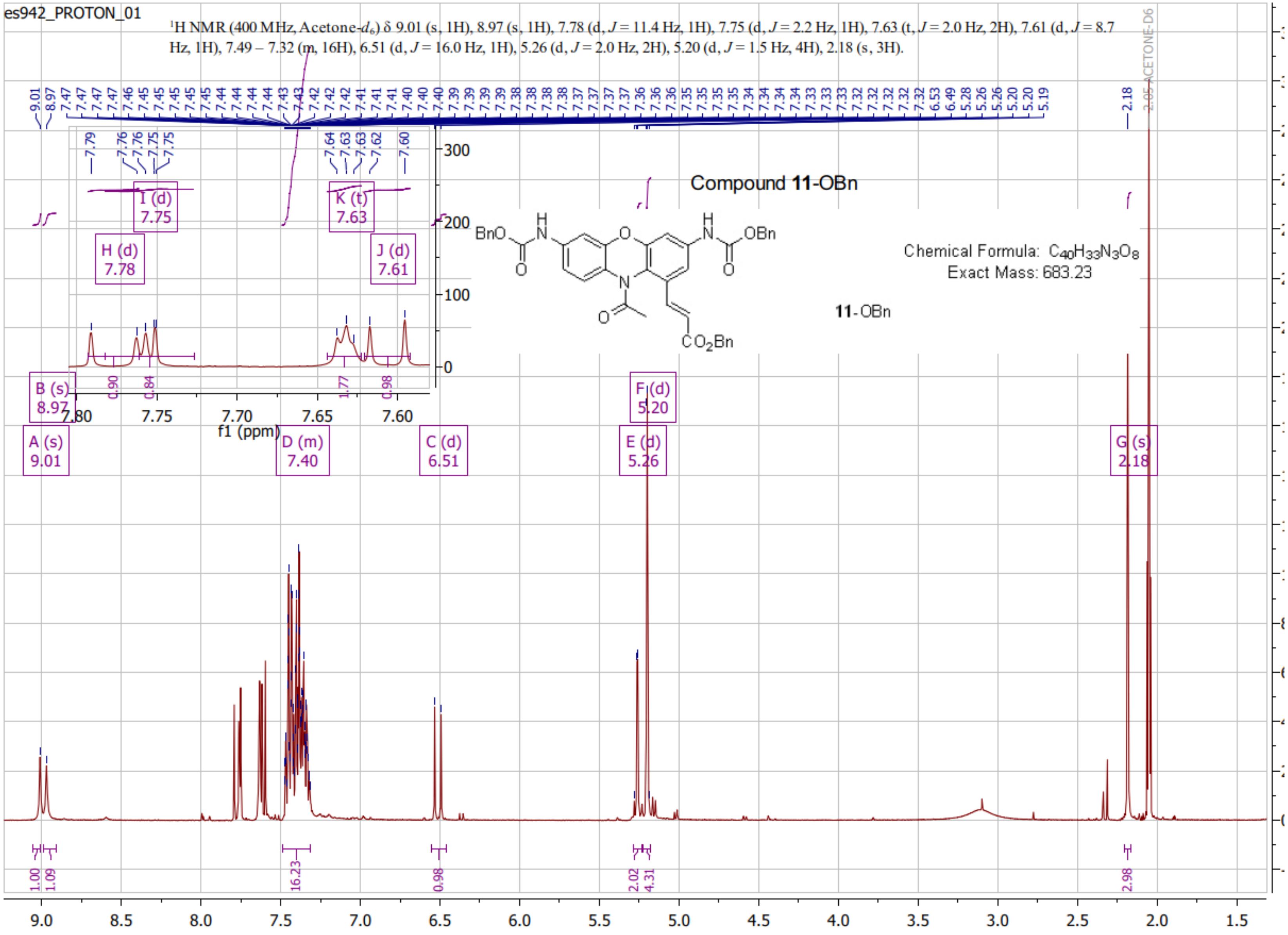

Probe : es942 HPLC Rein

Lösungsmittel : MeCN/H2O **Aufgabemenge:** 3.0 µl
Säule: Phenomenex Kinetex C18 2.6 µm **Länge:** 75 mm **iO :** 3.0 mm
Fluß (ml / Min) : 0.5 **Temperatur :** 25.0
Detektor: DAD-3000 **Pumpe:** HPG-3200SD **Sampler:** WPS-3000
Laufmittel: **A = Acetonitril 0.1% FA** **B = Wasser 0.1% FA**
Gradient: A 20.0 % B 80.0 % ----> A 100.0 % B 0.0 % T = 10 Min.

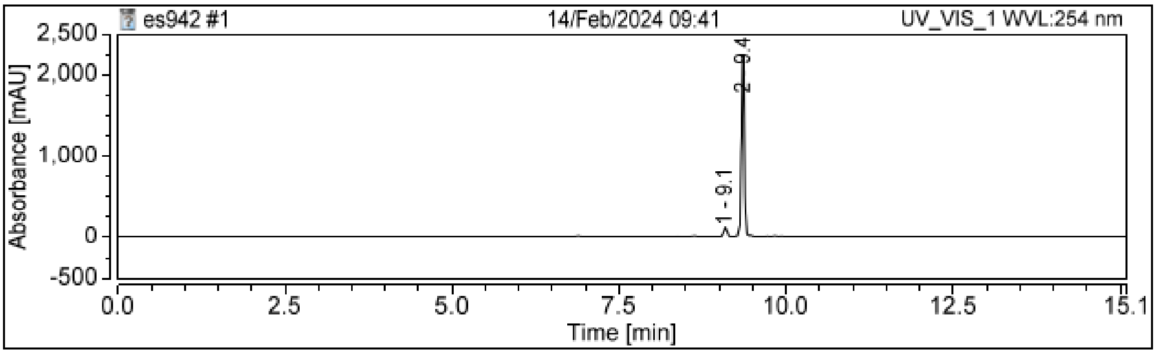

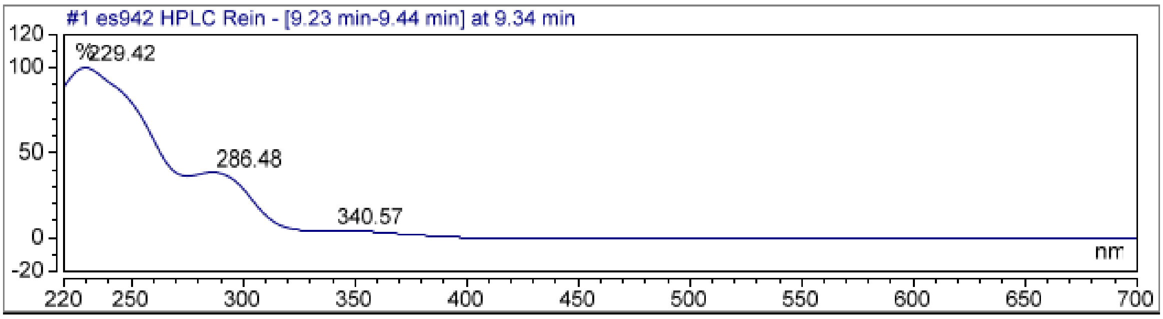

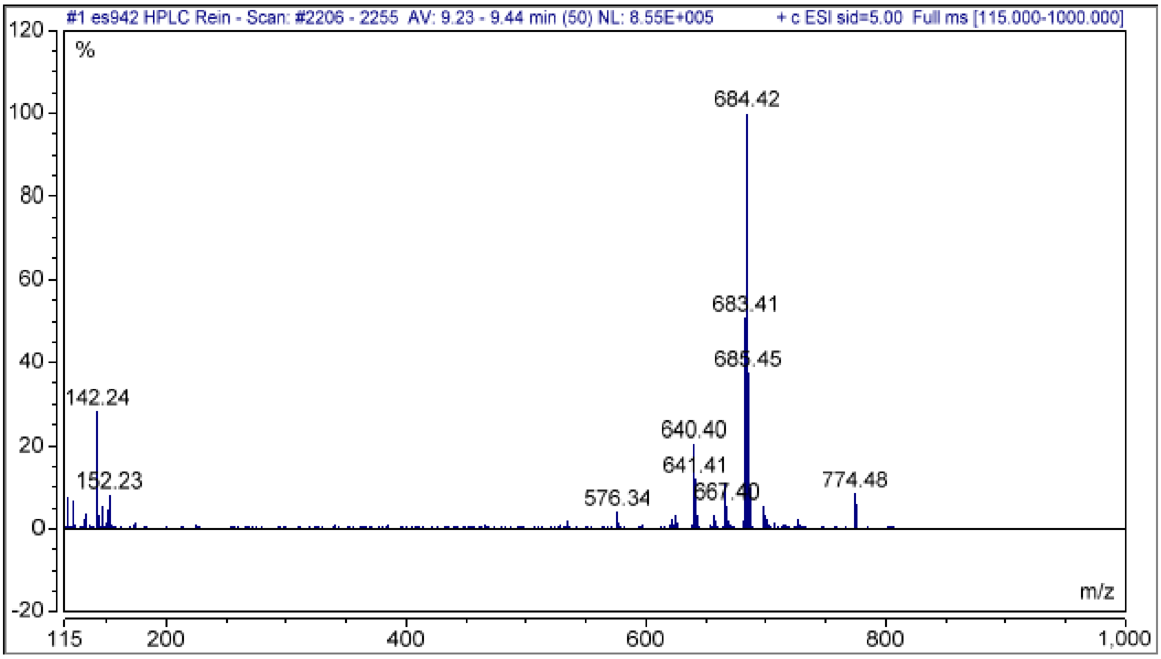

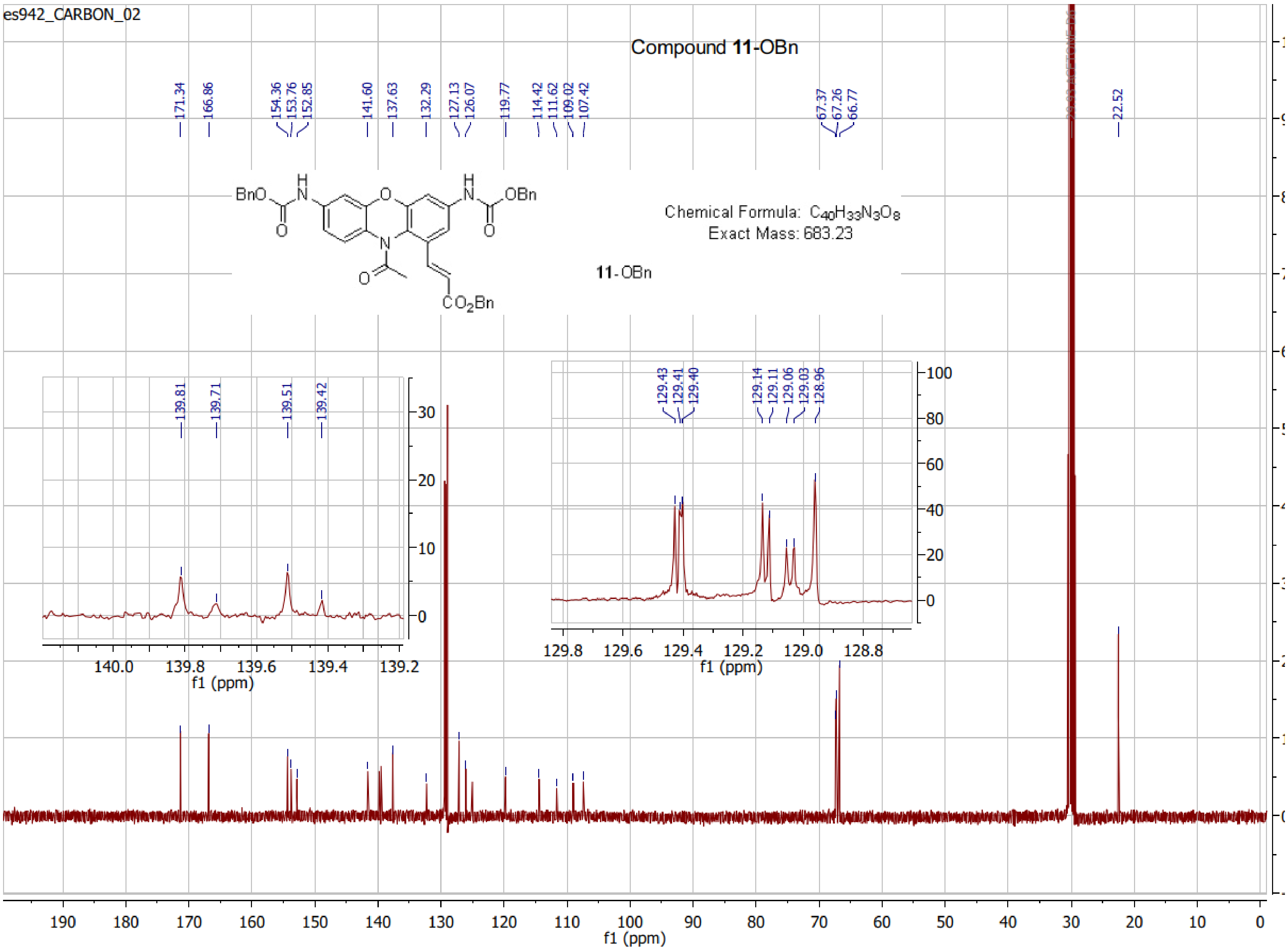

es942_CARBON_02
Compound 11-OBn
171.34
166.86
154.36
153.76
152.85
141.60
137.63
132.29
127.13
126.07
119.77
114.42
111.62
109.02
107.42
67.37
67.26
66.77
22.52
Chemical Formula: C40H33N3O8
Exact Mass: 683.23
11-OBn
BnO
OBn
CO2Bn
139.81
139.71
139.51
139.42
129.43
129.41
129.40
129.14
129.11
129.06
129.03
128.96
f1 (ppm)

Compound **11**-CF_3. ^{1}H-NMR (acetone-d_6, and LC-MS trace:

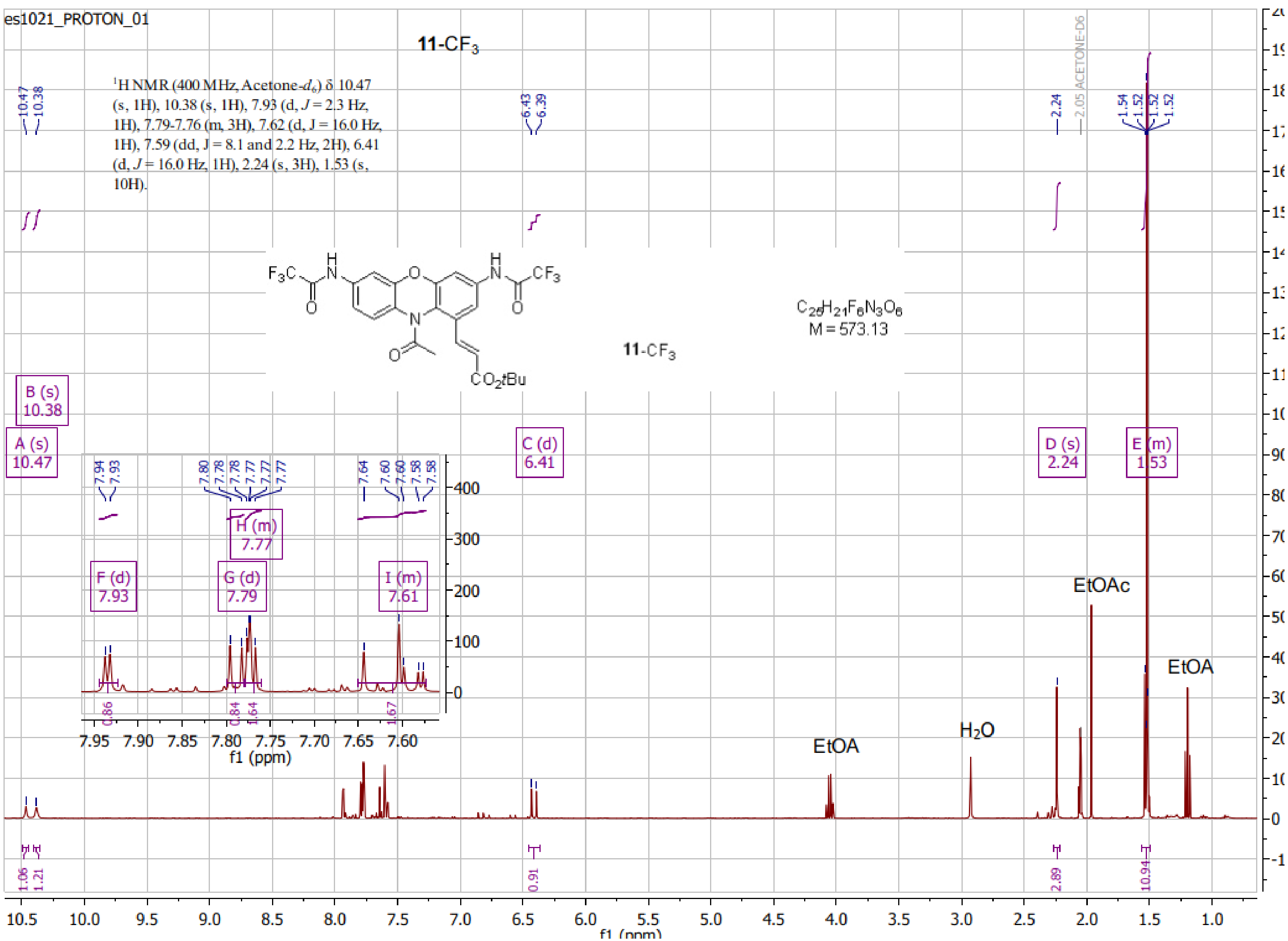

Probe : es1034-2

Lösungsmittel : MeCN/H2O
Aufgabemenge: 1.0 µl
Säule: Phenomenex Kinetex C18 1.7 µm
Länge: 50 mm
iO : 2.1 mm
Fluß (ml / Min) : 0.5
Temperatur : 25.0
Detektor: DAD-3000
Pumpe: HPG-3200SD
Sampler: WPS-3000
Laufmittel: A = Acetonitril 0.1% FA
B = Wasser 0.1% FA
Gradient: A 20.0 % B 80.0 % ----> A 100.0 % B 0.0 % T = 4 Min.

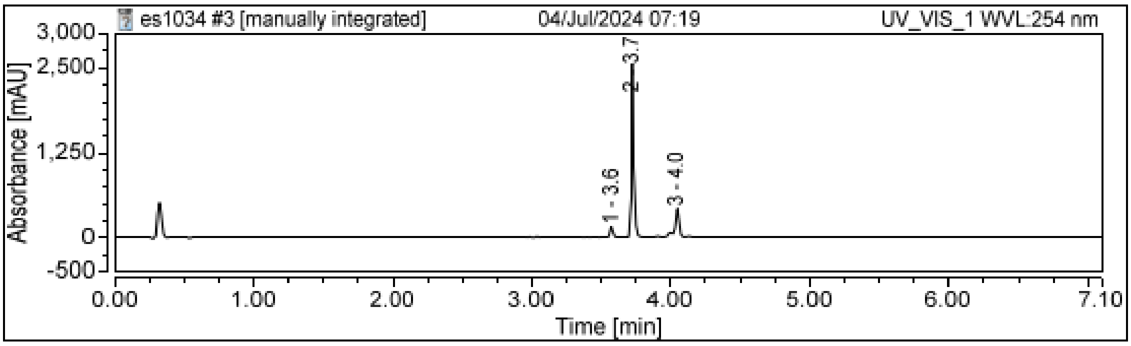

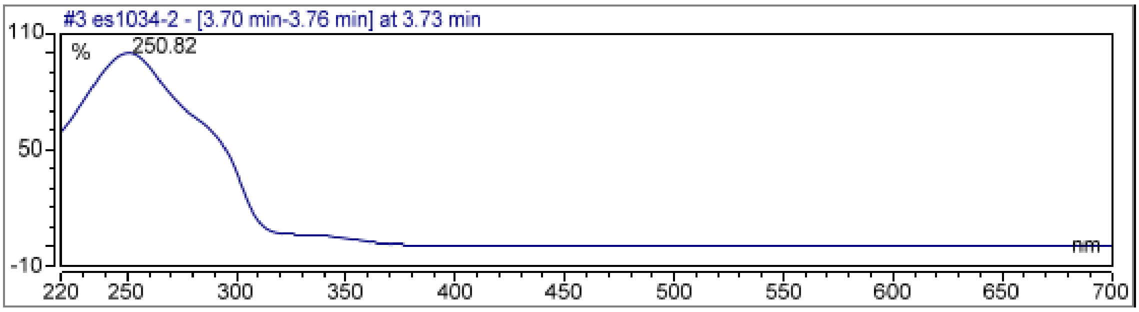

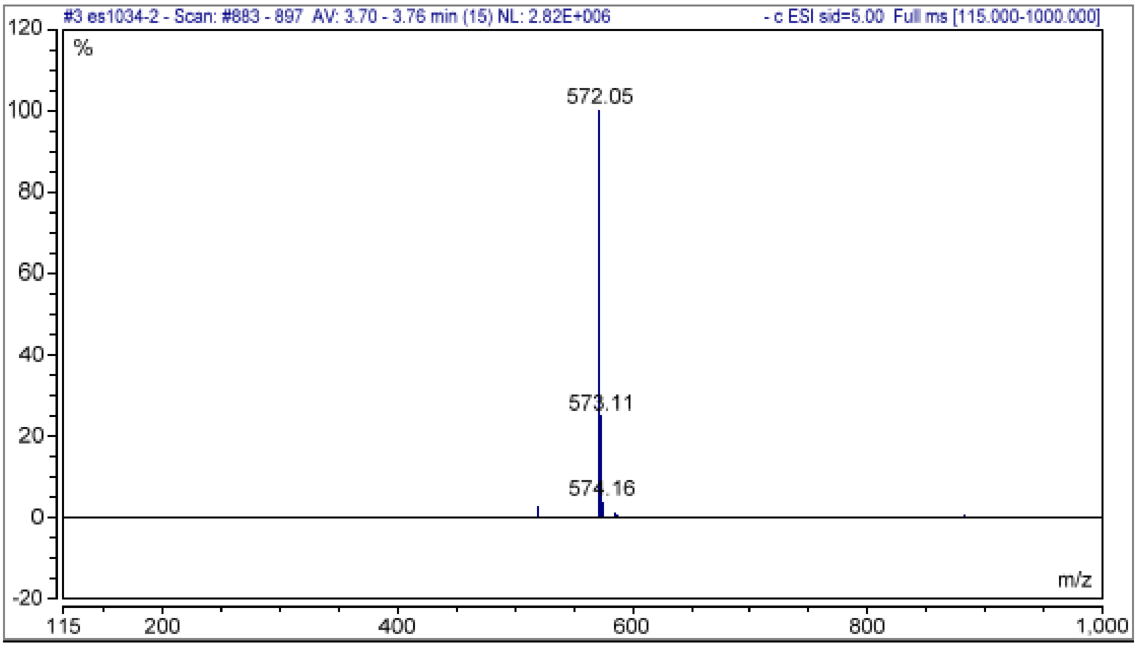

Compound **13**. LC-MS trace and ^{1}H-NMR (acetone-d_6):

Probe : es884-10

**Lösungsmittel :**	MeCN/H2O		**Aufgabemenge:**	10.0 µl	
**Säule:**	Phenomenex Kinetex C18	2.6 µm	**Länge:** 75 mm	**iO :**	3.0 mm
**Fluß (ml / Min) :**	0.5	**Temperatur :**	25.0		
**Detektor:**	DAD-3000	**Pumpe:**	HPG-3200SD	**Sampler:**	WPS-3000
**Laufmittel:**	**A = Acetonitril 0.1% FA**			**B = Wasser 0.1% FA**	
**Gradient:**	A 20.0 %	B 80.0 % --->	A 100.0 %	B 0.0 %	T = 10 Min.

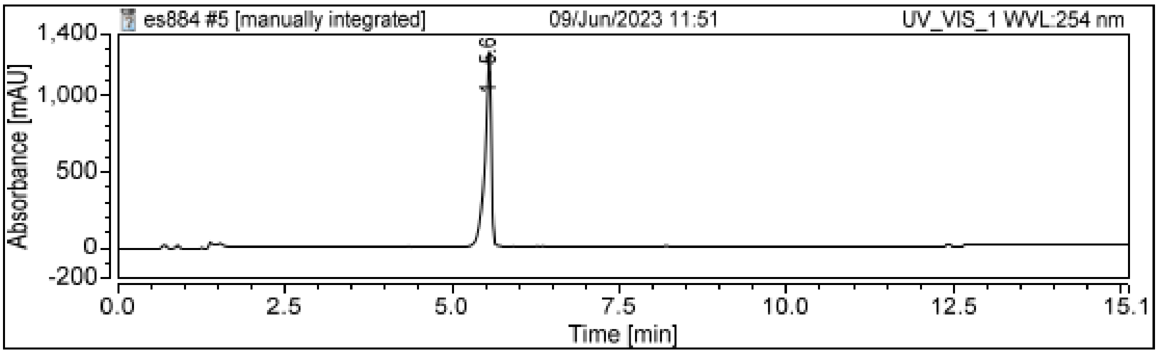

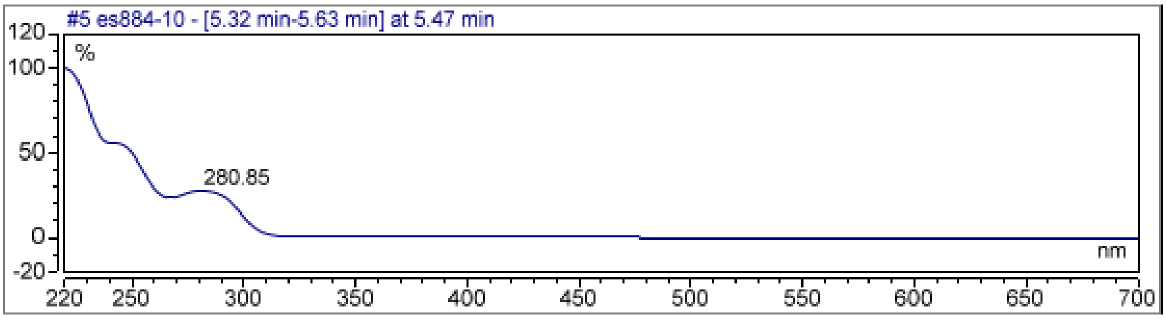

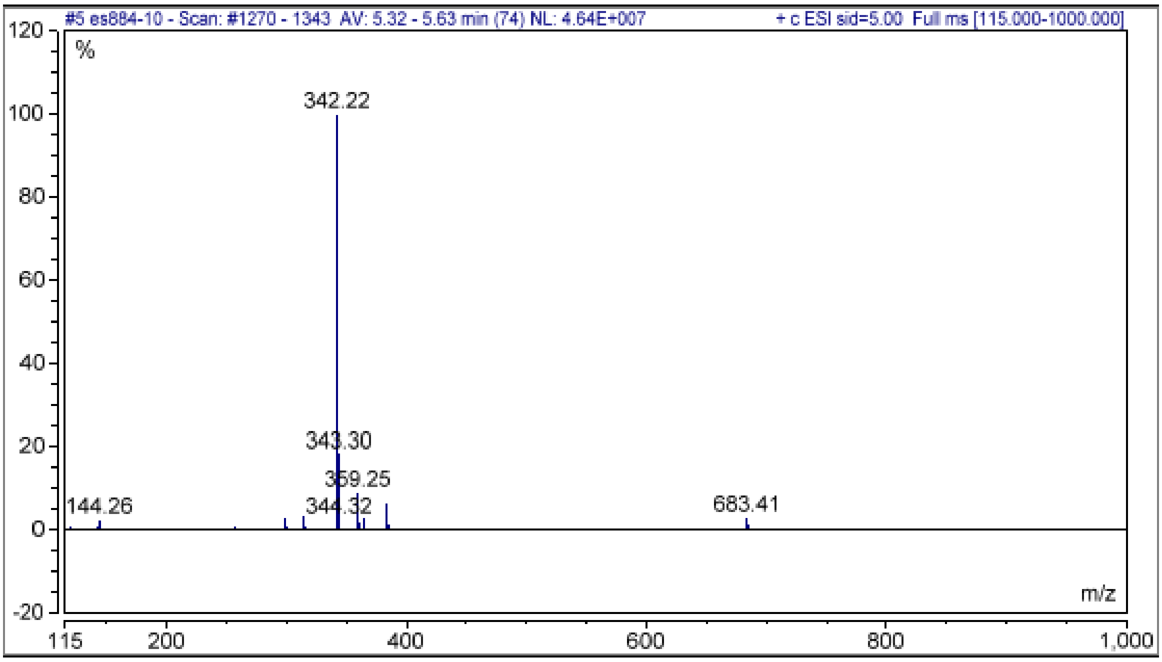

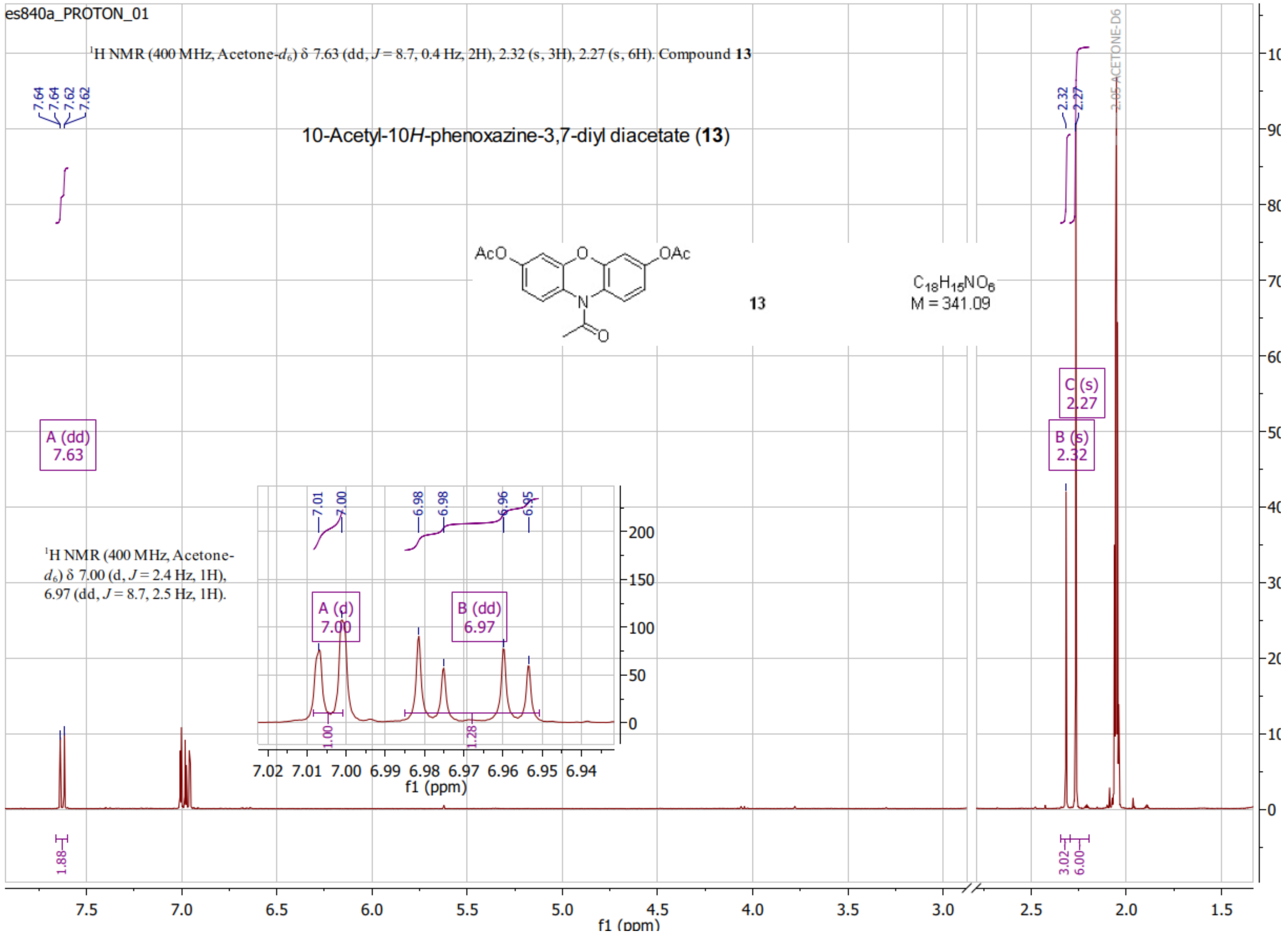

es840a_PROTON_01
¹H NMR (400 MHz, Acetone-d₆) δ 7.63 (dd, J = 8.7, 0.4 Hz, 2H), 2.32 (s, 3H), 2.27 (s, 6H). Compound 13
10-Acetyl-10H-phenoxazine-3,7-diyl diacetate (13)
AcO
OAc
O
N
O
13
C18H15NO6
M = 341.09
A (dd) 7.63
B (s) 2.32
C (s) 2.27
2.05 ACETONE-D6
¹H NMR (400 MHz, Acetone-d₆) δ 7.00 (d, J = 2.4 Hz, 1H), 6.97 (dd, J = 8.7, 2.5 Hz, 1H).
A (d) 7.00
B (dd) 6.97
f1 (ppm)

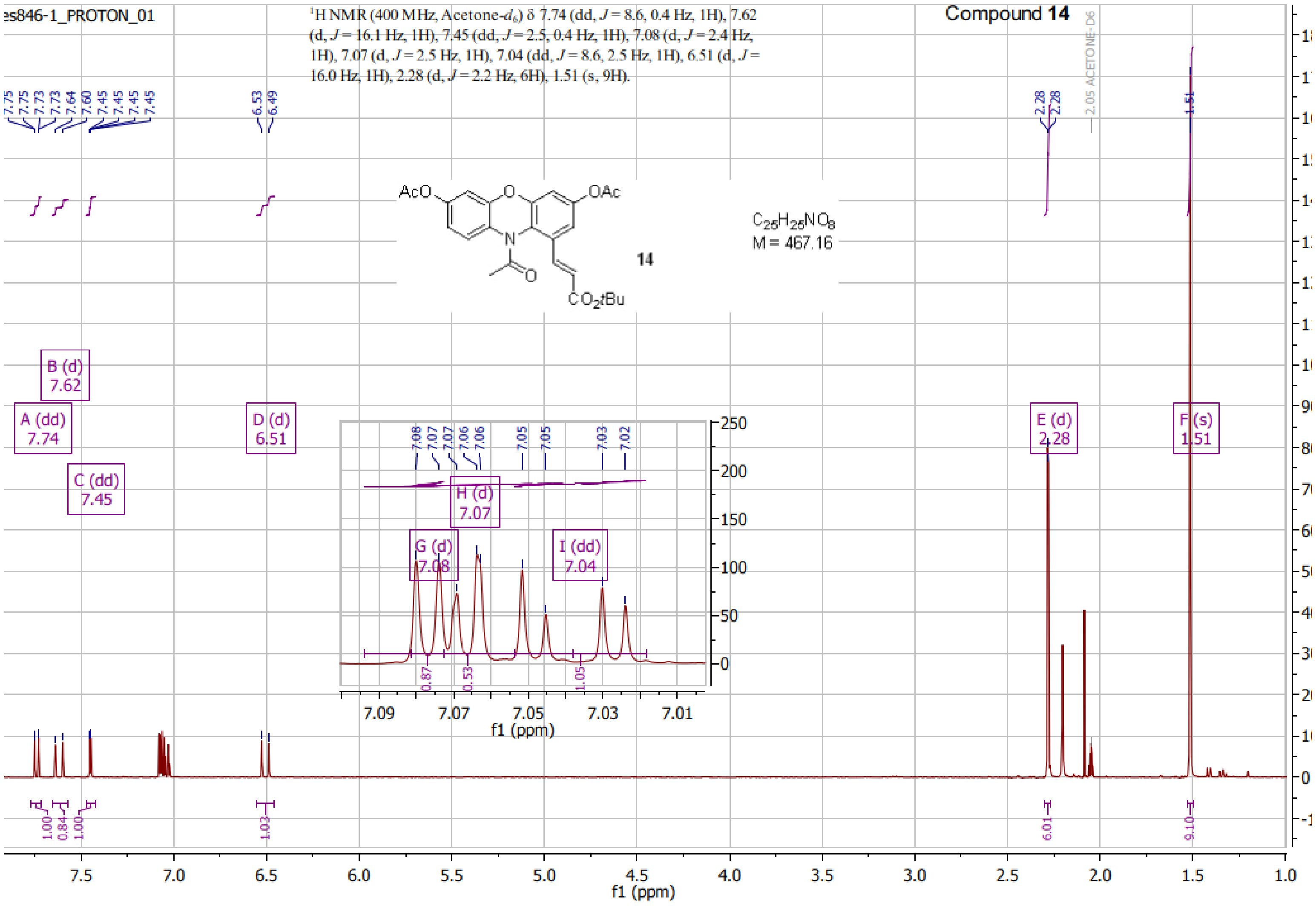

Compound **14**. ^{1}H- and ^{13}C-NMR spectra (acetrone-d$_6$), HPLC trace.

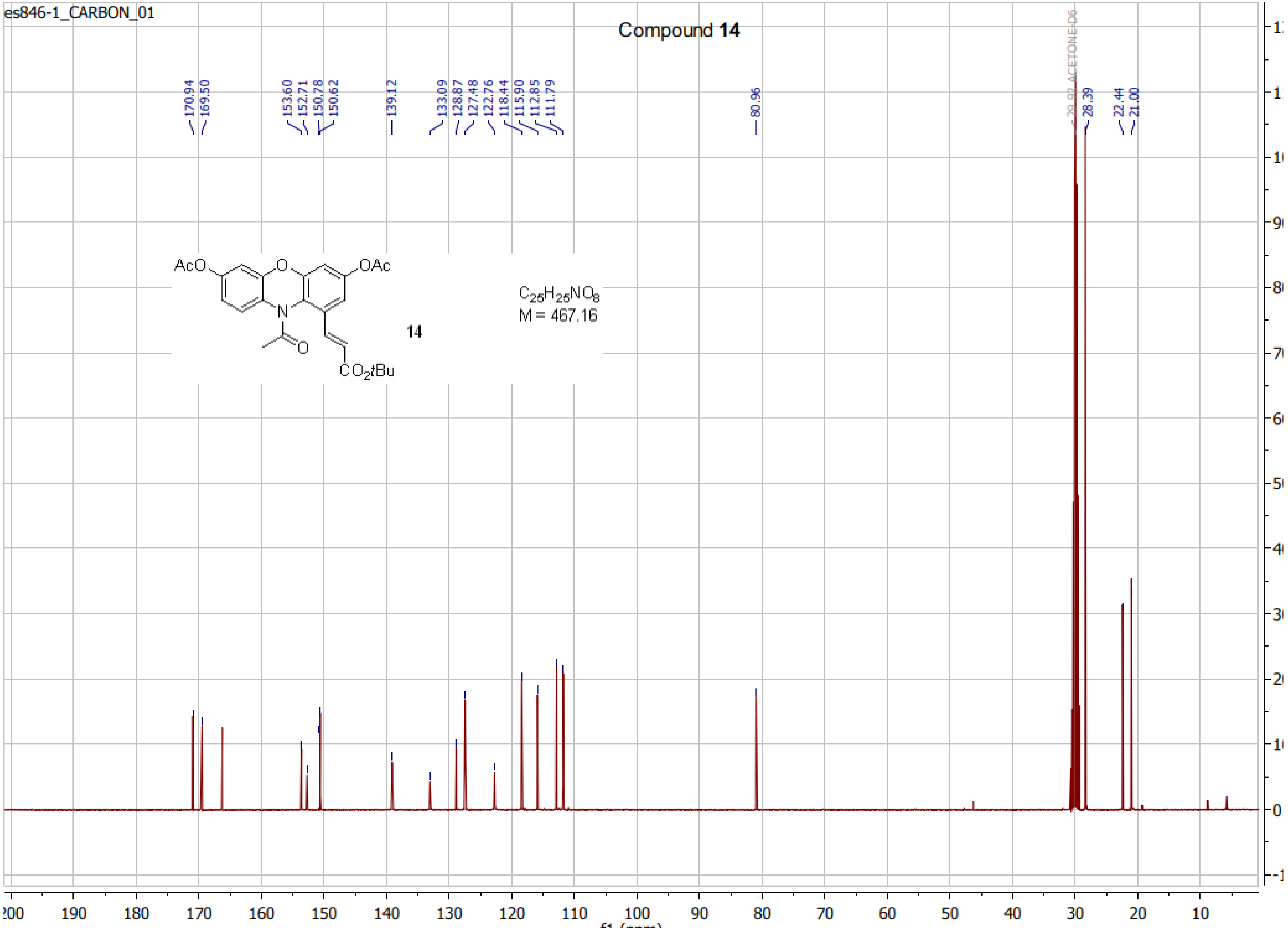
es846-1_CARBON_01
Compound **14**
170.94
169.50
153.60
152.71
150.78
150.62
139.12
133.09
128.87
127.48
122.76
118.44
115.90
112.85
111.79
80.96
29.92 ACETONE-D6
28.39
22.44
21.00
AcO
OAc
O
N
O
CO2tBu
14
C25H25NO8
M = 467.16
200
190
180
170
160
150
140
130
120
110
100
90
80
70
60
50
40
30
20
10
0

Probe : es846

**Lösungsmittel :**	**MeCN/H2O**		**Aufgabemenge:**		**3.0 µl**		
**Säule:**	Phenomenex	2.6 µm	**Länge:**	75 mm	**iO :**	3.0 mm	
**Fluß (ml / Min) :**	**0.5**	**Temperatur :**		**25.0**			
**Detektor:**	DAD-3000	**Pumpe:**	HPG-3200SD		**Sampler:**	WPS-3000	
**Laufmittel:**	**A = Acetonitril 0.1% FA**				**B = Wasser 0.1% FA**		
**Gradient:**	A 20.0 %	B 80.0 %	---->	A 100.0 %	B 0.0 %	T = 10 Min.	

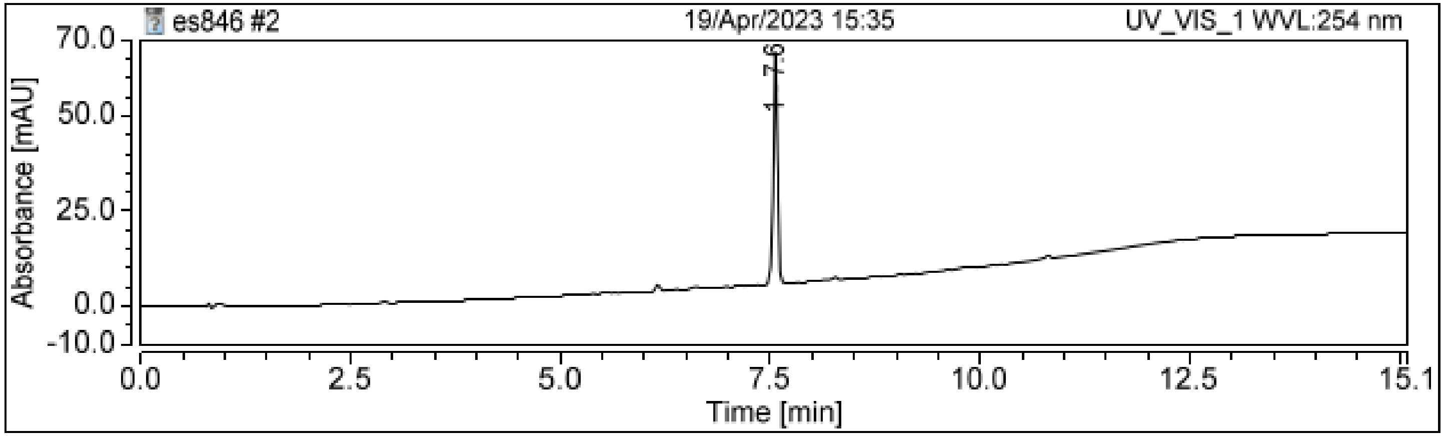

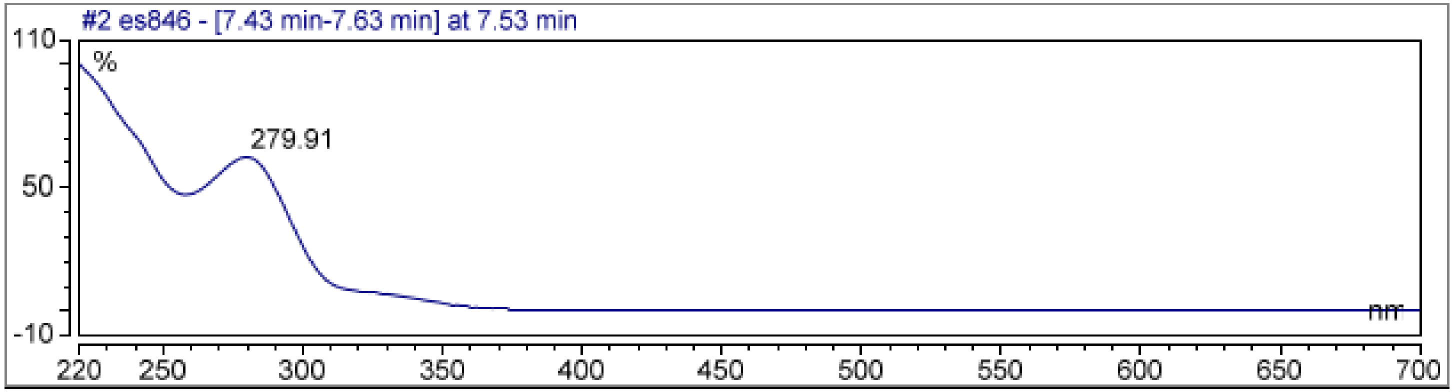

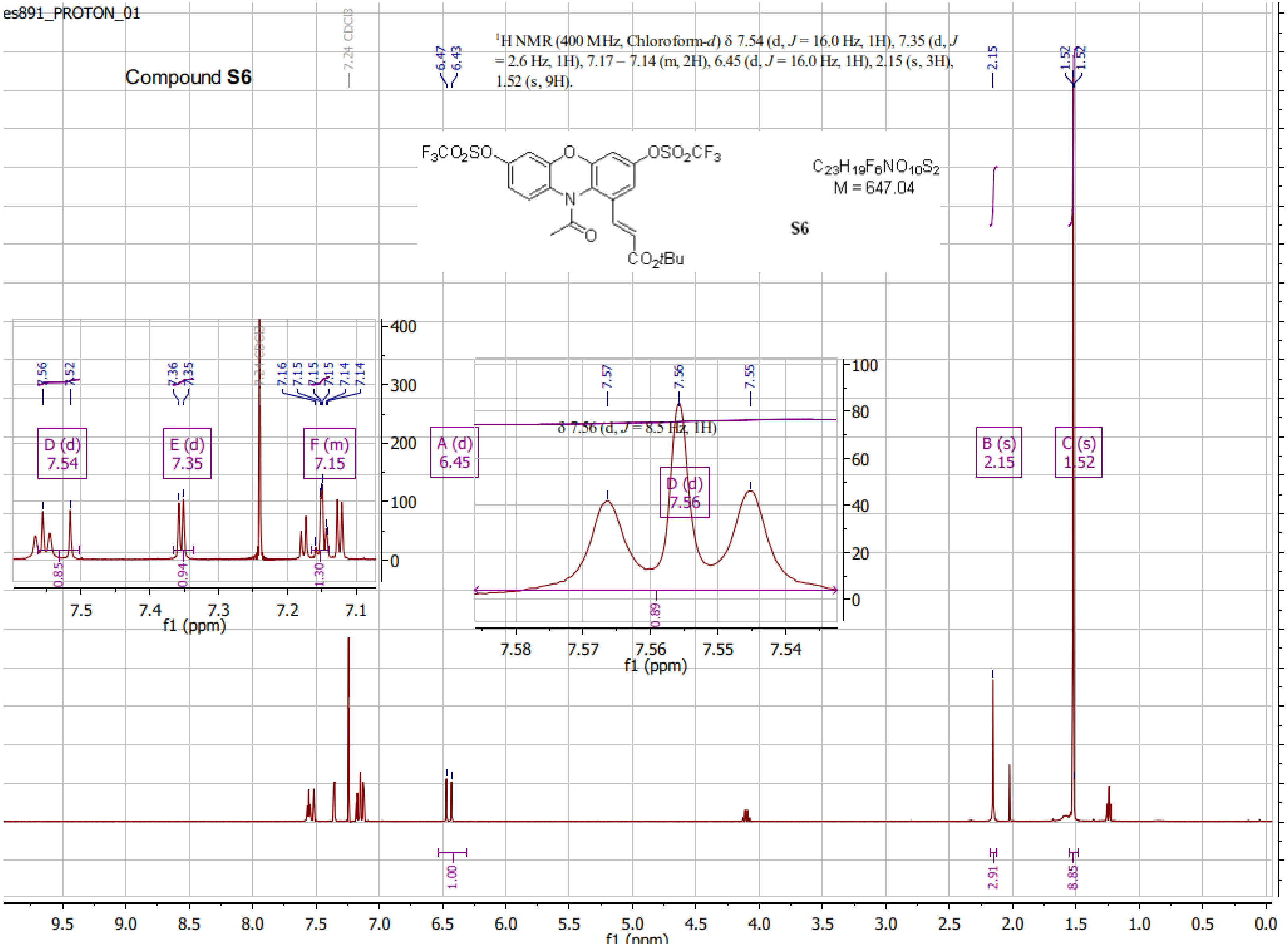

Bis-triflate **S6**. ^{1}H-and ^{19}F-NMR spectra (CDCl3).

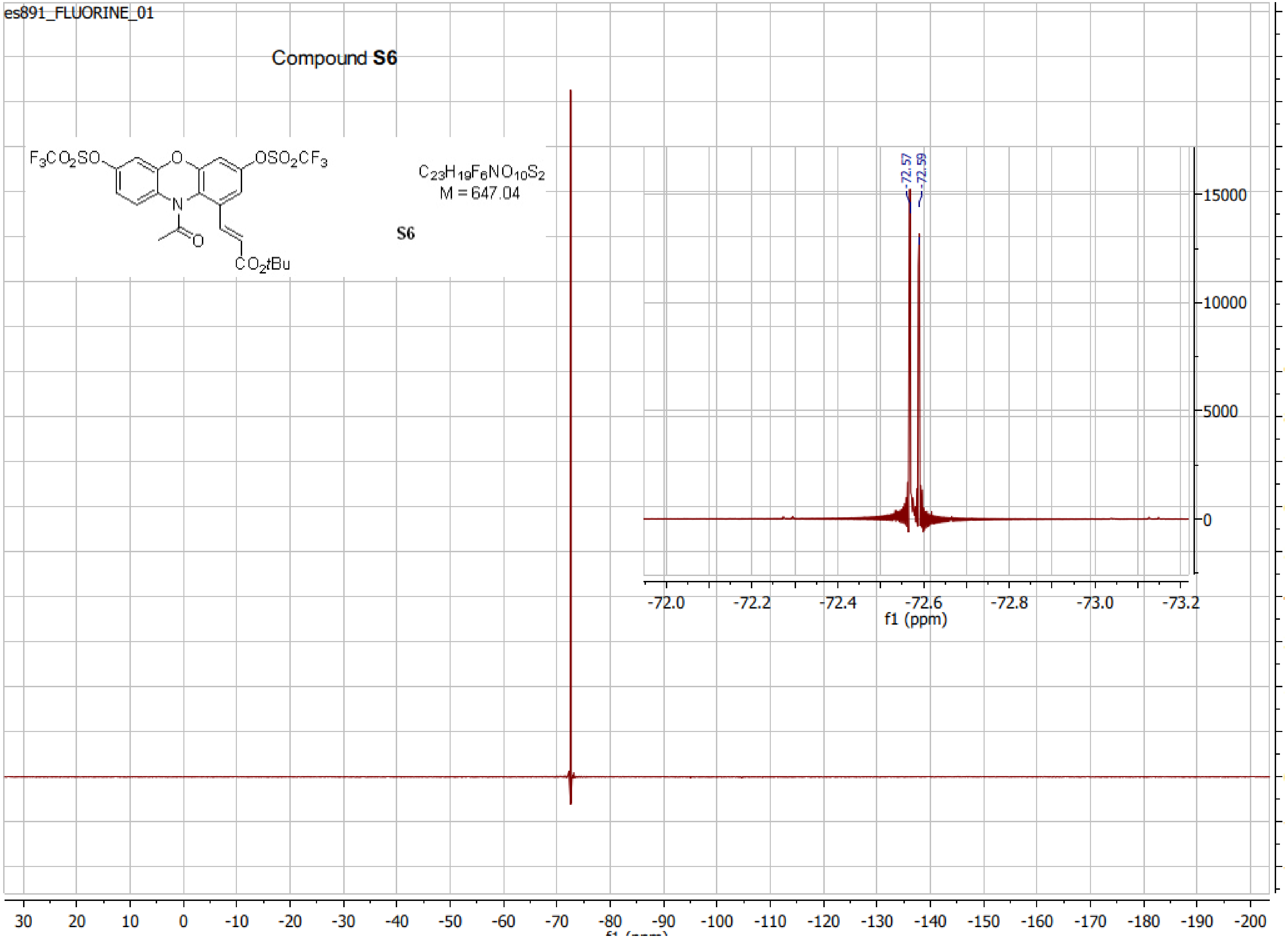

es891_FLUORINE_01
Compound S6
F3CO2SO
OSO2CF3
O
N
O
CO2tBu
C23H19F6NO10S2
M = 647.04
S6
-72.57
-72.59
15000
10000
5000
0
-72.0
-72.2
-72.4
-72.6
-72.8
-73.0
-73.2
f1 (ppm)
30
20
10
0
-10
-20
-30
-40
-50
-60
-70
-80
-90
-100
-110
-120
-130
-140
-150
-160
-170
-180
-190
-200

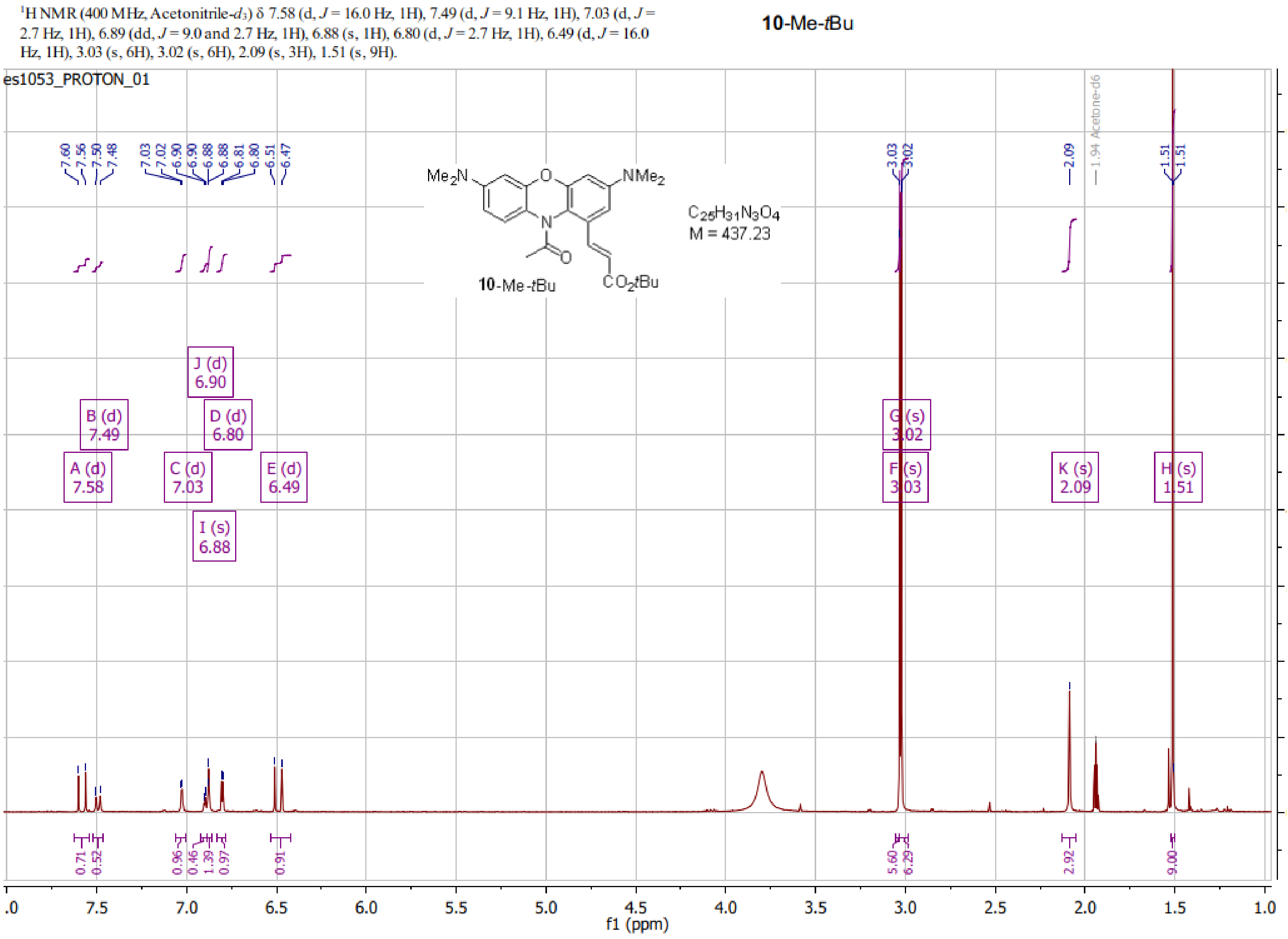

tert-Butyl ester **10**-Me-*t*Bu. NMR spectra (CD_3CN).

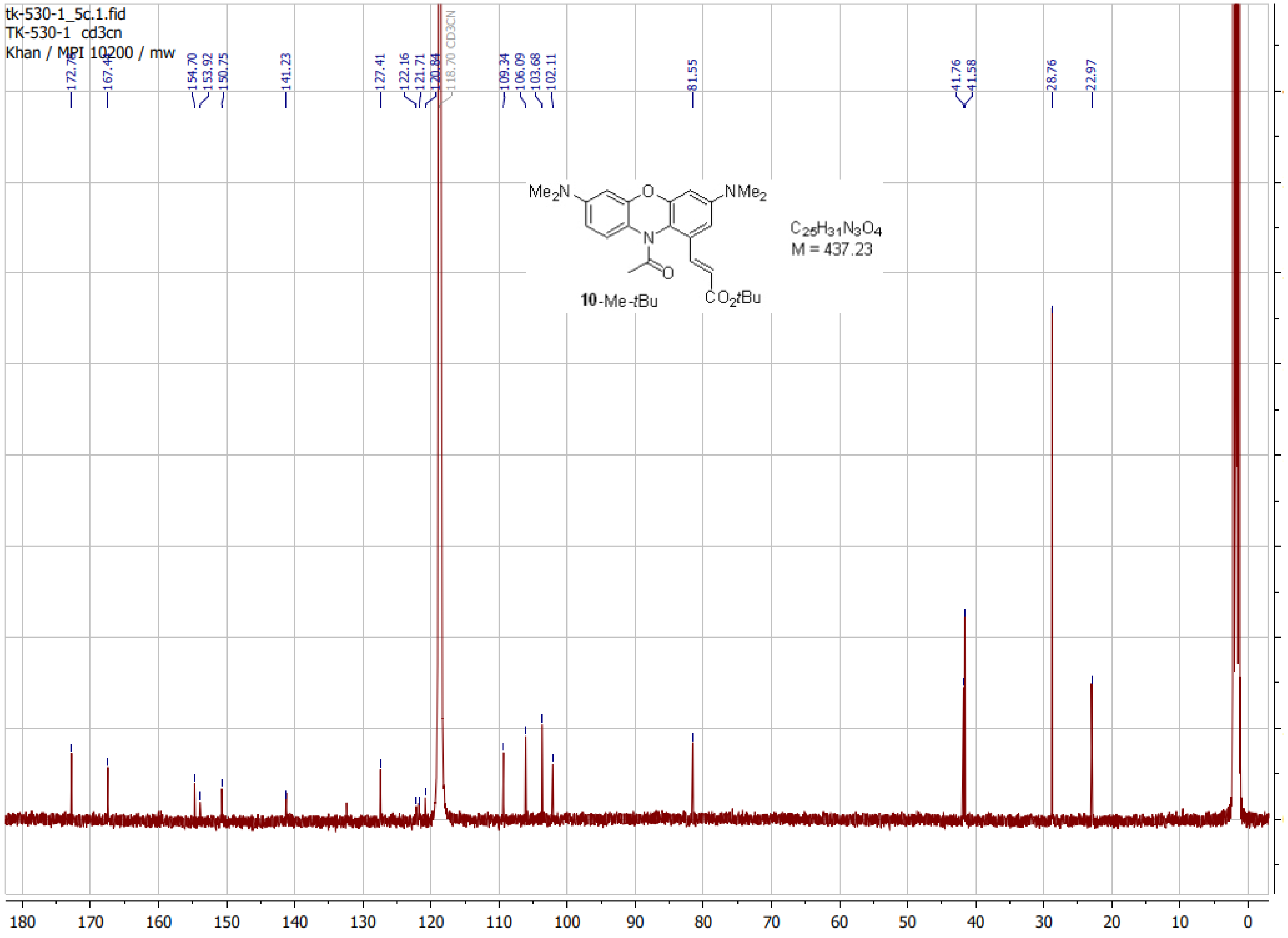
tk-530-1_5c.1.fid
TK-530-1 cd3cn
Khan / MPI 10200 / mw
172.76
167.44
154.70
153.92
150.75
141.23
127.41
122.16
121.71
120.84
118.70 CD3CN
109.34
106.09
103.68
102.11
81.55
41.76
41.58
28.76
22.97
Me2N
NMe2
O
N
O
CO2tBu
10-Me-tBu
C25H31N3O4
M = 437.23
180
170
160
150
140
130
120
110
100
90
80
70
60
50
40
30
20
10
0

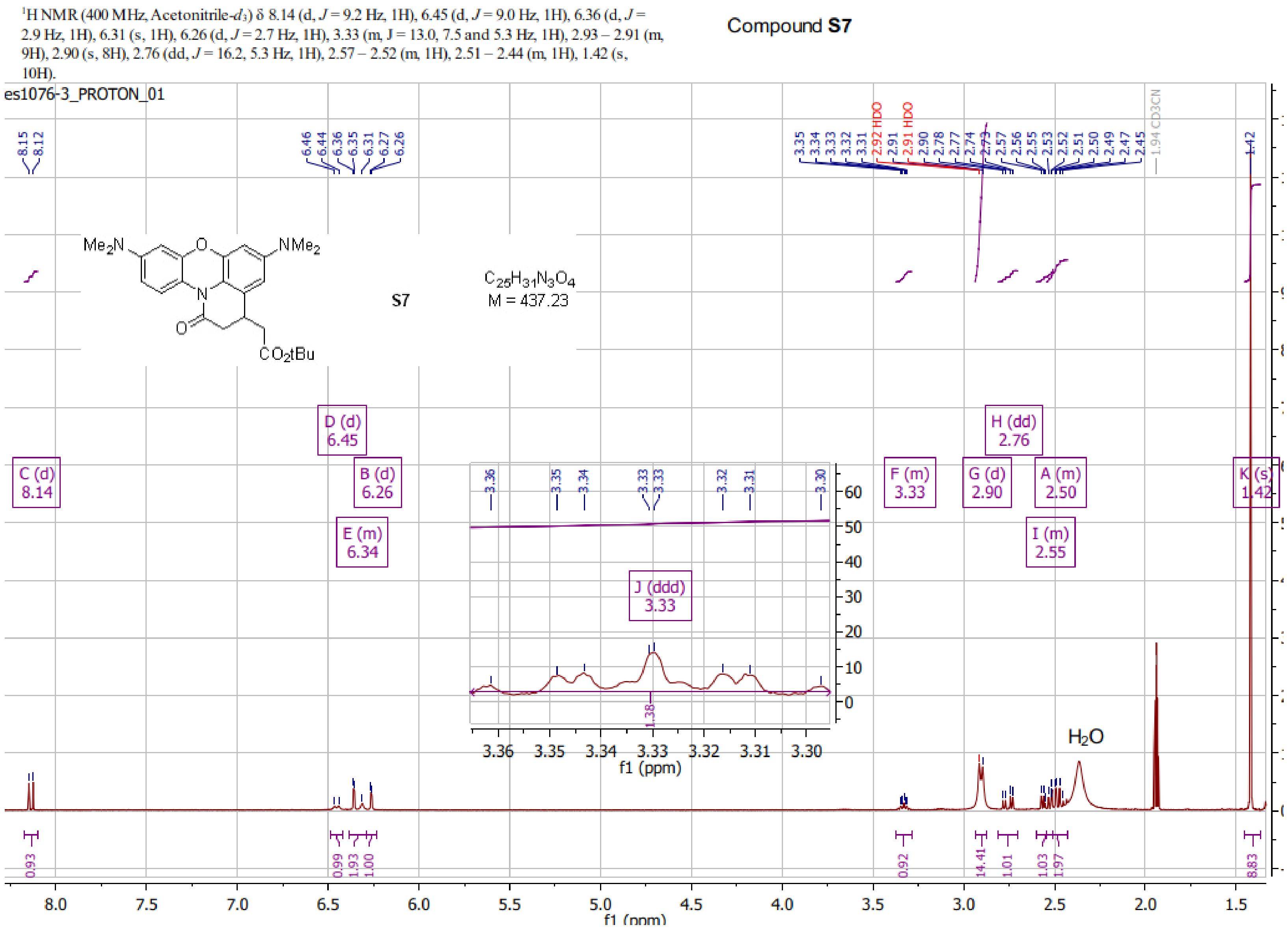

Compound **S7**. ¹H- ¹H-¹H-COSY and ¹³C-¹H-corr. (gHSQCAD) NMR spectra (CD_3CN).

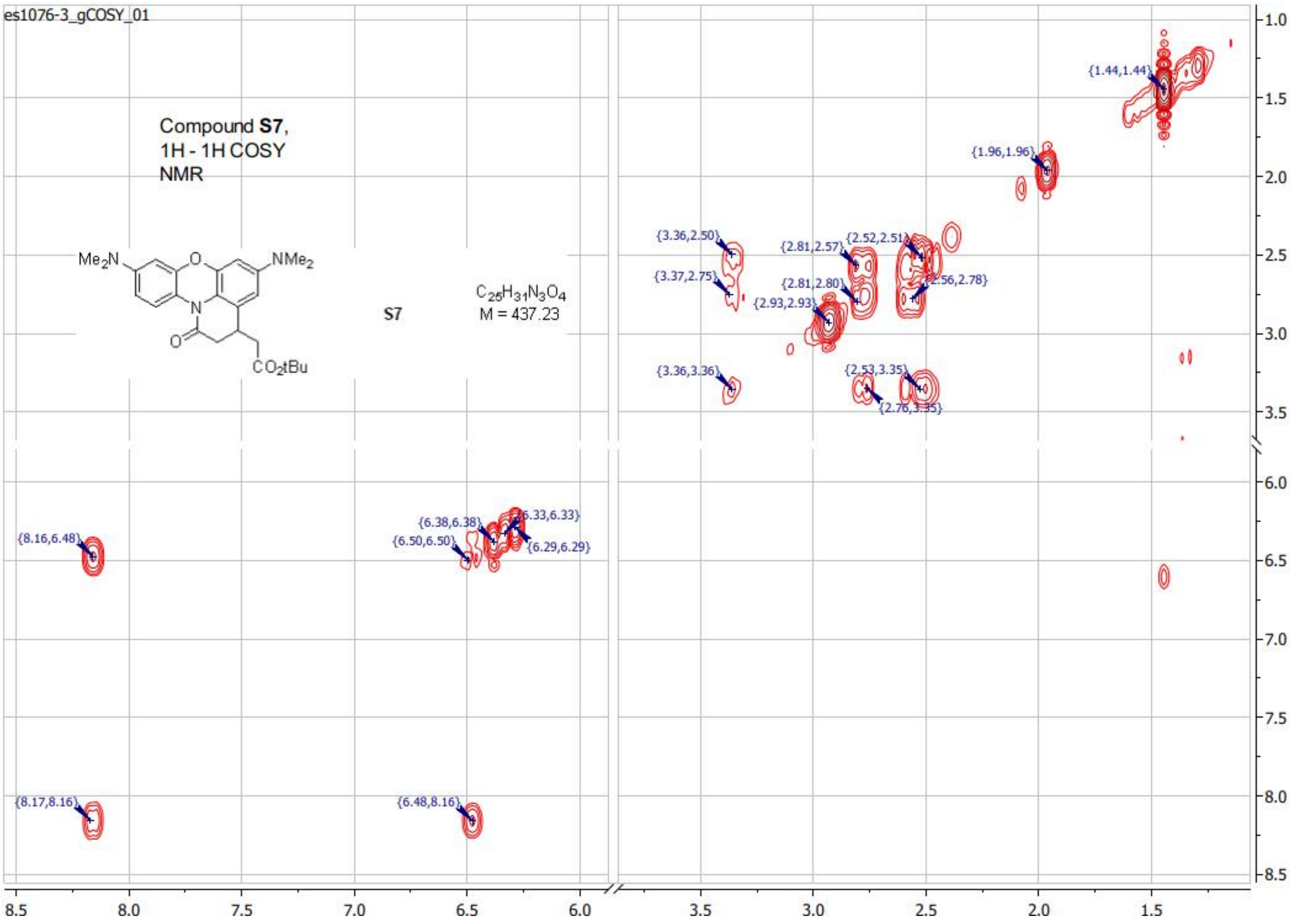
es1076-3_gCOSY_01
Compound S7,
1H - 1H COSY
NMR
Me2N
NMe2
O
N
CO2tBu
S7
C25H31N3O4
M = 437.23
{1.44,1.44}
{1.96,1.96}
{3.36,2.50}
{2.81,2.57}
{2.52,2.51}
{3.37,2.75}
{2.81,2.80}
{2.56,2.78}
{2.93,2.93}
{3.36,3.36}
{2.53,3.35}
{2.76,3.35}
{6.38,6.38}
{6.33,6.33}
{8.16,6.48}
{6.50,6.50}
{6.29,6.29}
{8.17,8.16}
{6.48,8.16}

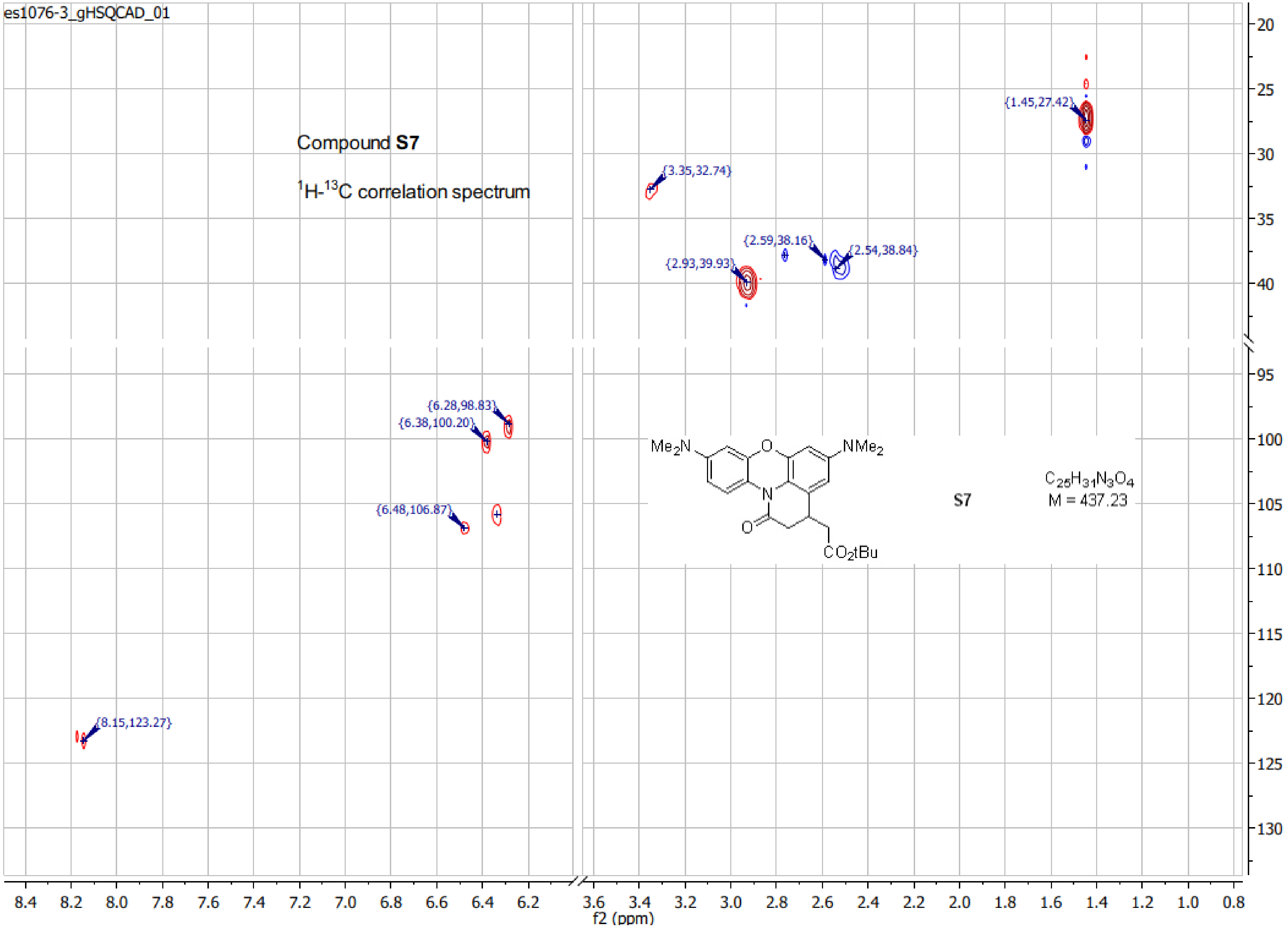

es1076-3_gHSQCAD_01
Compound **S7**
^{1}H-^{13}C correlation spectrum
{1.45,27.42}
{3.35,32.74}
{2.59,38.16}
{2.54,38.84}
{2.93,39.93}
{6.28,98.83}
{6.38,100.20}
{6.48,106.87}
{8.15,123.27}
Me_2N
O
NMe_2
N
O
CO_2tBu
S7
$C_{25}H_{31}N_3O_4$
M = 437.23
f2 (ppm)

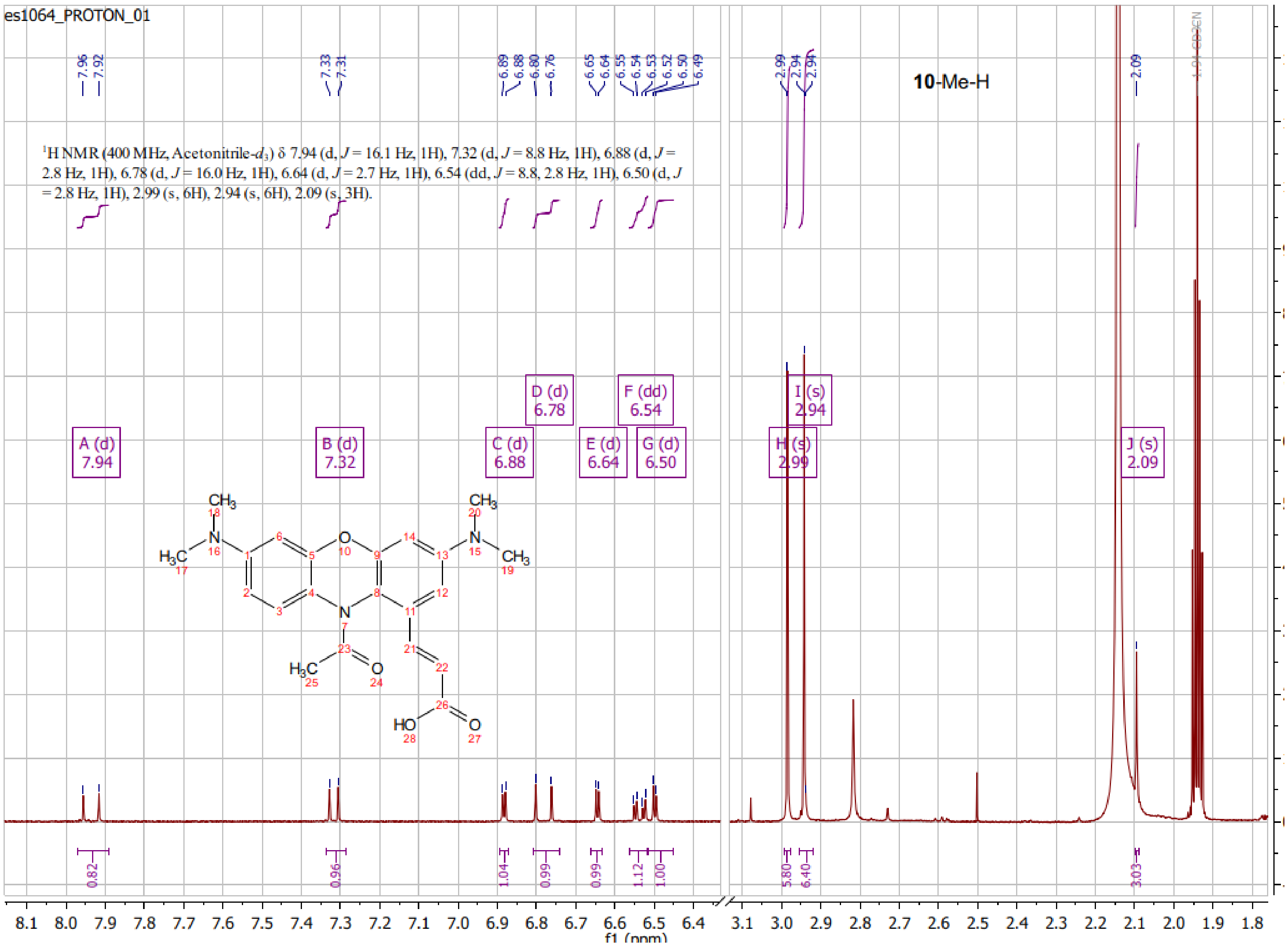

Compound **10**-Me-H: ^{1}H-NMR spectrum (400 MHz, CD_3CN), LC-MS trace and ^{13}C-NMR spectrum (125 MHz, CD_3CN).

Probe : es1060-5

**Lösungsmittel :**	MeCN/H2O		**Aufgabemenge:**	0.5 µl	
**Säule:**	Phenomenex Kinetex C18	1.7 µm	**Länge:** 50 mm	**iO :**	2.1 mm
**Fluß (ml / Min) :**	0.5	**Temperatur :**	25.0		
**Detektor:**	DAD-3000	**Pumpe:**	HPG-3200SD	**Sampler:**	WPS-3000
**Laufmittel:**	**A = Acetonitril 0.1% FA**			**B = Wasser 0.1% FA**	
**Gradient:**	A 20.0 %	B 80.0 % ---->	A 100.0 %	B 0.0 %	T = 4 Min.

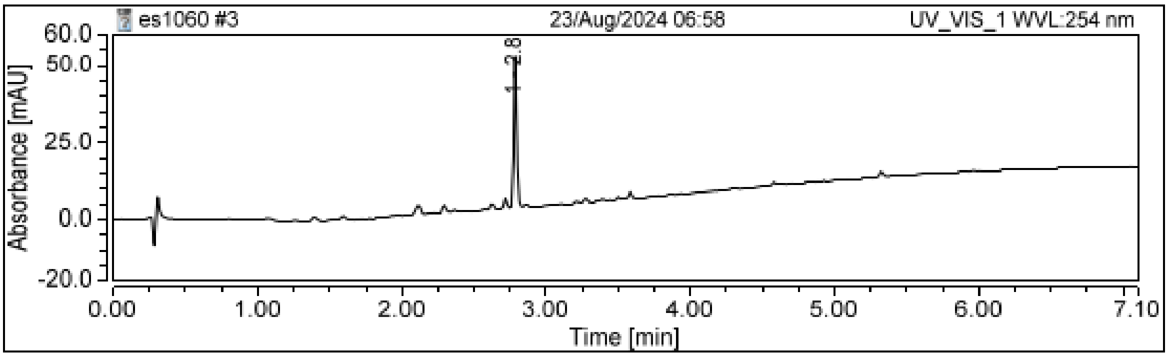

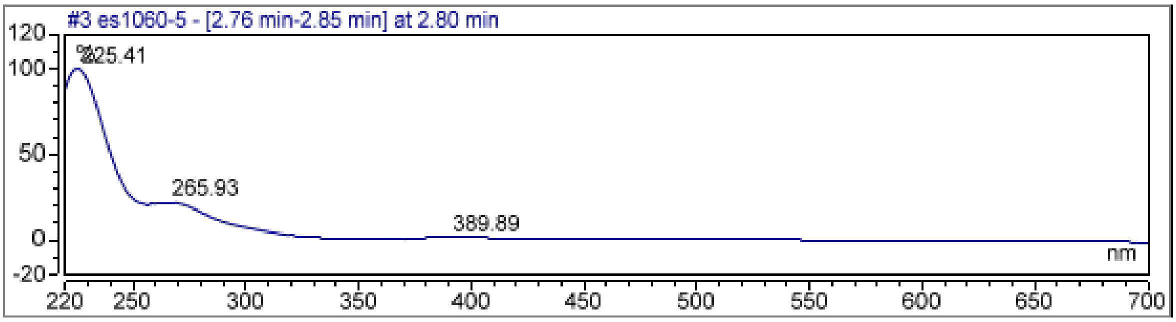

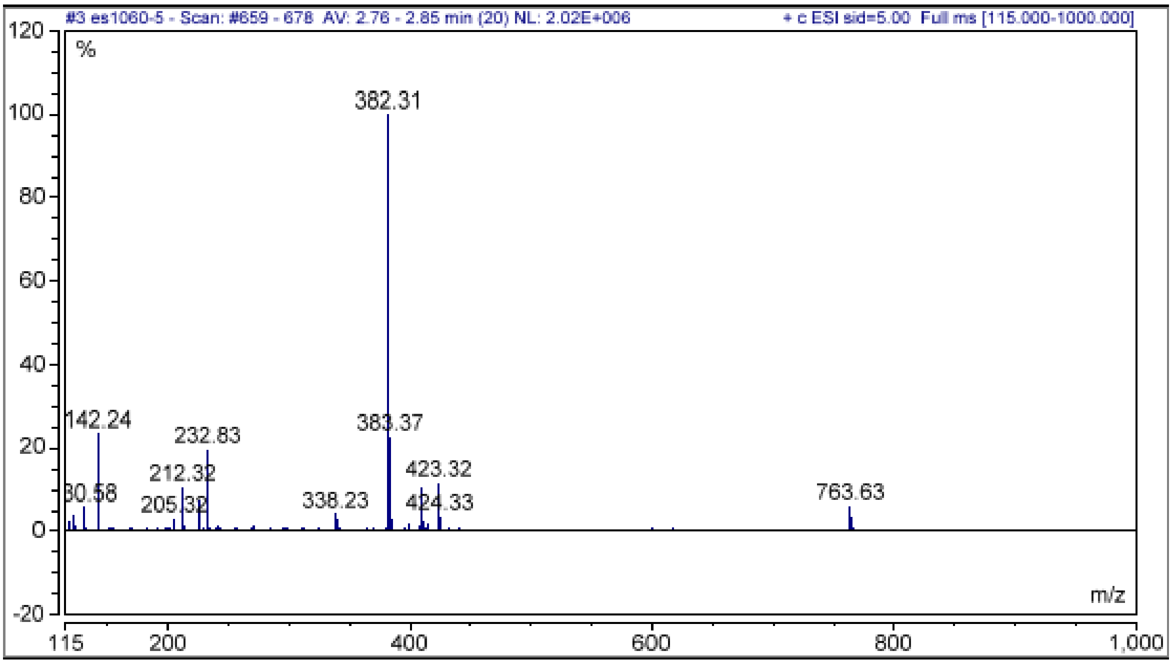

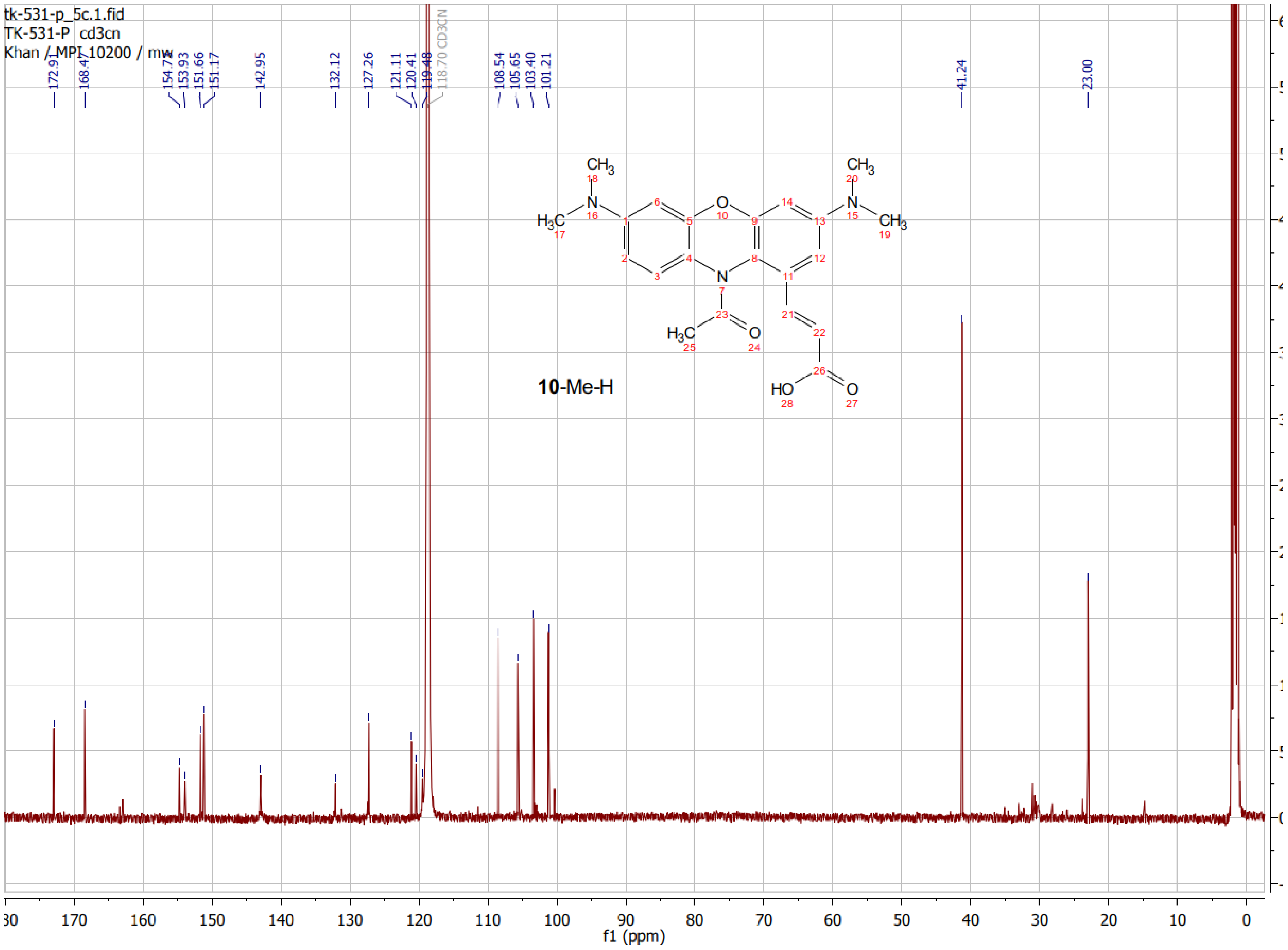
tk-531-p_5c.1.fid
TK-531-P cd3cn
Khan / MPI 10200 / mw
172.91
168.47
154.74
153.93
151.66
151.17
142.95
132.12
127.26
121.11
120.41
119.48
118.70 CD3CN
108.54
105.65
103.40
101.21
41.24
23.00
10-Me-H
f1 (ppm)

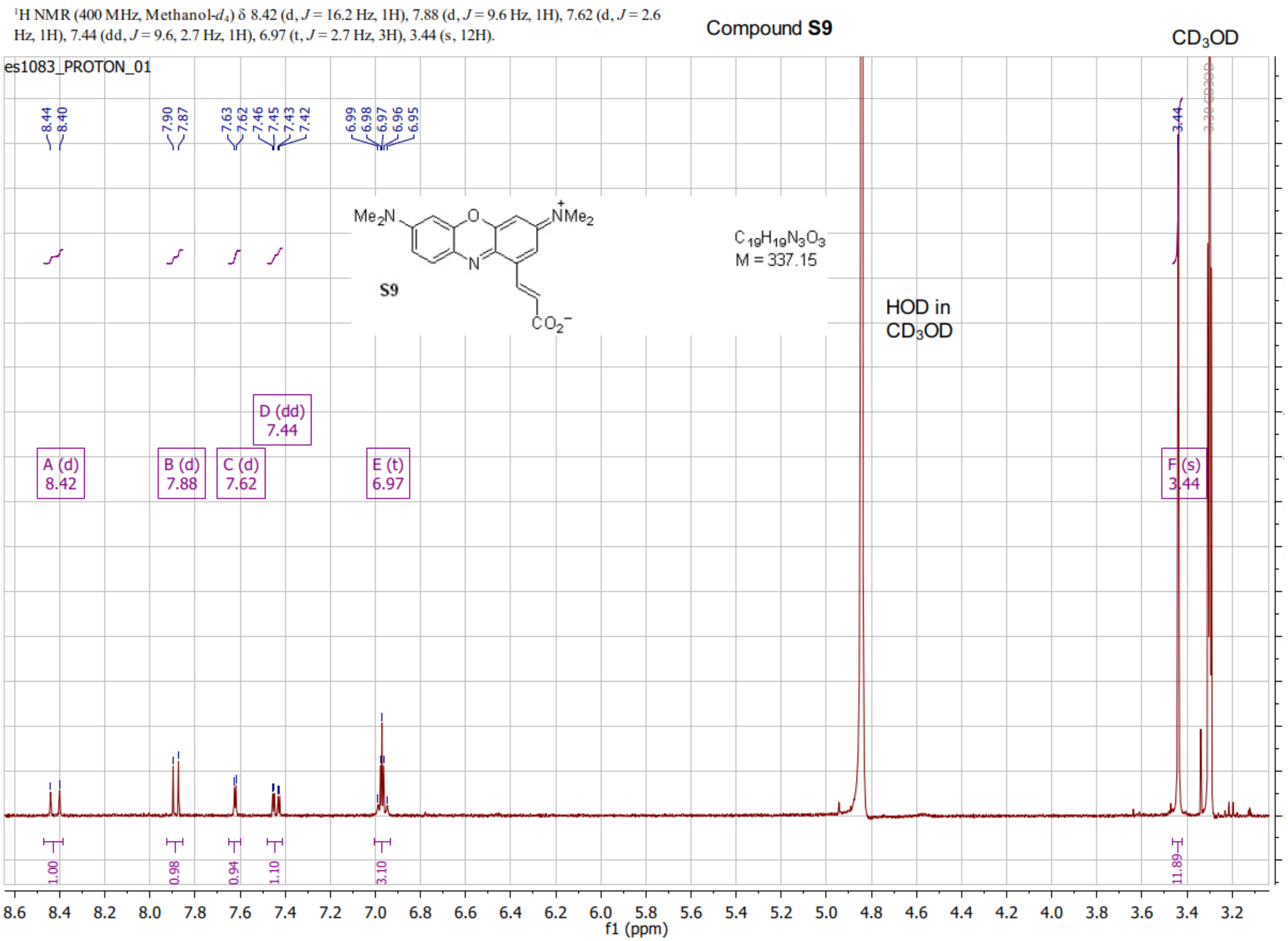

Compound **S9**. ¹H-NMR spectrum (CD_3OD).

^{1}H NMR (500 MHz, Acetonitrile-d_3) δ 8.27 (d, J = 16.5 Hz, 1H), 7.82 (d, J = 9.7 Hz, 1H), 7.48 (d, J = 2.6 Hz, 1H), 7.32 (dd, J = 9.6, 2.7 Hz, 1H), 7.20 (d, J = 16.5 Hz, 1H), 6.82 (dd, J = 5.6, 2.7 Hz, 2H), 3.72 (ddt, J = 14.0, 10.3, 4.7 Hz, 9H), 2.43 (s, 3H), 1.31 (t, J = 7.2 Hz, 12H). Compound **24**.

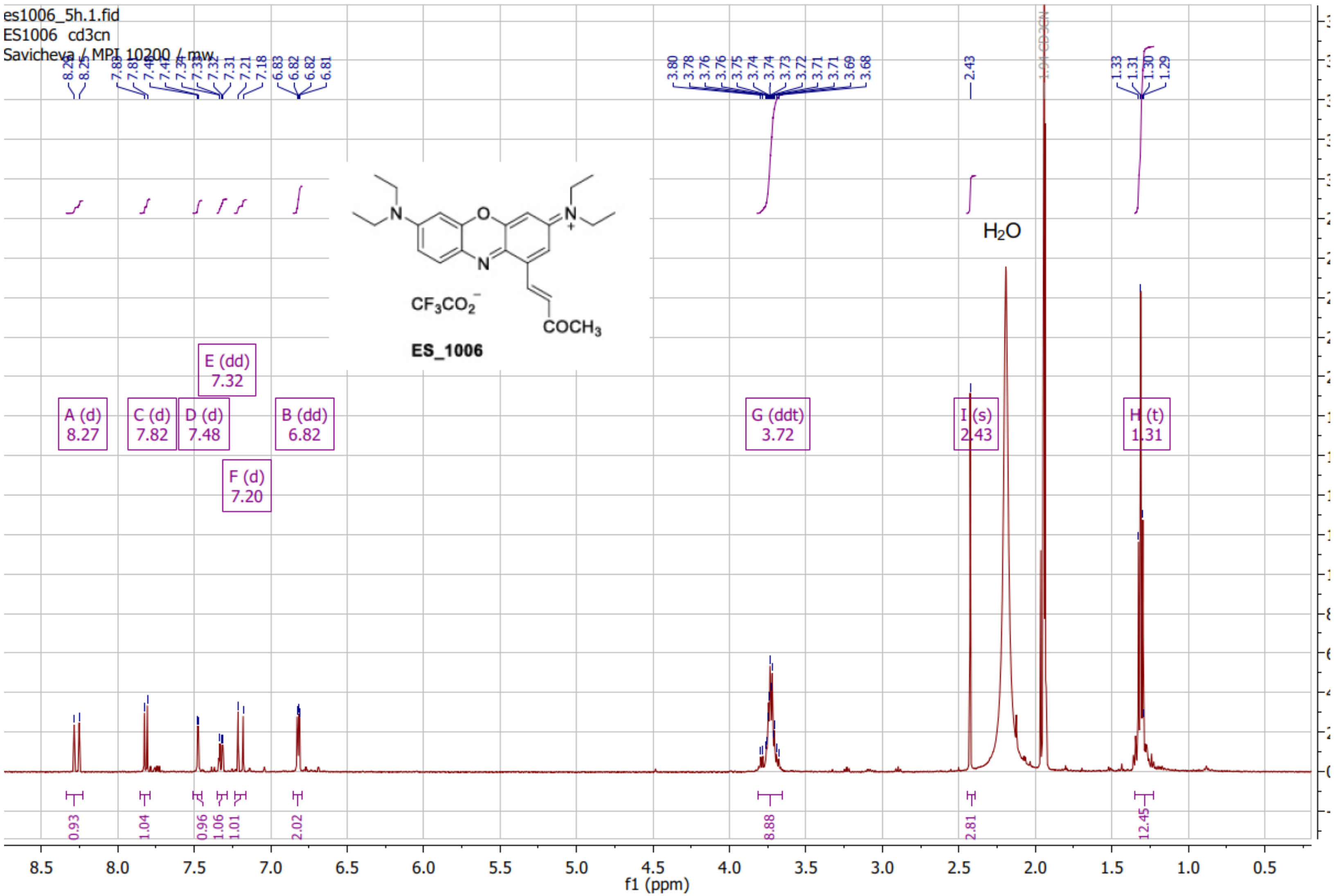

Ketone **ES-1006** – photolysis product of acrylic acid **2**-H formed in aq. buffer (for mechanism and structure, see Scheme **4** of the main text). ^{1}H- and ^{13}C-NMR spectra in CD_3CN.

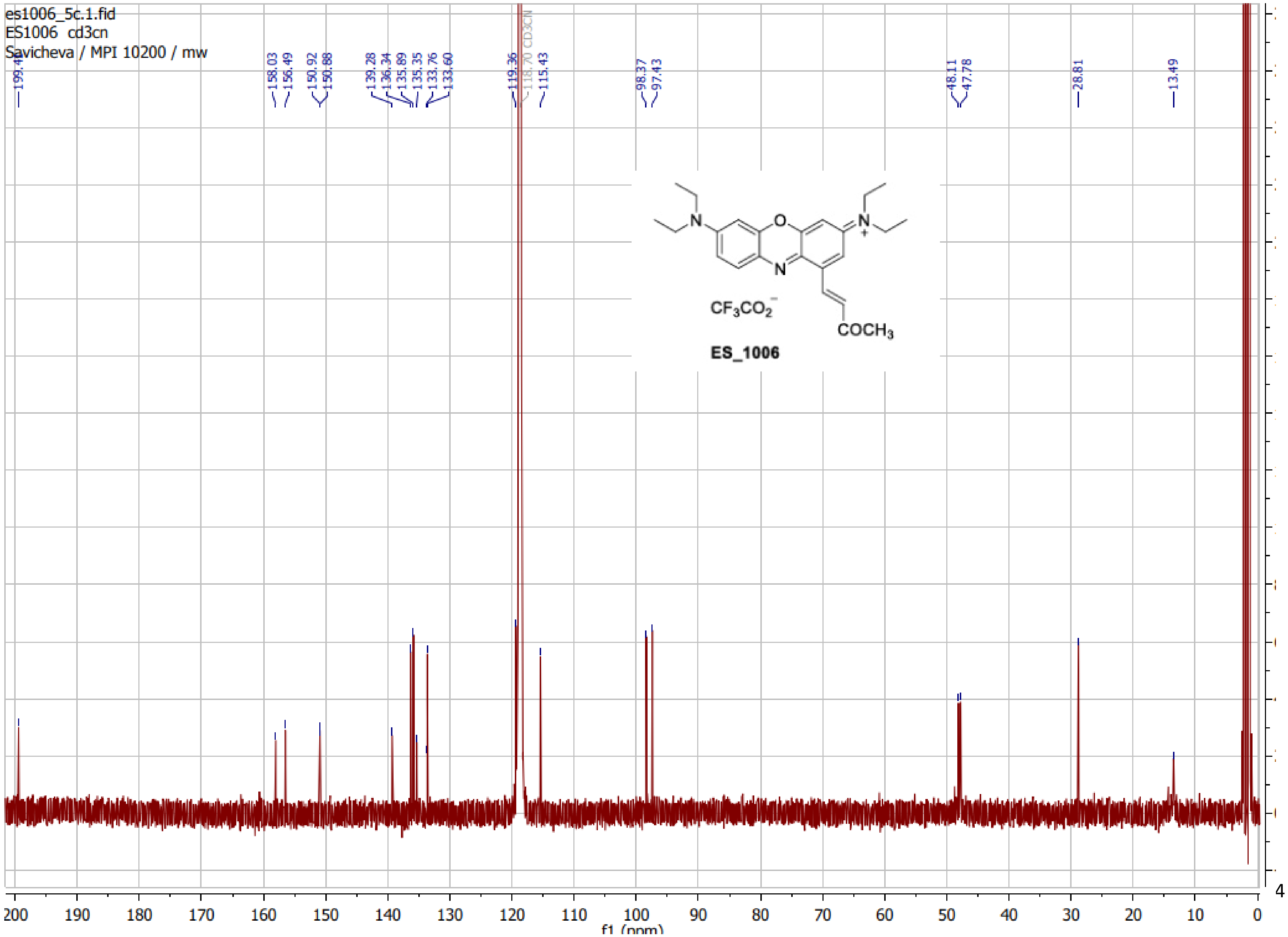
es1006_5c.1.fid
ES1006 cd3cn
Savicheva / MPI 10200 / mw
199.4
158.03
156.49
150.92
150.88
139.28
136.34
135.89
135.35
133.76
133.60
119.36
118.70 CD3CN
115.43
98.37
97.43
48.11
47.78
28.81
13.49
CF3CO2
COCH3
ES_1006
200 190 180 170 160 150 140 130 120 110 100 90 80 70 60 50 40 30 20 10 0
f1 (ppm)

Amide **10-H-NH(CH_2)$_2$OMe** (see Scheme 7 in the main text). LC-MS trace, ^{1}H-NMR spectrum (CD_3CN).

Probe : es1081 rein

Lösungsmittel : MeCN/H2O **Aufgabemenge:** 1.0 µl
Säule: Phenomenex Kinetex C18 1.7 µm **Länge:** 50 mm **iO :** 2.1 mm
Fluß (ml / Min) : 0.5 **Temperatur :** 25.0
Detektor: DAD-3000 **Pumpe:** HPG-3200SD **Sampler:** WPS-3000
Laufmittel: **A = Acetonitril 0.1% FA** **B = Wasser 0.1% FA**
Gradient: A 20.0 % B 80.0 % ---> A 100.0 % B 0.0 % T = 4 Min.

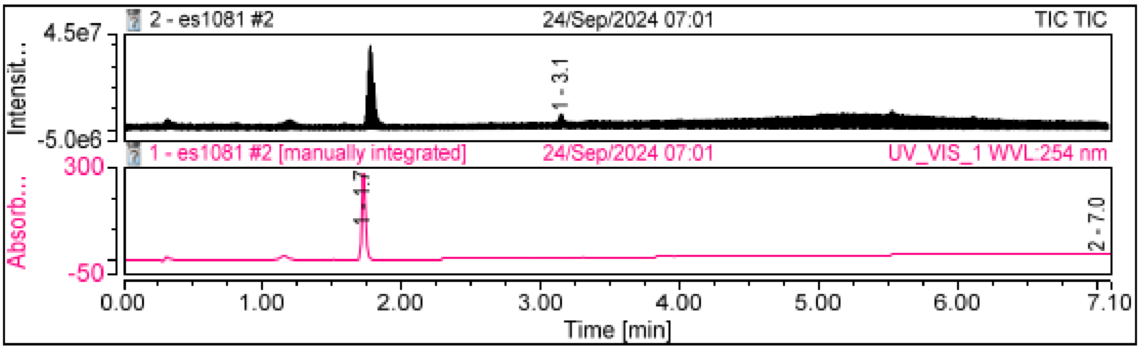

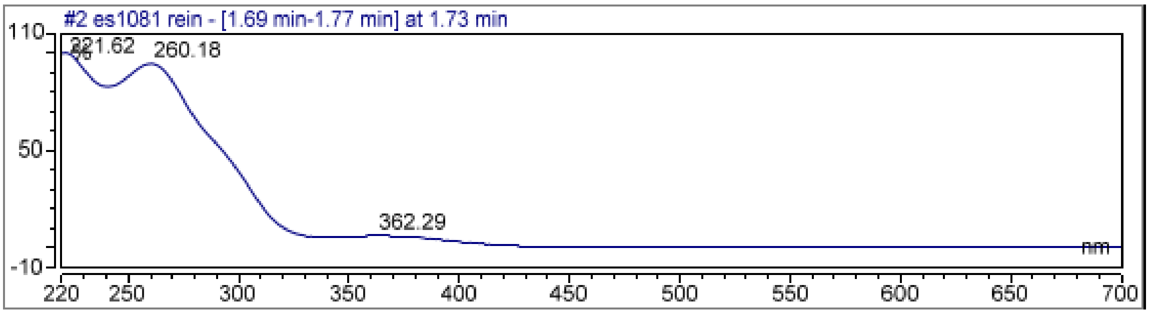

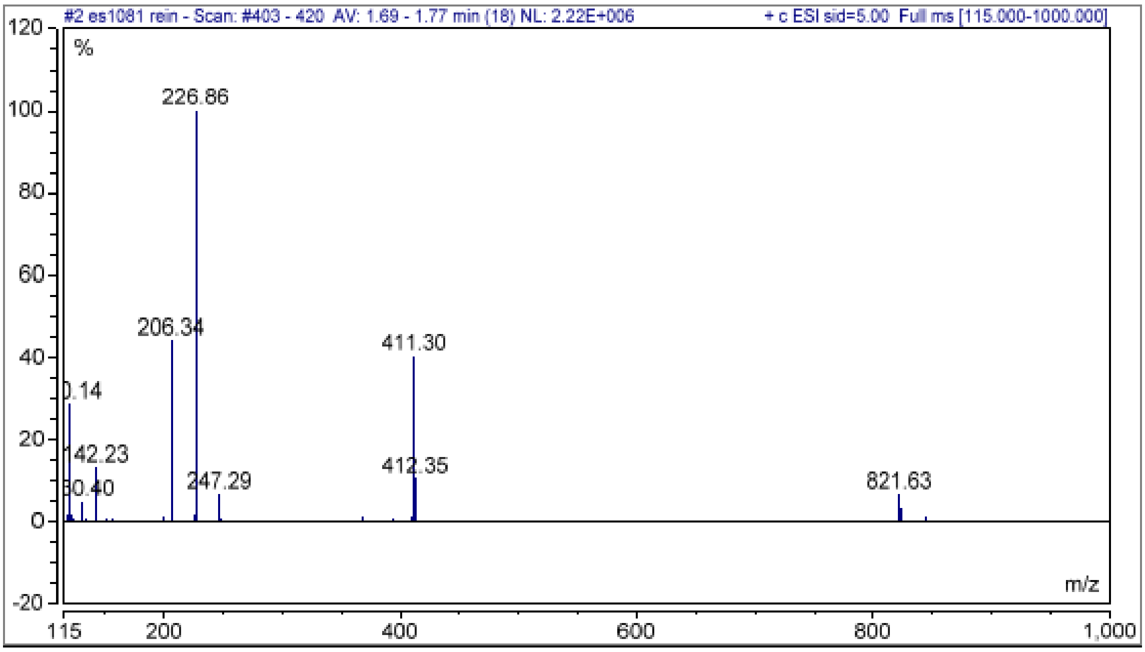

^{1}H NMR (400 MHz, Acetonitrile-d_3) δ 7.45 (d, J = 15.8 Hz, 1H), 7.28 (d, J = 8.5 Hz, 1H), 6.71 (s, 1H), 6.67 (d, J = 2.5 Hz, 1H), 6.53 (d, J = 15.7 Hz, 1H), 6.45 (dd, 8.4 and 2.6 Hz, 1H), 6.44 (d, 2.5 Hz, 1H), 6.42 (d, 2.6 Hz, 1H), 3.49 – 3.41 (m, 4H), 3.32 (s, 3H), 2.79 (s, 3H), 2.77 (s, 3H), 2.04 (s, 3H).

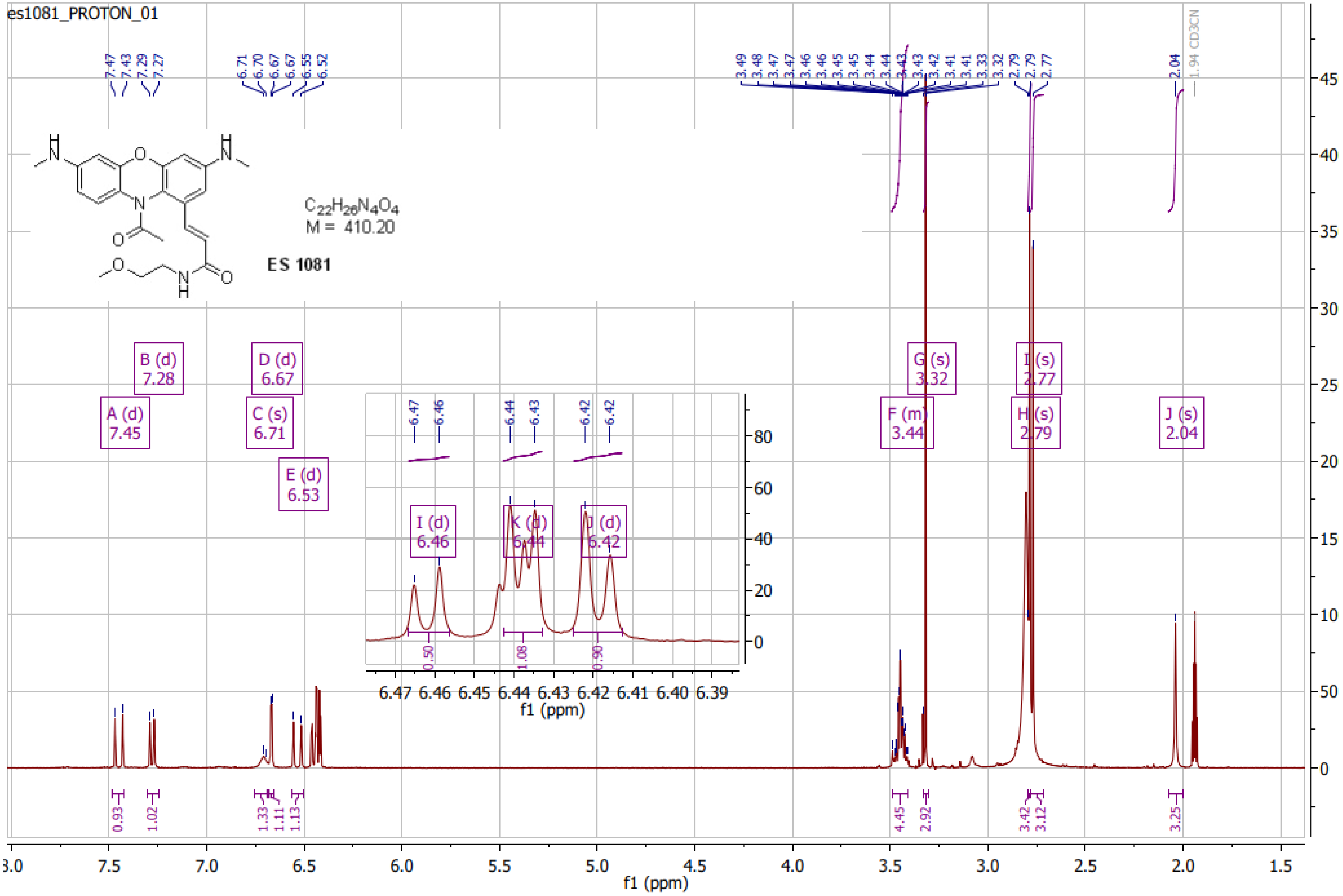

Probe **2-Halo** = **CA3**. ^{1}H-NMR (CD_3CN), LC-MS trace.

^{1}H NMR (400 MHz, Acetonitrile-d_3) δ 7.48 (d, J = 15.7 Hz, 1H), 7.33 (d, J = 8.6 Hz, 1H), 6.78 (t, J = 5.4 Hz, 1H), 6.74 (d, J = 2.8 Hz, 1H), 6.58 (d, J = 15.8 Hz, 1H), 6.56 – 6.49 (m, 3H), 3.61 – 3.48 (m, 10H), 3.47 – 3.31 (m, 13H), 2.06 (s, 3H), 1.68 (dq, J = 8.1, 6.7 Hz, 2H), 1.56 – 1.46 (m, 3H), 1.43 – 1.24 (m, 3H), 1.13 (dt, J = 10.3, 7.1 Hz, 12H).

2-H-Halo, compound **CA3**

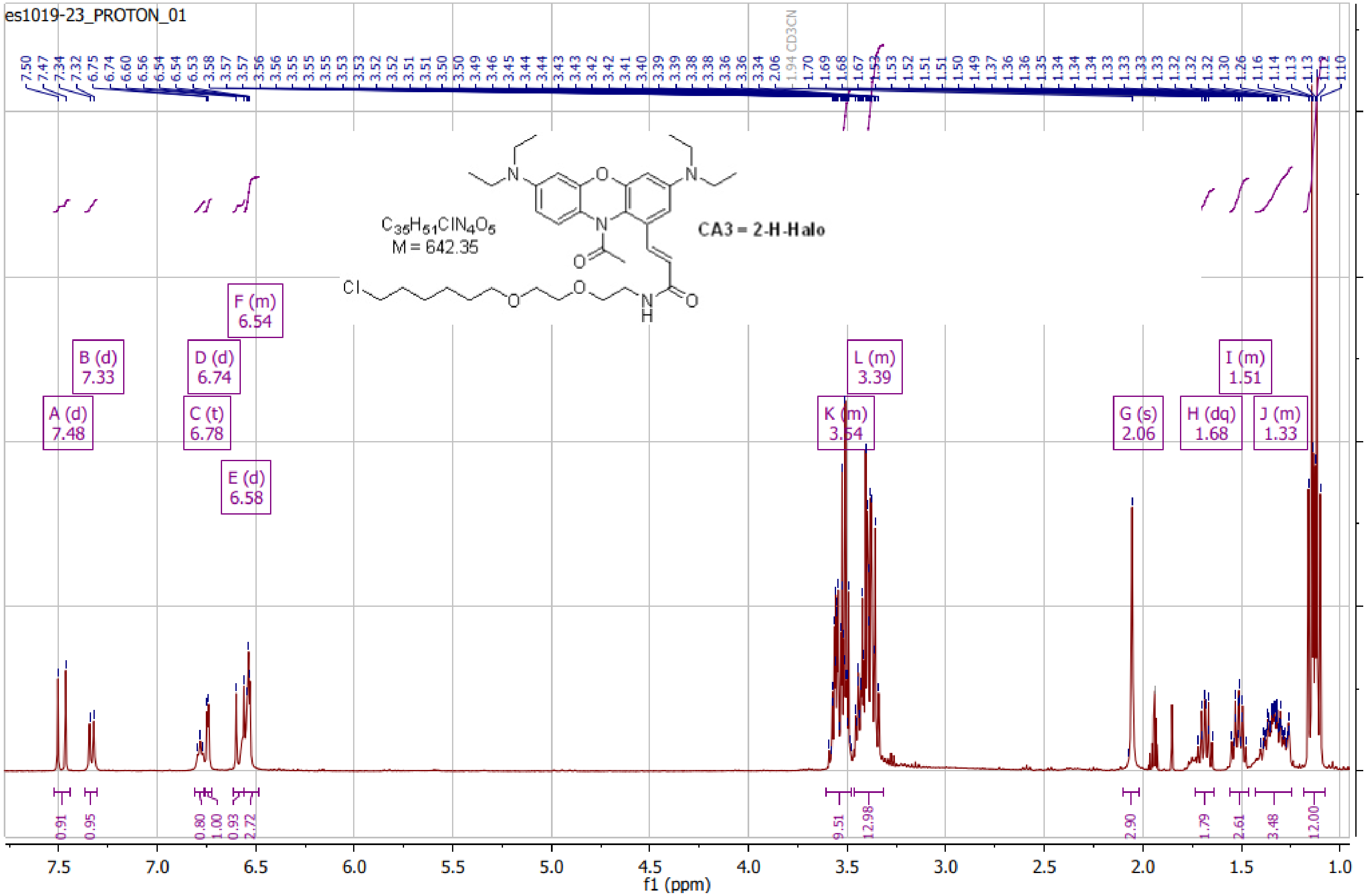

Probe : es1019-MeOH

Lösungsmittel : MeCN/H2O **Aufgabemenge:** 1.0 µl

Säule: Phenomenex Kinetex C18 1.7 µm **Länge:** 50 mm **iO :** 2.1 mm

Fluß (ml / Min) : 0.5 **Temperatur :** 25.0

Detektor: DAD-3000 **Pumpe:** HPG-3200SD **Sampler:** WPS-3000

Laufmittel: **A = Acetonitril 0.1% FA** **B = Wasser 0.1% FA**

Gradient: A 20.0 % B 80.0 % ----> A 100.0 % B 0.0 % T = 4 Min.

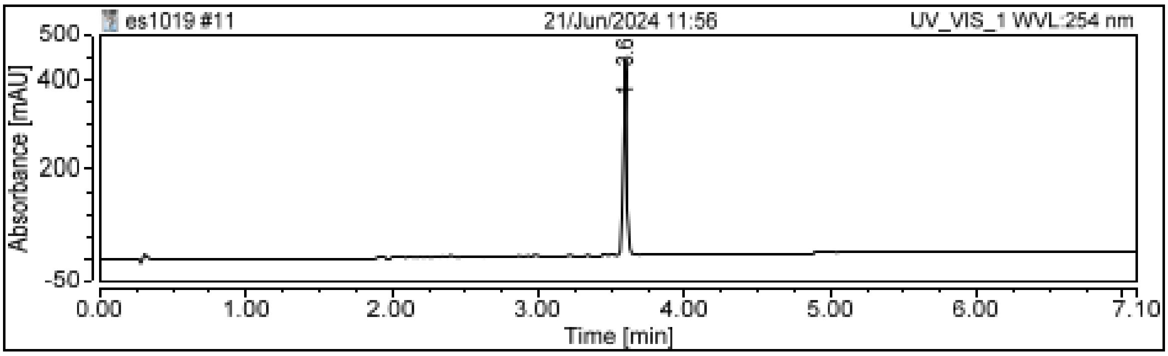

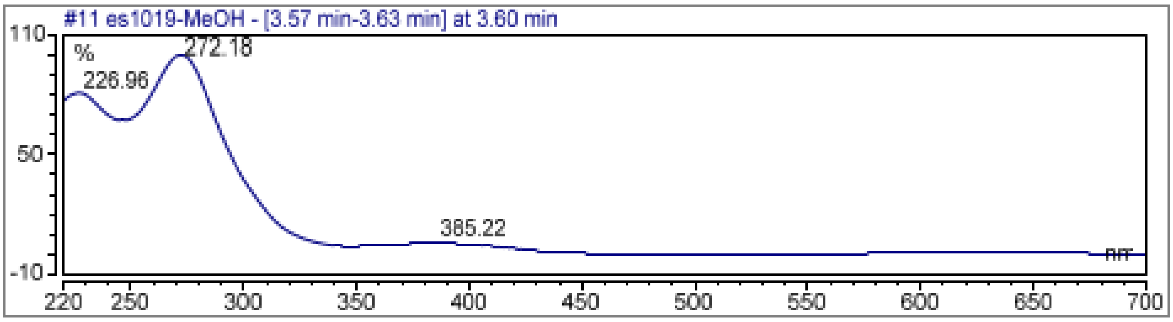

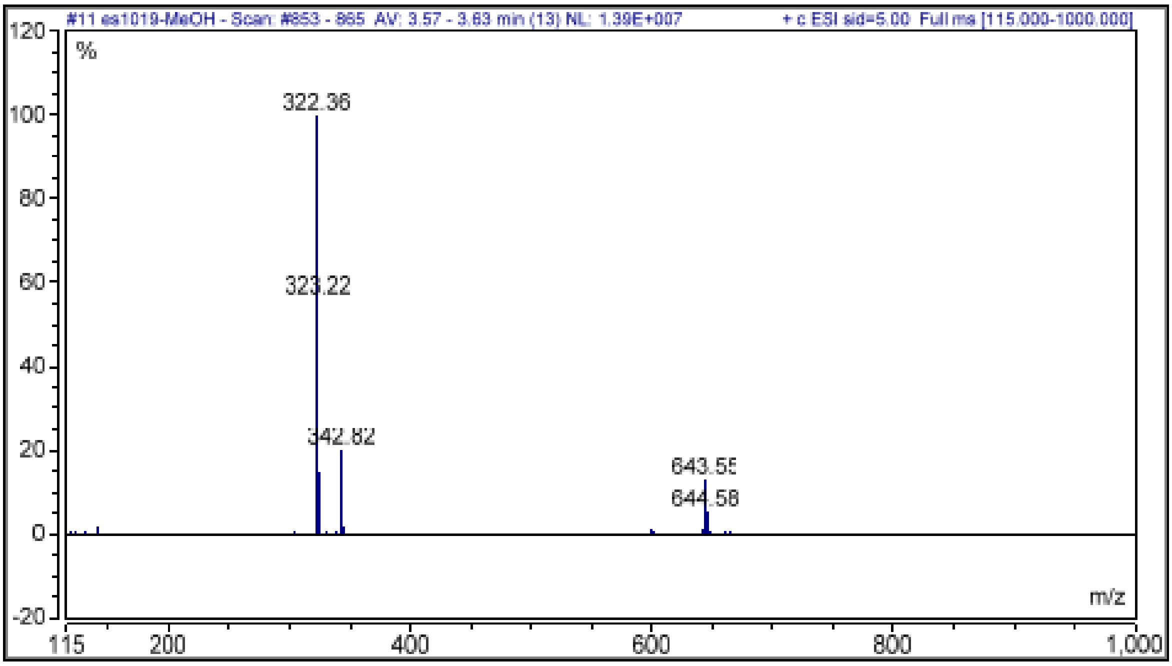

Probe **8a**-Halo = **CA1**. LC-MS trace and ^{1}H-NMR (CD_3CN).

Probe : es1008-43

Lösungsmittel : MeCN/H2O **Aufgabemenge:** 2.0 µl
Säule: Phenomenex Kinetex C18 1.7 µm **Länge:** 50 mm **iO :** 2.1 mm
Fluß (ml / Min) : 0.5 **Temperatur :** 25.0
Detektor: DAD-3000 **Pumpe:** HPG-3200SD **Sampler:** WPS-3000
Laufmittel: **A = Acetonitril 0.1% FA** **B = Wasser 0.1% FA**
Gradient: A 5.0 % B 95.0 % ----> A 50.0 % B 50.0 % T = 4 Min.

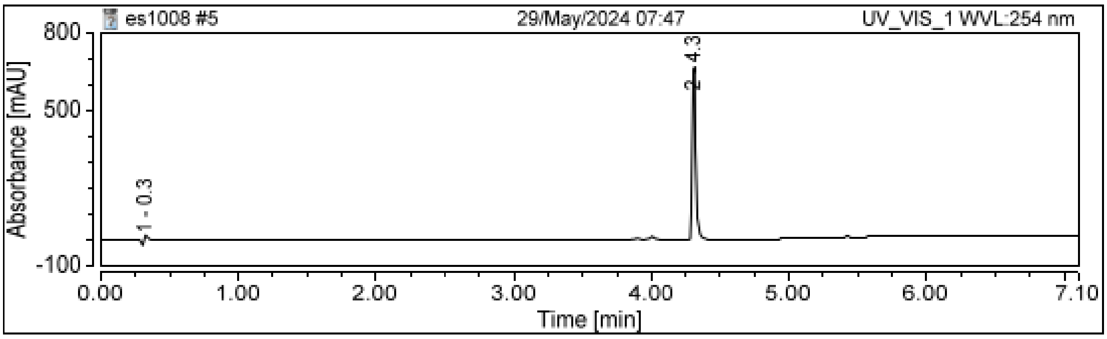

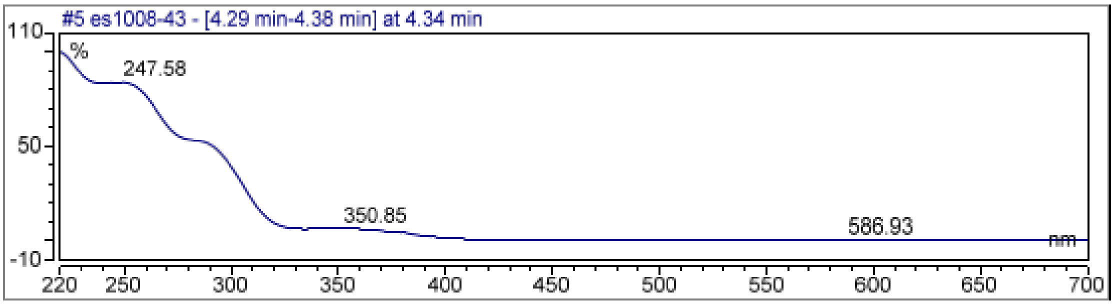

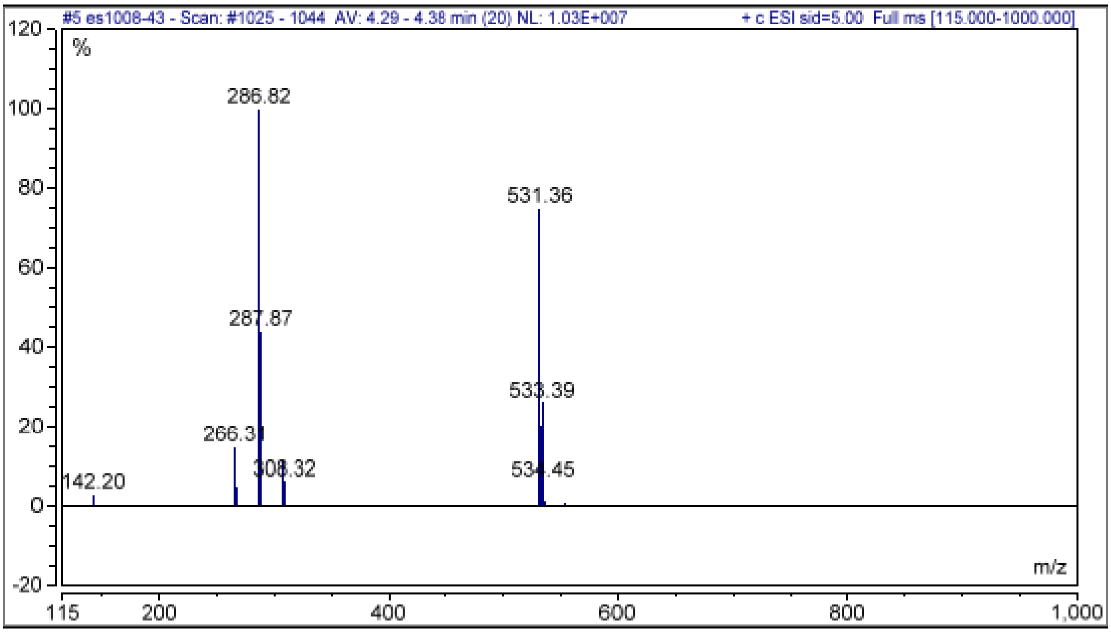

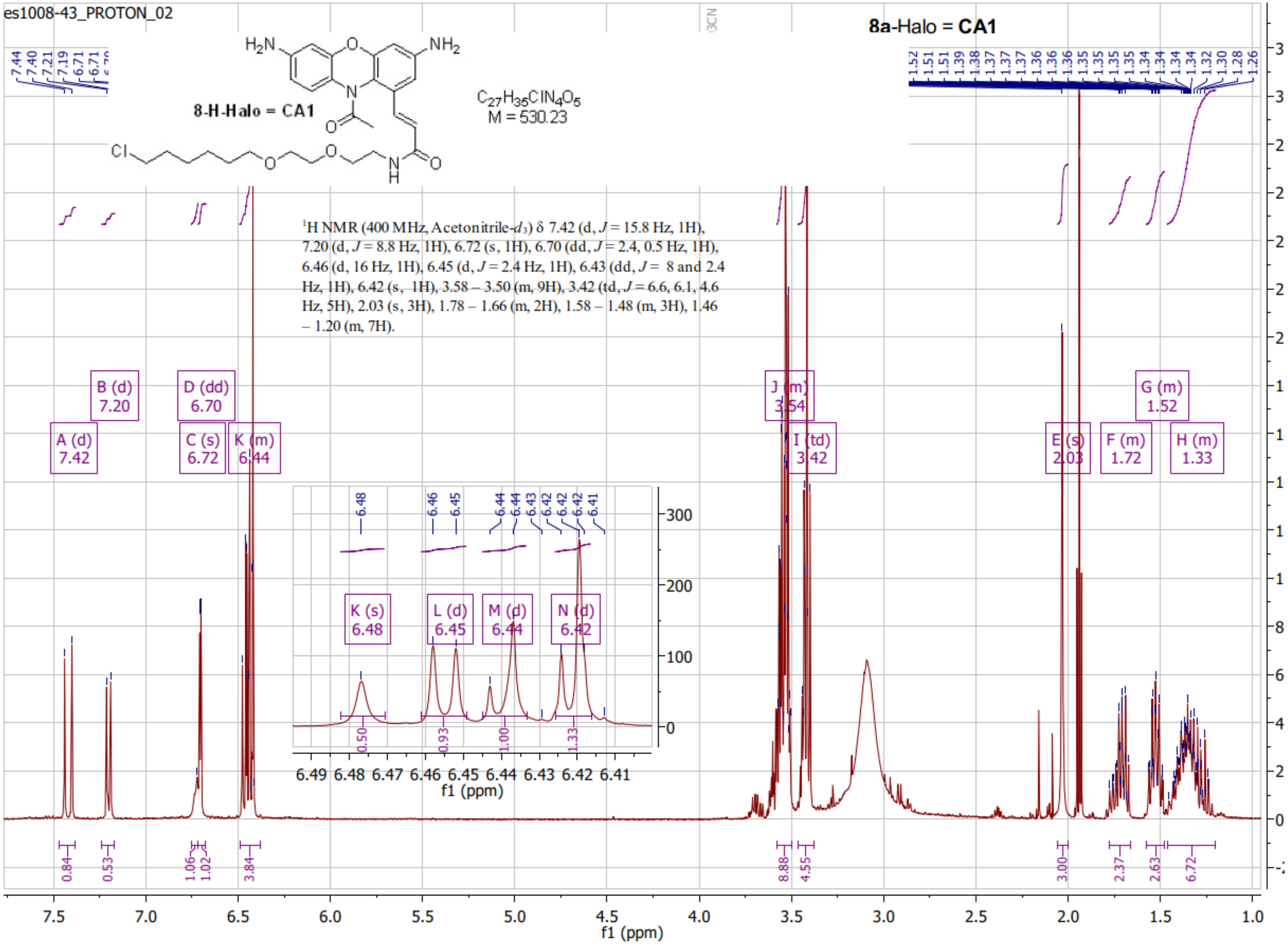
es1008-43_PROTON_02
8a-Halo = CA1
8-H-Halo = CA1
C27H35ClN4O5
M = 530.23
¹H NMR (400 MHz, Acetonitrile-d3) δ 7.42 (d, J = 15.8 Hz, 1H), 7.20 (d, J = 8.8 Hz, 1H), 6.72 (s, 1H), 6.70 (dd, J = 2.4, 0.5 Hz, 1H), 6.46 (d, 16 Hz, 1H), 6.45 (d, J = 2.4 Hz, 1H), 6.43 (dd, J = 8 and 2.4 Hz, 1H), 6.42 (s, 1H), 3.58 – 3.50 (m, 9H), 3.42 (td, J = 6.6, 6.1, 4.6 Hz, 5H), 2.03 (s, 3H), 1.78 – 1.66 (m, 2H), 1.58 – 1.48 (m, 3H), 1.46 – 1.20 (m, 7H).
A (d) 7.42
B (d) 7.20
C (s) 6.72
D (dd) 6.70
K (m) 6.44
J (m) 3.54
I (td) 3.42
E (s) 2.03
F (m) 1.72
G (m) 1.52
H (m) 1.33
K (s) 6.48
L (d) 6.45
M (d) 6.44
N (d) 6.42
f1 (ppm)
f1 (ppm)

^{1}H NMR (400 MHz, Acetonitrile-d_3) δ 7.45 (d, J = 15.7 Hz, 1H), 7.29 (d, J = 8.6 Hz, 1H), 6.74 (s, 1H), 6.68 (d, J = 2.5 Hz, 1H), 6.53 (d, J = 15.7 Hz, 1H), 6.48 – 6.41 (m, 3H), 3.60 – 3.50 (m, 9H), 3.43 (dt, J = 8.7, 6.1 Hz, 4H), 2.79 (s, 3H), 2.77 (s, 3H), 2.04 (s, 3H), 1.70 (dq, J = 8.1, 6.7 Hz, 2H), 1.52 (dq, J = 7.8, 6.7 Hz, 2H), 1.42 – 1.29 (m, 3H).

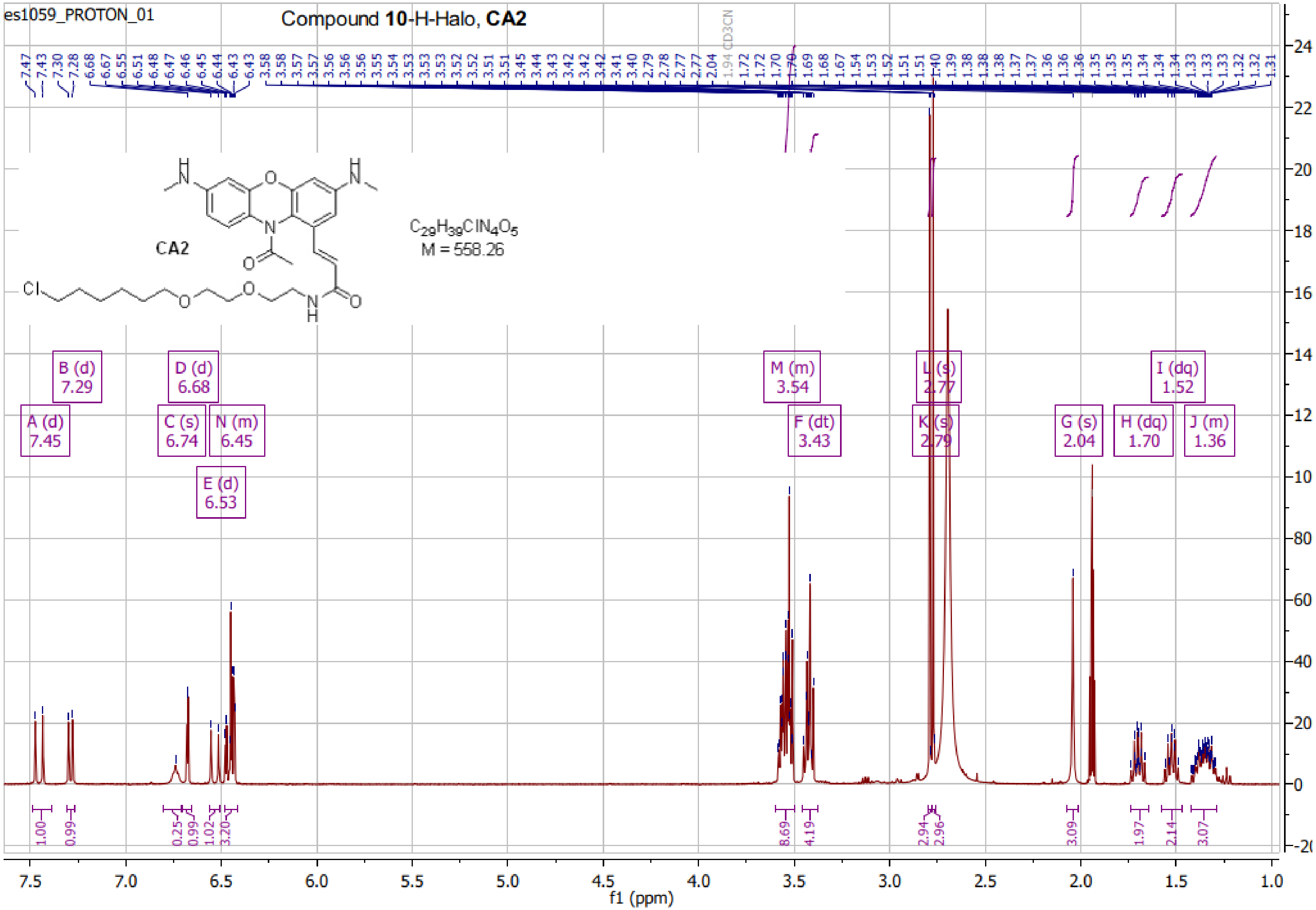

Probe **10-**H-Halo = **CA2**. ^{1}H-NMR (CD_3CN) and LC-MS trace.

Probe : es1059-37 rein

**Lösungsmittel :**	MeCN/H2O		**Aufgabemenge:**	5.0 µl	
**Säule:**	Phenomenex Kinetex C18	1.7 µm	**Länge:** 50 mm	**iO :**	2.1 mm
**Fluß (ml / Min) :**	0.5	**Temperatur :**	25.0		
**Detektor:**	DAD-3000	**Pumpe:**	HPG-3200SD	**Sampler:**	WPS-3000
**Laufmittel:**	**A = Acetonitril 0.1% FA**			**B = Wasser 0.1% FA**	
**Gradient:**	A 20.0 % B 80.0 %	---->	A 100.0 % B 0.0 %	T = 4 Min.	

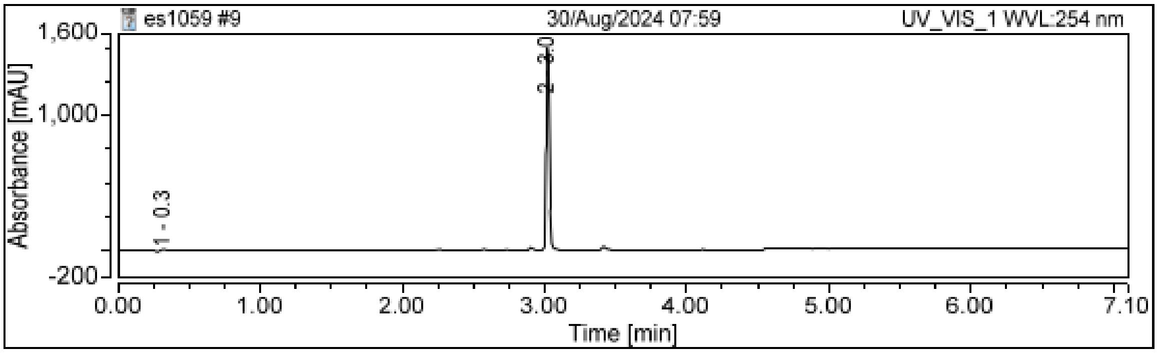

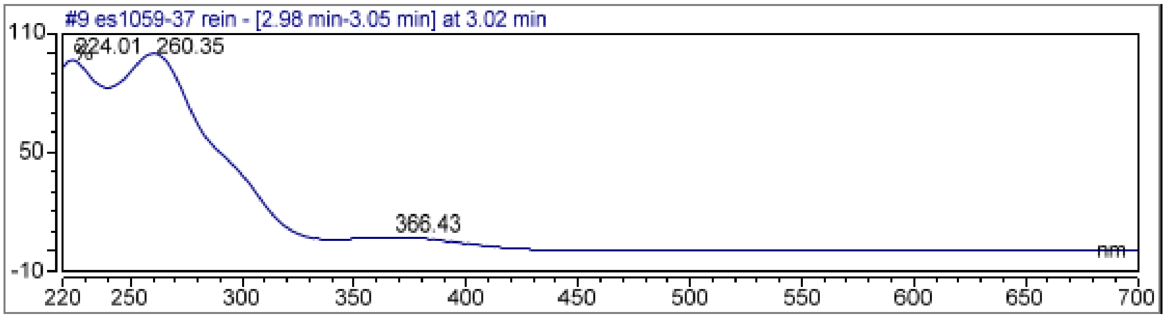

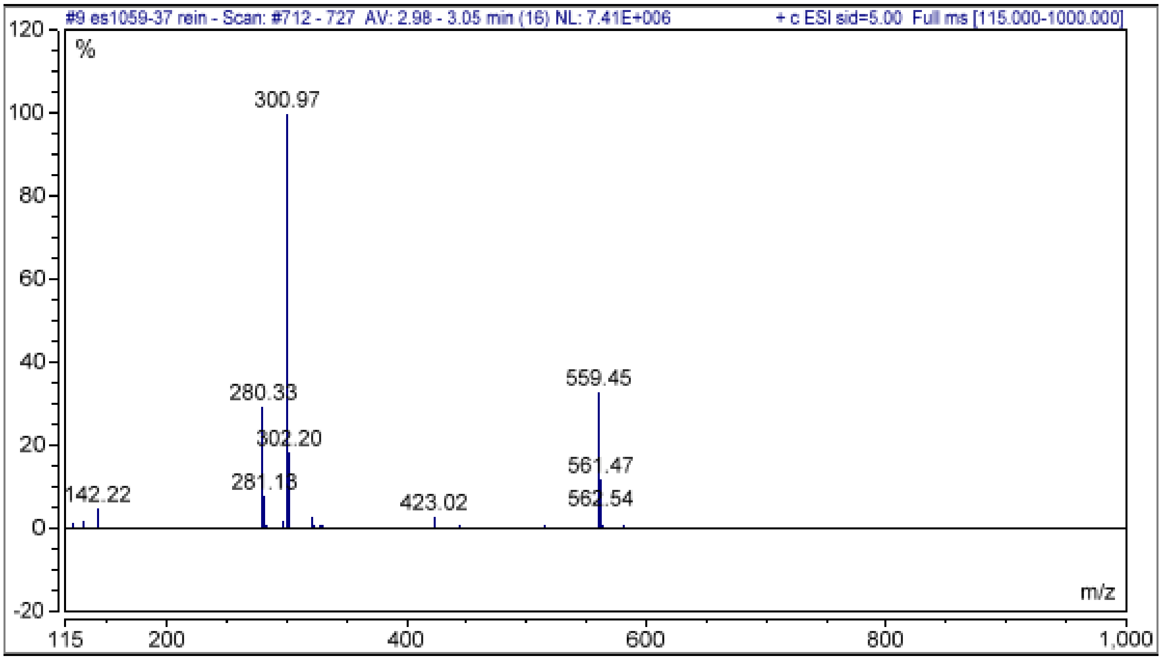

Probe **10**-Me-Halo = **CA4**. LC-MS trace and ^{1}H-NMR (CD_3CN).

Probe : es1080-2

Lösungsmittel : MeCN/H2O **Aufgabemenge:** 0.5 µl
Säule: Phenomenex Kinetex C18 1.7 µm **Länge:** 50 mm **iO :** 2.1 mm
Fluß (ml / Min) : 0.5 **Temperatur :** 25.0
Detektor: DAD-3000 **Pumpe:** HPG-3200SD **Sampler:** WPS-3000
Laufmittel: **A = Acetonitril 0.1% FA** **B = Wasser 0.1% FA**
Gradient: A 20.0 % B 80.0 % ----> A 100.0 % B 0.0 % T = 4 Min.

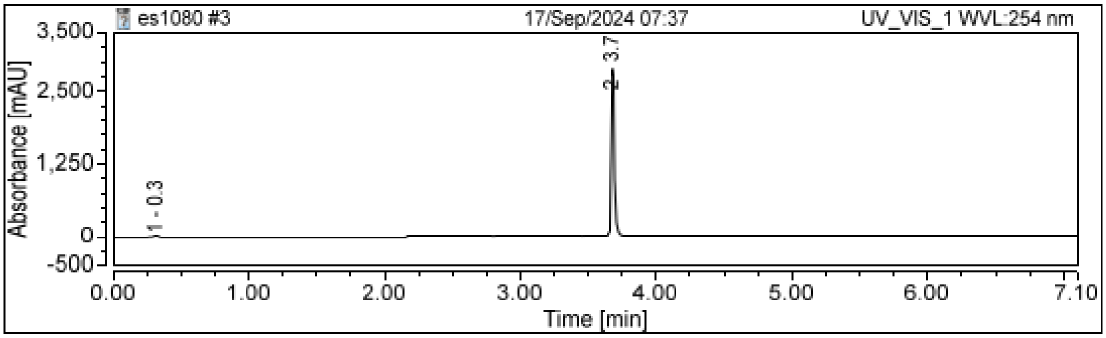

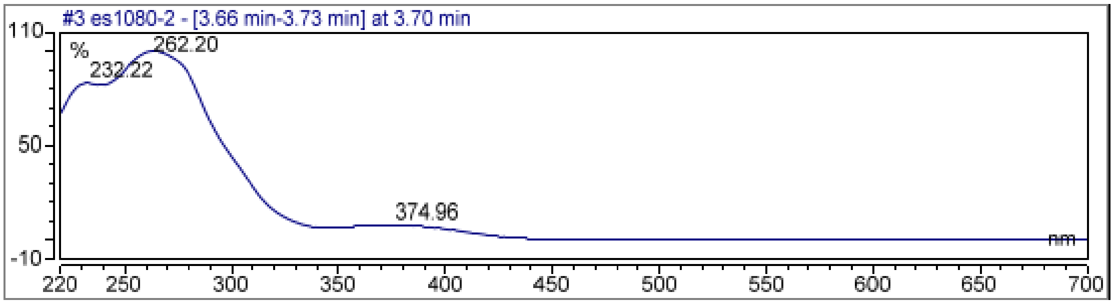

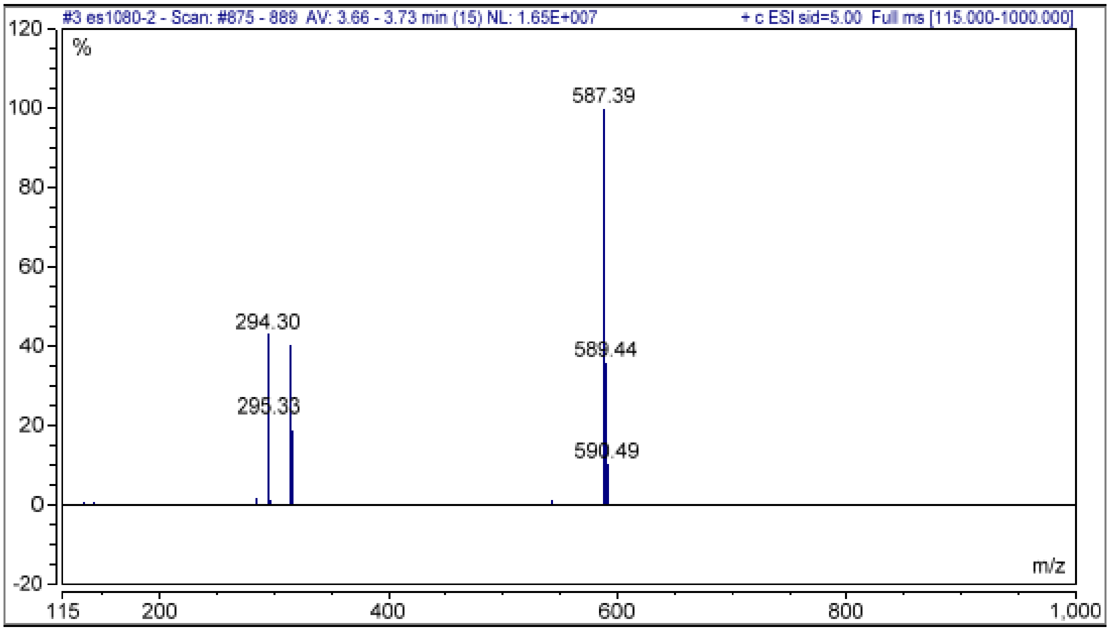

^{1}H NMR (400 MHz, Acetonitrile-d_3) δ 7.57 (d, J = 9.5 Hz, 1H), 7.50 (d, J = 15.8 Hz, 1H), 7.11 (d, J = 2.6 Hz, 1H), 7.04 (dd, J = 6.1, 2.8 Hz, 2H), 6.98 (t, J = 5.5 Hz, 1H), 6.87 (d, J = 2.6 Hz, 1H), 6.66 (d, J = 15.8 Hz, 1H), 3.62 – 3.50 (m, 9H), 3.46 (tt, J = 5.5, 2.3 Hz, 2H), 3.41 (t, J = 6.6 Hz, 2H), 3.08 (s, 6H), 3.05 (s, 7H), 2.07 (s, 3H), 1.69 (dq, J = 8.0, 6.6 Hz, 2H), 1.59 – 1.47 (m, 2H), 1.43 – 1.27 (m, 3H).

10-Me-Halo = CA4

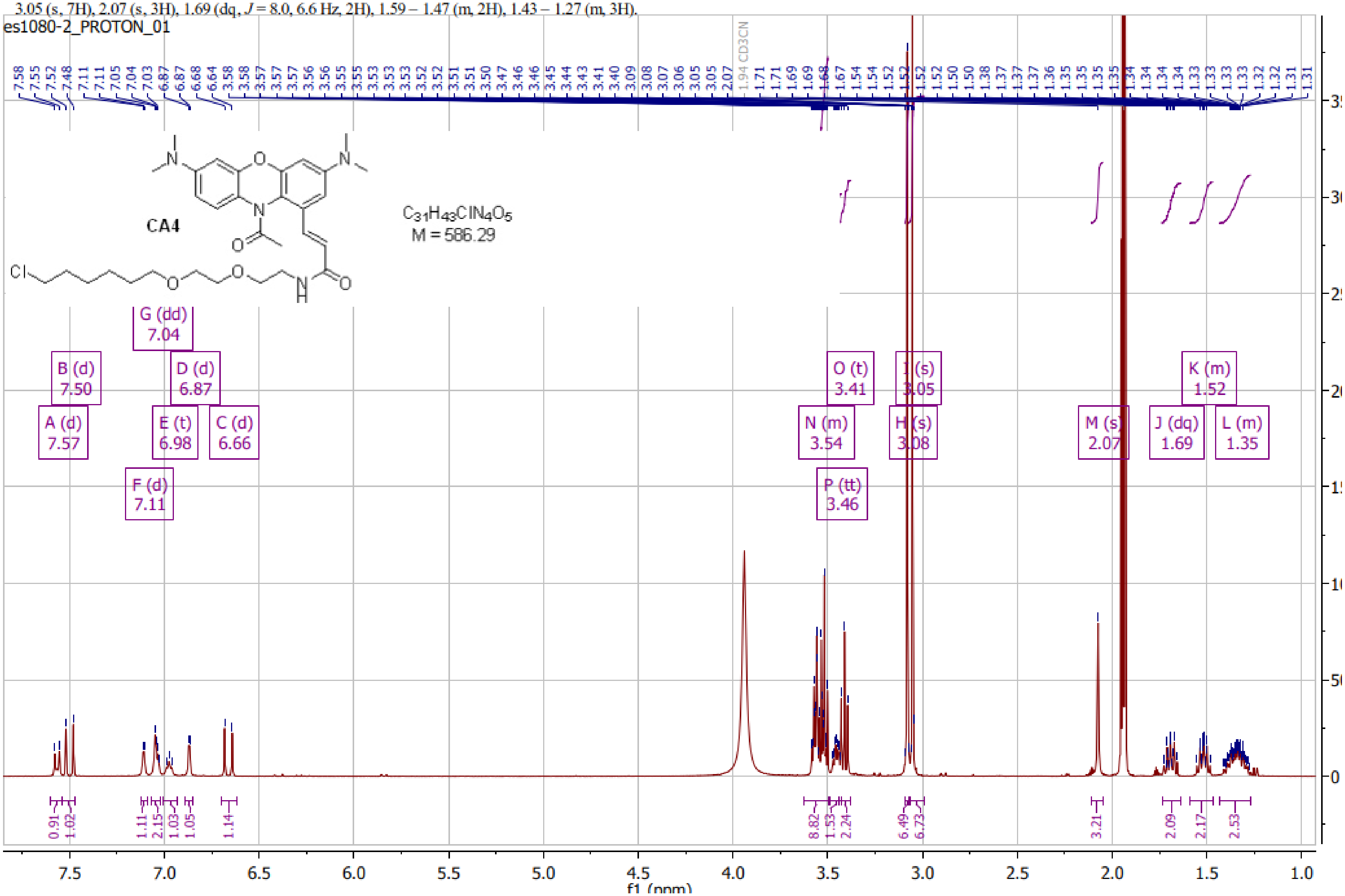

[i] K. Uno, M. L. Bossi, T. Konen, V. N. Belov, M. Irie, S. W. Hell, Asymmetric Diarylethenes with Oxidized 2-Alkylbenzothiophen-3-yl Units: Chemistry, Fluorescence, and Photoswitching, *Adv. Opt. Materials* **2019**, *7*, 1801746.
[ii] R. Lincoln, M. L. Bossi, M. Remmel, E. D'Este, A. N. Butkevich, S. W. Hell, A general design of caging-group-free photoactivatable fluorophores for live-cell nanoscopy, *Nat. Chem.* **2022**, *14*, 1013-1020.
[iii] A. N. Butkevich, H. Ta, Michael Ratz, S. Stoldt, S. Jakobs, V. N. Belov, S. W. Hell, Two-Color 810 nm STED Nanoscopy of Living Cells with Endogenous SNAP-Tagged Fusion Proteins, *ACS Chem. Biol.* **2018**, *13*, 475–480.
[iv] S. J. Sahl, J. Matthias, K. Inamdar, M. Weber, T. A. Khan, C. Brüser, S. Jakobs, S. Becker, C. Griesinger, J. Broichhagen, S. W. Hell, Direct optical measurement of intramolecular distances with angstrom precision, *Science* **2024**, 386, 180–187.
[v] M. S. Frei, M. Tarnawski, M. J. Roberti, B. Koch, J. Hiblot, K. Johnsson, Engineered HaloTag variants for fluorescence lifetime multiplexing, *Nat. Methods* **2022**, *19*, 65-70.
[vi] M. S. Frei, P. Hoess, M. Lampe, B. Nijmeijer, M. Kueblbeck, J. Ellenberg, H. Wadepohl, J. Ries, S. Pitsch, L. Reymond, K. Johnsson, Photoactivation of silicon rhodamines via a light-induced protonation. *Nat. Commun.* **2019**, *10*, 4580.
[vii] J. Nixon-Abell, C. J. Obara, A. V. Weigel, D. Li, W.R. Legant, C. S. Xu, H. A. Pasolli, K. Harvey, H. F. Hess, E. Betzig, C. Blackstone, J. Lippincott-Schwartz. Increased spatiotemporal resolution reveals highly dynamic dense tubular matrices in the peripheral ER. *Science* **2016**, *354*, 433-433.
[viii] M. Akiba, A. S. Dvornikov, P. M. Rentzepis, Formation of oxazine dye by photochemical reaction of *N*-acyl oxazine derivatives. *J. Photochem. Photobiol. A* **2007**, *190*, 69-76. DOI: 10.1016/j.photochem.2007.03.014.
